# Chemoselective Acylation of Nucleosides

**DOI:** 10.1002/chem.202201661

**Published:** 2022-07-26

**Authors:** Yu Tang, Rebecca L. Grange, Oliver D. Engl, Scott J. Miller

**Affiliations:** ^1^ Department of Chemistry Yale University New Haven CT 06520 USA; ^2^ Process Chemistry & Development Takeda Pharmaceuticals International Co. Cambridge MA 02139 USA

**Keywords:** acylation, chemoselectivity, nucleosides, synthetic methods

## Abstract

Acylated nucleoside analogues play an important role in medicinal chemistry and are extremely useful precursors to various other nucleoside analogues. However, chemoselective acylation of nucleosides usually requires several protection and deprotection steps due to the competing nucleophilicity of hydroxy and amino groups. In contrast, direct protecting‐group‐free chemoselective acylation of nucleosides is a preferred strategy due to lower cost and fewer overall synthetic steps. Herein, a simple and efficient chemoselective acylation of nucleosides and nucleotides under mild reaction conditions, giving either *O*‐ or *N*‐acylated products respectively with excellent chemoselectivity is reported.

## Graphical abstract

**Chemoselective functionalization** of oxygen atoms versus nitrogen atoms in bifunctional and polyfunctional molecules is a long‐standing challenge in both fundamental chemistry and in complex molecule synthesis. Different conditions that allow high selectivity in the derivatization of complex nucleosides that contain both *O*‐ and *N*‐based functionalities are described. Particularly intriguing cases are also presented, in which the reactivity of the *O*‐ and *N*‐based functionalities is quite context dependent, exhibiting reactivity that blurs conventional considerations, yet where selectivity may also be observed.

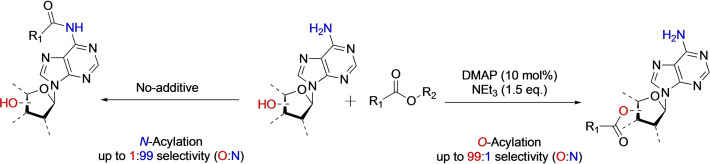

As the fundamental building blocks of RNA and DNA, nucleoside analogs can be incorporated in nucleic acids and prevent the normal replication of viruses and tumors.^[1]^ Thus, the synthesis and modification of nucleosides has attracted increasing attention in recent years.^[2]^ Acylated nucleosides are privileged scaffolds in medicinal chemistry and useful intermediates for the synthesis of other nucleoside derivatives, and acylating agents provide a convenient method for installing linkers that enable bioconjugation (Scheme 1a).^[3]^ Most nucleosides bear both amino and hydroxyl groups. To differentiate their reactivity, protecting groups are typically employed to induce acylation at the desired site on the nucleoside.^[3]^ While the direct chemoselective acylation of nucleosides remains underdeveloped,^[4]^ striking advances have been made, specifically in the area of chemoselective *O*‐ versus *N*‐acylation. The challenge is particularly interesting in light of the conventional wisdom around the greater reactivity of amines over alcohols under standard reactions conditions.^[5]^ That said, selective acylation via catalyst and reagent control has been achieved in certain settings, including striking recent work by Heller et al.,^[6]^ although the nucleoside context presents differing intrinsic reactivity hierarchies that are sparsely benchmarked.

**Scheme 1:**
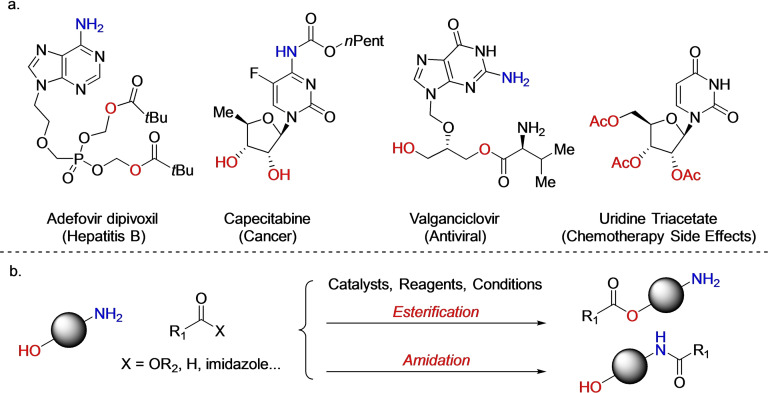
a. Selected bioactive nucleoside and nucleotide analogues bearing carbonyl groups. b. Chemoselective *O*‐ or *N*‐acylation of amino alcohols.

The era of protecting‐group‐free synthesis remains aspirational^[7]^ with even seemingly simple reactions such as acylation necessitating highly sophisticated methodological and physical organic studies.^[8]^ The chemoselective acylation of amino alcohols, which requires differentiation of amines and hydroxyl groups, is even more challenging.^[9]^ Due to the innate reactivities of amines and alcohols, the acylation of amino alcohols usually affords amides as the major products.^[10]^ Biocatalysts that reorder the reactivity hierarchy are available in certain cases^[11]^ and general protocols for selective *O*‐versus *N*‐acylation are gradually appearing in the literature. Metal complex‐catalyzed transesterifications[^5^, ^12^] and *N*‐heterocyclic carbene (NHC)‐catalyzed acylation of amino alcohols signal great promise,^[13]^ as do the recently reported conditions of Heller,^[6]^ featuring the use of *N*‐acylimidazole as an acylation reagent in the presence or absence of additives to achieve excellent chemoselectivity (Scheme 1b). Most broadly, the topic of chemoselective *O*‐ versus *N*‐acylation has recently been reviewed.^[14]^ However, the realization of direct chemoselectivity within complex nucleosides, in which the relative reactivity hierarchies of *O*‐ versus *N*‐based functional groups are blurred due to the attenuated nucleophilicity of heteroaryl amines, has not been achieved. Intriguingly, the ability to differentiate enantiomeric hydroxy or amino groups in acylative kinetic resolutions is arguably more advanced than capabilities for discriminating between amino and hydroxy functionalities in chemoselective acylations of amino alcohols.^[15]^ Herein we report our progress along these lines, culminating in the selective acylation of nucleosides under simple, complementary conditions: catalyst free *N*‐acylation and DMAP‐catalyzed *O*‐acylation.

We began our experiments with the speculative hypothesis that *N*‐methylimidazole (NMI) or 1,8‐diazabicycloundec‐7‐ene (DBU) might exhibit differential selectivities, as a function of their different reactivities and potentially different mechanisms of action. The literature, for example, includes evidence in support of each Lewis base functioning as a nucleophilic catalyst, although mechanisms involving noncovalent catalysis, such as general base or hydrogen bonding catalysis, are difficult to exclude.[^16^, ^17^, ^18^] Our study was thus initiated using 2‐deoxyadenosine‐derived nucleotide analog **1 a** as the substrate. With triethylamine as a basic additive, the incorporation of NMI or DBU as potential nucleophilic catalysts revealed a striking preference for the *O*‐acylated product, ester **3 a** (Table 1, entries 1 and 2), albeit with modest selectivities in the 4 : 1 to 2 : 1 range. However, use of the more potent nucleophilic catalyst DMAP provided **3 a** with 99 : 1 selectivity at moderate conversion (Table 1, entry 3), despite the presence of the highly reactive amino group. Notably, these reactions are quite clean, and isolated yields were subsequently found (see below) to track well with conversions. The conversion reached 75 % with addition of 1.2 equiv of benzoic anhydride (Bz_2_O, **2 a**), while the *O*‐selectivity was maintained (Table 1, entry 4). Comparable conversions and selectivities were observed when the DMAP loading was reduced to 10 % (Table 1, entry 5, henceforth referred to as Condition A), while further increasing the amount of Bz_2_O (**2 a**) did not improve the reaction efficiency (Table 1, entry 6). In addition, the exclusion of base slightly lowered the conversion and substantially diminished the chemoselectivity (70 : 30, Table 1, entry 7); this observation might relate to acid‐base chemistry involving the hydroxyl group (see below). Critically, exclusion of the nucleophilic catalyst, in the presence of NEt_3_, reveals inverted chemoselectivity, favoring *N*‐benzoylated **4 a** as the major product, as one might expect based on anticipated intrinsic reactivity (Table 1, entry 8). Moreover, decreasing the p*K*_b_ of the amine base substantially increased the *N*‐selectivity (Table 1, entries 8–10). We then tested the background reaction without any additives, and discovered that **4 a** was obtained in 1 : 99 *O* : *N* selectivity, with 17 % yield over 24 h (Table 1, entry 11). The optimization of the reaction conditions showed that higher temperature, appropriate concentration and carefully tuned Bz_2_O (**2 a**) stoichiometry can all contribute to improved efficiency (Table 1, entries 12–15); under these conditions, some bis‐benzoylated side product is also observed after 16 h (Table 1, entry 14). Ultimately, we settled on the use 1.5 equiv of Bz_2_O (**2 a**) at 50 °C for 16 h to offer *N*‐ benzoylated **4 a** in 53 % conversion without loss of site‐selectivity (Table 1, entry 13, henceforth referred to as Condition B). Overall, we identified catalyst‐controlled *O*‐acylation of **1 a** to give **3 a**, overturning the intrinsic, and optimizable reactivity preference of **1 a** to give *N*‐acylated product **4 a** under catalyst‐ and additive‐free conditions.

**Table 1:** Optimization of the chemoselective acylation.

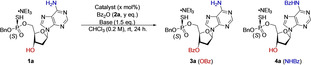					
Entry	Catalyst (20 mol %)	Base (1.5 equiv)	Bz_2_O (**2 a**, equiv)	Conv. [%]^[b]^	Ratio (*O : N*)^[b]^
1	NMI	NEt_3_	1.0	52	78 : 22
2	DBU	NEt_3_	1.0	52	63 : 37
3	DMAP	NEt_3_	1.0	64	99 : 1
4	DMAP	NEt_3_	1.2	73	99 : 1
**5** ^[c]^	**DMAP**	**NEt_3_**	**1.2**	**75**	**99 : 1**
6	DMAP	NEt_3_	1.4	75	99 : 1
7^[d]^	DMAP	/	1.2	65	70 : 30
8	/	NEt_3_	1.0	22	41 : 58
9	/	NMM	1.0	29	14 : 86
10	/	PhNMe_2_	1.0	25	6 : 94
11	/	/	1.0	17	1 : 99
12^[e]^	/	/	1.5	32	2 : 98
**13** ^[f]^	**/**	**/**	**1.5**	**53**	**1 : 99**
14^[g]^	/	/	1.5	57	2 : 98
15^[h]^	/	/	1.5	49	2 : 98

[a] **1 a** (0.05 mmol), Bz_2_O (0.05 mmol, 1.2 equiv), catalyst (0.01 mmol, 20 mol %), base (0.075 mmol, 1.5 equiv), CHCl_3_ (0.25 mL, 0.2 M), rt, 24 h. [b] determined by LC‐MS. [c] DMAP (10 mol %). [d] 16 % bis‐benzoylated product. [e] 50 °C for 8 h. [f] 50 °C for 16 h. [g] 50 °C for 24 h, 3 % bis‐benzoylated product was observed. [h] CHCl_3_ (0.5 M) as the solvent, 50 °C for 8 h. NMI=*N*‐methylimidazole, DBU=1,8‐diazabicycloundec‐7‐ene, DMAP=4‐dimethylaminopyridine, NMM=*N*‐methylmorpholine.

To probe the origin of the site selectivity, we conducted several competition experiments to compare the reactivity between various amines and alcohols (Scheme 2), and some striking subtlety was observed. In the presence of equimolar cyclopentanol (**6**) and cyclohexylamine (**7**), both condition A (with DMAP catalyst) and condition B (no additive) gave amide **9** as the major product (Scheme 2, equation 1), reflecting the anticipated intrinsic reactivity, and the inability of DMAP to effectively overturn the reactivity hierarchy. On the other hand, a competition between cyclopentanol (**6**) and the less nucleophilic aniline (**10**) produced ester **8** and amide **11** equally with DMAP as the catalyst; catalyst‐ and additive‐free condition B still favored the amidation product **11** (Scheme 2, equation 2), suggesting that DMAP is capable of inverting the intrinsic reactivity hierarchy, to at least some extent. Most dramatically, the competition between cyclopentanol (**6**) and 2‐aminopyridine (**12**) produces a result that mirrors our observations with nucleoside **1 a**: divergent chemoselectivity was observed under condition A and B respectively (Scheme 2, equation 3), with the catalyst favoring formation of ester **8** significantly (95 : 5), while the intrinsic reactivity conditions favor formation of the *N*‐acylated product **13**. Consistent with the prevailing wisdom about the mechanism of DMAP‐catalyzed acylation of alcohols,^[16c]^ it seems likely that the illustrated [DMAP‐Bz]^+^ cationic intermediate (Scheme 2, **I**) is more oxophilic, in concert with the role of the counteranion benzoate as a Brønsted base (or H‐bond acceptor), further enhancing the nucleophilicity of the more acidic O−H group through general base activation. In the absence of any basic additives, raw functional group nucleophilicity could govern the chemoselectivity, and acylation of the more nucleophilic amino group is favored under condition B (Scheme 2, **II**). It is also striking that in the series of nucleophilic amines – **7–10‐12** – the trend reveals a progression of the nucleophilic *N*‐atom to become a more *“O*‐like” nucleophile in terms of the decreasing preference of *N*‐ over *O*‐reactivity. Accordingly, the impact of catalysis by DMAP is correspondingly more readily observed.

**Scheme 2:**
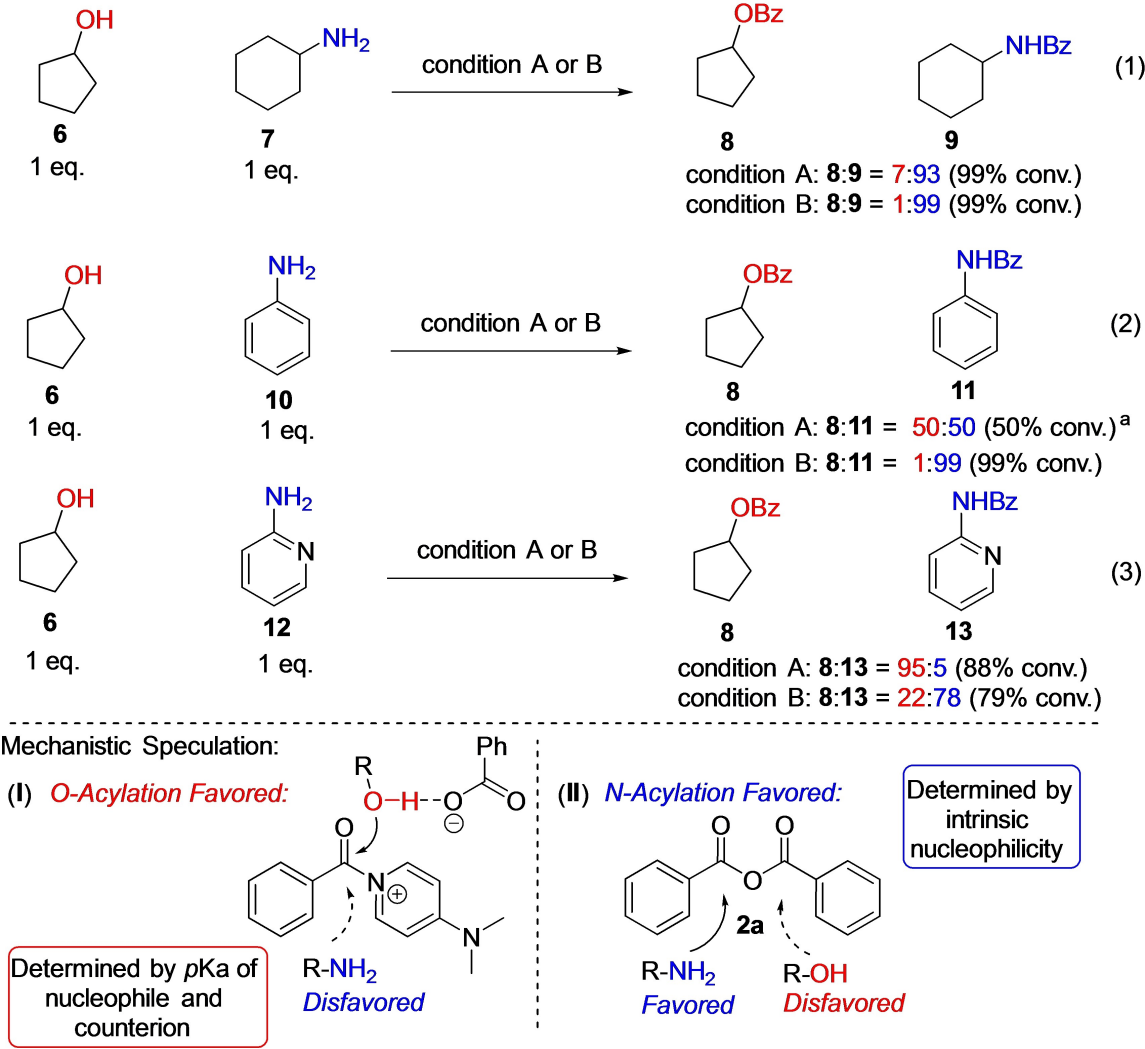
Condition A: Bz_2_O (**2 a**; 1.0 equiv), DMAP (10 mol %)/NEt_3_ (1.5 equiv), CDCl_3_ (0.2 M), rt, 24 h. Condition B: Bz_2_O (**2 a**, 1.0 equiv), CDCl_3_ (0.2 M), 50 °C, 24 h. The ratio was detected by ^1^H NMR. [a] 50 % conversion of **6**.

With this knowledge in hand, we set out to examine the substrate scope for other nucleosides. Using optimized condition A (targeting *O*‐acylation; Table 1, entry 5), along with optimized condition B (targeting *N*‐acylation; Table 1, entry 13), we investigated various nucleoside derivatives (Scheme 3). Nucleotide analogs **1 a** and **1 b**, differing only in their stereochemistry at the phosphorous atom, gave similar yield and chemoselectivity with **1 a** under both conditions A and B, delivering the same chemoselectivity under the specified conditions. So too, phosphoric acid **1 c** gave a very similar result, revealing that the phosphorothioate or phosphoric acid moieties are, at a minimum, well‐tolerated under the complementary reaction conditions; that said, their mechanistic engagement was not fully explored. Since the thiophosphoryl or phosphoryl moiety could participate in acylation (by forming active mixed anhydride intermediates from Bz_2_O, **2 a**), we also tested the *tert*‐butyldimethylsilyl (TBS) protected 2‐deoxyadenosine analog **1 d** under both conditions. Indeed, the complementary chemoselectivity is once again nearly identical to that observed for substrate **1 a**, indicating once again that the phosphate moiety is not required for chemoselectivity. Adenosine derivative **1 e** gave **3 e** and **4 e** in 99 : 1 and 8 : 92 chemoselectivity under the respective complementary conditions. It seems plausible that the slightly lower *N*‐selectivity could reflect the higher reactivity of the primary alcohol. As expected, substrate **1 f**, with the hydroxy group vicinal to the adenine substituent maintained the excellent chemoselectivity (99 : 1, Condition A; and 2 : 98, Condition B). To further expand the applicability of this method, other nucleosides were also examined under both conditions. Surprisingly, *N*‐benzoylated 2‐deoxycytidine analog **4 g** was obtained exclusively in 97 % yield without any additives (condition B), indicating the superior nucleophilicity of the cytosine moiety (similar to aniline **10**, Scheme 2, equation 2). Thus, under the catalysis of DMAP, −OH and −NH_2_ have comparable reactivity (Scheme 2, equation 2, condition A), offering bis‐benzoylated product **5 g** in 42 % yield. On the other hand, 2‐deoxyguanosine derivative **1 h** gave no *N*‐acylation product under condition B which might be due to the low nucleophilicity of its amino group, while the *O*‐acylation result was not affected.

**Scheme 3:**
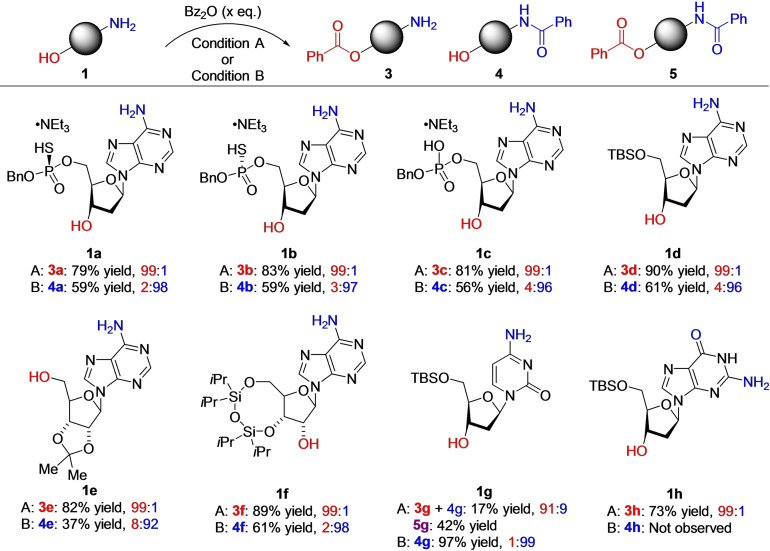
Investigation of nucleoside analogs. Condition A: **1** (0.1 mmol), Bz_2_O (**2 a**, 0.12 mmol, 1.2 equiv), DMAP (0.01 mmol, 10 mol %), NEt_3_ (0.15 mmol, 1.5 equiv), CHCl_3_ (0.5 mL, 0.2 M), rt, 16 h. Condition B: **1** (0.1 mmol), Bz_2_O (0.15 mmol, 1.5 equiv), CHCl_3_ (0.5 mL, 0.2 M), 50 °C, 16 h. The absolute configuration of **1 b** was determined by the single‐crystal X‐ray diffraction.

Other electrophiles were also tested under the chemodivergent conditions A and B (Scheme 4). Acetic anhydride showed higher reactivity than benzoic anhydride, providing high selectivity (99 : 1) for the *O*‐acylated product **3 ab** with condition A; Condition B afforded *N*‐acylated product **4 ab** with somewhat diminished selectivity (17 : 83 *N*‐selectivity). To extend this strategy to complex acyl groups, active esters were evaluated.^[19]^ Active ester **2 c**, derived from hydroxybenzotriazole (HOBt), gave moderate yield and good *O*‐ versus *N*‐selectivity under the respective conditions. Fluorinated aryl esters gave curious results. Acylation products were not detected with substrate **2 d**, but **2 e**, a perfluoro‐*p*‐cresol ester, delivered products with high chemoselectivity, albeit in low yields. We then examined HOBt‐derived active ester **2 f** to introduce an amino acid residue to the nucleoside analog **1 a** and obtained both ester and amide products in good yield and selectivity. Notably, HOBt has been known to form as mixtures of *O*‐ and *N*‐acylated active esters in the case of amino acids with α‐protons,^[20]^ so we also evaluated the perfluoro‐*p*‐cresol‐derived active ester **2 g** of a phenylalanine derivative, which gave the corresponding products in excellent yield. Peptide **2 h**, which could be seen as a model for a peptide‐nucleoside conjugate, was also tolerated in this protocol, and the corresponding ester and amide derivatives were obtained in good selectivity under the respective conditions (A, 93 : 7 *O : N*‐acylation; B, 12 : 88 *O : N*‐acylation), giving **3 ah** and **4 ah** in 43 % and 32 % isolated yield respectively.

**Scheme 4:**
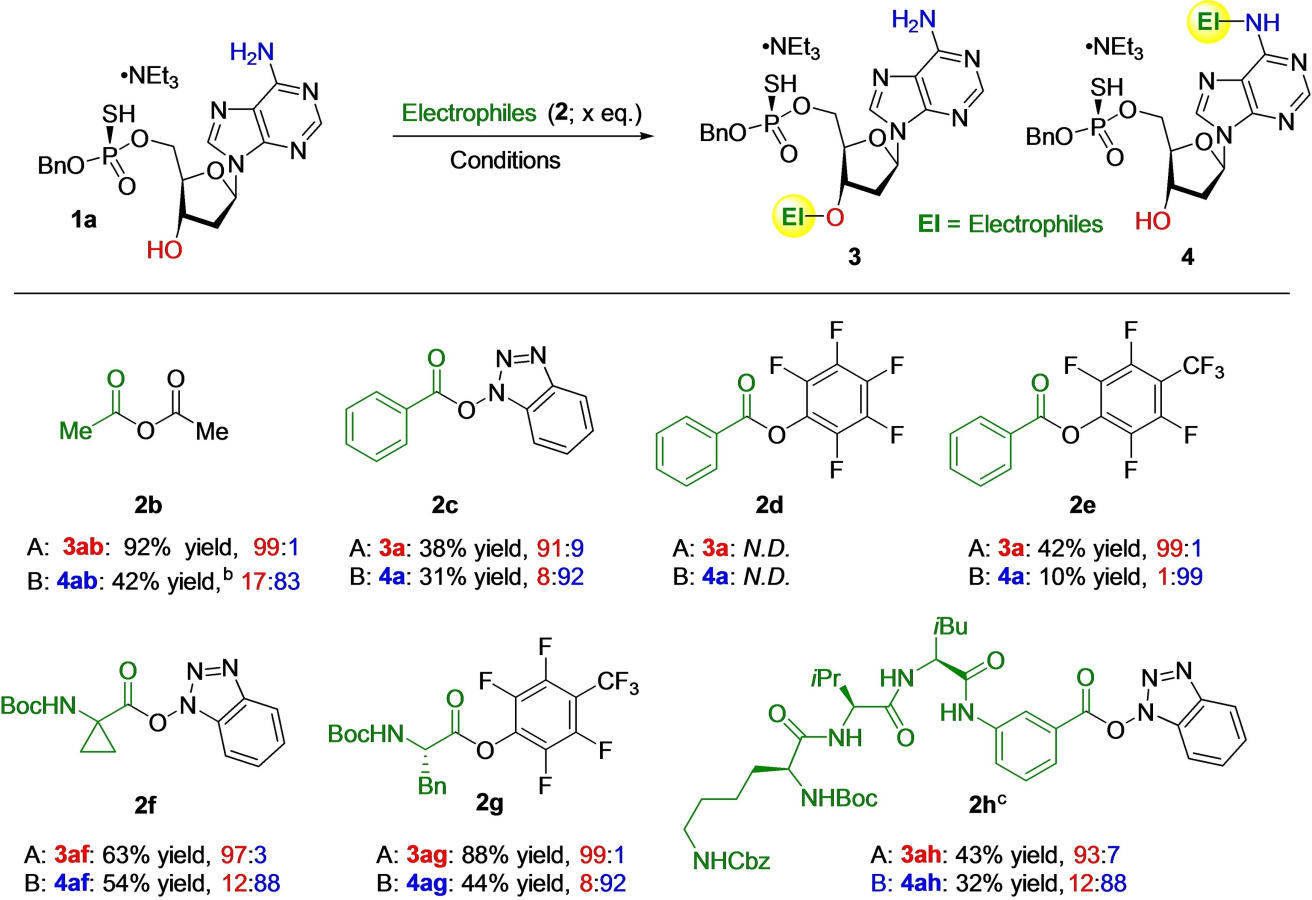
Investigation of the electrophiles. Condition A: **1 a** (0.1 mmol), **2** (0.12 mmol, 1.2 equiv), DMAP (0.01 mmol, 10 mol %), NEt_3_ (0.15 mmol, 1.5 equiv), CHCl_3_ (0.5 mL, 0.2 M), rt, 16 h. Condition B: **1 a** (0.1 mmol), **2** (0.15 mmol, 1.5 equiv), CHCl_3_ (0.5 mL, 0.2 M), 50 °C, 16 h. b. Yield was detected by ^1^H NMR of **2 b** and **4 ab** mixture. c. Conducted at 0.05 mmol scale.

Finally, since cytidine analogs remained recalcitrant substrates in our optimized Condition A and Condition B selective acylation protocols, we decided to examine substrate **1 g** under the conditions of Heller.^[6]^ Indeed, using *N*‐benzoylimidazole as the benzoylation reagent, pyridinium chloride and DBU additives produced the amide and the ester, respectively (Scheme 5, equation 1). However, for benzoic anhydride, pyridinium chloride and DBU both favored the formation of the amide (Scheme 5, equation 2). These results could relate to the counter anion effect postulated in the mechanistic speculation above (Scheme 2). That is, in comparison to benzoate anion, DBU is a stronger base and therefore may more readily activate the O−H bond, promoting esterification.^[6]^ On the other hand, given the fact that the catalysts are different (DBU vs. DMAP), it is surely possible that the relative reactivity of each *N*‐functionality is also a contributing factor. We also revisited 2‐deoxyadenosine analog **1 d** with benzoyl imidazole, and interestingly no product was observed with pyridinium chloride, while with DBU ester **3 d** was obtained in over 99 : 1 chemoselectivity and 43 % yield (Scheme 3, equation 3). As noted above (Table 1, entry 2), using Bz_2_O (**2 a**) as the acylation reagent with DBU and analog **1 a** gave 63 : 37 *O : N*‐selectivity. This result is consistent with our hypothesis that the more basic counter anion favors the activation of the O−H bond. More globally, these results signal that there is a complex interplay between the factors that contribute to chemoselective *O*‐ versus *N*‐acylation of various nucleosides.

**Scheme 5:**
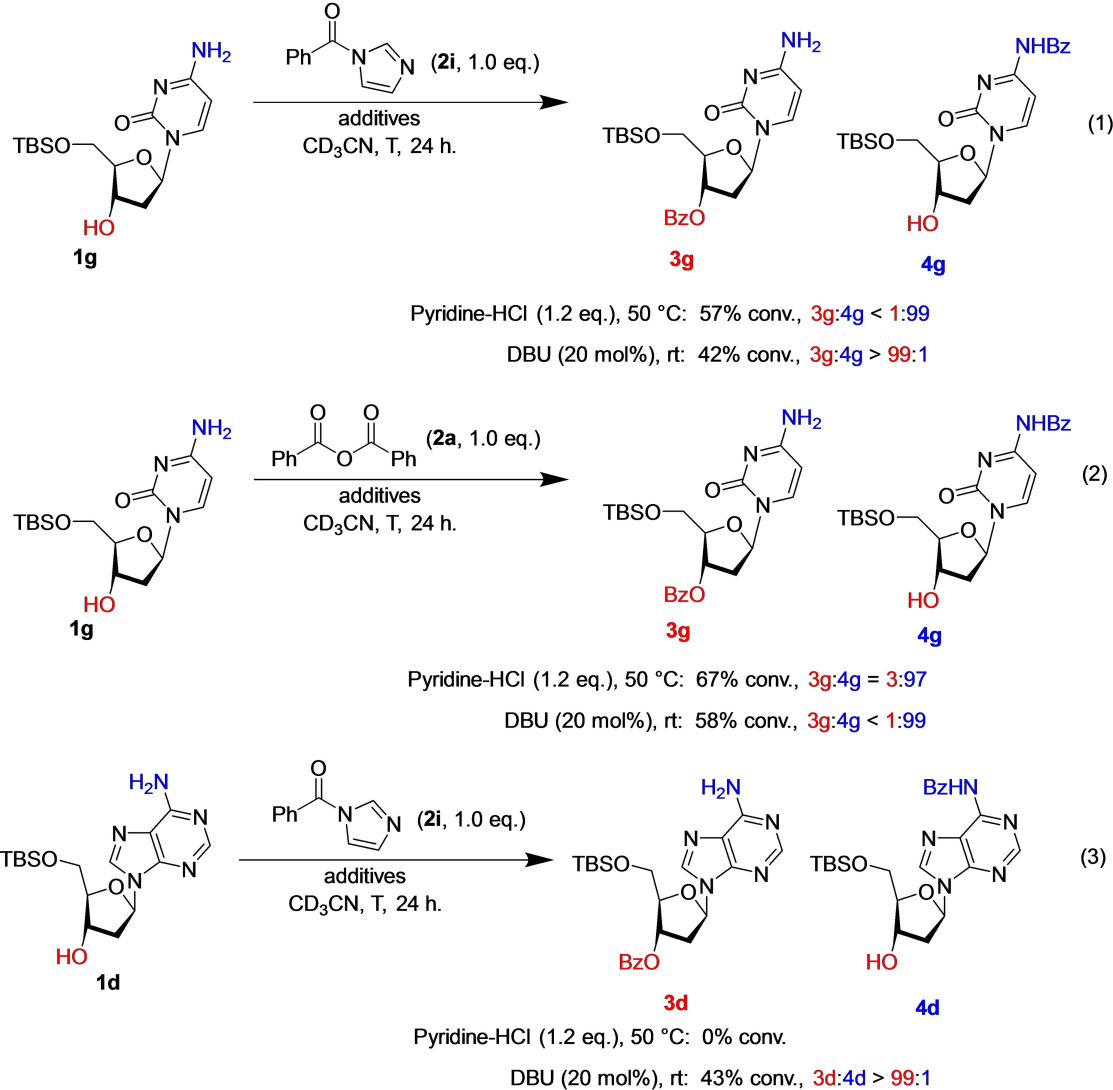
Chemoselective acylation of cytidine derivatives. Pyridinium chloride conditions: **1** (0.05 mmol), **2** (0.05 mmol, 1.0 equiv), Pyridine‐HCl (0.06 mmol, 1.2 equiv), CD_3_CN (0.25 mL, 0.2 M), 50 °C, 24 h. DBU conditions: **1** (0.05 mmol), **2** (0.05 mmol, 1.0 equiv), DBU (0.01 mmol, 20 mol %), CD_3_CN (0.25 mL, 0.2 M), rt, 24 h. The ratio was detected by ^1^H NMR.

In conclusion, we have developed a simple and efficient method for the chemoselective acylation of various nucleoside and nucleotide analogues. The method works for more sophisticated acyl groups, as was demonstrated by chemoselective delivery of several acylated amino acids and a more elaborate peptide sequence. Control experiments support the classical notion that selective *N*‐acylation is due to higher nucleophilicity of the amino group, while selective *O*‐acylation can become dominant with catalytic activation of O−H bond by counter anions associated with DMAP‐derived acyl pyridinium intermediates. Considerable nuance is observed, however, with various nucleosides, highlighting the specifically tuned reactivity of their nucleophilic sites, and the tunability of product ratios as a function of reaction conditions.

## Experimental Section

Deposition Number **1 b** contain the supplementary crystallographic data for this paper. These data are provided free of charge by the joint Cambridge Crystallographic Data Centre and Fachinformationszentrum Karlsruhe Access Structures service.

## Conflict of interest

The authors declare no conflict of interest.

## Supporting information

As a service to our authors and readers, this journal provides supporting information supplied by the authors. Such materials are peer reviewed and may be re‐organized for online delivery, but are not copy‐edited or typeset. Technical support issues arising from supporting information (other than missing files) should be addressed to the authors.

Supporting Information

## Data Availability

The data that support the findings of this study are available in the supplementary material of this article.
