# Supplementary material for: Chemoselective Acylation of Nucleosides
Source: Chemistry. 2022 Jul 26;28(52):e202201661. doi: 10.1002/chem.202201661 (PMC9481663; doi:10.1002/chem.202201661)
Supplement: Supplementary file 1 — Supporting Information [file CHEM-28-0-s001.pdf]

Chemistry–A European Journal

Supporting Information

Chemoselective Acylation of Nucleosides

Yu Tang, Rebecca L. Grange, Oliver D. Engl,* and Scott J. Miller*

Table of Contents

I. Materials and Methods S2
II. Synthetic Details S5
1. Synthesis of nucleoside analogs and active esters S5
2. General procedure and scope for the chemoselective acylation S15
3. Competition experiments S31
4. Crystal information S35
5. NMR spectra S37
6. Reference S105

I. Materials and Methods

General Information. Room temperature is generally considered 20–23 °C. All reactions were carried out without exclusion of air or moisture, unless otherwise stated. All commercially available reagents and solvents were obtained from common suppliers and used as received without further purification, unless otherwise indicated. Acetonitrile (MeCN), diethyl ether (Et_2O), dichloromethane (CH_2Cl_2), *N*,*N*-dimethylformamide (DMF), tetrahydrofuran (THF), and toluene (PhMe) were dried over alumina and dispensed under argon from a Seca Solvent purification system by GlassContour. Triethylamine (Et_3N) and *N*,*N*-diisopropyl ethylamine ($^iPr_2NEt$) were distilled over CaH_2 under a nitrogen atmosphere prior to use. Deionized water was used for reactions, extraction solutions, and reversed phase chromatography. HPLC grade solvents were used for all other chromatography.

Analytical Methods.

• **TLC and Column Chromatography:** Analytical thin-layer chromatography (TLC) was performed using EMD Millipore silica gel 60 F254 precoated plates (0.25 mm thickness). Normal phase column chromatography was conducted using an automated Biotage® Isolera™ One flash purification system equipped with a 10, 25, or 50 g SNAP Ultra (HP Sphere, 25-micron silica) cartridge. Reversed-phase column chromatography was performed using an automated Biotage® Isolera™ One flash purification system equipped with a 12, 30, 60, or 120 g SNAP C18 (HS 50-micron silica) or SNAP Ultra C18 (HP Sphere, 25-micron silica) cartridge.

• **NMR:** Unless otherwise stated, all NMR data were acquired at ambient temperature. NMR solvents, chloroform-*d* ($CDCl_3$), dimethylsulfoxide-d_6 (DMSO-d_6), methanol-d_4 (CD_3OD), and acetonitrile-d_3 (CD_3CN) were purchased from Cambridge Isotope Laboratories and used as received. DMSO-d_6 and CD_3OD ampules were used immediately upon opening. NMR spectra were processed with MestReNova software (v. 10.0.2) using the baseline and phasing correction features. Multiplicities and coupling constants were calculated using the multiplet analysis feature with manual intervention as necessary. ^{1}H NMR spectra were obtained on Agilent 400 MHz, 500 MHz, or 600 MHz spectrometers. Proton chemical shifts (δ) are reported in ppm and referenced to residual solvent peaks for $CDCl_3$ (δ 7.26 ppm), DMS*O*-d_6 (δ 2.50 ppm), and CD_3OD (δ 3.31 ppm).[1] Proton data are reported as chemical shift, multiplicity (noted as singlet (s), doublet (d), triplet (t), quartet (q), pentet (p), heptet (hept), multiplet (m), broad singlet (bs), doublet of doublets (dd), doublet of doublet of doublets (ddd), doublet of doublet of triplets (ddt), doublet of triplets (dt), doublet of triplet of triplets (dtt), etc.) coupling constants [Hz], and integration. ^{13}C NMR spectra were obtained on Agilent 400 (100) MHz, 500 (126) MHz, or 600 (150) MHz spectrometers with full proton decoupling. Carbon chemical shifts (δ) are reported in ppm and referenced to residual solvent peaks for $CDCl_3$ (δ 77.16 ppm), DMS*O*-d_6 (δ 39.52 ppm), and CD_3OD (δ 49.00 ppm) with multiplicity and coupling constants [Hz] indicated when present; ^{19}F

NMR spectra were obtained on Agilent 400 (376) MHz or 500 (471) MHz spectrometers without proton decoupling. Fluorine chemical shifts (δ) are referenced to $CFCl_3$ (δ 0.00 ppm) and were calibrated by the spectrometer using the solvent deuterium lock signal. Fluorine data are reported as chemical shift, multiplicity, coupling constant [Hz], and integration.

• **Infrared Spectroscopy:** Infrared spectra were recorded on a Shimadzu IRTracer-100/FT-ATR spectrometer, and select ν_{max} are reported in cm^{-1}.

• **Mass Spectrometry:** Ultra high-Performance Liquid Chromatography-mass spectrometry (UPLC/MS) was performed on a Waters Acquity SQD2 instrument equipped with an Ultra BEH C-18 column (1.7 µm particle size, 2.1 x 50 mm), a dual atmospheric pressure chemical ionization (API)/electrospray ionization (ESI) mass spectrometry detector, and a photodiode array detector. High-Resolution Mass Spectrometry (HRMS) was conducted by the Chemical and Biophysical Instrumentation Center in the chemistry department at Yale University, on a Waters Xevo Q-TOF high-resolution mass spectrometer using ESI.

Abbreviations

Ac	acetyl
Acpc	1-aminocyclopropane-1-carboxylic acid
aq	aqueous
Bn	benzyl
Bz	benzoyl
Boc	*tert*-butoxycarbonyl
^{t}Bu	*tert*-butyl
CV	column volume
Cbz	benzyl carbamate
DCM	dichloromethane
DIC	*N,N'*-diisopropylcarbodiimide
DMF	*N*,*N*-dimethylformamide
DMSO	dimethylsulfoxide
EDC•HCl	*N*-(3-dimethylaminopropyl)-*N'*-ethylcarbodiimide hydrochloride
eq.	equivalents
ESI	electrospray ionization
Et	ethyl
EtOAc	ethyl acetate
HOBt	1-hydroxybenzotriazole
HRMS	high-resolution mass spectrometry
IR	infrared

LCMS	liquid chromatography mass spectrometry
Me	methyl
NMR	nuclear magnetic resonance
Ph	phenyl
^{i}Pr	*iso*-propyl
RP	reversed-phase
rt	room temperature
sat	saturated
THF	tetrahydrofuran
TLC	thin-layer chromatography
TOF	time-of-flight
TBS	tert-butyl(dimethyl)silyl
UPLC-MS	ultra-performance liquid chromatography mass spectrometry

II. Synthetic Details

1. Synthesis of nucleoside analogs and active esters

***O*-(((2*R*,2*R*)-5-(6-amino-9*H*-purin-9-yl)-3-hydroxytetrahydrofuran-2-yl)methyl) *O*-benzyl (*S*)-phosphorothioate triethylammonium (1a)**

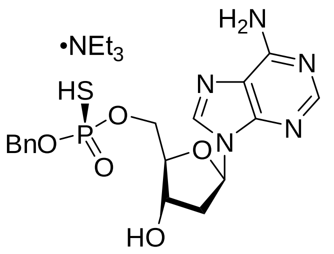

1a was purchased from WuXi AppTec. White powder. **^{1}H NMR** (400 MHz, Methanol-*d*4) δ 8.61 (s, 1H), 8.19 (s, 1H), 7.44 – 7.13 (m, 5H), 6.50 (dd, *J* = 7.8, 6.1 Hz, 1H), 5.02 – 4.89 (m, 2H), 4.64 (dt, *J* = 5.2, 2.4 Hz, 1H), 4.16 – 4.04 (m, 3H), 3.17 (q, *J* = 7.2 Hz, 6H), 2.78 – 2.71 (m, 1H), 2.46 – 2.40 (m, 1H), 1.28 (t, *J* = 7.3 Hz, 9H). **^{13}C NMR** (101 MHz, Methanol-d4) δ 157.2, 153.7, 150.4, 141.3, 139.5 (d, *J* = 8.7 Hz), 129.2, 128.6, 128.6, 120.1, 88.0 (d, *J* = 8.9 Hz), 85.5, 73.3, 68.8 (d, *J* = 5.3 Hz), 66.5 (d, *J* = 6.1 Hz), 47.7, 41.5, 9.2. **^{31}P NMR** (162 MHz, Methanol-*d*4) δ 57.8. **IR** (neat, ν/cm^{-1}) = 3326, 3182, 1645, 1597, 1576, 1474, 1416, 1332, 1297, 1246, 1213, 934, 800, 699; **HRMS** (ESI, m/z): [M + H]$^+$ calcd for $C_{17}H_{21}N_5O_5PS$ = 438.0996; found = 438.0993.

***O*-(((2*R*,2*R*)-5-(6-amino-9*H*-purin-9-yl)-3-hydroxytetrahydrofuran-2-yl)methyl) *O*-benzyl (*R*)-phosphorothioate triethylammonium (1b)**

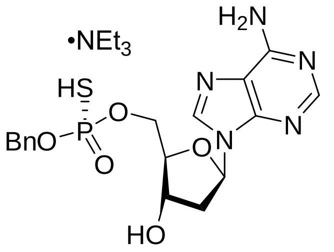

1b was purchased from WuXi AppTec. White powder. **^{1}H NMR** (400 MHz, Methanol-*d*4) δ 8.59 (s, 1H), 8.19 (s, 1H), 7.40 – 7.17 (m, 5H), 6.49 (dd, *J* = 7.8, 6.1 Hz, 1H), 5.03 – 4.91 (m, 2H), 4.61 (dt, *J* = 5.4, 2.5 Hz, 1H), 4.19 – 4.02 (m, 3H), 3.15 (q, *J* = 7.3 Hz, 6H), 2.76 – 2.69 (m, 1H), 2.45 – 2.40 (m, 1H), 1.26 (t, *J* = 7.3 Hz, 9H). **^{13}C NMR** (101 MHz, Methanol-*d*4) δ 157.2, 153.7, 150.4, 141.2, 139.5 (d, *J* = 8.8 Hz), 129.2, 128.6, 128.6, 120.1, 87.9 (d, *J* = 9.0 Hz), 85.5, 73.3, 68.7 (d, *J* = 5.2 Hz), 66.8 (d, *J* = 5.9 Hz), 47.7, 41.6, 9.2. **^{31}P NMR** (162 MHz, Methanol-*d*4) δ 57.9. **IR** (neat, ν/cm^{-1}) = 3323, 3176, 3006, 2990, 1598, 1576, 1474, 1456, 1332, 1297, 1276, 1268, 1261, 1214, 1098, 933, 800, 764, 749, 700; **HRMS** (ESI, m/z): [M + H]$^+$ calcd for $C_{17}H_{21}N_5O_5PS$ = 438.0996; found = 438.0991.

((2*R*,2*R*)-5-(6-amino-9*H*-purin-9-yl)-3-hydroxytetrahydrofuran-2-yl)methyl benzyl phosphate triethylammonium (1c)

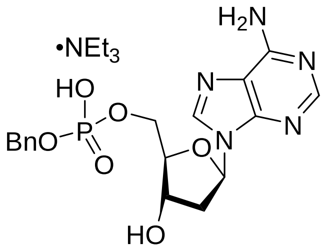

Purchased from WuXi AppTec. White powder **^{1}H NMR** (400 MHz, Methanol-*d*4) δ 8.46 (s, 1H), 8.18 (s, 1H), 7.37 – 7.15 (m, 5H), 6.47 (dd, *J* = 7.6, 6.0 Hz, 1H), 4.86 – 4.81 (m, 2H), 4.60 (dt, *J* = 6.0, 2.8 Hz, 1H), 4.13 – 4.10 (m, 1H), 4.03 (dd, *J* = 5.2, 4.0 Hz, 2H), 3.15 (q, *J* = 7.2 Hz, 6H), 2.81 – 2.70 (m, 1H), 2.44 (ddd, *J* = 13.6, 6.4, 3.2 Hz, 1H), 1.27 (t, *J* = 7.3 Hz, 9H). **^{13}C NMR** (101 MHz, Methanol-*d*4) δ 157.1,

153.5, 150.4, 141.0, 139.6 (d, J = 8.0 Hz), 129.3, 128.6, 128.4, 120.2, 87.8 (d, J = 8.7 Hz), 85.4, 72.9, 68.4 (d, J = 5.1 Hz), 66.5 (d, J = 5.5 Hz), 47.7, 41.1, 9.2. **^{31}P NMR** (162 MHz, Methanol-d_4) δ 0.2. **IR** (neat, ν/cm^{-1}) = 3325, 3185, 1653, 1599, 1573, 1497, 1455, 1417, 1332, 1297, 1275, 1268, 1260, 1211, 1162, 1079, 1052, 1021, 893, 800, 764, 749, 698, 668, 599, 511; **HRMS** (ESI, m/z): [M + H]$^+$ calcd for $C_{17}H_{21}N_5O_6P$ = 422.1224; found = 422.1203.

Synthesis of **1d**

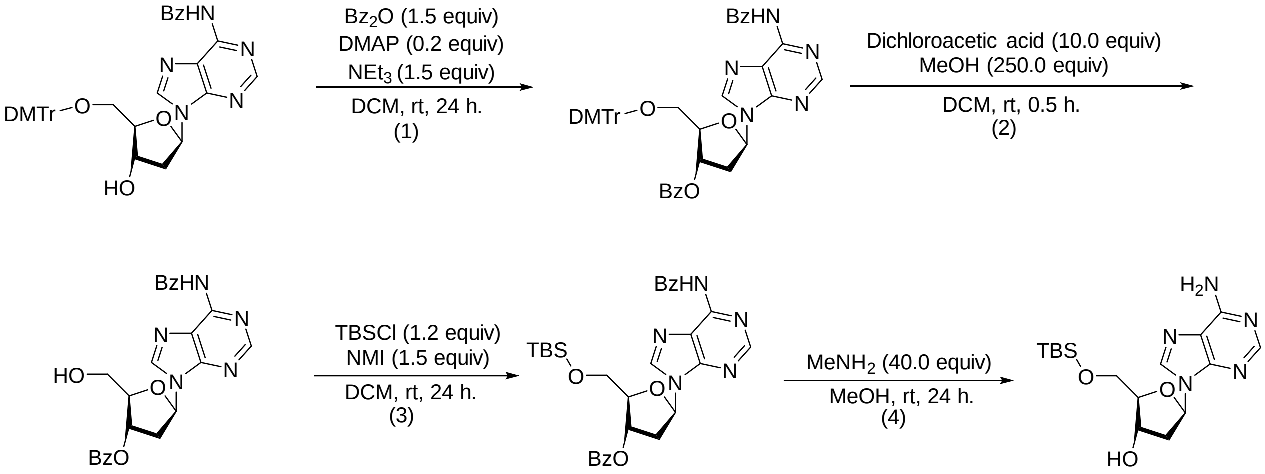

1. *N*-(9-((2*R*,5*R*)-5-((Bis(4-methoxyphenyl)(phenyl)methoxy)methyl)-4-hydroxytetrahydrofuran-2-yl)-9*H*-purin-6-yl)benzamide (1315.5 mg, 2.0 mmol, 1.0 eq.), benzoic anhydride (678.6 mg, 3.0 mmol, 1.5 eq.), NEt_3 (418.0 mg, 3.0 mmol, 1.5 eq.), and DMAP (73.2 mg, 0.2 mmol, 0.2 eq.) were added to a 100 mL round-bottom flask, containing a magnetic stir bar, and dissolved in CH_2Cl_2 (20.0 mL, 0.5 M). The reaction mixture was stirred at rt for 24 h. The mixture was diluted with EtOAc (100.0 mL) and washed with aqueous HCl (20.0 mL, 1.0 M) three times. The organic layer was dried over Na_2SO_4 and concentrated to dryness under reduced pressure. The residue was used directly in the next step without further purification.
2. The residue from the last step was dissolved in CH_2Cl_2 (20.0 mL, 0.5 M). Dichloroacetic acid (1653.1 µL, 20.0 mmol, 10.0 eq.) was added and the mixture was allowed to stir at rt for 0.5 h. After completion of the reaction, MeOH (20.0 mL, 500 mmol, 250.0 eq.) was added to quench the reaction. After 5 min, $NaHCO_3$ (200.0 mL, saturated aq) was added slowly, the organic layer was dried over Na_2SO_4 and concentrated to dryness under reduced pressure. The residue was used directly in the next step without further purification.
3. The residue from the last step was dissolved in CH_2Cl_2 (20.0 mL, 0.5 M), TBSCl (361.7 mg, 2.4 mmol, 1.2 eq.) and NMI (204.2 mg, 3.0 mmol, 1.5 eq.) were added, the mixture was allowed to stir at rt for 24 h. The mixture was washed with aqueous HCl (20.0 mL, 1.0 M) three times. The organic layer was dried over Na_2SO_4 and concentrated to dryness under reduced pressure. The residue was used directly in the next step without further purification.

4. The residue from the last step was dissolved in a $MeNH_2$ solution (9957.4 μL, 33% in MeOH, 40.0 eq.) and the mixture was allowed to stir at rt for 24 h. The resulting mixture was concentrated to dryness under reduced pressure. The residue was purified by normal phase column chromatography (Biotage®, SNAP Ultra 25 g; gradient 0%-20% MeOH/EtOAc over 12 CV), and then recrystallized from EtOAc/hexane to afford the desired product **1d** in 74% yield (538.2 mg).

(*2R,2R*)-5-(6-amino-9*H*-purin-9-yl)-2-(((*tert*-butyldimethylsilyl)oxy)methyl) tetrahydrofuran-3-ol (1d)

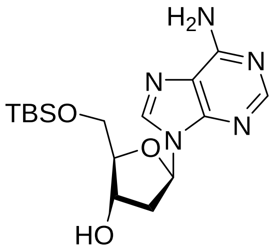

^{1}H NMR (400 MHz, Chloroform-*d*) δ 8.32 (s, 1H), 8.17 (s, 1H), 6.51 (t, *J* = 6.4 Hz, 1H), 5.98 (s, 2H), 4.68 (dt, *J* = 6.4, 3.6 Hz, 1H), 4.11 (tq, *J* = 3.6, 2.0 Hz, 1H), 3.92 – 3.81 (m, 2H), 2.73 – 2.63 (m, 1H), 2.56 (ddd, *J* = 13.2, 6.0, 4.0 Hz, 1H), 0.89 (s, 9H), 0.09 (s, 6H). **^{13}C NMR** (101 MHz, Chloroform-*d*) δ 155.5, 153.0, 149.5, 139.0, 119.9, 87.3, 84.4, 72.2, 63.6, 41.5, 26.1, 18.5, –5.2, –5.4. **IR** (neat, ν/cm^{-1}) = 3325, 3172, 2929, 2858, 1653, 1647, 1598, 1577, 1559, 1507, 1473, 1362, 1329, 1299, 1276, 1268, 1261, 1208, 1126, 1066, 1001, 935, 834, 799, 765, 759, 749; **HRMS** (ESI, m/z): $[M + H]^+$ calcd for $C_{16}H_{28}N_5O_3Si$ = 366.1956; found = 366.1948.

((3*aR*,6*aR*)-6-(6-amino-9*H*-purin-9-yl)-2,2-dimethyltetrahydrofuro[3,4-*d*][1,3]dioxol-4-yl)methanol (1e)

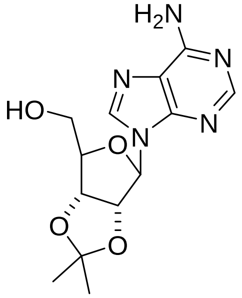

Purchased from Sigma-Aldrich. **^{1}H NMR** (400 MHz, Methanol-*d*4) δ 8.33 (s, 1H), 8.19 (s, 1H), 6.15 (d, *J* = 3.5 Hz, 1H), 5.28 (dd, *J* = 6.0, 3.6 Hz, 1H), 5.04 (dd, *J* = 6.0, 2.4 Hz, 1H), 4.38 (td, *J* = 3.7, 2.3 Hz, 1H), 3.84 – 3.66 (m, 2H), 1.62 (s, 3H), 1.38 (s, 3H). **^{13}C NMR** (101 MHz, Methanol-*d*4) δ 157.5, 153.8, 150.0, 141.7, 120.7, 115.3, 92.9, 88.1, 85.3, 83.0, 63.6, 27.6, 25.5. **IR** (neat, ν/cm^{-1}) = 3324, 3164, 1601, 1374, 1334, 1275, 1209, 1156, 1110, 1079, 851, 764, 749; **HRMS** (ESI, m/z): $[M + H]^+$ calcd for $C_{13}H_{18}N_5O_4$ = 308.1354; found = 308.1352.

Synthesis of **1f**

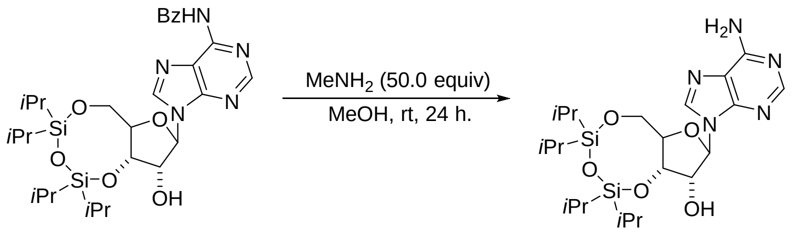

N-(9-((9*R*,9*aS*)-9-Hydroxy-2,2,4,4-tetraisopropyltetrahydro-6*H*-furo[3,2-f][1,3,5,2,4] trioxadisilocin-8-yl)-9*H*-purin-6-yl)benzamide was dissolved in a $MeNH_2$ solution (6.0 mL, 33% in MeOH, 50.0 eq.) and the mixture was allowed to stir at rt for 24 h. The resulting mixture was concentrated to dryness under reduced pressure. The residue was purified by normal phase column chromatography (Biotage®, SNAP Ultra 25 g; gradient 0%-100% EtOAc/hexane over 12 CV; gradient 0%-20% MeOH/EtOAc over 8 CV), and then recrystallized from EtOAc/hexane to afford the desired product **1f** in 74% yield (375.6 mg).

(9*R*,9*aS*)-8-(6-amino-9*H*-purin-9-yl)-2,2,4,4-tetraisopropyltetrahydro-6*H*-furo[3,2-f][1,3,5,2,4]trioxadisilocin-9-ol (1f)

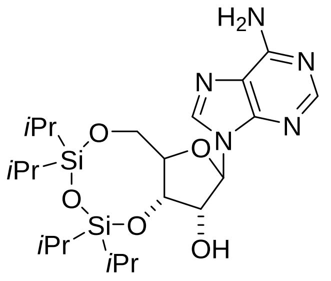

^{1}H NMR (400 MHz, DMS*O*-*d*$_6$) δ 8.21 (s, 1H), 8.07 (s, 1H), 7.33 (s, 2H), 5.87 (s, 1H), 5.63 (d, *J* = 4.8 Hz, 1H), 4.79 (dd, *J* = 8.4, 5.2 Hz, 1H), 4.51 (t, *J* = 5.2 Hz, 1H), 4.09 – 3.89 (m, 3H), 1.24 – 0.76 (m, 28H). **^{13}C NMR** (101 MHz, DMS*O*-*d*$_6$) δ 156.1, 152.5, 148.6, 139.2, 119.3, 89.4, 80.8, 73.6, 69.8, 60.7, 17.4, 17.2, 17.2, 17.0, 16.9, 16.8, 12.7, 12.5, 12.3, 12.1. **IR** (neat, ν/cm^{-1}) = 3328, 3162, 2945, 2868, 1653, 1600, 1465, 1420, 1331, 1210, 1121, 1037, 991, 906, 884, 859, 823, 799, 765; **HRMS** (ESI, m/z): $[M + H]^+$ calcd for $C_{22}H_{40}N_5O_5Si_2$ = 510.2563; found = 510.2550.

Synthesis of **1g**

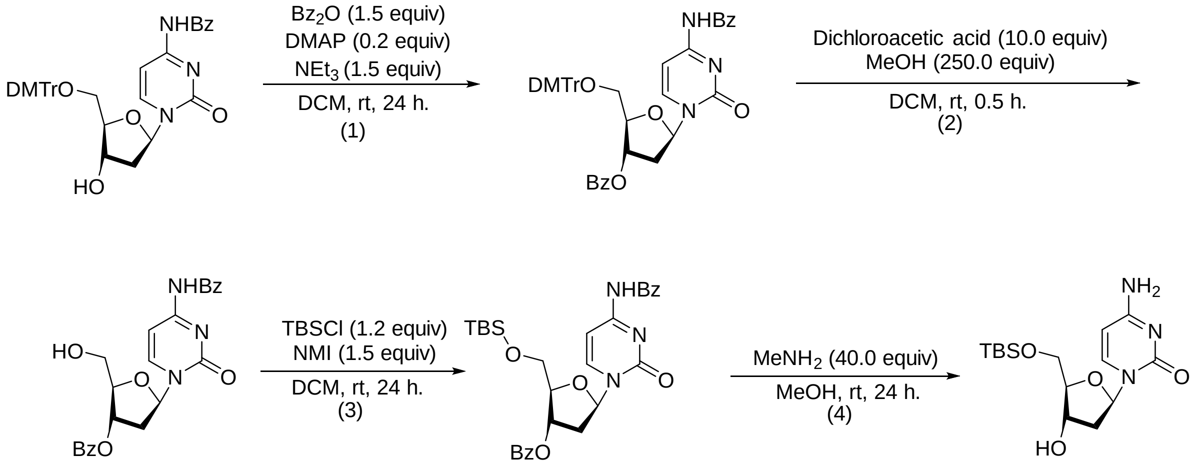

1. *N*-(1-((2*R*,5*R*)-5-((Bis(4-methoxyphenyl)(phenyl)methoxy)methyl)-4-hydroxytetrahydrofuran-2-yl)-2-oxo-1,2-dihydropyrimidin-4-yl)benzamide (1143.2 mg, 2.0 mmol, 1.0 eq.), benzoic anhydride (678.6 mg, 3.0 mmol, 1.5 eq.), NEt_3 (418.0 mg, 3.0 mmol, 1.5 eq.), and DMAP (73.2 mg, 0.2 mmol, 0.2 eq.) were added to a 100 mL round-bottom flask, containing a magnetic stir bar, and dissolved in CH_2Cl_2 (20.0 mL, 0.5 M). The reaction mixture was stirred at rt for 24 h. The mixture was diluted with EtOAc (100.0 mL) and washed with aqueous HCl (20.0 mL, 1.0 M) three

times. The organic layer was dried over Na_2SO_4 and concentrated to dryness under reduced pressure. The residue was used directly in the next step without further purification.

2. The residue from the last step was dissolved in CH_2Cl_2 (20.0 mL, 0.5 M). Dichloroacetic acid (1653.1 μL, 20.0 mmol, 10.0 eq.) was added, the mixture was allowed to stir at rt for 0.5 h. After completion of the reaction, MeOH (20.0 mL, 500 mmol, 250.0 eq.) was added to quench the reaction. After 5 min, $NaHCO_3$ (200.0 mL, saturated aq) was added slowly. The organic layer was dried over Na_2SO_4 and concentrated to dryness under reduced pressure. The residue was used directly in the next step without further purification.

3. The residue from the last step was dissolved in CH_2Cl_2 (20.0 mL, 0.5 M), TBSCl (361.7 mg, 2.4 mmol, 1.2 eq.) and NMI (204.2 mg, 3.0 mmol, 1.5 eq.) were added. The mixture was allowed to stir at rt for 24 h and then washed with aqueous HCl (20.0 mL, 1.0 M) three times. The organic layer was dried over Na_2SO_4 and concentrated to dryness under reduced pressure. The residue was used directly in the next step without further purification.

4. The residue from the last step was dissolved in a $MeNH_2$ solution (9957.4 μL, 33% in MeOH, 40.0 eq.), the mixture was allowed to stir at rt for 24 h. The resulting mixture was concentrated to dryness under reduced pressure. The residue was purified by normal phase column chromatography (Biotage®, SNAP Ultra 25 g; gradient 0%-20% MeOH/EtOAc over 12 CV), and then recrystallized from EtOAc/hexane to afford the desired product **1g** in 71% yield (487.1 mg).

4-amino-1-((*2R,2R*)-5-(((*tert*-butyldimethylsilyl)oxy)methyl)-4-hydroxytetrahydrofuran-2-yl)pyrimidin-2(1*H*)-one (1g)

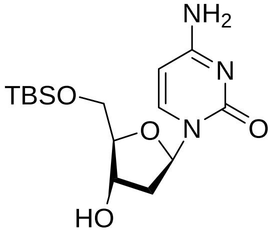

^{1}H NMR (400 MHz, DMS*O-d*$_6$) δ 7.76 (d, J = 7.6 Hz, 1H), 7.14 (s, 2H), 6.15 (t, J = 6.4 Hz, 1H), 5.68 (d, J = 7.6 Hz, 1H), 5.25 (d, J = 4.2 Hz, 1H), 4.17 (dq, J = 7.2, 3.6 Hz, 1H), 3.83 – 3.67 (m, 3H), 2.15 (ddd, J = 13.2, 6.0, 3.6 Hz, 1H), 1.90 (dt, J = 13.2, 6.4 Hz, 1H), 0.88 (s, 9H), 0.07 (s, 6H). **^{13}C NMR** (101 MHz, DMS*O-d*$_6$) δ 165.5, 155.0, 140.5, 93.7, 86.6, 84.8, 70.1, 63.0, 40.8, 25.8, 18.0, –5.5, –5.6. **IR** (neat, ν/cm^{-1}) = 2954, 2930, 2857, 1664, 1621, 1600, 1483, 1399, 1253, 1196, 1099, 1001, 935, 784, 712, 595; **HRMS** (ESI, m/z): $[M + H]^+$ calcd for $C_{15}H_{28}N_3O_4Si$ = 342.1844; found = 342.1837.

Synthesis of **1h**

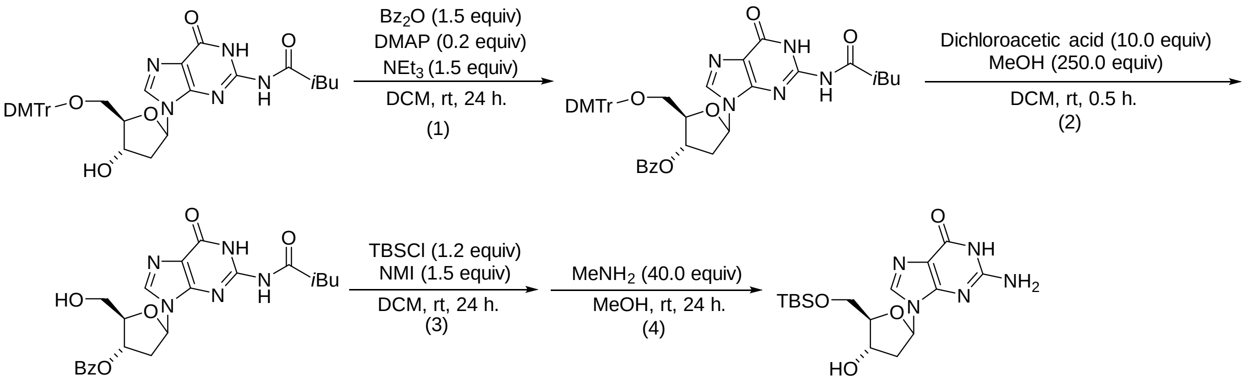

1. *N*-(9-((4*S*,5*R*)-5-((Bis(4-methoxyphenyl)(phenyl)methoxy)methyl)-4-hydroxytetrahydrofuran-2-yl)-6-oxo-6,9-dihydro-1*H*-purin-2-yl)-3-methylbutanamide (1307.5 mg, 2.0 mmol, 1.0 eq.), Benzoic anhydride (678.6 mg, 3.0 mmol, 1.5 eq.), NEt_3 (418.0 mg, 3.0 mmol, 1.5 eq.), and DMAP (73.2 mg, 0.2 mmol, 0.2 eq.) were added to a 100 mL round-bottom flask, containing a magnetic stir bar, and dissolved in CH_2Cl_2 (20.0 mL, 0.5 M). The reaction mixture was stirred at rt for 24 h. The mixture was diluted with EtOAc (100.0 mL) and washed with aqueous HCl (20.0 mL, 1.0 M) three times. The organic layer was dried over Na_2SO_4 and concentrated to dryness under reduced pressure. The residue was used directly in the next step without further purification.
2. The residue from the last step was dissolved in CH_2Cl_2 (20.0 mL, 0.5 M). Dichloroacetic acid (1653.1 μL, 20.0 mmol, 10.0 eq.) was added, and the mixture was allowed to stir at rt for 0.5 h. After completion of the reaction, MeOH (20.0 mL, 500 mmol, 250.0 eq.) was added to quench the reaction. After 5 min, $NaHCO_3$ (200.0 mL, saturated. aq) was added slowly. The organic layer was dried over Na_2SO_4 and concentrated to dryness under reduced pressure. The residue was used directly in the next step without further purification.
3. The residue from the last step was dissolved in CH_2Cl_2 (20.0 mL, 0.5 M), TBSCl (361.7 mg, 2.4 mmol, 1.2 eq.) and NMI (204.2 mg, 3.0 mmol, 1.5 eq.) was added, the mixture was allowed to stir at rt for 24 h. The mixture was washed with aqueous HCl (20.0 mL, 1.0 M) three times. The organic layer was dried over Na_2SO_4 and concentrated to dryness under reduced pressure. The residue was used directly in the next step without further purification.
4. The residue from the last step was dissolved in a $MeNH_2$ solution (9957.4 μL, 33% in MeOH, 40.0 eq.) and the mixture was allowed to stir at rt for 24 h. The resulting mixture was concentrated to dryness under reduced pressure. The residue was purified by normal phase column chromatography (Biotage®, SNAP Ultra 25 g; gradient 0%-20% MeOH/EtOAc over 12 CV), and then recrystallized from EtOAc/hexane to afford the desired product **1g** in 52% yield (395.4 mg).

2-amino-9-((*2R,2R*)-5-(((*tert*-butyldimethylsilyl)oxy)methyl)-4-hydroxytetrahydrofuran-2-yl)-1,9-dihydro-6*H*-purin-6-one (1h)

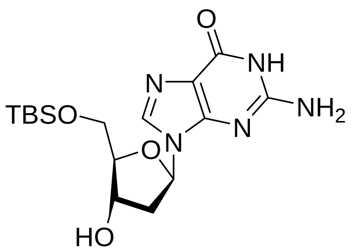

^{1}H NMR (400 MHz, DMS*O*-*d*$_6$) δ 10.62 (s, 1H), 7.84 (s, 1H), 6.47 (s, 2H), 6.11 (t, *J* = 6.8 Hz, 1H), 5.32 (d, *J* = 4.2 Hz, 1H), 4.32 (dq, *J* = 6.8, 3.6 Hz, 1H), 3.82 (q, *J* = 4.2 Hz, 1H), 3.70 (qd, *J* = 11.2, 4.4 Hz, 2H), 2.49 – 2.42 (m, 1H), 2.23 (ddd, *J* = 13.2, 6.0, 3.6 Hz, 1H), 0.86 (s, 9H), 0.03 (s, 6H). **^{13}C NMR** (101 MHz, DMS*O*-*d*$_6$) δ 156.8, 153.7, 150.9, 134.9, 116.6, 87.0, 82.4, 70.4, 63.3, 25.9, 18.1, –5.4, –5.4. **IR** (neat, ν/cm^{-1}) = 3361, 2929, 2363, 1684, 1653, 1559, 1540, 1506, 1259, 1164, 1077, 1003, 936, 834, 778; **HRMS** (ESI, m/z): $[M + H]^+$ calcd for $C_{16}H_{28}N_5O_4Si$ = 382.1906; found = 382.1897.

Synthesis of **2e**

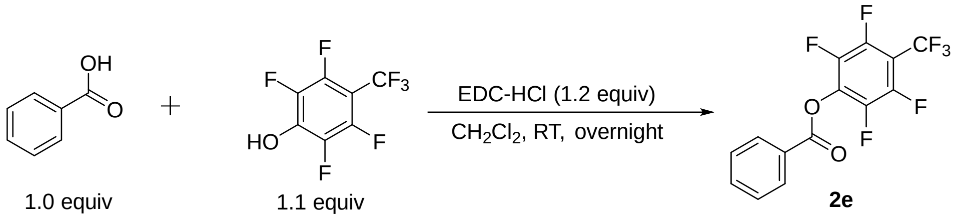

Benzoate (2012.2 mg, 10.0 mmol, 1.0 eq.), EDC-HCl (2300.4 mg, 12.0 mmol, 1.2 eq.), and Perfluoro-*p*-cresol (1837.7 mg, 12.0 mmol, 1.2 eq.) were added to a 100 mL round-bottom flask, containing a magnetic stir bar, and dissolved in CH_2Cl_2 (20.0 mL, 0.5 M). The reaction mixture was stirred at rt for 24 h. The mixture was diluted with EtOAc (100.0 mL), and washed with aqueous HCl (20.0 mL, 1.0 M) three times. The organic layer was dried over Na_2SO_4 and concentrated to dryness under reduced pressure. The residue was purified by normal phase column chromatography (Biotage®, SNAP Ultra 50 g; gradient 0%-100% EtOAc/hexane over 10 CV), and then recrystallized from EtOAc/hexane to afford the desired product **2e** in 68% yield (1154.8 mg).

2,3,5,6-tetrafluoro-4-(trifluoromethyl)phenyl benzoate (2e)

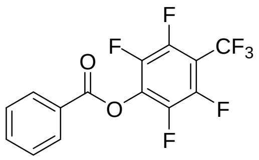

^{1}**H NMR** (400 MHz, Chloroform-*d*) δ 8.21 (d, *J* = 7.2 Hz, 2H), 7.73 (t, *J* = 7.2 Hz, 1H), 7.57 (t, *J* = 7.2 Hz, 2H). ^{13}**C NMR** (101 MHz, Chloroform-*d*) δ 162.2, 151.5 – 150.1 (m), 146.5 – 146.0 (m), 143.2 – 142.6 (m), 140.5 – 140.2 (m), 135.2, 131.1, 129.2, 126.8. ^{19}**F NMR** (376 MHz, Chloroform-*d*) δ -56.1 (t, *J* = 22.1 Hz), -138.9 – -143.0 (m), -147.8 – -152.6 (m). **IR** (neat, ν/cm^{-1}) = 1773, 1766, 1453, 1347, 1176, 1136, 993, 877, 719, 703.

Synthesis of **2f**

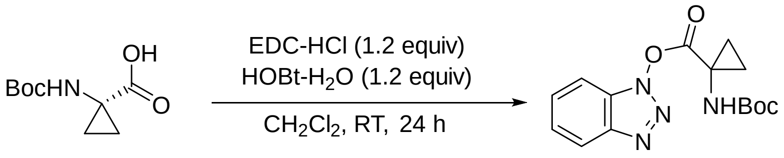

2f

Boc-Acpc-OH (2012.2 mg, 10.0 mmol, 1.0 eq.), EDC-HCl (2300.4 mg, 12.0 mmol, 1.2 eq.), and HOBt-H_2O (1837.7 mg, 12.0 mmol, 1.2 eq.) were added to a 100 mL round-bottom flask, containing a magnetic stir bar, and dissolved in CH_2Cl_2 (20.0 mL, 0.5 M). The reaction mixture was stirred at rt for 24 h. The mixture was diluted with EtOAc (100.0 mL), and washed with aqueous HCl (20.0 mL, 1.0 M) three times. The organic layer was dried over Na_2SO_4 and concentrated to dryness under reduced pressure. The residue was purified by normal phase column chromatography (Biotage®, SNAP Ultra 50 g; gradient 0%-100% EtOAc/hexane over 10 CV), and then recrystallized from EtOAc/hexane to afford the desired product **2f** in 87% yield (2779.0 mg).

1*H*-benzo[d][1,2,3]triazol-1-yl 1-((*tert*-butoxycarbonyl)amino)cyclopropane-1-carboxylate (2f)

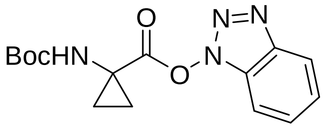

^{1}H NMR (400 MHz, Chloroform-*d*) δ 8.05 (d, *J* = 8.4 Hz, 1H), 7.61 (d, *J* = 8.0 Hz, 1H), 7.55 (t, *J* = 7.5 Hz, 1H), 7.42 (t, *J* = 8.0 Hz, 1H), 5.43 (s, 1H), 1.87 (q, *J* = 5.0 Hz, 2H), 1.57 – 1.36 (m, 11H). **^{13}C NMR** (101 MHz, Chloroform-*d*) δ 169.9, 143.6, 129.0, 128.6, 125.1, 120.4, 108.9, 81.2, 48.4, 28.4, 20.4. **IR** (neat, ν/cm^{-1}) = 1803, 1711, 1497, 1368, 1282, 1250, 1163, 1062, 1022, 782, 767, 743; **HRMS** (ESI, m/z): [M + H]$^{+}$ calcd for $C_{15}H_{19}N_{4}O_{4}$ = 319.1401; found = 319.1393.

Synthesis of **2g**

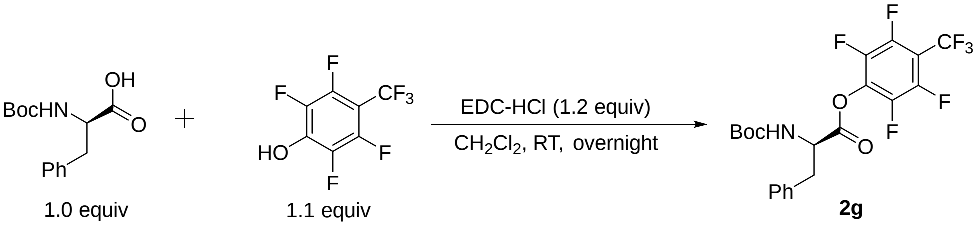

Boc-Phe-OH (2012.2 mg, 10.0 mmol, 1.0 eq.), EDC-HCl (2300.4 mg, 12.0 mmol, 1.2 eq.), and Perfluoro-*p*-cresol (1837.7 mg, 12.0 mmol, 1.2 eq.) were added to a 100 mL round-bottom flask, containing a magnetic stir bar, and dissolved in CH_2Cl_2 (20.0 mL, 0.5 M). The reaction mixture was stirred at rt for 24 h. The mixture was diluted with EtOAc (100.0 mL), and washed with aqueous HCl (20.0 mL, 1.0 M) three times. The organic layer was dried over Na_2SO_4 and concentrated to dryness under reduced pressure. The residue was purified by normal phase column chromatography (Biotage®, SNAP Ultra 50 g; gradient 0%-100% EtOAc/hexane over 10 CV), and then recrystallized from EtOAc/hexane to afford the desired product **2g** in 60% yield (2873.4 mg).

2,3,5,6-tetrafluoro-4-(trifluoromethyl)phenyl (*tert*-butoxycarbonyl)-*L*-phenylalaninate (2g)

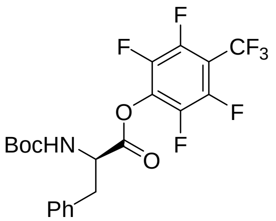

^{1}H NMR (400 MHz, Chloroform-*d*) δ 7.40 – 7.28 (m, 3H), 7.26 – 7.21 (m, 2H), 4.96 – 4.91 (m, 1H), 3.35 – 3.15 (m, 2H), 1.43 (s, 9H). **^{13}C NMR** (101 MHz, Chloroform-*d*) δ 168.0, 155.1, 149.0 – 147.8 (m), 146.5 – 145.4 (m), 143.9 – 142.8 (m), 142.7 – 142.1 (m), 140.2 – 139.3 (m), 134.8, 130.2 (q, *J* = 137.3 Hz), 129.4, 129.1, 127.7, 80.9, 54.6, 37.9, 28.3. **^{19}F NMR** (376 MHz, Chloroform-*d*) δ -56.12 (t, *J* = 22.0 Hz), -140.02 (tt, *J* = 20.2, 10.8 Hz), -149.33 (dq, *J* = 15.4, 8.9, 7.2 Hz). **IR** (neat, ν/cm^{-1}) = 1785, 1659, 1507, 1428, 1369, 1342, 1227, 1149, 1082, 1047, 999, 936, 876, 717;

Synthesis of **2h**

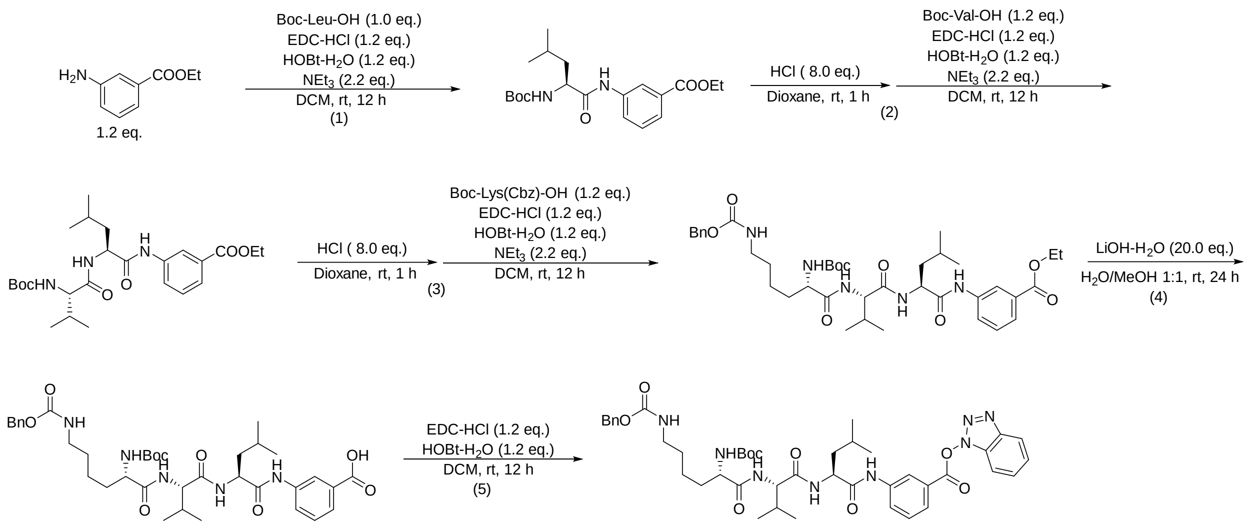

1. Ethyl 3-aminobenzoate (726.0 mg, 4.8 mmol, 1.2 eq.), Boc-Leu-OH (925.6 mg, 4.0 mmol, 1.0 eq.), EDC•HCl (917.6 mg, 4.8 mmol, 1.2 eq.), and HOBt•H_2O (735.0 mg, 4.8 mmol, 1.2 eq.) were dissolved in CH_2Cl_2 (20.0 mL, 0.2 M), followed by addition of NEt_3 (1532.8 μL, 8.8 mmol, 2.2 eq.). The reaction mixture was stirred at rt for 12 h, and the volatiles were removed under reduced pressure. The residue was diluted with EtOAc, washed with aqueous citric acid (50.0 mL, 10% aq.), $NaHCO_3$ (50.0 mL, saturated aq) and brine. The organic layer was dried over Na_2SO_4, filtered, and concentrated to dryness under reduced pressure. The residue was then used directly in the next step without further purification.

2. To the peptide residue, HCl (4 N in dioxane,8.0 mL, 32.0 mmol, 8.0 eq.) was added. The reaction was stirred at rt for 1 h followed by concentration to dryness under reduced pressure. The residue was dissolved in CH_2Cl_2 (20.0 mL, 0.2 M) followed by the addition of Boc-Leu-OH (1042.8 mg, 4.8 mmol, 1.2 eq.), EDC•HCl (917.6 mg, 4.8 mmol, 1.2 eq.), HOBt•H_2O (735.0 mg, 4.8 mmol, 1.2 eq.) and NEt_3 (1532.8 μL, 8.8 mmol, 2.2 eq.). The reaction mixture was stirred at rt for 12 h, and the volatiles were removed under reduced pressure. The residue was diluted with EtOAc, washed with citric acid (0.5 M *aq* solution), *sat* $NaHCO_{3\,(aq)}$, and brine. The organic layer was dried over Na_2SO_4, filtered, and concentrated to dryness under reduced pressure. The residue was purified by reversed phase column chromatography (Biotage®, SNAP Ultra C18 25 g; gradient 0%-50% MeCN/H_2O over 12 CV). The solvent was removed under reduced pressure, and the residue was used directly in the next step.

3. To the peptide residue, HCl (4 N in dioxane, 8.0 mL, 32.0 mmol, 8.0 eq.) was added. The reaction was stirred at rt for 1 h followed by concentration to dryness under reduced pressure The residue was dissolved in CH_2Cl_2 (20.0 mL, 0.2 M) followed by the addition of Boc-Lys(Cbz)-OH (1737.6mg, 4.8 mmol, 1.2 eq.), EDC•HCl (917.6 mg, 4.8 mmol, 1.2 eq.), HOBt•H_2O (735.0 mg, 4.8 mmol, 1.2 eq.) and NEt_3 (1532.8 μL, 8.8 mmol, 2.2 eq.). The reaction mixture was stirred at rt for 12 h, and the volatiles were removed under reduced pressure. The residue was diluted with EtOAc, washed with citric acid (0.5 M *aq* solution), *sat* $NaHCO_{3\,(aq)}$, and brine. The organic layer

was dried over Na_2SO_4, filtered, and concentrated under reduced pressure. The residue was purified by reversed phase column chromatography (Biotage®, SNAP Ultra C18 25 g; gradient 0%-50% MeCN/H_2O over 12 CV). The solvent was removed under reduced pressure, and the residue was used directly in the next step.

4. The peptide residue (1479.8 mg, 2.0 mmol, 2.0 eq.) was dissolved in water (20.0 mL) and methanol (20 mL) followed by the addition of LiOH-H_2O (3356.8 mg, 80.0 mmol, 20.0 eq.). The reaction mixture was stirred at rt for 24 h, and the volatiles were removed under reduced pressure. The reaction mixture was acidified with aqueous HCl (1 M, 200 mL), extracted with DCM (50 mL X 3). The organic layer was dried over Na_2SO_4, filtered, and concentrated to dryness under reduced pressure. The residue was purified by reversed phase column chromatography (Biotage®, SNAP Ultra C18 25 g; gradient 0%-40% MeOH/H_2O over 2 CV, 40%-90% MeOH/H_2O over 15 CV, 90%-100% MeOH/H_2O over 2 CV) to afford the desired product (859.6 mg, 58% yield).

5. The product from step 4 (569.5 mg, 0.8 mmol, 1.0 eq.), EDC•HCl (184.0 mg, 0.96 mmol, 1.2 eq.), and HOBt•H_2O (147.0 mg, 0.96 mmol, 1.2 eq.) were dissolved in CH_2Cl_2 (20.0 mL, 0.2 M). The reaction mixture was stirred at rt for 12 h, and the volatiles were removed under reduced pressure. The residue was diluted with EtOAc, washed with citric acid (0.5 M *aq* solution), *sat* $NaHCO_{3\ (aq)}$, and brine. The organic layer was dried over Na_2SO_4, filtered, and concentrated *in vacuo*. The residue was purified by reversed phase column chromatography (Biotage®, SNAP Ultra C18 25 g; gradient 0%-50% MeCN/H_2O over 12 CV) to afford the desired product **2h** (483.5 mg, 73% yield).

1*H*-benzo[d][1,2,3]triazol-1-yl 3-((9*S*,12*S*,15*S*)-9-((tert-butoxycarbonyl)amino)-15-isobutyl-12-isopropyl-3,10,13-trioxo-1-phenyl-2-oxa-4,11,14-triazahexadecan-16-amido)benzoate (2h)

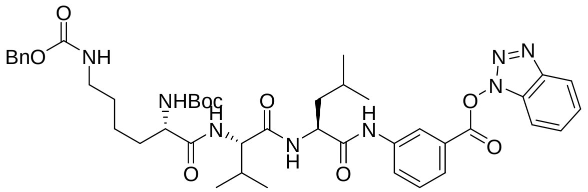

^{1}H NMR (400 MHz, Chloroform-*d*) δ 9.11 (s, 1H), 8.81 (s, 1H), 8.45 (d, *J* = 8.0 Hz, 1H), 8.06 (d, *J* = 8.4 Hz, 1H), 7.93 (d, *J* = 8.0 Hz, 1H), 7.61 – 7.28 (m, 8H), 6.68 (d, *J* = 5.2 Hz, 1H), 5.99 (s, 1H), 5.21 – 5.06 (m, 2H), 5.01 (t, *J* = 6.4 Hz, 1H), 4.71 (td, *J* = 9.6, 8.4, 3.4 Hz, 1H), 4.29 – 4.16 (m, 1H), 4.00 – 3.87 (m, 1H), 3.15 (s, 1H), 3.08 (s, 1H), 2.41 (ddd, *J* = 13.6, 10.4, 6.4 Hz, 1H), 2.02 – 1.67 (m, 5H), 1.44 (s, 11H), 1.08 – 0.85 (m, 10H). **^{13}C NMR** (101 MHz, Chloroform-*d*) δ 174.5, 171.6, 171.0, 162.9, 157.7, 157.5, 143.6, 139.9, 136.5, 129.8, 128.9, 128.7, 128.4, 128.1, 127.0, 125.9, 125.9, 122.3, 120.5, 108.7, 81.4, 66.9, 60.4, 57.2, 52.6, 39.9, 39.0, 29.8, 29.7, 28.9, 28.3, 25.0, 23.5, 22.1, 20.7, 19.6, 17.4, 14.3. **IR** (neat, ν/cm^{-1}) = 3283, 2962, 1793, 1640, 1542, 1437, 1368, 1266, 1169, 1088, 995, 736; **HRMS** (ESI, m/z): $[M + H]^+$ calcd for $C_{43}H_{57}N_8O_9$ = 829.4244; found = 829.4230.

3. General procedure and scope for chemoselective acylation

Figure S1. Substrate scope of nucleoside analogs[a]

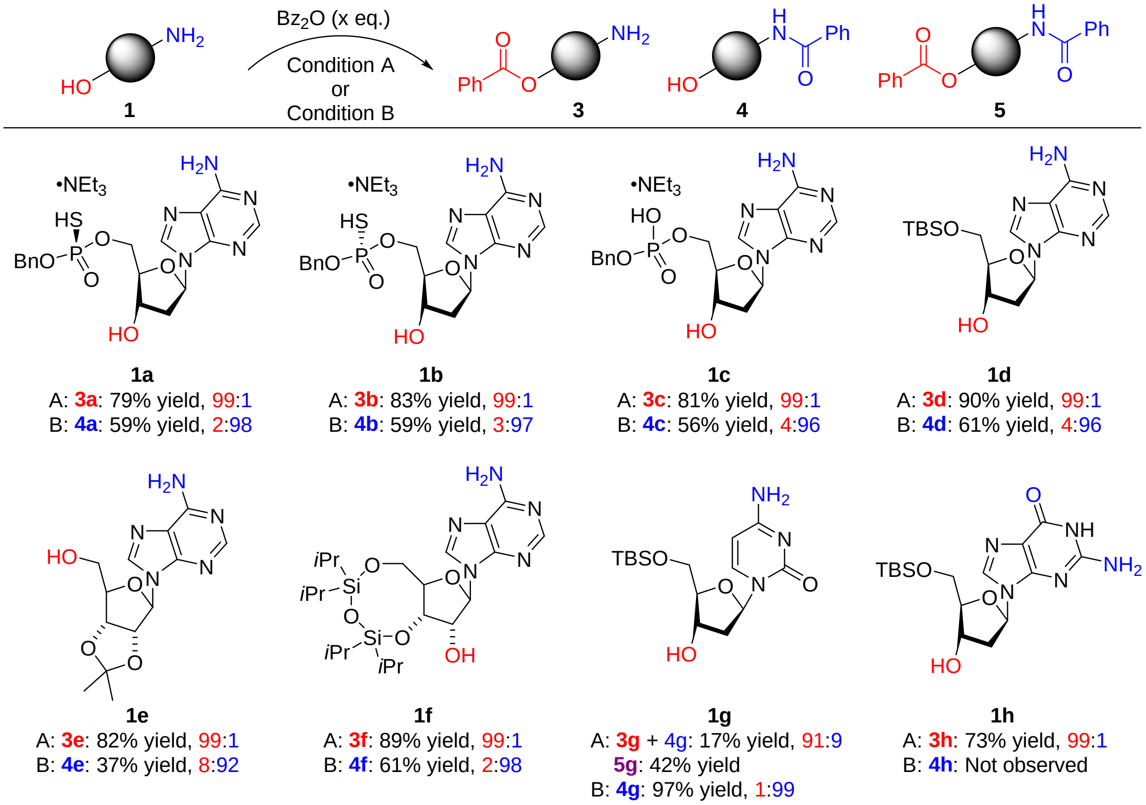

a. Condition A: **1** (0.1 mmol, 1.0 eq.), Bz_2O (0.12 mmol, 1.2 eq.), DMAP (0.01 mmol, 10 mmol%), NEt_3 (0.15 mmol, 1.5 eq.), $CHCl_3$ (0.5 mL, 0.2 M), rt, 16 h. Condition B: **1** (0.1 mmol, 1.0 eq.), Bz_2O (0.15 mmol, 1.5 eq.), $CHCl_3$ (0.5 mL, 0.2 M), 50 ˚C, 16 h.

Figure S2. Investigation of the electrophiles.[a]

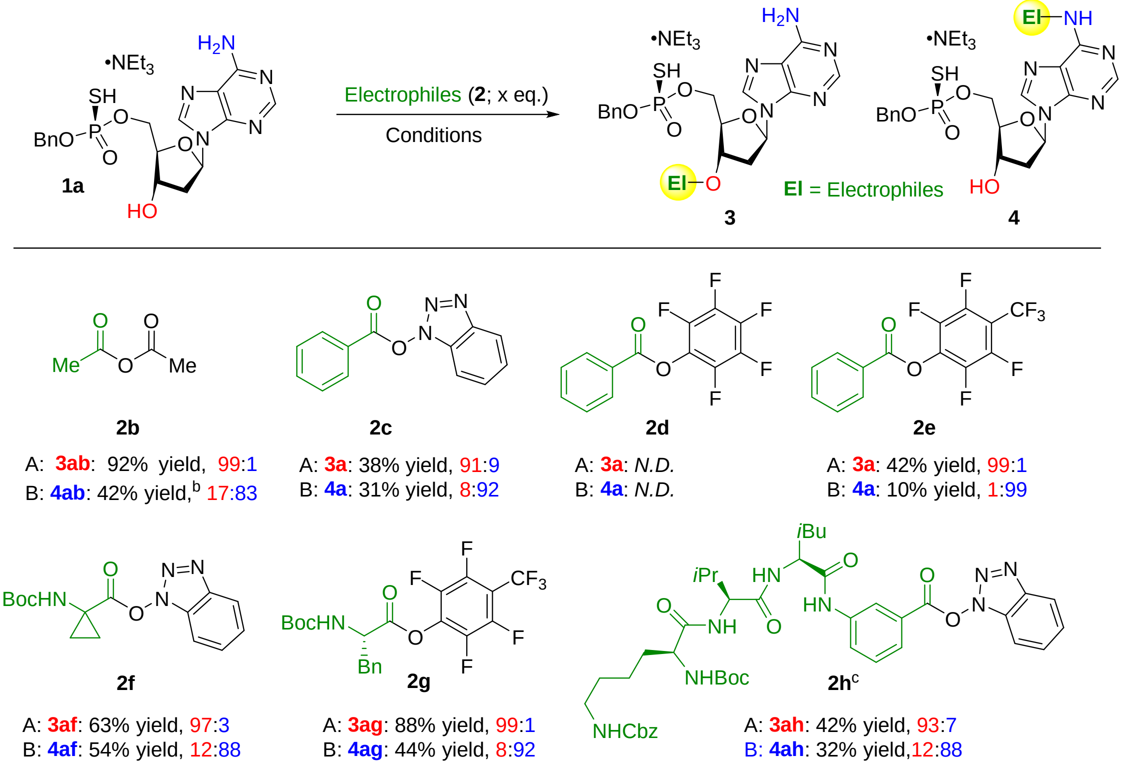

a. Condition A: **1a** (0.1 mmol), **2** (0.12 mmol, 1.2 eq.), DMAP (0.01 mmol, 10 mmol%), NEt_3 (0.15 mmol, 1.5 eq.), $CHCl_3$ (0.5 mL, 0.2 M), rt, 16 h. Condition B: **1a** (0.1 mmol), **2** (0.15 mmol, 1.5 eq.), $CHCl_3$ (0.5 mL, 0.2 M), 50 ˚C, 16 h. b. Yield was determined by ^{1}H NMR of **2b** and **4ab** mixture. c. Conducted at 0.05 mmol scale.

Condition A: Nucleoside **1** (0.10 mmol, 1.0 eq.) and acylation reagent **2** (0.12 mmol, 1.2 eq.) were dissolved in DCM (0.5 mL, 0.2 M) in a 2-mL vial equipped with a stir bar, followed by addition of DMAP (10.0 μL (1 M in $CHCl_3$), 0.01 mmol, 10 mol%) and NEt_3 (20.9 μL, 0.15 mmol, 1.5 eq.). The reaction was stirred at rt for 16 h. The reaction was quenched with MeOH (0.5 mL) and concentrated to dryness under reduced pressure. The chemoselectivity of the reaction was determined by ^{1}H NMR analysis of the crude reaction mixture. The mixture was purified by reversed-phase column chromatography to afford the desired *O*-acylated product **3**.

Condition B: Nucleoside **1** (0.10 mmol, 1.0 eq.) and acylation reagent **2** (0.12 mmol, 1.2 eq.) were dissolved in $CHCl_3$ (0.5 mL, 0.2 M) in a 2 mL vial equipped with a stir bar. The reaction was stirred at 50 ˚C for 16 h. The reaction was quenched with MeOH (0.5 mL) and concentrated to dryness under reduced pressure. The chemoselectivity of the reaction was determined by ^{1}H NMR analysis. The mixture was purified by reversed-phase column chromatography to afford the desired *N*-acylated product **4**.

O-(((2R,2R)-5-(6-amino-9H-purin-9-yl)-3-(benzoyloxy)tetrahydrofuran-2-yl)methyl) O-benzyl (S)-phosphorothioate triethylammonium (3a)

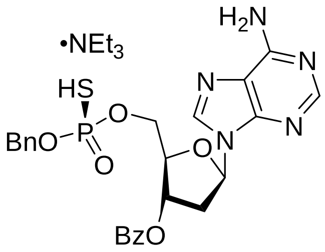

From **1a** under condition A. The chemoselectivity was determined by ^{1}H NMR of the crude sample (**3a**:**4a** > 99:1). The reaction mixture was purified by reversed-phase column chromatography (Biotage®, SNAP Ultra C18 25 g; gradient 0% MeCN (2% NEt_3)/H_2O over 2 CV, 0%-40% MeCN (2% NEt_3)/H_2O over 15 CV.) The acylation product **3a** was obtained as an amorphous solid (79% yield, average of two runs: 49.9 mg, 78%; 51.1 mg, 80%). **^{1}H NMR** (400 MHz, Methanol-*d*$_4$) δ 8.72 (s, 1H), 8.21 (s, 1H), 8.10 (d, *J* = 7.2 Hz, 2H), 7.64 (t, *J* = 7.6 Hz, 1H), 7.51 (t, *J* = 7.6 Hz, 2H), 7.31 – 7.13 (m, 3H), 6.62 (dd, *J* = 9.2, 5.6 Hz, 1H), 5.77 (dt, *J* = 5.6, 1.6 Hz, 1H), 5.04 – 4.92 (m, 2H), 4.47 (dp, *J* = 3.2, 1.6 Hz, 1H), 4.29 (ddd, *J* = 11.2, 6.8, 2.8 Hz, 1H), 4.16 (ddd, *J* = 11.2, 6.0, 3.2 Hz, 1H), 3.16 (q, *J* = 7.2 Hz, 6H), 3.03 (ddd, *J* = 14.4, 9.2, 5.6 Hz, 1H), 2.72 (ddd, *J* = 14.0, 5.6, 1.6 Hz, 1H), 1.27 (t, *J* = 7.2 Hz, 9H). **^{13}C NMR** (101 MHz, Methanol-*d*$_4$) δ 167.2, 157.3, 153.9, 150.5, 141.2, 139.5 (d, *J* = 8.7 Hz), 134.6, 131.0, 130.7, 129.7, 129.3, 128.6, 128.6, 120.0, 85.7 (d, *J* = 8.9 Hz), 85.5, 78.1, 68.9 (d, *J* = 5.2 Hz), 66.6 (d, *J* = 6.2 Hz), 47.7, 39.2, 9.2. **^{31}P NMR** (162 MHz, Methanol-*d*$_4$) δ 57.8. **IR** (neat, ν/cm^{-1}) = 3323, 3172, 2990, 2354, 1718, 1597, 1473, 1451, 1420, 1315, 1272, 1247, 1214, 1096, 1024, 932, 846, 800, 713, 699, 652; **HRMS** (ESI, m/z): $[M + H]^+$ calcd for $C_{24}H_{25}N_5O_6PS$ = 542.1258; found = 542.1251.

O-(((2R,2R)-5-(6-benzamido-9H-purin-9-yl)-3-hydroxytetrahydrofuran-2-yl)methyl) O-benzyl (S)-phosphorothioate triethylammonium (4a)

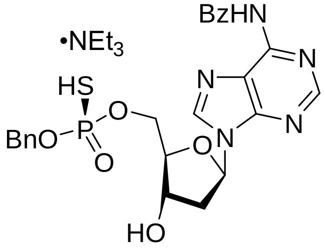

From **1a** under condition B. The chemoselectivity was determined by ^{1}H NMR analysis of the crude sample (**3a**:**4a** = 2:98). The reaction mixture was purified by reversed-phase column chromatography (Biotage®, SNAP Ultra C18 25 g; gradient -0% MeCN (2% NEt_3)/H_2O over 2 CV, 0%-40% MeCN (2% NEt_3)/H_2O over 15 CV.) The acylation product **4a** was obtained as an amorphous solid (59% yield, average of two runs: 39.9 mg, 62%; 36.9 mg, 57%). **^{1}H NMR** (400 MHz, Methanol-*d*$_4$) δ 8.70 (s, 1H), 8.21 (s, 1H), 8.09 (d, *J* = 7.2 Hz, 2H), 7.64 (t, *J* = 7.2 Hz, 1H), 7.51 (t, *J* = 7.6 Hz, 2H), 7.36 (d, *J* = 7.2 Hz, 2H), 7.30 – 7.15 (m, 3H), 6.61 (dd, *J* = 9.2, 5.6 Hz, 1H), 5.74 (dt, *J* = 5.6, 1.6 Hz, 1H), 4.98 (dt, *J* = 7.6, 4.0 Hz, 2H), 4.47 (tt, *J* = 3.2, 1.6 Hz, 1H), 4.30 (ddd, *J* = 11.2, 6.8, 2.8 Hz, 1H), 4.17 (ddd, *J* = 11.2, 6.0, 3.2 Hz, 1H), 3.15 (q, *J* = 7.2 Hz, 6H), 3.02 – 2.93 (m, 1H), 2.72 (ddd, *J* = 14.0, 5.6, 1.6 Hz, 1H), 1.26 (t, *J* = 7.2 Hz, 9H). **^{13}C NMR** (101 MHz, Methanol-*d*$_4$) δ 153.2, 148.3, 144.6, 143.1, 133.9, 132.4, 129.8, 129.4, 129.2, 128.6, 88.2, 85.9 (d, *J* = 2.5 Hz), 73.3, 68.8 (d, *J* = 5.0 Hz), 66.4 (d, *J* = 6.2 Hz), 47.8, 41.6, 9.3. **^{31}P NMR** (162 MHz, Methanol-*d*$_4$) δ 57.8. **IR** (neat, ν/cm^{-1}) = 2985, 2943, 1694, 1600, 1511, 1452, 1377, 1249, 1219, 1158, 1098, 1061, 933,

901, 829, 798, 716, 698, 672; **HRMS** (ESI, m/z): $[M + H]^+$ calcd for $C_{24}H_{25}N_5O_6PS$ = 542.1258; found = 542.1249.

***O*-(((*1R,4R*)-4-(6-amino-9*H*-purin-9-yl)-2-hydroxycyclopentyl)methyl) *O*-benzyl (*R*)-phosphorothioate triethylammonium (3b)**

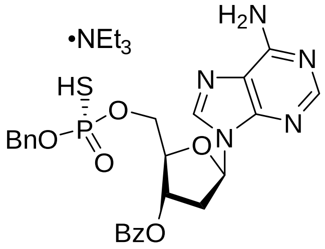

From **1b** under condition A. The chemoselectivity was determined by ^{1}H NMR analysis of the crude sample (**3b**:**4b** = 99:1). The reaction mixture was purified by reversed-phase column chromatography (Biotage®, SNAP Ultra C18 25 g; gradient 0% MeCN (2% NEt_3)/H_2O over 2 CV, 0%-40% MeCN (2% NEt_3)/H_2O over 15 CV.) The acylation product **3b** was obtained as an amorphous solid (83% yield, average of two runs: 53.3 mg, 83%; 53.1 mg, 83%). **^{1}H NMR** (400 MHz, Methanol-*d*4) δ 8.70 (s, 1H), 8.21 (s, 1H), 8.09 (d, *J* = 7.2 Hz, 2H), 7.64 (t, *J* = 7.6 Hz, 1H), 7.51 (t, *J* = 7.6 Hz, 2H), 7.36 (d, *J* = 7.2 Hz, 2H), 7.30 – 7.15 (m, 3H), 6.61 (dd, *J* = 9.0, 5.7 Hz, 1H), 5.74 (dt, *J* = 5.6, 1.6 Hz, 1H), 4.98 (dt, *J* = 7.6, 4.0 Hz, 2H), 4.47 (tt, *J* = 3.2, 1.6 Hz, 1H), 4.30 (ddd, *J* = 11.2, 6.8, 2.8 Hz, 1H), 4.17 (ddd, *J* = 11.2, 6.0, 3.2 Hz, 1H), 3.15 (q, *J* = 7.2 Hz, 6H), 3.02 – 2.93 (m, 1H), 2.72 (ddd, *J* = 14.0, 5.6, 1.6 Hz, 1H), 1.26 (t, *J* = 7.2 Hz, 9H). **^{13}C NMR** (101 MHz, Methanol-*d*4) δ 167.2, 157.3, 153.9, 150.5, 141.1, 139.5 (d, *J* = 8.4 Hz), 134.6, 131.0, 130.7, 129.7, 129.3, 128.6, 120.1, 85.6 (d, *J* = 9.3 Hz), 85.5, 78.0, 68.8 (d, *J* = 5.2 Hz), 66.8 (d, *J* = 6.0 Hz), 47.7, 39.2, 9.2. **^{31}P NMR** (162 MHz, Methanol-*d*4) δ 58.0. **IR** (neat, ν/cm^{-1}) = 3325, 3176, 2990, 1718, 1599, 1473, 1451, 1420, 1316, 1271, 1214, 1096, 1069, 1024, 933, 847, 799, 713, 698; **HRMS** (ESI, m/z): $[M + H]^+$ calcd for $C_{24}H_{25}N_5O_6PS$ = 542.1258; found = 542.1252.

***O*-(((*1R,4R*)-4-(6-benzamido-9*H*-purin-9-yl)-2-hydroxycyclopentyl)methyl) *O*-benzyl (*R*)-phosphorothioate triethylammonium (4b)**

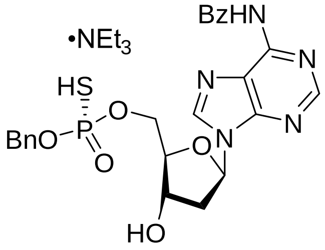

From **1b** under condition B. The chemoselectivity was determined by ^{1}H NMR analysis of the crude sample (**3b**:**4b** = 3:97). The reaction mixture was purified by reversed-phase column chromatography (Biotage®, SNAP Ultra C18 25 g; gradient 0% MeCN (2% NEt_3)/H_2O over 2 CV, 0%-40% MeCN (2% NEt_3)/H_2O over 15 CV.) The acylation product **4b** was obtained as an amorphous solid (59% yield, average of two runs: 38.9 mg, 60%; 37.1 mg, 58%). **^{1}H NMR** (400 MHz, Methanol-*d*4) δ 8.87 (s, 1H), 8.70 (s, 1H), 8.08 (d, *J* = 7.6 Hz, 2H), 7.65 (t, *J* = 7.2 Hz, 1H), 7.56 (t, *J* = 7.6 Hz, 2H), 7.33 (d, *J* = 7.2 Hz, 2H), 7.28 – 7.16 (m, 3H), 6.62 (dd, *J* = 7.6, 6.4 Hz, 1H), 5.00 – 4.92 (m, 2H), 4.65 (dt, *J* = 5.6, 2.4 Hz, 1H), 4.15 (ddt, *J* = 20.0, 10.0, 3.2 Hz, 3H), 3.26 – 3.11 (m, 6H), 2.80 (ddd, *J* = 13.6, 7.6, 5.6 Hz, 1H), 2.49 (ddd, *J* = 13.6, 6.4, 2.8 Hz, 1H), 1.34 – 1.13 (m, 9H). **^{13}C NMR** (101 MHz, Methanol-*d*4) δ 168.8, 152.9, 147.0, 146.3, 139.4 (d, *J* = 8.6 Hz), 134.2, 134.1, 130.7, 129.8,

129.5, 129.3, 128.7, 128.5, 106.5 (d, *J* = 6.1 Hz), 85.5 (d, *J* = 8.0 Hz), 72.7, 68.8 (d, *J* = 5.4 Hz), 67.0 (d, *J* = 5.9 Hz), 47.8, 41.9, 9.2. **^{31}P NMR** (162 MHz, Methanol-d_4) δ 58.0. **IR** (neat, ν/cm^{-1}) = 2990, 1698, 1611, 1582, 1511, 1452, 1314, 1249, 1219, 1159, 1097, 1058, 1013, 933, 900, 834, 798, 732, 713, 699, 554; **HRMS** (ESI, m/z): [M + H]$^+$ calcd for $C_{24}H_{25}N_5O_6PS$ = 542.1258; found = 542.1251.

((*2R,2R*)-5-(6-amino-9*H*-purin-9-yl)-3-(benzoyloxy)tetrahydrofuran-2-yl)methyl benzyl phosphate triethylammonium (3c)

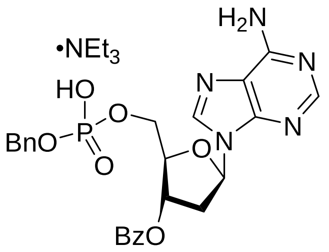

From **1c** under condition A. The chemoselectivity was determined by ^{1}H NMR analysis of the crude sample (**3c**:**4c** = 99:1). The reaction mixture was purified by reversed-phase column chromatography (Biotage®, SNAP Ultra C18 25 g; gradient 0% MeCN (2% NEt_3)/H_2O over 2 CV, 0%-40% MeCN (2% NEt_3)/H_2O over 15 CV.) The acylation product **3c** was obtained as an amorphous solid (81% yield, average of two runs: 49.5 mg, 79%; 51.7 mg, 83%). **^{1}H NMR** (400 MHz, Methanol-d_4) δ 8.56 (s, 1H), 8.21 (s, 1H), 8.10 (d, *J* = 7.2 Hz, 2H), 7.64 (t, *J* = 7.2 Hz, 1H), 7.52 (t, *J* = 7.6 Hz, 2H), 7.33 (d, *J* = 7.2 Hz, 2H), 7.29 – 7.11 (m, 3H), 6.59 (dd, *J* = 8.8, 5.6 Hz, 1H), 5.73 (dt, *J* = 6.0, 1.6 Hz, 1H), 4.90 (d, *J* = 2.4 Hz, 2H), 4.44 (dp, *J* = 3.6, 1.6 Hz, 1H), 4.25 – 4.09 (m, 2H), 3.14 (q, *J* = 7.2 Hz, 6H), 3.09 – 3.02 (m, 1H), 2.73 (ddd, *J* = 14.0, 5.6, 1.6 Hz, 1H), 1.26 (t, *J* = 7.2 Hz, 9H). **^{13}C NMR** (101 MHz, Methanol-d_4) δ 167.2, 157.4, 153.9, 150.6, 140.9, 139.5 (d, *J* = 7.7 Hz), 134.6, 131.0, 130.7, 129.7, 129.3, 128.6, 128.5, 120.2, 85.6 (d, *J* = 8.6 Hz), 85.4, 77.6, 68.5 (d, *J* = 5.4 Hz), 66.6 (d, *J* = 5.5 Hz), 47.7, 38.8, 9.2. **^{31}P NMR** (162 MHz, Methanol-d_4) δ 0.1. **IR** (neat, ν/cm^{-1}) = 3334, 3178, 2990, 1718, 1653, 1599, 1476, 1451, 1420, 1245, 1212, 1052, 1024, 932, 856, 800, 713, 698, 598, 507; **HRMS** (ESI, m/z): [M + H]$^+$ calcd for $C_{24}H_{25}N_5O_7P$ = 526.1487; found = 526.1481.

((*2R,2R*)-5-(6-benzamido-9*H*-purin-9-yl)-3-hydroxytetrahydrofuran-2-yl)methyl benzyl phosphate triethylammonium (4c)

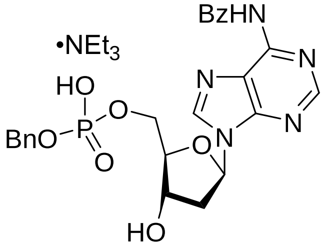

From **1c** under condition B. The chemoselectivity was determined by ^{1}H NMR analysis of the crude sample (**3c**:**4c** = 4:96). The reaction mixture was purified by reversed-phase column chromatography (Biotage®, SNAP Ultra C18 25 g; gradient 0% MeCN (2% NEt_3)/H_2O over 2 CV, 0%-40% MeCN (2% NEt_3)/H_2O over 15 CV.) The acylation product **4c** was obtained as an amorphous solid (56% yield, average of two runs: 35.8 mg, 57%; 35.1 mg, 56%). **^{1}H NMR** (400 MHz, Methanol-d_4) δ 8.56 (s, 1H), 8.21 (s, 1H), 8.10 (d, *J* = 7.2 Hz, 2H), 7.64 (t, *J* = 7.2 Hz, 1H), 7.52 (t, *J* = 7.6 Hz, 2H), 7.33 (d, *J* = 7.2 Hz, 2H), 7.28 – 7.14 (m, 3H), 6.59 (dd, *J* = 8.8, 5.6 Hz, 1H), 5.73 (dt, *J* = 6.0, 1.6 Hz,

1H), 4.90 (d, J = 2.4 Hz, 2H), 4.44 (dp, J = 3.6, 1.6 Hz, 1H), 4.25 – 4.07 (m, 2H), 3.14 (q, J = 7.2 Hz, 6H), 3.09 – 3.01 (m, 1H), 2.73 (ddd, J = 14.0, 5.6, 1.6 Hz, 1H), 1.26 (t, J = 7.2 Hz, 9H). **^{13}C NMR** (101 MHz, Methanol-d_4) δ 168.0, 153.2, 151.0, 144.2, 139.6 (d, J = 8.1 Hz), 135.1, 133.9, 129.8, 129.4, 129.3, 128.6, 128.3, 124.8, 88.0 (d, J = 8.7 Hz), 85.8 (d, J = 2.6 Hz), 73.0, 68.3 (d, J = 5.4 Hz), 66.4, 47.7, 41.1, 9.2. **^{31}P NMR** (162 MHz, Methanol-d_4) δ 0.1. **IR** (neat, ν/cm^{-1}) = 2987, 1694, 1603, 1582, 1453, 1315, 1208, 1051, 1023, 934, 798, 732, 713, 697, 598, 505; **HRMS** (ESI, m/z): $[M + H]^+$ calcd for $C_{24}H_{25}N_5O_7P$ = 526.1487; found = 526.1483.

(*2R,2R*)-5-(6-amino-9*H*-purin-9-yl)-2-(((*tert*-butyldimethylsilyl)oxy)methyl)tetrahydrofuran-3-yl benzoate (3d)

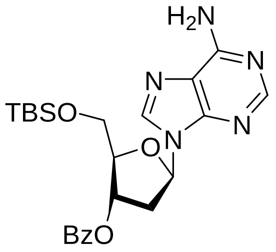

From **1d** under condition A. The chemoselectivity was determined by ^{1}H NMR analysis of the crude sample (**3d**:**4d** > 99:1). The reaction mixture was purified by reversed-phase column chromatography (Biotage®, SNAP Ultra C18 25 g; gradient 0% MeCN/H_2O over 2 CV, 0%-100% MeCN /H_2O over 15 CV.) The acylation product **3d** was obtained as an amorphous solid (90% yield, average of two runs: 41.8 mg, 89%; 42.6 mg, 91%). **^{1}H NMR** (400 MHz, Methanol-d_4) δ 8.36 (s, 1H), 8.22 (s, 1H), 8.12 – 8.05 (m, 2H), 7.64 (ddt, J = 8.8, 7.2, 1.2 Hz, 1H), 7.55 – 7.46 (m, 2H), 6.56 (dd, J = 7.6, 6.0 Hz, 1H), 5.71 (dt, J = 6.0, 2.4 Hz, 1H), 4.38 (td, J = 3.6, 2.0 Hz, 1H), 4.04 – 3.96 (m, 2H), 2.97 (ddd, J = 14.0, 8.0, 6.0 Hz, 1H), 2.81 (ddd, J = 14.0, 6.0, 2.4 Hz, 1H), 0.91 (s, 9H), 0.11 (s, 6H). **^{13}C NMR** (101 MHz, Methanol-d_4) δ 167.3, 157.4, 153.9, 150.3, 140.5, 134.6, 131.0, 130.7, 129.7, 120.4, 87.0, 86.0, 86.0, 76.9, 64.7, 39.4, 26.4, 19.2, –5.3, –5.4. **IR** (neat, ν/cm^{-1}) = 3334, 3170, 2929, 2858, 1718, 1597, 1472, 1451, 1420, 1316, 1211, 1177, 1070, 1026, 1003, 935, 832, 799, 777, 735, 710; **HRMS** (ESI, m/z): $[M + H]^+$ calcd for $C_{23}H_{32}N_5O_4Si$ = 470.2219; found = 470.2213.

***N*-(9-((*2R,2R*)-5-(((*tert*-butyldimethylsilyl)oxy)methyl)-4-hydroxytetrahydrofuran-2-yl)-9*H*-purin-6-yl)benzamide (4d)**

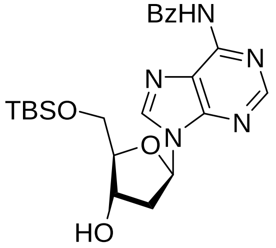

From **1d** under condition B. The chemoselectivity was determined by ^{1}H NMR analysis of the crude sample (**3d**:**4d** = 4:96). The reaction mixture was purified by reversed-phase column chromatography (Biotage®, SNAP Ultra C18 25 g; gradient 0% MeCN/H_2O over 2 CV, 0%-100% MeCN /H_2O over 15 CV.) The acylation product **4d** was obtained as an amorphous solid (61% yield, average of two runs: 28.5 mg, 61%; 29.2 mg, 62%). **^{1}H NMR** (400 MHz, Methanol-d_4) δ 8.71 (s, 1H), 8.63 (s, 1H), 8.13 – 8.04 (m, 2H), 7.70 – 7.62 (m, 1H), 7.61 – 7.52 (m, 2H), 6.57 (t, J = 6.4 Hz, 1H), 4.62 (dt, J = 6.0, 4.0 Hz, 1H), 4.06 (q, J = 3.6 Hz, 1H), 3.92 (ddd, J = 41.6, 11.2, 3.6 Hz, 2H), 2.82 (dt, J = 13.6, 6.0 Hz, 1H), 2.56 (ddd, J = 13.6, 6.4, 4.4 Hz, 1H), 0.90 (s, 9H), 0.08 (s, 6H). **^{13}C NMR** (101 MHz, Methanol-d_4) δ 168.1, 153.2,

153.0, 151.1, 144.0, 135.0, 133.9, 129.8, 129.4, 125.2, 89.2, 86.3, 72.0, 64.3, 41.5, 26.4, 19.3, –5.3. **IR** (neat, ν/cm^{-1}) = 3338, 2952, 2929, 2857, 1611, 1581, 1454, 1329, 1296, 1253, 1217, 1089, 1072, 1002, 935, 834, 798, 779, 735, 709, 668; **HRMS** (ESI, m/z): $[M + H]^+$ calcd for $C_{23}H_{32}N_5O_4Si$ = 470.2219; found = 470.2217.

((*3aR,6aR*)-6-(6-amino-9*H*-purin-9-yl)-2,2-dimethyltetrahydrofuro[3,4-*d*][1,3]dioxol-4-yl)methyl benzoate (3e)

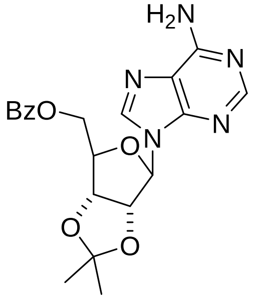

From **1e** under condition A. The chemoselectivity was determined by ^1H NMR analysis of the crude sample (**3e**:**4e** = 99:1). The reaction mixture was purified by reversed-phase column chromatography (Biotage®, SNAP Ultra C18 25 g; gradient 0% MeCN/H_2O over 2 CV, 0%-100% MeCN /H_2O over 15 CV.) The acylation product **3e** was obtained as an amorphous solid (82% yield, average of two runs: 33.2 mg, 80%; 34.9 mg, 85%). **^{1}H NMR** (400 MHz, Methanol-*d*$_4$) δ 8.20 (s, 1H), 8.14 (s, 1H), 7.84 (d, *J* = 7.2 Hz, 2H), 7.54 (t, *J* = 7.6 Hz, 1H), 7.38 (t, *J* = 7.6 Hz, 2H), 6.20 (d, *J* = 2.0 Hz, 1H), 5.60 (dd, *J* = 6.4, 2.0 Hz, 1H), 5.21 (dd, *J* = 6.4, 2.8 Hz, 1H), 4.65 – 4.54 (m, 2H), 4.45 (dd, *J* = 13.2, 6.8 Hz, 1H), 1.60 (s, 3H), 1.40 (s, 3H). **^{13}C NMR** (101 MHz, Methanol-*d*$_4$) δ 167.4, 157.3, 153.9, 150.2, 141.5, 134.4, 130.7, 130.5, 129.5, 120.6, 115.6, 92.4, 86.2, 85.4, 83.0, 65.5, 27.4, 25.5. **IR** (neat, ν/cm^{-1}) = 2990, 1722, 1653, 1559, 1506, 1273, 1213, 1157, 1028, 712; **HRMS** (ESI, m/z): $[M + H]^+$ calcd for $C_{20}H_{22}N_5O_5$ = 412.1616; found = 412.1608.

***N*-(9-((*3aR,6aR*)-6-(hydroxymethyl)-2,2-dimethyltetrahydrofuro[3,4-*d*][1,3]dioxol-4-yl)-9*H*-purin-6-yl)benzamide (4e)**

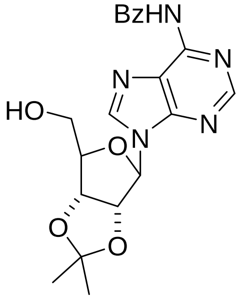

From **1e** under condition B. The chemoselectivity was determined by ^1H NMR analysis of the crude sample (**3e**:**4e** = 8:92). The reaction mixture was purified by reversed-phase column chromatography (Biotage®, SNAP Ultra C18 25 g; gradient 0% MeCN/H_2O over 2 CV, 0%-100% MeCN /H_2O over 15 CV.) The acylation product **4e** was obtained as an amorphous solid (37% yield, average of two runs: 16.0 mg, 39%; 15.0 mg, 36%). **^{1}H NMR** (400 MHz, Methanol-*d*$_4$) δ 8.19 (s, 1H), 8.14 (s, 1H), 7.85 (dd, *J* = 8.4, 1.2 Hz, 2H), 7.58 – 7.52 (m, 1H), 7.38 (t, *J* = 7.6 Hz, 2H), 6.20 (d, *J* = 2.0 Hz, 1H), 5.61 (dd, *J* = 6.4, 2.0 Hz, 1H), 5.21 (dd, *J* = 6.4, 2.8 Hz, 1H), 4.64 – 4.55 (m, 2H), 4.50 – 4.42 (m, 1H), 1.61 (s, 3H), 1.41 (s, 3H). **^{13}C NMR** (101 MHz, Methanol-*d*$_4$) δ 167.4, 157.3, 154.0, 150.2, 141.5, 134.4, 130.7, 130.5, 129.5, 120.7, 115.6, 92.4, 86.2, 85.4, 83.0, 65.5, 27.4, 25.5. **IR** (neat, ν/cm^{-1}) = 3350, 3062, 2990, 1709, 1605, 1591, 1464, 1380, 1371, 1327, 1292, 1260, 1212, 1191, 1158, 1057, 1028, 1003, 931, 875, 846, 828, 800, 785, 736, 701, 688, 645; **HRMS** (ESI, m/z): $[M + H]^+$ calcd for $C_{20}H_{22}N_5O_5$ = 412.1616; found = 412.1609.

(9*R*,9*aR*)-8-(6-amino-9*H*-purin-9-yl)-2,2,4,4-tetraisopropyltetrahydro-6*H*-furo[3,2-*f*][1,3,5,2,4]trioxadisilocin-9-yl benzoate (3f)

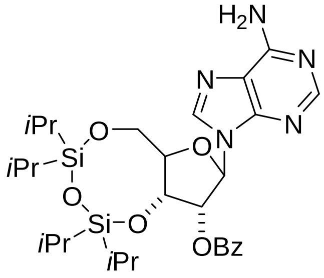

From **1f** under condition A. The chemoselectivity was determined by ^{1}H NMR analysis of the crude sample (**3f**:**4f** = 99:1). The reaction mixture was purified by reversed-phase column chromatography (Biotage®, SNAP Ultra C18 25 g; gradient 0% MeCN/H_2O over 2 CV, 0%-100% MeCN /H_2O over 15 CV.) The acylation product **3f** was obtained as an amorphous solid (89% yield, average of two runs: 54.8 mg, 89%; 55.1 mg, 90%). **^{1}H NMR** (400 MHz, Chloroform-*d*) δ 8.30 (s, 1H), 8.12 – 8.06 (m, 2H), 8.01 (s, 1H), 7.62 – 7.55 (m, 1H), 7.46 (t, J = 7.6 Hz, 2H), 6.17 (d, *J* = 1.2 Hz, 1H), 6.08 – 5.93 (m, 3H), 5.28 – 5.20 (m, 1H), 4.27 – 4.15 (m, 2H), 4.11 – 4.02 (m, 1H), 1.21 – 0.74 (m, 28H). **^{13}C NMR** (101 MHz, Chloroform-*d*) δ 165.4, 155.5, 152.9, 149.4, 139.6, 133.5, 130.0, 129.7, 128.6, 120.4, 87.8, 87.8, 82.4, 76.5, 69.6, 61.0, 17.6, 17.51, 17.48, 17.4, 17.1, 17.0, 16.93, 16.91, 13.4, 13.1, 12.9, 12.8. **IR** (neat, ν/cm^{-1}) = 2944, 2867, 1730, 1601, 1465, 1420, 1331, 1264, 1158, 1120, 1034, 988, 903, 883, 857, 698; **HRMS** (ESI, m/z): [M + H]$^+$ calcd for $C_{29}H_{44}N_5O_6Si_2$ = 614.2825; found = 614.2816.

***N*-(9-((9*R*,9*aS*)-9-hydroxy-2,2,4,4-tetraisopropyltetrahydro-6*H*-furo[3,2-f][1,3,5,2,4]trioxadisilocin-8-yl)-9*H*-purin-6-yl)benzamide (4f)**

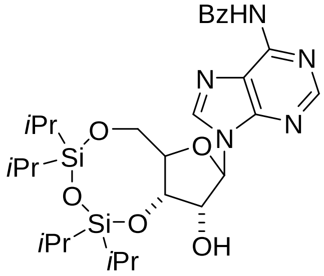

From **1f** under condition B. The chemoselectivity was determined by ^{1}H NMR analysis of the crude sample (**3f**:**4f** = 2:98). The reaction mixture was purified by reversed-phase column chromatography (Biotage®, SNAP Ultra C18 25 g; gradient 0% MeCN/H_2O over 2 CV, 0%-100% MeCN /H_2O over 15 CV.) The acylation product **4f** was obtained as an amorphous solid (61% yield, average of two runs: 36.0 mg, 59%; 38.5 mg, 63%). **^{1}H NMR** (400 MHz, Methanol-*d*$_4$) δ 8.68 (s, 1H), 8.56 (s, 1H), 8.09 (d, *J* = 7.6 Hz, 2H), 7.66 (t, *J* = 7.6 Hz, 1H), 7.57 (t, *J* = 7.6 Hz, 2H). 6.09 (d, *J* = 1.1 Hz, 1H), 4.91 (d, *J* = 4.9 Hz, 1H), 4.69 (d, *J* = 5.0 Hz, 1H), 4.24 – 4.15 (m, 2H), 4.11 – 4.05 (m, 1H), 1.25 – 0.91 (m, 28H). **^{13}C NMR** (101 MHz, Methanol-*d*$_4$) δ 168.1, 153.3, 152.6, 151.2, 148.4, 144.3, 143.5, 133.9, 129.8, 129.4, 91.8, 83.1, 75.7, 71.2, 62.1, 18.0, 17.97, 17.92, 17.8, 17.6, 17.5, 17.4, 14.6, 14.3, 14.2, 13.8. **IR** (neat, ν/cm^{-1}) = 2945, 2868, 1613, 1583, 1366, 1337, 1290, 1249, 1122, 1035, 990, 883, 857, 822, 798, 774, 735, 693, 645, 554; **HRMS** (ESI, m/z): [M + H]$^+$ calcd for $C_{29}H_{44}N_5O_6Si_2$ = 614.2825; found = 614.2817.

(2*R*,5*R*)-5-(4-amino-2-oxopyrimidin-1(2*H*)-yl)-2-(((*tert*-butyldimethylsilyl)oxy)methyl)tetrahydrofuran-3-yl benzoate (3g)

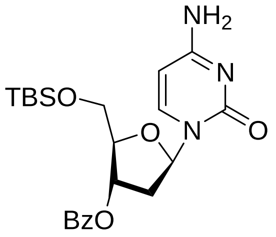

From **1g** under condition A. The chemoselectivity was determined by ^{1}H NMR analysis of the crude product (**3g**:**4g** = 87:13). The reaction mixture was purified by reversed-phase column chromatography (Biotage®, SNAP Ultra C18 25 g; gradient 0% MeCN/H_2O over 2 CV, 0%-100% MeCN /H_2O over 15 CV.) A mixture of acylation products **3g** and **4g** was obtained as an amorphous solid (17% yield, average of two runs: 7.8 mg, 17%; 7.6 mg, 17%). **^{1}H NMR** (400 MHz, Methanol-d_4) δ 8.07 (d, *J* = 7.2 Hz, 2H), 8.03 (d, *J* = 7.6 Hz, 1H), 7.67 – 7.62 (m, 1H), 7.51 (t, *J* = 7.6 Hz, 2H), 6.38 (dd, *J* = 8.0, 5.6 Hz, 1H), 5.92 (d, *J* = 7.6 Hz, 1H), 5.53 (dt, *J* = 6.4, 2.0 Hz, 1H), 4.33 (q, *J* = 2.4 Hz, 1H), 4.01 (d, *J* = 2.8 Hz, 2H), 2.73 – 2.67 (m, 1H), 2.31 – 2.24 (m, 1H), 0.95 (s, 9H), 0.17 (s, 6H). **^{13}C NMR** (101 MHz, Methanol-d_4) δ 167.4, 158.2, 142.1, 134.6, 132.3, 130.9, 130.7, 129.7, 87.7, 87.0, 77.1, 73.6, 64.7, 47.5, 40.1, 26.4, 19.2, –5.4, –5.5. **IR** (neat, ν/cm^{-1}) = 3328, 2928, 2857, 1718, 1487, 1408, 1363, 1315, 1200, 1177, 1070, 1026, 1004, 941, 834, 779, 711; **HRMS** (ESI, m/z): [M + H]$^+$ calcd for $C_{22}H_{32}N_3O_5Si$ = 446.2106; found = 446.2100.

***N*-(1-((2*R*,5*R*)-5-(((*tert*-butyldimethylsilyl)oxy)methyl)-4-hydroxytetrahydrofuran-2-yl)-2-oxo-1,2-dihydropyrimidin-4-yl)benzamide (4g)**

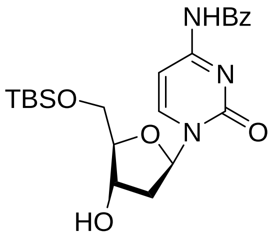

From **1g** under condition C. The chemoselectivity was determined by ^{1}H NMR analysis of the crude sample (**3g**:**4g** = 1:99). The reaction mixture was purified by reversed-phase column chromatography (Biotage®, SNAP Ultra C18 25 g; gradient 0% MeCN/H_2O over 2 CV, 0%-100% MeCN /H_2O over 15 CV.) The acylation product **4g** was obtained as an amorphous solid (97% yield, average of two runs: 44.3 mg, 99%; 42.4 mg, 95%). **^{1}H NMR** (400 MHz, Methanol-d_4) δ 8.53 (d, *J* = 7.6 Hz, 1H), 8.04 – 7.93 (m, 2H), 7.67 – 7.48 (m, 4H), 6.21 (t, *J* = 6.0 Hz, 1H), 4.38 (dt, *J* = 6.0, 4.2 Hz, 1H), 4.05 (dd, *J* = 4.0, 2.4 Hz, 1H), 4.00 (dd, *J* = 11.6, 2.8 Hz, 1H), 3.88 (dd, *J* = 11.6, 2.8 Hz, 1H), 2.56 (ddd, *J* = 13.6, 6.4, 4.4 Hz, 1H), 2.20 (dt, *J* = 13.6, 6.0 Hz, 1H), 0.94 (s, 9H), 0.15 (d, *J* = 5.6 Hz, 6H). **^{13}C NMR** (101 MHz, Methanol-d_4) δ 169.1, 164.7, 157.7, 146.2, 134.7, 134.1, 129.8, 129.2, 98.1, 89.4, 88.8, 71.3, 63.8, 42.9, 26.4, 19.2, –5.4, –5.4. **IR** (neat, ν/cm^{-1}) = 2955, 2929, 2858, 1649, 1619, 1481, 1432, 1393, 1312, 1253, 1185, 1124, 1090, 1071, 1002, 937, 834, 810, 783, 733, 702, 598; **HRMS** (ESI, m/z): [M + H]$^+$ calcd for $C_{22}H_{32}N_3O_5Si$ = 446.2106; found = 446.2101.

(*2R*,*5R*)-5-(4-benzamido-2-oxopyrimidin-1(2*H*)-yl)-2-(((*tert*-butyldimethylsilyl)oxy)methyl)tetrahydrofuran-3-yl benzoate (5g)

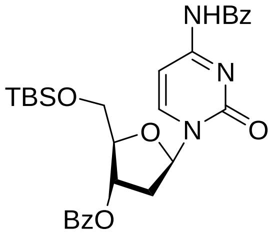

From **1g** under condition A. The reaction mixture was purified by reversed-phase column chromatography (Biotage®, SNAP Ultra C18 25 g; gradient 0% MeCN/H_2O over 2 CV, 0%-100% MeCN /H_2O over 15 CV.) The acylation product **5g** was obtained as an amorphous solid (42% yield, average of two runs: 21.4 mg, 39%; 25.1 mg, 46%). **^{1}H NMR** (400 MHz, DMSO-d_6) δ 11.29 (s, 1H), 8.32 (d, *J* = 7.6 Hz, 1H), 8.05 – 8.00 (m, 4H), 7.73 – 7.67 (m, 1H), 7.66 – 7.60 (m, 1H), 7.59 – 7.48 (m, 4H), 7.40 (d, *J* = 7.2 Hz, 1H), 6.28 (dd, *J* = 7.2, 6.0 Hz, 1H), 5.47 (dt, *J* = 6.4, 2.4 Hz, 1H), 4.40 (q, *J* = 3.2 Hz, 1H), 3.94 (qd, *J* = 11.6, 3.6 Hz, 2H), 2.74 (ddd, *J* = 14.4, 6.0, 2.4 Hz, 1H), 2.37 (dt, *J* = 14.0, 6.8 Hz, 1H), 0.88 (s, 9H), 0.11 (d, *J* = 1.6 Hz, 6H). **^{13}C NMR** (101 MHz, DMSO-d_6) δ 168.6, 167.4, 165.2, 157.7, 144.5, 144.4, 133.7, 132.8, 129.4, 129.3, 128.8, 128.5, 86.7, 85.1, 75.2, 63.0, 39.1, 25.7, 17.9, -5.6, -5.7. **IR** (neat, ν/cm^{-1}) = 2955, 2929, 2859, 1721, 1698, 1667, 1620, 1602, 1483, 1451, 1398, 1349, 1325, 1315, 1248, 1200, 1106, 1071, 1024, 1003, 954, 943, 892, 865, 829, 812, 737, 708; **HRMS** (ESI, m/z): $[M + H]^+$ calcd for $C_{29}H_{36}N_3O_6Si$ = 550.2368; found = 550.2357.

(*2R*,*5R*)-5-(2-amino-6-oxo-1,6-dihydro-9*H*-purin-9-yl)-2-(((*tert*-butyldimethylsilyl)oxy)methyl)tetrahydrofuran-3-yl benzoate (3h)

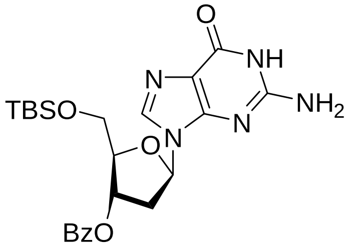

From **1h** under condition A. The chemoselectivity was determined by ^{1}H NMR analysis of the crude sample (**3h**:**4h** = 99:1). The reaction mixture was purified by reversed-phase column chromatography (Biotage®, SNAP Ultra C18 25 g; gradient 0% MeCN/H_2O over 2 CV, 0%-100% MeCN /H_2O over 15 CV.) The acylation product **3h** was obtained as an amorphous solid (73% yield, average of two runs: 34.4 mg, 71%; 36.2 mg, 75%). **^{1}H NMR** (400 MHz, DMSO-d_6) δ 10.68 (s, 1H), 8.07 – 7.99 (m, 2H), 7.94 (s, 1H), 7.74 – 7.67 (m, 1H), 7.57 (t, *J* = 7.6 Hz, 2H), 6.52 (s, 2H), 6.23 (dd, *J* = 8.4, 5.6 Hz, 1H), 5.54 (dt, *J* = 6.0, 2.0 Hz, 1H), 4.24 (td, *J* = 4.8, 2.0 Hz, 1H), 3.85 (d, *J* = 4.8 Hz, 2H), 2.89 (ddd, *J* = 14.4, 8.8, 6.0 Hz, 1H), 2.63 (ddd, *J* = 14.0, 6.0, 2.0 Hz, 1H), 0.86 (s, 9H), 0.05 (d, *J* = 3.6 Hz, 6H). **^{13}C NMR** (101 MHz, DMSO-d_6) δ 165.1, 156.6, 153.8, 151.0, 134.7, 133.6, 129.33, 129.31, 128.8, 116.5, 84.4, 82.7, 75.4, 63.3, 48.6, 36.6, 25.8, 18.0, –5.46, –5.52. **IR** (neat, ν/cm^{-1}) = 2981, 2901, 2368, 1653, 1449, 1387, 1250, 1082, 1067, 1046, 880, 668; **HRMS** (ESI, m/z): $[M + H]^+$ calcd for $C_{23}H_{32}N_5O_5Si$ = 486.2168; found = 486.2164.

***O*-(((2*R*,5*R*)-3-acetoxy-5-(6-amino-9*H*-purin-9-yl)tetrahydrofuran-2-yl)methyl) *O*-benzyl (*S*)-phosphorothioate triethylammonium (3ab)**

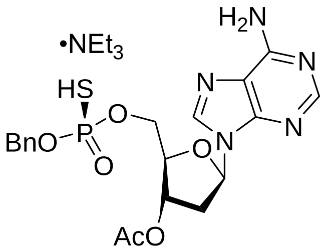

From **1a** and **2b** under condition A. The chemoselectivity was determined by ^{1}H NMR analysis of the crude sample (**3ab**:**4ab** > 99:1). The reaction mixture was purified by reversed-phase column chromatography (Biotage®, SNAP Ultra C18 25 g; gradient 0% MeCN (2% NEt_3)/H_2O over 2 CV, 0%-40% MeCN (2% NEt_3)/H_2O over 15 CV.) The acylation product **3ab** was obtained as an amorphous solid (92% yield, average of two runs: 53.8 mg, 93%; 52.8 mg, 91%). **^{1}H NMR** (400 MHz, Methanol-*d*$_4$) δ 8.68 (s, 1H), 8.20 (s, 1H), 7.34 (d, *J* = 7.2 Hz, 1H), 7.30 – 7.17 (m, 3H), 6.49 (dd, *J* = 9.2, 5.6 Hz, 1H), 5.51 (dt, *J* = 5.6, 1.6 Hz, 1H), 5.02 – 4.91 (m, 2H), 4.32 (dp, *J* = 3.2, 1.5 Hz, 1H), 4.14 (dddd, *J* = 45.2, 11.2, 6.4, 3.2 Hz, 2H), 3.16 (q, *J* = 7.2 Hz, 6H), 2.89 (ddd, *J* = 14.4, 9.2, 6.0 Hz, 1H), 2.56 (ddd, *J* = 14.0, 6.0, 1.6 Hz, 1H), 2.11 (s, 3H), 1.27 (t, *J* = 7.2 Hz, 9H). **^{13}C NMR** (101 MHz, Methanol-*d*$_4$) δ 172.0, 157.3, 153.9, 150.5, 141.1, 139.5 (d, *J* = 8.7 Hz), 129.3, 128.6, 120.0, 85.6 (d, *J* = 9.1 Hz), 85.3 (d, *J* = 3.3 Hz), 77.4, 77.4, 68.9 (d, *J* = 5.2 Hz), 66.6 (d, *J* = 5.9 Hz), 47.7, 39.1, 20.9, 9.2. **^{31}P NMR** (162 MHz, Methanol-*d*$_4$) δ 57.8. **IR** (neat, ν/cm^{-1}) = 3318, 3178, 2985, 2370, 1738, 1645, 1596, 1576, 1498, 1474, 1454, 1419, 1334, 1297, 1234, 1158, 1109, 1017, 969, 935, 919, 883, 829, 800, 730, 698, 652; **HRMS** (ESI, m/z): [M + H]$^+$ calcd for $C_{19}H_{23}N_5O_6PS$ = 480.1102; found = 480.1093.

***O*-(((2*R*,5*R*)-5-(6-acetamido-9*H*-purin-9-yl)-3-hydroxytetrahydrofuran-2-yl)methyl) *O*-benzyl (*S*)-phosphorothioate triethylammonium (4ab)**

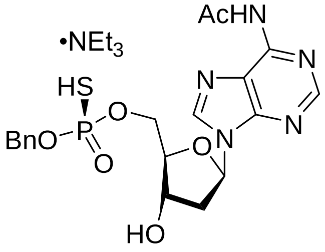

From **1a** and **2b** under condition B. The chemoselectivity was determined by ^{1}H NMR analysis of the crude sample (**3ab**:**4ab** = 17:83). The reaction mixture was purified by reversed-phase column chromatography (Biotage®, SNAP Ultra C18 25 g; gradient 0% MeCN (2% NEt_3)/H_2O over 2 CV, 0%-40% MeCN (2% NEt_3)/H_2O over 15 CV.) The acylation product **4ab** and substrate **1a** was obtained as an in separable mixture, yield was detected by **^{1}H NMR** (42% yield, average of two runs: 43%; 41%). **^{1}H NMR** (400 MHz, Methanol-*d*$_4$) δ 8.83 (s, 1H), 8.61 (s, 1H), 7.33 – 7.22 (m, 5H), 6.60 (dd, *J* = 7.6, 6.0 Hz, 1H), 4.99 – 4.93 (m, 2H), 4.67 – 4.63 (m, 1H), 4.16 – 4.06 (m, 3H), 3.17 (q, *J* = 7.2 Hz, 6H), 2.84 – 2.77 (m, 1H), 2.49 – 2.40 (m, 1H), 2.37 (s, 3H), 1.28 (t, *J* = 7.3 Hz, 9H). **^{13}C NMR** (101 MHz, Methanol-d4) δ 171.9, 157.2, 153.8, 153.1, 152.9, 150.4, 144.3, 129.2, 128.6, 123.8, 88.2 (d, *J* = 8.9 Hz), 85.8, 73.3, 68.8 (d, *J* = 5.3 Hz), 66.5 (d, *J* = 6.1 Hz), 47.7, 41.5, 24.7, 9.2. **^{31}P NMR** (162 MHz, Methanol-*d*$_4$) δ 57.8. **IR** (neat, ν/cm^{-1}) = 3334, 2981, 1607, 1454, 1372, 1332, 1297, 1216, 1039, 935, 800, 741, 699, 574. **HRMS** (ESI, m/z): [M + H]$^+$ calcd for $C_{19}H_{23}N_5O_6PS$ = 480.1102; found = 480.1096.

***O*-(((2*R*,2*R*)-5-(6-amino-9*H*-purin-9-yl)-3-(benzoyloxy)tetrahydrofuran-2-yl)methyl) *O*-benzyl (*S*)-phosphorothioate triethylammonium (3a)**

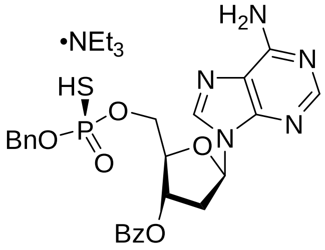

From **1a** and **2c** under condition A. The chemoselectivity was determined by ^{1}H NMR analysis of the crude sample (**3a**:**4a** = 91:9). The reaction mixture was purified by reversed-phase column chromatography (Biotage®, SNAP Ultra C18 25 g; gradient 0% MeCN (2% NEt_3)/H_2O over 2 CV, 0%-40% MeCN (2% NEt_3)/H_2O over 15 CV.) The acylation product **3a** was obtained as an amorphous solid (38% yield, average of two runs: 24.3 mg, 38%; 25.0 mg, 39%).

***O*-(((2*R*,2*R*)-5-(6-benzamido-9*H*-purin-9-yl)-3-hydroxytetrahydrofuran-2-yl)methyl) *O*-benzyl (*S*)-phosphorothioate triethylammonium (4a)**

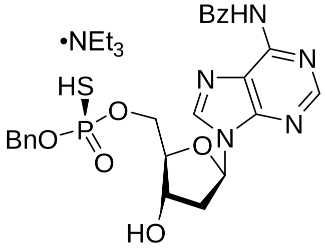

From **1a** and **2c** under condition B. The chemoselectivity was determined by ^{1}H NMR analysis of the crude sample (**3a**:**4a** = 8:92). The reaction mixture was purified by reversed-phase column chromatography (Biotage®, SNAP Ultra C18 25 g; gradient 0% MeCN (2% NEt_3)/H_2O over 2 CV, 0%-40% MeCN (2% NEt_3)/H_2O over 15 CV.) The acylation product **4a** was obtained as an amorphous solid (31% yield, average of two runs: 20.3 mg, 32%; 19.1 mg, 30%).

***O*-(((2*R*,2*R*)-5-(6-amino-9*H*-purin-9-yl)-3-(benzoyloxy)tetrahydrofuran-2-yl)methyl) *O*-benzyl (*S*)-phosphorothioate triethylammonium (3a)**

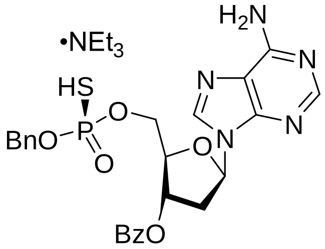

From **1a** and **2e** under condition A. The chemoselectivity was determined by ^{1}H NMR analysis of the crude sample (**3a**:**4a** > 99:1). The reaction mixture was purified by reversed-phase column chromatography (Biotage®, SNAP Ultra C18 25 g; gradient 0% MeCN (2% NEt_3)/H_2O over 2 CV, 0%-40% MeCN (2% NEt_3)/H_2O over 15 CV.) The acylation product **3a** was obtained as an amorphous solid (42% yield, average of two runs: 27.1 mg, 42%; 27.6 mg, 43%).

***O*-(((2*R*,2*R*)-5-(6-benzamido-9*H*-purin-9-yl)-3-hydroxytetrahydrofuran-2-yl)methyl) *O*-benzyl (*S*)-phosphorothioate triethylammonium (4a)**

From **1a** and **2e** under condition B. The chemoselectivity was determined by ^{1}H NMR analysis of the crude sample (**3a**:**4a** = 1:99). The reaction mixture was purified by reversed-phase column chromatography (Biotage®, SNAP Ultra C18 25 g; gradient 0% MeCN (2% NEt_3)/H_2O over 2 CV,

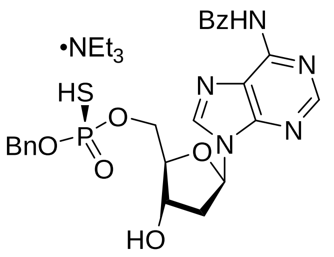

0%-40% MeCN (2% NEt_3)/H_2O over 15 CV.) The acylation product **4a** was obtained as an amorphous solid (10% yield, average of two runs: 6.7 mg, 10%; 6.1 mg, 10%).

***O*-(((2*R*,5*R*)-5-(6-amino-9*H*-purin-9-yl)-3-((1-((*tert*-butoxycarbonyl)amino)cyclopropane-1-carbonyl)oxy)tetrahydrofuran-2-yl)methyl) *O*-benzyl (*S*)-phosphorothioate triethylammonium (3af)**

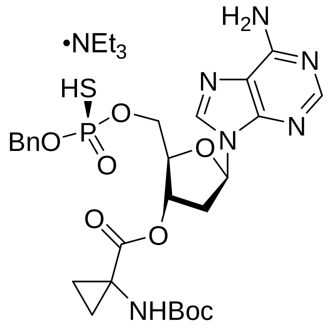

From **1a** and **2f** under condition A. The chemoselectivity was determined by ^{1}H NMR analysis of the crude sample (**3af**:**4af** = 97:3). The reaction mixture was purified by reversed-phase column chromatography (Biotage®, SNAP Ultra C18 25 g; gradient 0% MeCN (2% NEt_3)/H_2O over 2 CV, 0%-40% MeCN (2% NEt_3)/H_2O over 15 CV.) The acylation product **3af** was obtained as an amorphous solid (63% yield, average of two runs: 45.8 mg, 64%; 45.2 mg, 63%). **^{1}H NMR** (400 MHz, Methanol-*d*$_4$) δ 8.68 (s, 1H), 8.19 (s, 1H), 7.39 – 7.30 (m, 2H), 7.30 – 7.17 (m, 3H), 6.50 (dd, *J* = 9.2, 5.6 Hz, 1H), 5.53 (d, *J* = 5.6 Hz, 1H), 5.03 – 4.91 (m, 2H), 4.30 (s, 1H), 4.22 – 4.04 (m, 2H), 3.16 (q, *J* = 7.2 Hz, 6H), 2.89 (ddd, *J* = 14.4, 9.2, 5.6 Hz, 1H), 2.54 (dd, *J* = 14.0, 5.6 Hz, 1H), 1.63 – 1.37 (m, 11H), 1.28 (t, *J* = 7.2 Hz, 9H), 1.16 (q, *J* = 4.2 Hz, 2H). **^{13}C NMR** (101 MHz, Methanol-*d*$_4$) δ 174.1, 158.9, 157.2, 153.9, 150.6, 141.1, 139.5 (d, *J* = 8.7 Hz), 129.3, 128.64, 128.60, 119.9, 85.6 (d, *J* = 9.0 Hz), 85.3, 80.8, 78.3, 68.9 (d, *J* = 5.3 Hz), 66.6 (d, *J* = 6.8 Hz), 47.7, 39.1, 35.0, 28.7, 18.4, 9.2. **^{31}P NMR** (162 MHz, Methanol-*d*$_4$) δ 57.8. **IR** (neat, ν/cm^{-1}) = 3333, 3178, 2981, 1645, 1597, 1576, 1498, 1475, 1454, 1417, 1392, 1367, 1336, 1295, 1266, 1248, 1215, 1154, 1109, 1034, 961, 932, 839, 800, 731, 698, 667, 654; **HRMS** (ESI, m/z): $[M + H]^+$ calcd for $C_{26}H_{34}N_6O_8PS$ = 621.1891; found = 621.1882.

***O*-benzyl *O*-(((2*R*,5*R*)-5-(6-(1-((*tert*-butoxycarbonyl)amino)cyclopropane-1-carboxamido)-9*H*-purin-9-yl)-3-hydroxytetrahydrofuran-2-yl)methyl) (*S*)-phosphorothioate triethylammonium (4af)**

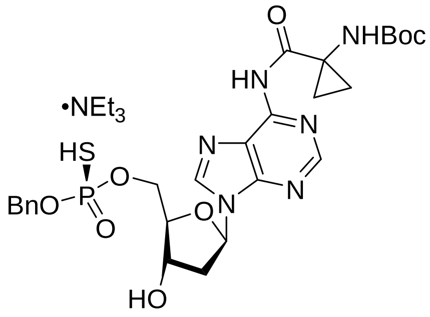

From **1a** and **2f** under condition B. The chemoselectivity was determined by ^{1}H NMR analysis of the crude sample (**3af**:**4af** = 12:88). The reaction mixture was purified by reversed-phase column chromatography (Biotage®, SNAP Ultra C18 25 g; gradient 0% MeCN (2% NEt_3)/H_2O over 2 CV, 0%-40% MeCN (2% NEt_3)/H_2O over 15 CV.) The acylation product **4af** was obtained as an amorphous solid (54% yield, average of two runs: 39.9 mg, 55%; 38.4 mg, 53%). **^{1}H NMR** (400 MHz, Methanol-*d*$_4$) δ 8.83 (s, 1H), 8.61 (s, 1H), 7.32 (d, *J* = 6.8 Hz, 1H), 7.27 – 7.17 (m, 3H), 6.60 (dd, *J* = 7.6, 6.4 Hz, 1H), 4.96 (dd, *J* = 12.0, 8.4 Hz, 2H), 4.66 (dt, *J* = 5.6, 2.8 Hz, 1H), 4.19 – 4.02 (m, 3H), 3.17 (q, *J* = 7.2 Hz, 6H), 2.80 (ddd, *J* = 13.6, 7.6, 5.6 Hz, 1H), 2.46 (ddd, *J* = 13.6, 6.0, 2.8

Hz, 1H), 1.64 (q, J = 4.4 Hz, 2H), 1.47 – 1.40 (m, 10H), 1.28 (t, J = 7.2 Hz, 9H), 1.21 (q, J = 4.4 Hz, 2H). **^{13}C NMR** (101 MHz, Methanol-d_4) δ 153.1, 152.8, 150.0, 144.4, 139.5 (d, J = 8.7 Hz), 129.2, 128.62, 128.60, 88.1 (d, J = 9.2 Hz), 85.8, 73.4, 68.8 (d, J = 5.1 Hz), 66.5 (d, J = 6.7 Hz), 60.0, 47.7, 41.5, 28.7, 19.2, 9.2. **^{31}P NMR** (162 MHz, Methanol-d_4) δ 57.8. **IR** (neat, ν/cm^{-1}) = 2980, 1712, 1609, 1453, 1367, 1249, 1160, 1060, 1023, 935, 800, 736, 699; **HRMS** (ESI, m/z): [M + H]$^+$ calcd for $C_{26}H_{34}N_6O_8PS$ = 621.1891; found = 621.1883.

***O*-(((2*R*,5*R*)-5-(6-amino-9*H*-purin-9-yl)-3-(((*tert*-butoxycarbonyl)-*L*-phenylalanyl)oxy) tetrahydrofuran-2-yl)methyl) *O*-benzyl (*S*)-phosphorothioate triethylammonium (3ag)**

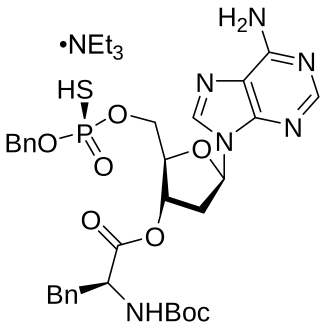

From **1a** and **2g** under condition A. The chemoselectivity was determined by ^{1}H NMR analysis of the crude sample (**3ag**:**4ag** = 99:1). The reaction mixture was purified by reversed-phase column chromatography (Biotage®, SNAP Ultra C18 25 g; gradient 0% MeCN (2% NEt_3)/H_2O over 2 CV, 0%-40% MeCN (2% NEt_3)/H_2O over 15 CV.) The acylation product **3ag** was obtained as an amorphous solid (88% yield, average of two runs: 68.5 mg, 87%; 70.3 mg, 89%). **^{1}H NMR** (400 MHz, Methanol-d_4) δ 8.66 (s, 1H), 8.20 (s, 1H), 7.41 – 7.13 (m, 10H), 6.42 (dd, J = 8.8, 5.6 Hz, 1H), 5.48 (d, J = 5.6 Hz, 1H), 5.04 – 4.91 (m, 2H), 4.43 – 4.36 (m, 1H), 4.17 – 4.00 (m, 3H), 3.14 (q, J = 7.2 Hz, 6H), 3.10 – 2.95 (m, 2H), 2.90 – 2.83 (m, 1H), 2.52 (dd, J = 14.0, 5.6 Hz, 1H), 1.42 (s, 9H), 1.27 (t, J = 7.2 Hz, 9H). **^{13}C NMR** (101 MHz, Methanol-d_4) δ 173.1, 157.8, 157.2, 153.9, 150.6, 141.1, 139.5 (d, J = 8.5 Hz), 130.4, 129.6, 129.3, 128.6, 128.0, 120.0, 85.3, 85.2, 80.7, 78.1 (d, J = 4.8 Hz), 68.9 (d, J = 5.3 Hz), 66.5 (d, J = 4.6 Hz), 56.8, 47.7, 39.0, 38.7, 28.7, 9.3. **^{31}P NMR** (162 MHz, Methanol-d_4) δ 57.8. **IR** (neat, ν/cm^{-1}) = 2982, 1743, 1708, 1643, 1597, 1498, 1475, 1454, 1421, 1393, 1367, 1324, 1296, 1265, 1244, 1213, 1157, 1110, 976, 933, 875, 800, 731, 698; **HRMS** (ESI, m/z): [M + H]$^+$ calcd for $C_{31}H_{38}N_6O_8PS$ = 685.2204; found = 685.2202.

***O*-benzyl *O*-(((2*R*,5*R*)-5-(6-((*S*)-2-((*tert*-butoxycarbonyl)amino)-3-phenylpropanamido)-9*H*-purin-9-yl)-3-hydroxytetrahydrofuran-2-yl)methyl) (*S*)-phosphorothioate triethylammonium (4ag)**

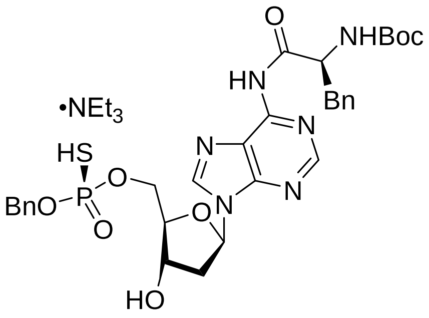

From **1a** and **2g** under condition B. The chemoselectivity was determined by ^{1}H NMR analysis of the crude sample (**3ag**:**4ag** = 8:92). The reaction mixture was purified by reversed-phase column chromatography (Biotage®, SNAP Ultra C18 25 g; gradient 0% MeCN (2% NEt_3)/H_2O over 2 CV, 0%-40% MeCN (2% NEt_3)/H_2O over 15 CV.) The acylation product **4ag** was obtained as an amorphous solid

(44% yield, average of two runs: 34.9 mg, 44%; 35.6 mg, 45%). **^{1}H NMR** (400 MHz, Methanol-*d*$_4$) δ 8.85 (s, 1H), 8.63 (s, 1H), 7.38 – 7.17 (m, 10H), 6.60 (t, *J* = 6.8 Hz, 1H), 4.96 (dd, *J* = 12.0, 8.4 Hz, 2H), 4.80 (s, 1H), 4.67 (dt, *J* = 5.6, 2.4 Hz, 1H), 4.17 – 4.05 (m, 3H), 3.26 (d, *J* = 6.8 Hz, 1H), 3.17 (q, *J* = 7.2 Hz, 6H), 3.06 – 2.93 (m, 2H), 2.80 (ddd, *J* = 13.6, 7.6, 5.6 Hz, 1H), 2.46 (ddd, *J* = 13.6, 6.4, 2.8 Hz, 1H), 1.42 – 1.21 (m, 18H). **^{13}C NMR** (101 MHz, Methanol-*d*$_4$) δ 173.0, 157.8153.0, 150.1, 144.3, 139.5 (d, *J* = 8.8 Hz), 138.4, 130.5, 129.4, 129.2, 128.6, 127.7, 123.8, 88.2 (d, *J* = 10.0 Hz), 85.8 (d, *J* = 4.6 Hz), 80.7, 73.4, 68.8 (d, *J* = 5.4 Hz), 66.5, 60.1, 47.8, 41.5, 39.0, 28.7, 9.3. **^{31}P NMR** (162 MHz, Methanol-*d*$_4$) δ 57.8. **IR** (neat, ν/cm^{-1}) = 2982, 1703, 1612, 1583, 1454, 1393, 1367, 1161, 1057, 935, 800, 738, 700; **HRMS** (ESI, m/z): [M + H]$^+$ calcd for $C_{31}H_{38}N_6O_8PS$ = 685.2204; found = 685.2195.

(2*R*,5R)-5-(6-amino-9*H*-purin-9-yl)-2-((((*R*)-(benzyloxy)(mercapto)phosphoryl)oxy)methyl)tetrahydrofuran-3-yl 3-((9*S*,12*S*,15*S*)-9-((tert-butoxycarbonyl)amino)-15-isobutyl-12-isopropyl-3,10,13-trioxo-1-phenyl-2-oxa-4,11,14-triazahexadecan-16-amido)benzoate triethylammonium (3ah)

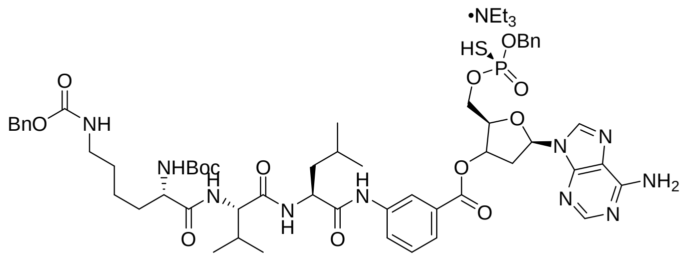

From **1a** and **2h** under condition A at 0.05 mmol scale. The chemoselectivity was determined by ^{1}H NMR analysis of the crude sample (**3ah**:**4ah** = 93:7). The reaction mixture was purified by reversed-phase column chromatography (Biotage®, SNAP Ultra C18 25 g; gradient 0% MeOH (2% NEt_3)/H_2O over 2 CV, 0%-100% MeOH (2% NEt_3)/H_2O over 15 CV.) The acylation product **3ah** was obtained as an amorphous solid (42% yield, average of two runs: 26.6 mg, 43%; 26.0 mg, 42%). **^{1}H NMR** (400 MHz, Chloroform-*d*) δ 8.74 (s, 1H), 8.26 (t, *J* = 2.0 Hz, 1H), 8.21 (s, 1H), 8.02 (d, *J* = 8.4 Hz, 1H), 7.83 (dt, *J* = 7.6, 1.4 Hz, 1H), 7.46 (t, *J* = 8.0 Hz, 1H), 7.41 – 7.11 (m, 8H), 6.62 (dd, *J* = 9.2, 5.6 Hz, 1H), 5.76 (d, *J* = 5.6 Hz, 1H), 5.07 – 4.93 (m, 3H), 4.56 (dd, *J* = 9.6, 4.8 Hz, 1H), 4.45 (s, 1H), 4.29 (ddd, *J* = 11.2, 6.8, 2.4 Hz, 1H), 4.21 (d, *J* = 6.8 Hz, 1H), 4.20 – 4.05 (m, 2H), 3.09 (t, *J* = 6.4 Hz, 1H), 3.02 (q, *J* = 7.2 Hz, 7H), 2.71 (dd, *J* = 14.0, 5.6 Hz, 1H), 2.13 (dt, *J* = 22.0, 7.6 Hz, 1H), 1.81 – 1.58 (m, 5H), 1.43 (s, 9H), 1.23 (t, *J* = 7.2 Hz, 9H), 1.06 – 0.86 (m, 12H). **^{13}C NMR** (101 MHz, Chloroform-*d*) δ 173.1, 166.9, 157.3, 153.9, 150.6, 141.2, 140.1, 139.5 (d, *J* = 8.5 Hz),131.7, 130.2, 129.4, 129.2, 128.9, 128.8, 128.6, 128.5, 126.3, 126.0, 122.3, 120.0, 85.7 (d, *J* = 9.1 Hz), 85.5, 78.4, 68.9 (d, *J* = 5.2 Hz), 67.3, 66.6 (d, *J* = 6.4 Hz), 60.4, 54.0, 47.6, 41.7, 41.4, 39.3, 32.0, 30.5, 28.7, 26.0, 24.1, 23.6, 21.9, 19.7, 9.7. **^{31}P NMR** (162 MHz, Methanol-*d*$_4$) δ 57.9. **IR** (neat, ν/cm^{-1}) = 3306, 2968, 1640, 1601, 1454, 1368, 1242, 1160, 1103, 1058, 935, 800, 736, 697; **HRMS** (ESI, m/z): [M + H]$^+$ calcd for $C_{54}H_{71}N_{10}O_{13}PS$ = 1131.4738; found = 1131.4805.

benzyl tert-butyl ((5S)-6-(((2S)-1-(((2S)-1-((3-((9-((2R,5R)-5-((((R)-(benzyloxy)(mercapto)phosphoryl)oxy)methyl)-4-hydroxytetrahydrofuran-2-yl)-9H-purin-6-yl)carbamoyl)phenyl)amino)-4-methyl-1-oxopentan-2-yl)amino)-3-methyl-1-oxobutan-2-yl)amino)-6-oxohexane-1,5-diyl)dicarbamate triethylammonium (4ah)

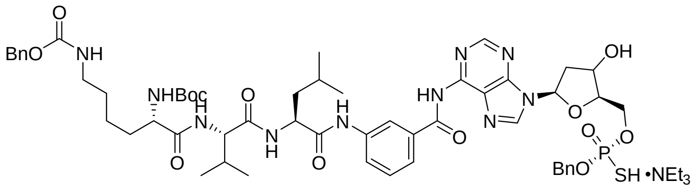

From **1a** and **2h** under condition B at 0.05 mmol scale. The chemoselectivity was determined by ^{1}H NMR analysis of the crude sample (**3ah**:**4ah** = 12:88). The reaction mixture was purified by reversed-phase column chromatography (Biotage®, SNAP Ultra C18 25 g; gradient 0% MeCN (2% NEt_3)/H_2O over 2 CV, 0%-100% MeCN (2% NEt_3)/H_2O over 15 CV.) The acylation product **4ah** was obtained as an amorphous solid (32% yield, average of two runs: 19.4 mg, 31%; 20.8 mg, 34%). **^{1}H NMR** (400 MHz, Methanol-*d*$_4$) δ 11.46 (s, 1H), 9.04 (s, 1H), 8.72 (s, 1H), 8.58 (s, 1H), 8.35 (s, 1H), 8.23 (d, *J* = 8.4 Hz, 1H), 7.71 (d, *J* = 7.8 Hz, 1H), 7.53 – 7.14 (m, 10H), 6.74 (s, 1H), 6.52 (t, J = 6.1 Hz, 1H), 6.21 – 6.09 (m, 1H), 5.59 (s, 1H), 5.23 – 4.86 (m, 4H), 4.69 (s, 2H), 4.33 – 4.01 (m, 4H), 3.94 (s, 1H), 3.06 (qd, *J* = 7.2, 4.4 Hz, 8H), 2.74 – 2.61 (m, 1H), 2.52 (dt, J = 13.2, 6.0 Hz, 1H), 2.42 – 2.31 (m, 1H), 2.14 – 1.53 (m, 6H), 1.51 – 1.15 (m, 18H), 1.03 – 0.89 (m, 12H). **^{13}C NMR** (101 MHz, Methanol-*d*$_4$) δ 174.4, 171.4, 171.2, 166.8, 157.7, 157.3, 152.5, 151.6, 149.5, 144.6, 142.1, 139.2, 136.6, 134.1, 129.5, 128.7, 128.4, 128.3, 128.1, 127.9, 127.8, 124.4, 123.8, 119.4, 84.1, 81.2, 68.3, 66.7, 65.5, 60.4, 56.8, 52.7, 45.9, 40.0, 39.4, 37.8, 30.2, 29.8, 29.6, 29.1, 28.3, 25.0, 23.5, 22.3, 20.8, 19.6, 17.6, 8.7. **^{31}P NMR** (162 MHz, Methanol-*d*$_4$) δ 58.9. **IR** (neat, ν/cm^{-1}) = 696, 731, 798,936, 998,1018, 1185, 1159, 1263,1454, 1517,1645, 1700, 2923, 3286; **HRMS** (ESI, m/z): [M - H]$^{-}$ calcd for $C_{54}H_{71}N_{10}O_{13}PS$ = 1129.4582; found = 1129.4683.

4. Competition experiments

Figure S3. Competition reaction of amines and alcohols.

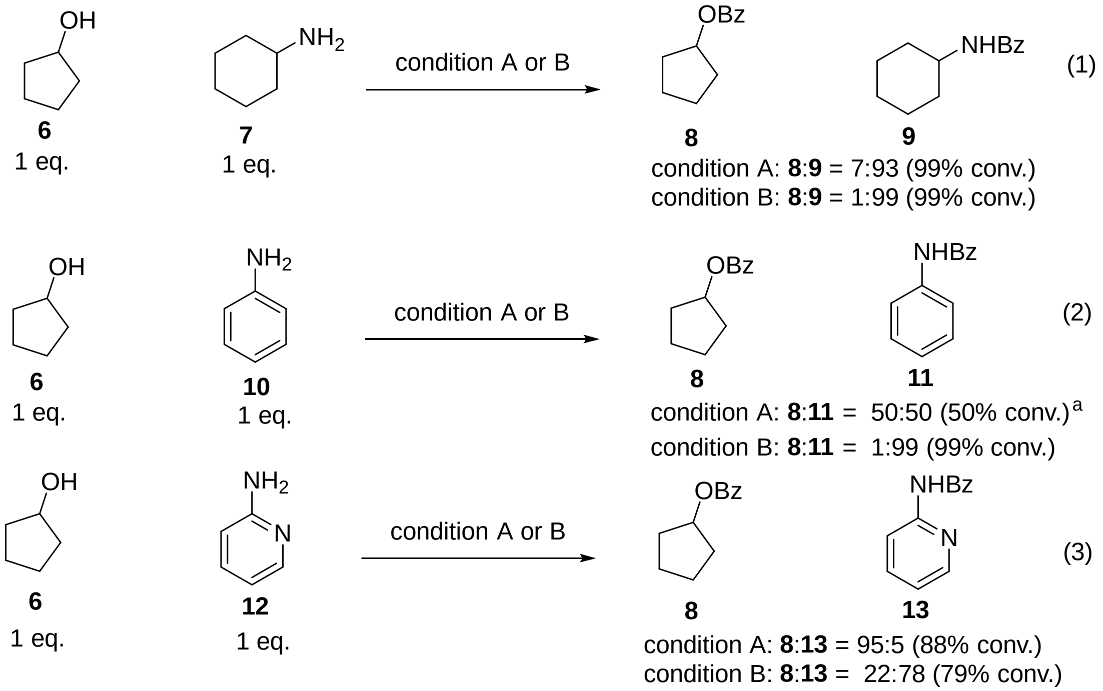

Equation 1, Condition A:

6 (4.5 µL, 0.05 mmol, 1.0 eq.), **7** (5.7 µL, 0.05 mmol, 1.0 eq.), and Bz_2O (11.3 mg, 0.05 mmol, 1.0 eq.) were dissolved in $CDCl_3$ (0.25 mL, 0.2 M) in a 2 mL vial equipped with a stir bar, followed by addition of DMAP (10.0 µL (1 M in $CHCl_3$), 0.01 mmol, 20 mol%) and NEt_3 (10.5 µL, 0.15 mmol, 1.5 eq.). The reaction was stirred at rt for 16 h. The chemoselectivity of the reaction was determined by ^{1}H NMR analysis of the crude reaction mixture (**8**:**9** = 7:93, 99% conversion).

Equation 1, Condition B:

6 (4.5 µL, 0.05 mmol, 1.0 eq.), **7** (5.7 µL, 0.05 mmol, 1.0 eq.), and Bz_2O (11.3 mg, 0.05 mmol, 1.0 eq.) were dissolved in $CDCl_3$ (0.25 mL, 0.2 M) in a 2 mL vial equipped with a stir bar. The reaction was stirred at 50 ˚C for 16 h. The chemoselectivity of the reaction was determined by ^{1}H NMR analysis of the crude reaction mixture (**8**:**9** = 1:99, 99% conversion).

Equation 2, Condition A:

6 (4.5 µL, 0.05 mmol, 1.0 eq.),**10** (4.7 µL, 0.05 mmol, 1.0 eq.), and Bz_2O (11.3 mg, 0.05 mmol, 1.0 eq.) were dissolved in $CDCl_3$ (0.25 mL, 0.2 M) in a 2 mL vial equipped with a stir bar, followed by addition of DMAP (10.0 µL (1 M in $CHCl_3$), 0.01 mmol, 20 mol%) and NEt_3 (10.5 µL, 0.15 mmol, 1.5 eq.). The reaction was stirred at rt for 16 h. The chemoselectivity of the reaction was determined by ^{1}H NMR analysis of the crude reaction mixture (**8**:**11** = 50:50, 50% conversion according to **6**).

Equation 1, Condition B:

6 (4.5 μL, 0.05 mmol, 1.0 eq.), **10** (4.7 μL, 0.05 mmol, 1.0 eq.) and Bz_2O (11.3 mg, 0.05 mmol, 1.0 eq.) were dissolved in $CDCl_3$ (0.25 mL, 0.2 M) in a 2 mL vial equipped with a stir bar. The reaction was stirred at 50 ˚C for 16 h. The chemoselectivity of the reaction was determined by ^{1}H NMR analysis of the crude reaction mixture (**8**:**11** = 1:99, 99% conversion).

Equation 3, Condition A:
6 (4.5 μL, 0.05 mmol, 1.0 eq.),**12** (4.7 mg, 0.05 mmol, 1.0 eq.), and Bz_2O (11.3 mg, 0.05 mmol, 1.0 eq.) were dissolved in $CDCl_3$ (0.25 mL, 0.2 M) in a 2 mL vial equipped with a stir bar, followed by addition of DMAP (10.0 μL (1 M in $CHCl_3$) and 0.01 mmol, 20 mol%), NEt_3 (10.5 μL, 0.15 mmol, 1.5 eq.). The reaction was stirred at rt for 16 h. The chemoselectivity of the reaction was determined by ^{1}H NMR analysis of the crude reaction mixture (**8**:**13** = 95:5, 88% conversion)
Equation 1, Condition B:
6 (4.5 μL, 0.05 mmol, 1.0 eq.),**12** (4.7 mg, 0.05 mmol, 1.0 eq.), and Bz_2O (11.3 mg, 0.05 mmol, 1.0 eq.) were dissolved in $CDCl_3$ (0.25 mL, 0.2 M) in a 2 mL vial equipped with a stir bar. The reaction was stirred at 50 ˚C for 16 h. The chemoselectivity of the reaction was determined by ^{1}H NMR analysis of the crude reaction mixture (**8**:**13** = 22:78, 79% conversion).

Figure S4. Test of nucleosides under Heller's conditions[1]

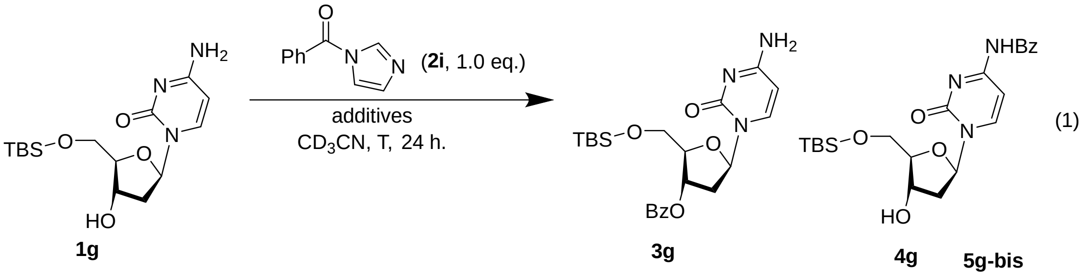

Pyridine-HCl (1.2 eq.), 50 °C: 57% conv., 3g:4g > 1:99

DBU (20 mol%), rt: 42% conv., 3g:4g > 99:1

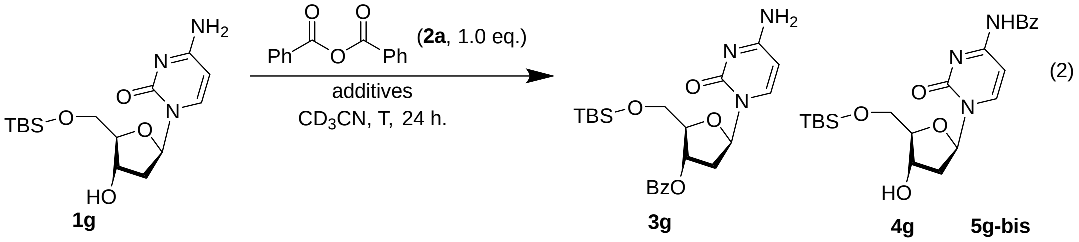

Pyridine-HCl (1.2 eq.), 50 °C: 67% conv., 3g:4g = 3:97

DBU (20 mol%), rt: 58% conv., 3g:4g > 1:99

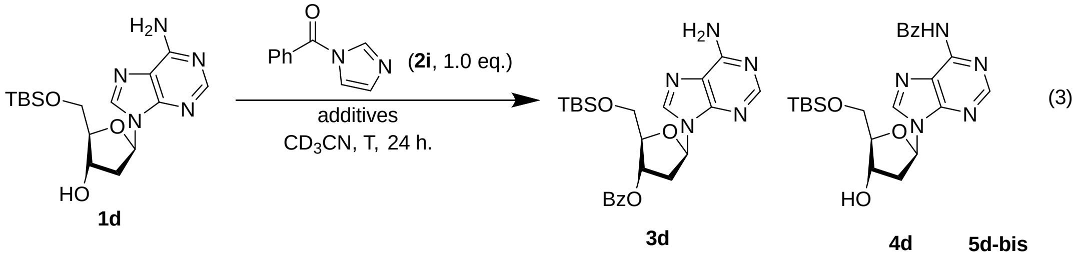

Pyridine-HCl (1.2 eq.), 50 °C: 0% Conv.

DBU (20 mol%), rt: 43% conv., 3d:4d > 99:1

Equation 1, entry 1:

1g (17.1 mg, 0.05 mmol, 1.0 eq.), **2i** (8.6 mg, 0.05 mmol, 1.0 eq.), and pyridinium chloride (6.9 mg, 0.06 mmol, 1.2 eq.) were dissolved in CD_3CN (0.25 mL, 0.2 M) in a 2 mL vial equipped with a stir bar. The reaction was stirred at 50 ℃ for 24 h. The chemoselectivity of the reaction was determined by ^{1}H NMR analysis of the crude reaction mixture (**1g**:**3g**:**4g**:**5g** = 43:0:57:0, **3g**:**4g** > 1:99).

Equation 1, entry 2:

1g (17.1 mg, 0.05 mmol, 1.0 eq.), **2i** (8.6 mg, 0.05 mmol, 1.0 eq.), and DBU (2.4 μL, 0.01 mmol, 20 mol%) were dissolved in CD_3CN (0.25 mL, 0.2 M) in a 2 mL vial equipped with a stir bar. The reaction was stirred at rt for 24 h. The chemoselectivity of the reaction was determined by ^{1}H NMR analysis of the crude reaction mixture (**1g**:**3g**:**4g**:**5g** = 58:42:0:0, **3g**:**4g** > 99:1).

Equation 2, entry 1:

1g (17.1 mg, 0.05 mmol, 1.0 eq.), Bz_2O (11.3 mg, 0.05 mmol, 1.0 eq.), and pyridinium chloride (6.9 mg, 0.06 mmol, 1.2 eq.) were dissolved in CD_3CN (0.25 mL, 0.2 M) in a 2 mL vial equipped with a stir bar. The reaction was stirred at 50 ̊C for 24 h. The chemoselectivity of the reaction was determined by ^1H NMR analysis of the crude reaction mixture (**1g**:**3g**:**4g**:**5g** = 33:2:65:0, **3g**:**4g** = 3:97).

Equation 2, entry 2:

1g (17.1 mg, 0.05 mmol, 1.0 eq.), Bz_2O (11.3 mg, 0.05 mmol, 1.0 eq.), and DBU (2.4 μL, 0.01 mmol, 20 mol%) were dissolved in CD_3CN (0.25 mL, 0.2 M) in a 2 mL vial equipped with a stir bar. The reaction was stirred at rt for 24 h. The chemoselectivity of the reaction was determined by ^1H NMR analysis of the crude reaction mixture (**1g**:**3g**:**4g**:**5g** = 42:0:50:8, **3g**:**4g** = 1:99).

Equation 3, entry 1:

1d (18.3 mg, 0.05 mmol, 1.0 eq.), **2i** (8.6 mg, 0.05 mmol, 1.0 eq.), and pyridinium chloride (6.9 mg, 0.06 mmol, 1.2 eq.) were dissolved in CD_3CN (0.25 mL, 0.2 M) in a 2 mL vial equipped with a stir bar. The reaction was stirred at 50 ̊C for 24 h. The chemoselectivity of the reaction was determined by ^1H NMR analysis of the crude reaction mixture (**1d**:**3d**:**4d**:**5d** = 100:0:0:0).

Equation 3, entry 2:

1d (18.3 mg, 0.05 mmol, 1.0 eq.), **2i** (8.6 mg, 0.05 mmol, 1.0 eq.), and DBU (2.4 μL, 0.01 mmol, 20 mol%) were dissolved in CD_3CN (0.25 mL, 0.2 M) in a 2 mL vial equipped with a stir bar. The reaction was stirred at rt for 24 h. The chemoselectivity of the reaction was determined by ^1H NMR analysis of the crude reaction mixture (**1d**:**3d**:**4d**:**5d** = 57:43:0:0, **3d**:**4d** = 99:1).

4. Crystal information

Crystal preparation: **1b** (20.0 mg) was dissolved in a mixture of MeOH and water (1:1, 1.0 mL) in an uncapped 2 mL vial and then let stand still for two days.

Low-temperature diffraction data (ω-scans) were collected on a Rigaku MicroMax-007HF diffractometer coupled to a Saturn994+ CCD detector with Cu Kα (λ = 1.54178 Å) for the structure of 007a-21110. The diffraction images were processed and scaled using Rigaku Oxford Diffraction software (CrysAlisPro; Rigaku OD: The Woodlands, TX, 2015). The structure was solved with SHELXT and was refined against F^2 on all data by full-matrix least squares with SHELXL (Sheldrick, G. M. Acta Cryst. 2008, A64, 112–122). All non-hydrogen atoms were refined anisotropically. Hydrogen atoms were included in the model at geometrically calculated positions and refined using a riding model. The isotropic displacement parameters of all hydrogen atoms were fixed to 1.2 times the U value of the atoms to which they are linked (1.5 times for methyl groups). One of the two triethylamine models is disordered. The site occupancies of the two orientations were freely refined to converge values 0.66/0.34. The thermal parameters were constrained to be similar to each other. All disordered, chemically eq.alent C-C and C-N distances were restrained to be similar. The full numbering scheme of compound 007a-21110 can be found in the full details of the X-ray structure determination (CIF), which is included as Supporting Information. CCDC number 2158557 (007a-21110) contains the supplementary crystallographic data for this paper. These data can be obtained free of charge from The Cambridge Crystallographic Data Center via www.ccdc.cam.ac.uk/data_request/cif.

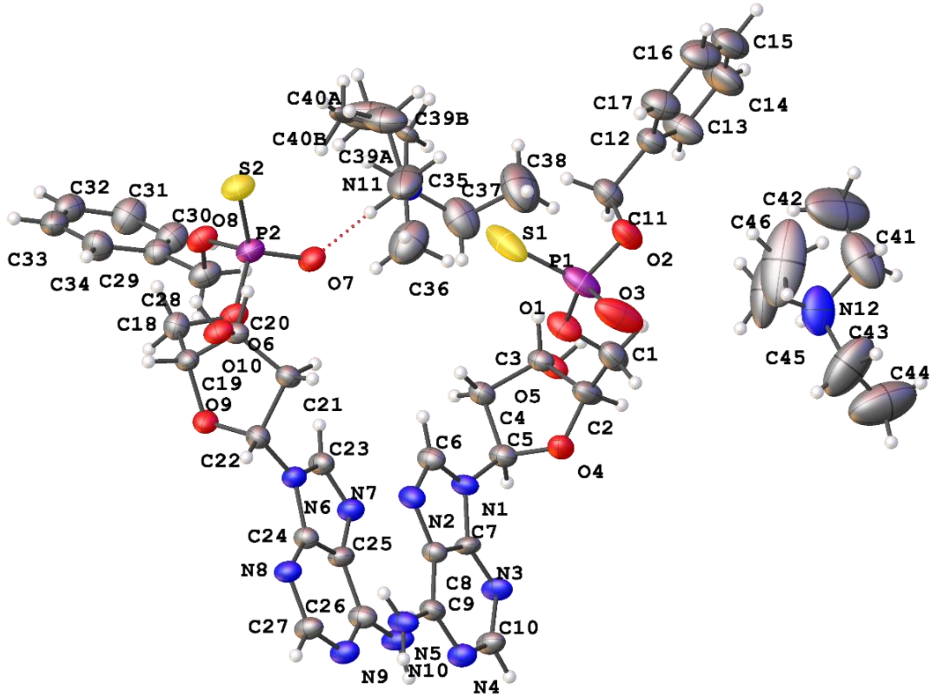

Figure S5. The complete numbering scheme of 007a-21110 with 50% thermal ellipsoid probability levels. The hydrogen atoms are shown as circles for clarity.

Table S1. Crystal data and structure refinement for 007a-21110.

Identification code	007a-21110	
Empirical formula	C23 H35 N6 O5 P S	
Formula weight	538.60	
Temperature	93(2) K	
Wavelength	1.54184 Å	
Crystal system	Monoclinic	
Space group	C2	
Unit cell dimensions	a = 34.2146(17) Å	a= 90°.
	b = 6.7156(2) Å	b= 125.753(8)°.
	c = 30.4667(16) Å	g = 90°.
Volume	5681.1(6) $Å^3$	
Z	8	
Density (calculated)	1.259 Mg/m^3	
Absorption coefficient	1.901 mm^{-1}	
F(000)	2288	
Crystal size	0.200 x 0.200 x 0.020 mm^3	
Crystal color and habit	Colorless Plate	
Diffractometer	Rigaku Saturn 944+ CCD	
Theta range for data collection	1.787 to 71.898°.	
Index ranges	-41<=h<=40, -8<=k<=7, -36<=l<=36	
Reflections collected	95075	
Independent reflections	10305 [R(int) = 0.1750]	
Observed reflections (I > 2sigma(I))	7654	
Completeness to theta = 67.684°	99.9 %	
Absorption correction	Semi-empirical from eq.alents	
Max. and min. transmission	1.00000 and 0.72406	
Solution method	SHELXT-2014/5 (Sheldrick, 2014)	
Refinement method	SHELXL-2014/7 (Sheldrick, 2014)	
Data / restraints / parameters	10305 / 46 / 675	
Goodness-of-fit on F^2	1.206	
Final R indices [I>2sigma(I)]	R1 = 0.0863, wR2 = 0.2417	
R indices (all data)	R1 = 0.1319, wR2 = 0.3124	
Absolute structure parameter	0.01(2)	
Largest diff. peak and hole	1.208 and -0.743 e.$Å^{-3}$	

5. NMR spectra

5.1 NMR spectra of substrates and products

_O_-(((2_R_,2_R_)-5-(6-amino-9_H_-purin-9-yl)-3-hydroxytetrahydrofuran-2-yl)methyl) _O_-benzyl (_S_)-phosphorothioate triethylammonium (1a)

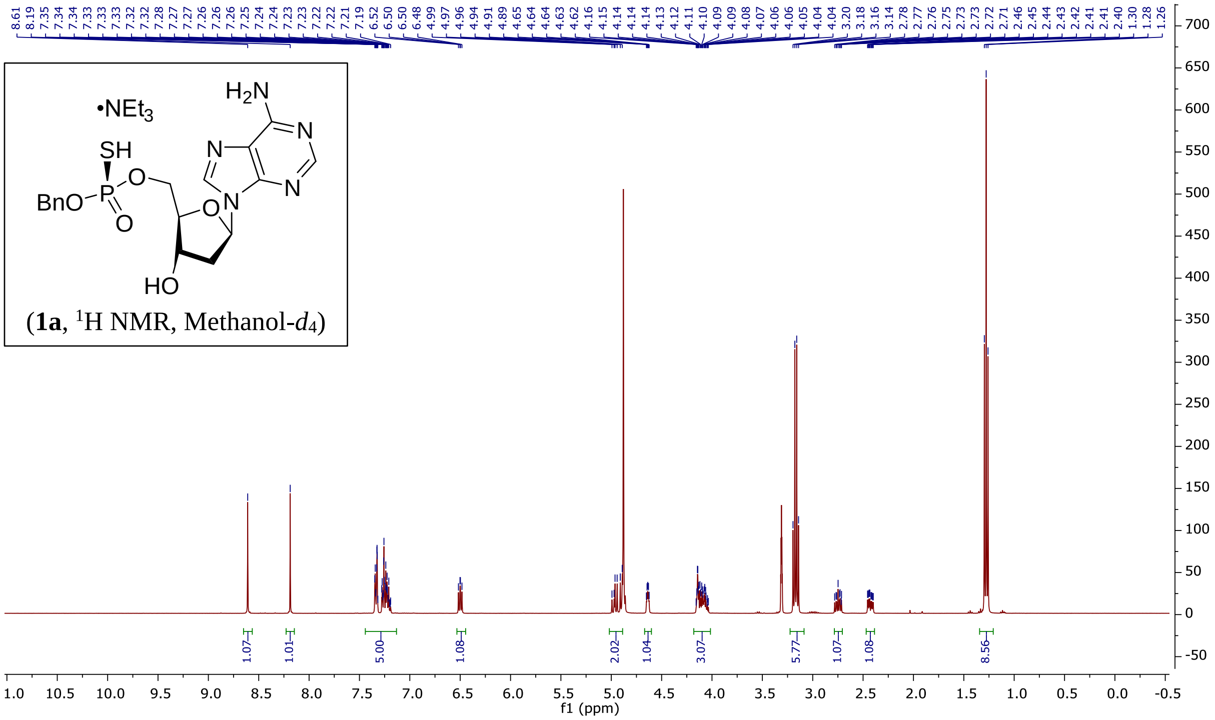

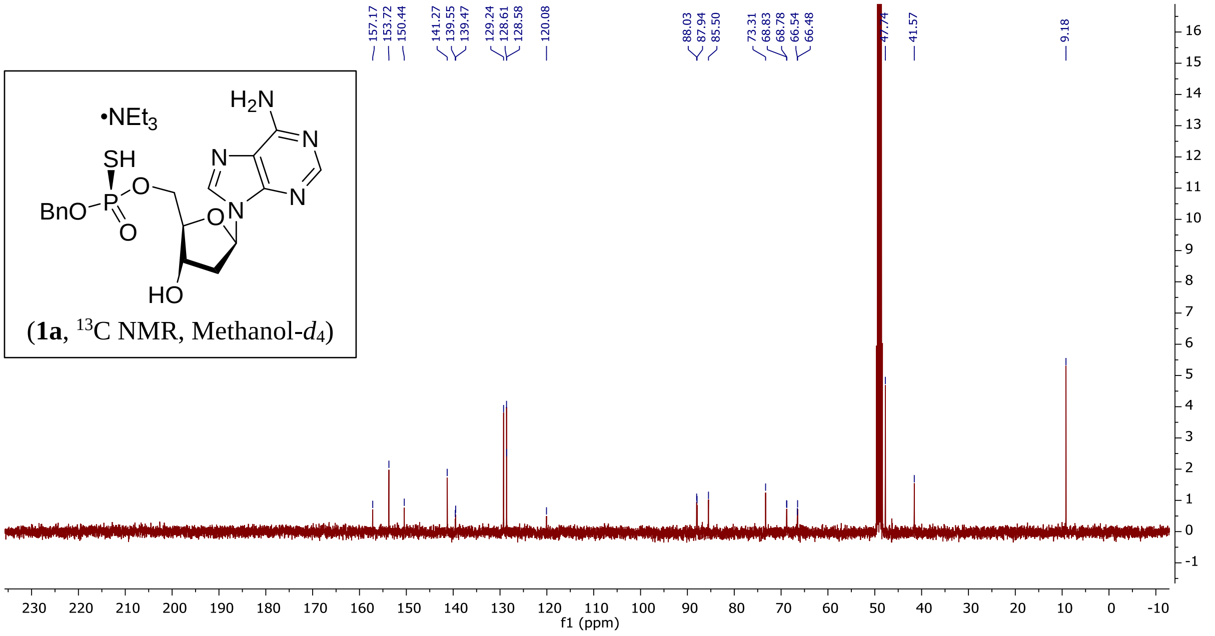

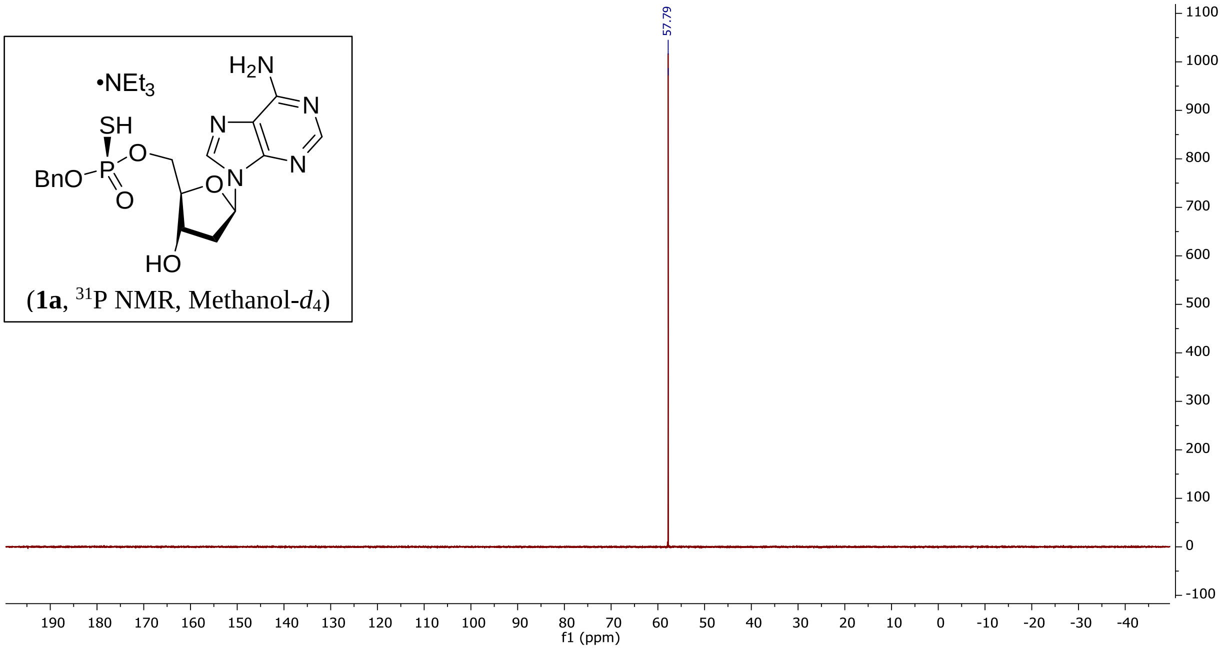

O-(((2*R*,2*R*)-5-(6-amino-9*H*-purin-9-yl)-3-hydroxytetrahydrofuran-2-yl)methyl) *O*-benzyl (*R*)-phosphorothioate triethylammonium (1b)

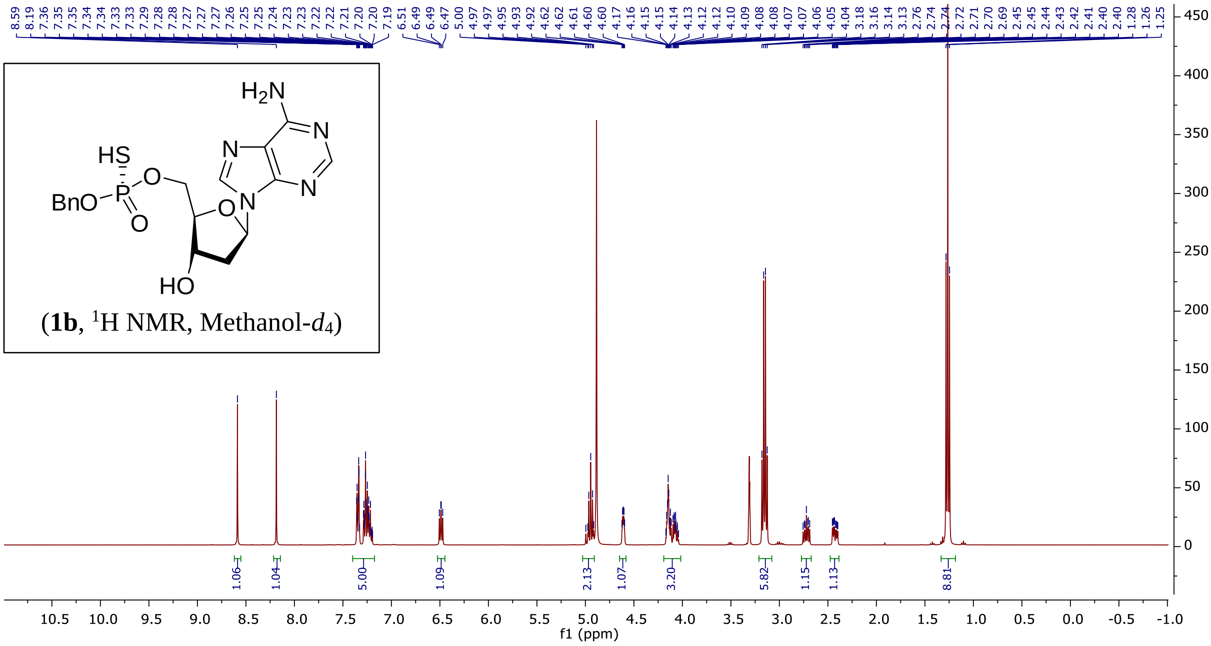

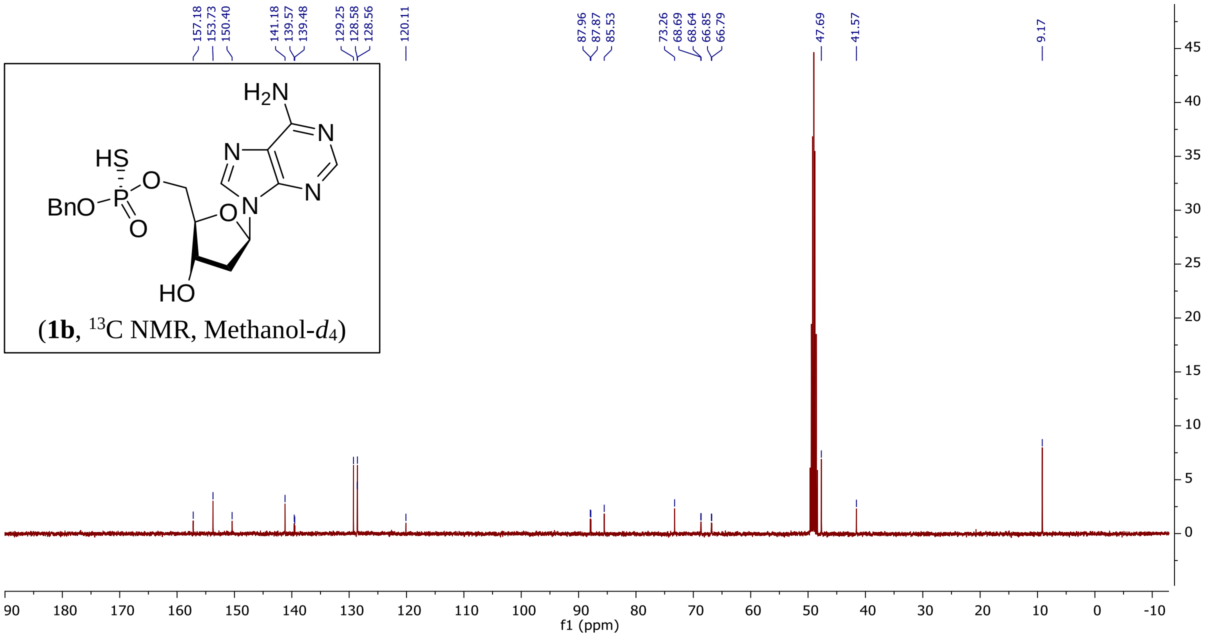
157.18
153.73
150.40
141.18
139.57
139.48
129.25
128.58
128.56
120.11
87.96
87.87
85.53
73.26
68.69
68.64
66.85
66.79
47.69
41.57
9.17
(1b, 13C NMR, Methanol-d4)
f1 (ppm)

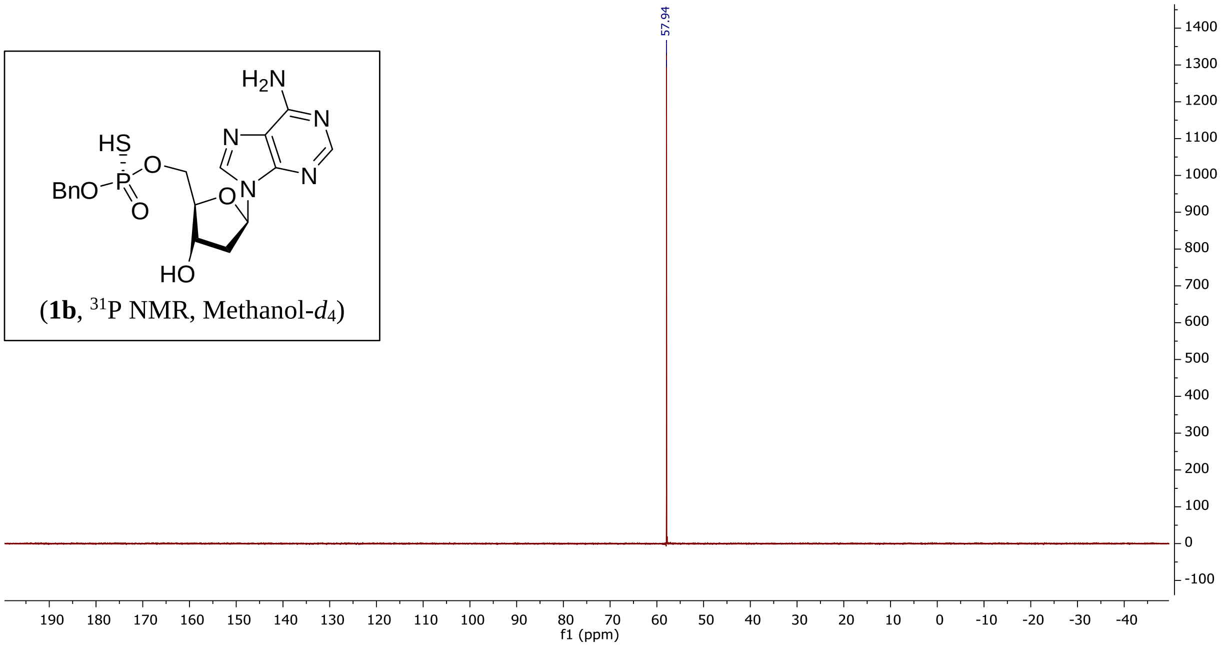
57.94
(1b, 31P NMR, Methanol-d4)
f1 (ppm)

((2*R*,2*R*)-5-(6-amino-9*H*-purin-9-yl)-3-hydroxytetrahydrofuran-2-yl)methyl benzyl phosphate triethylammonium (1c)

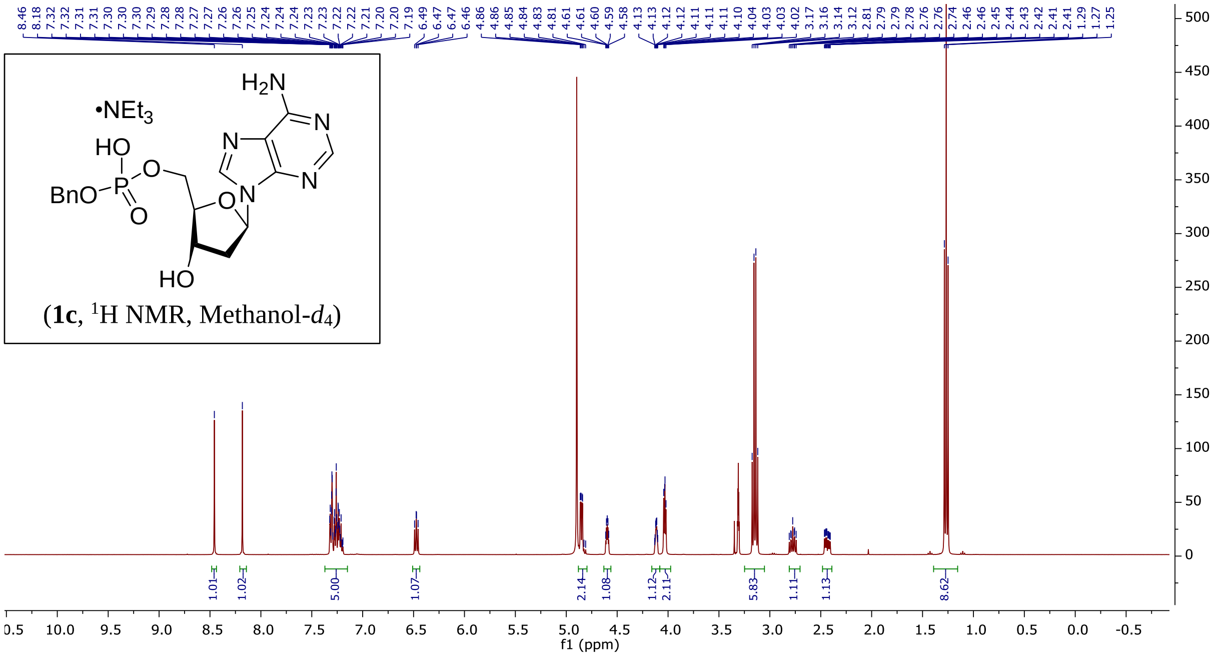

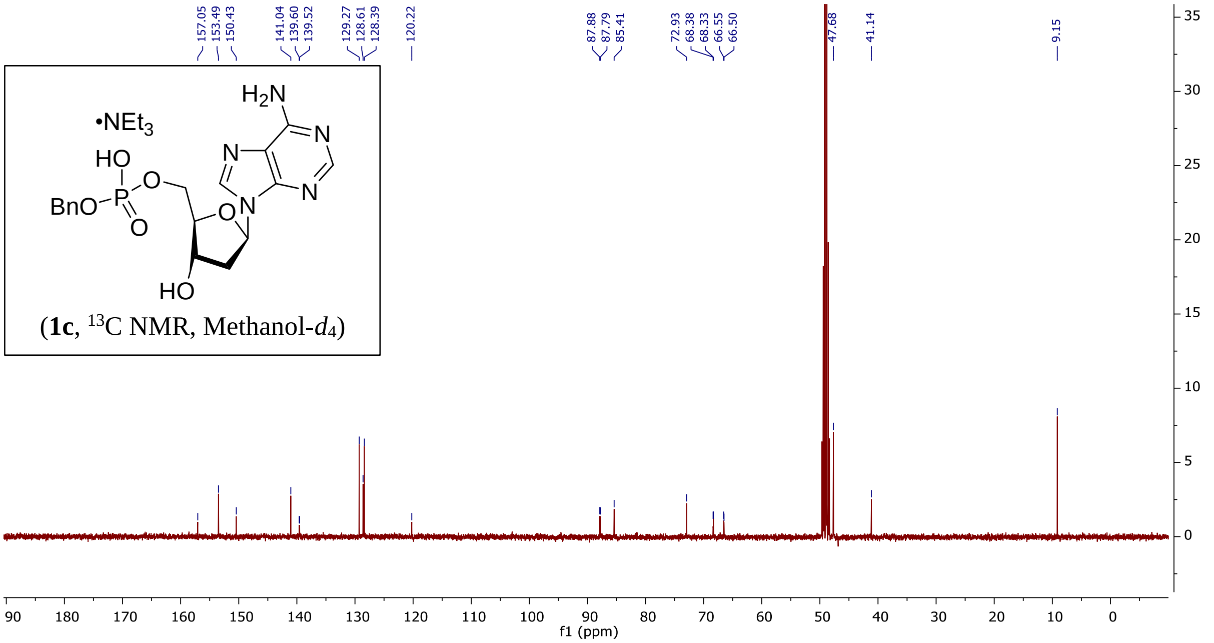

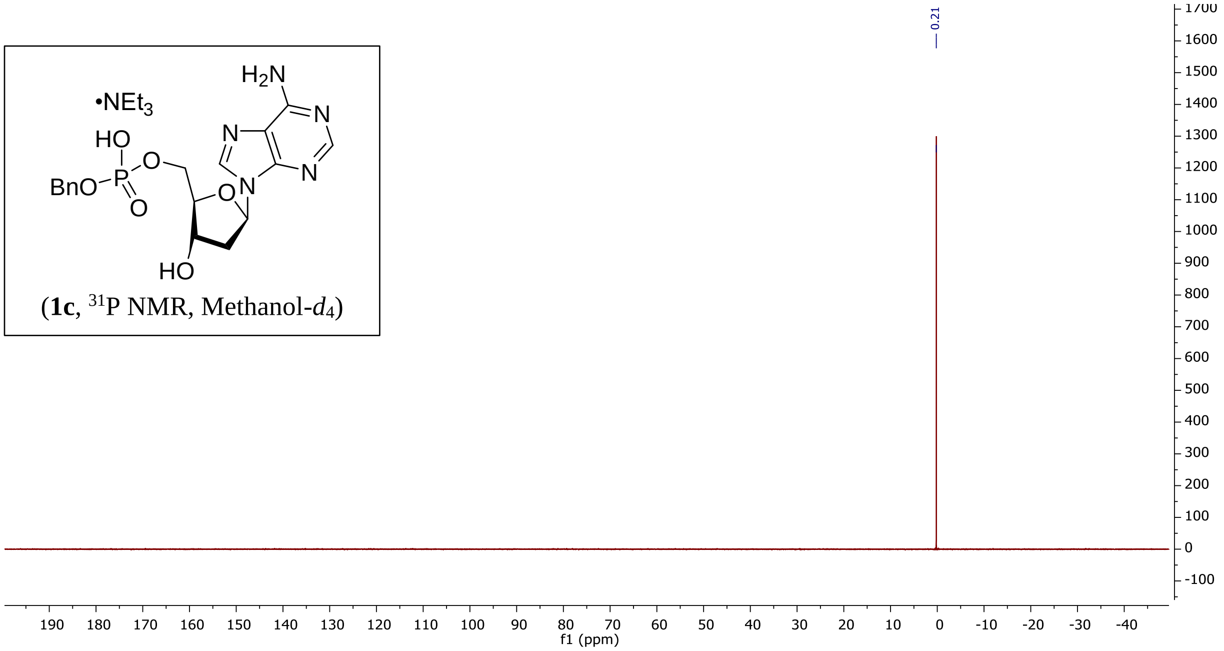

(2*R*,2*R*)-5-(6-amino-9*H*-purin-9-yl)-2-(((*tert*-butyldimethylsilyl)oxy)methyl)tetrahydrofuran-3-ol (1d)

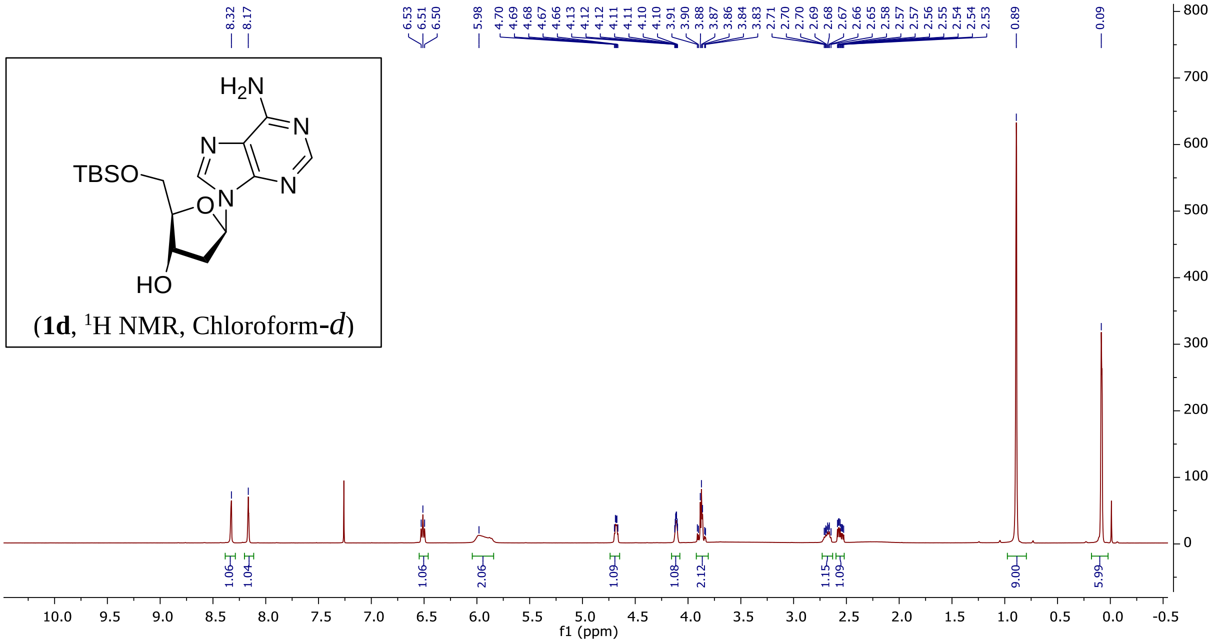

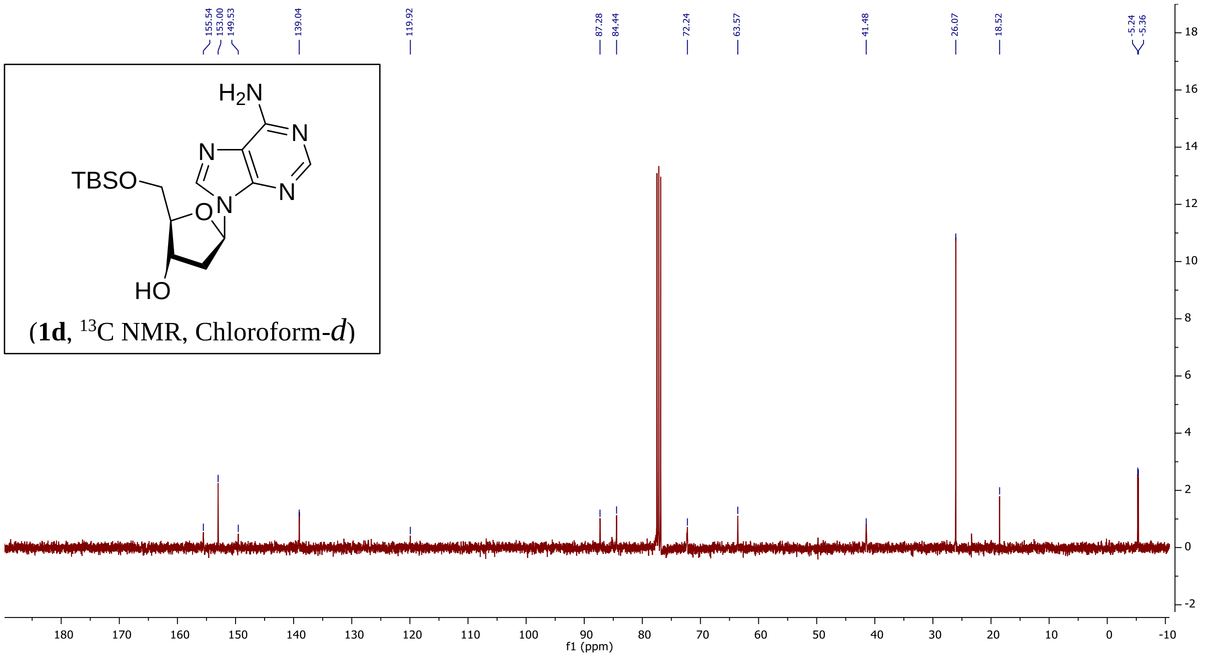

((3*aR*,6*aR*)-6-(6-amino-9*H*-purin-9-yl)-2,2-dimethyltetrahydrofuro[3,4-*d*][1,3]dioxol-4-yl)methanol (1e)

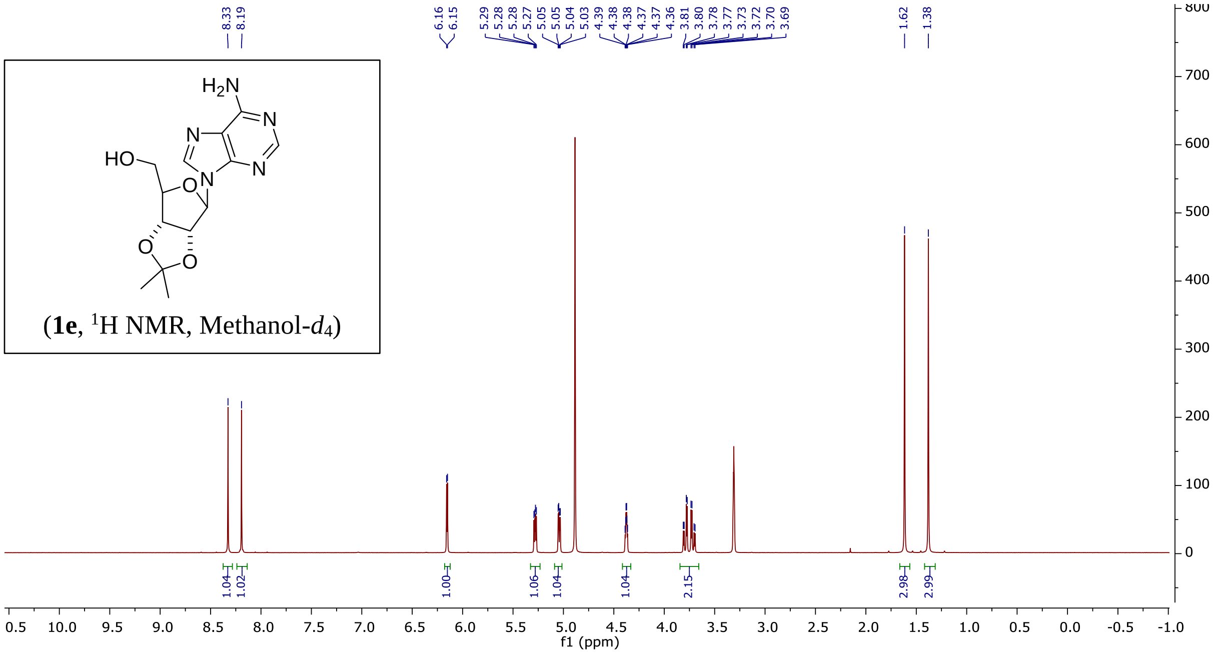

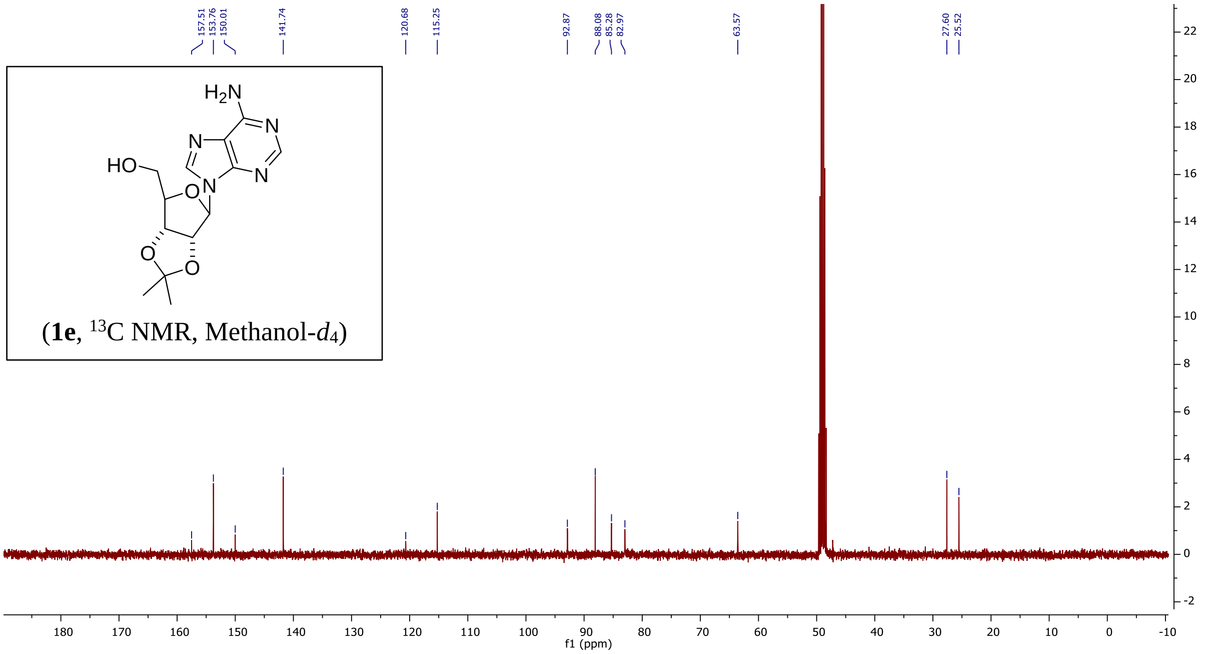

(9*R*,9*aS*)-8-(6-amino-9*H*-purin-9-yl)-2,2,4,4-tetraisopropyltetrahydro-6*H*-furo[3,2-*f*][1,3,5,2,4]trioxadisilocin-9-ol (1f)

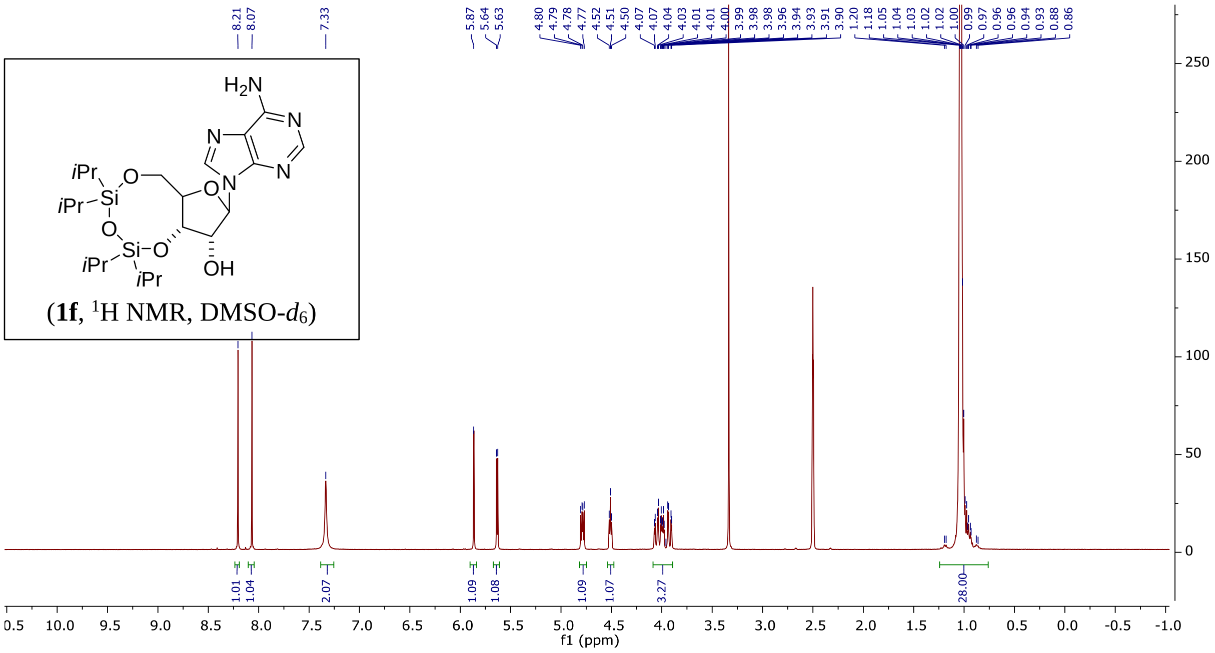

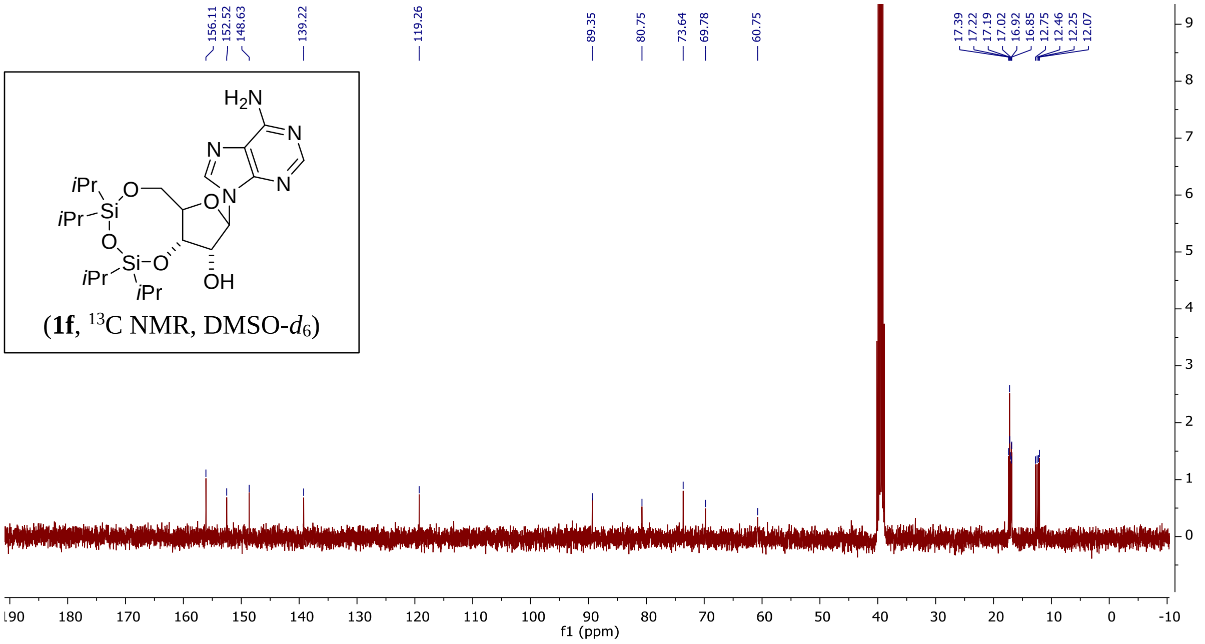

(**1f**, ^{13}C NMR, DMSO-d_6)

4-amino-1-((2*R*,2*R*)-5-(((*tert*-butyldimethylsilyl)oxy)methyl)-4-hydroxytetrahydrofuran-2-yl)pyrimidin-2(1*H*)-one (1g)

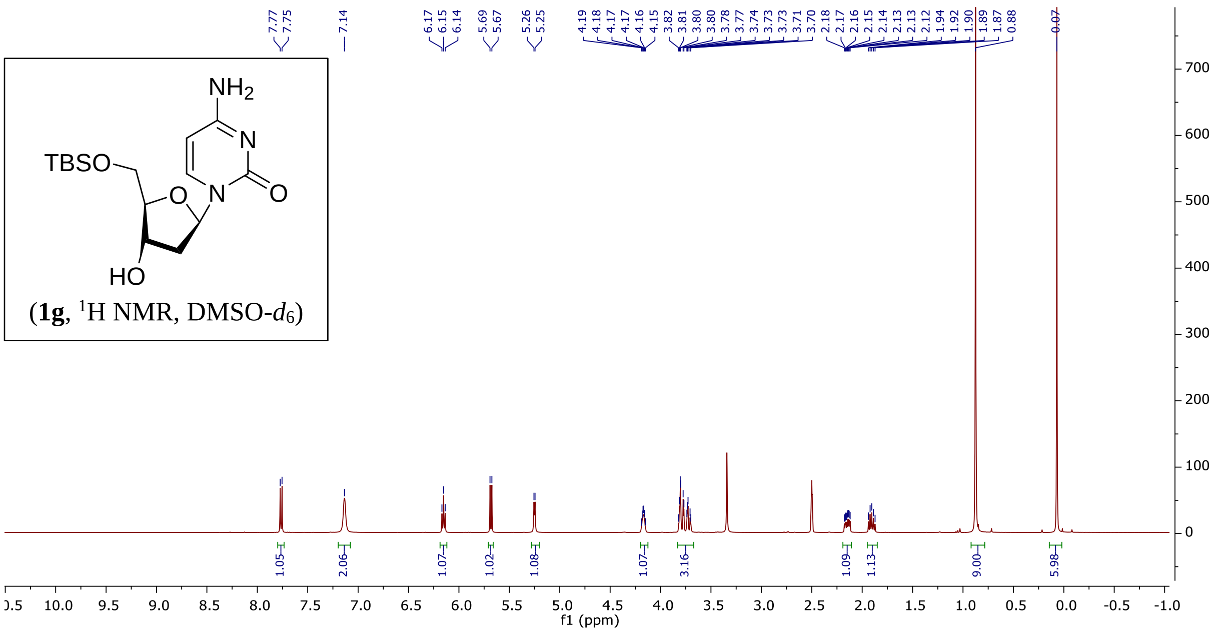

(**1g**, ^{1}H NMR, DMSO-d_6)

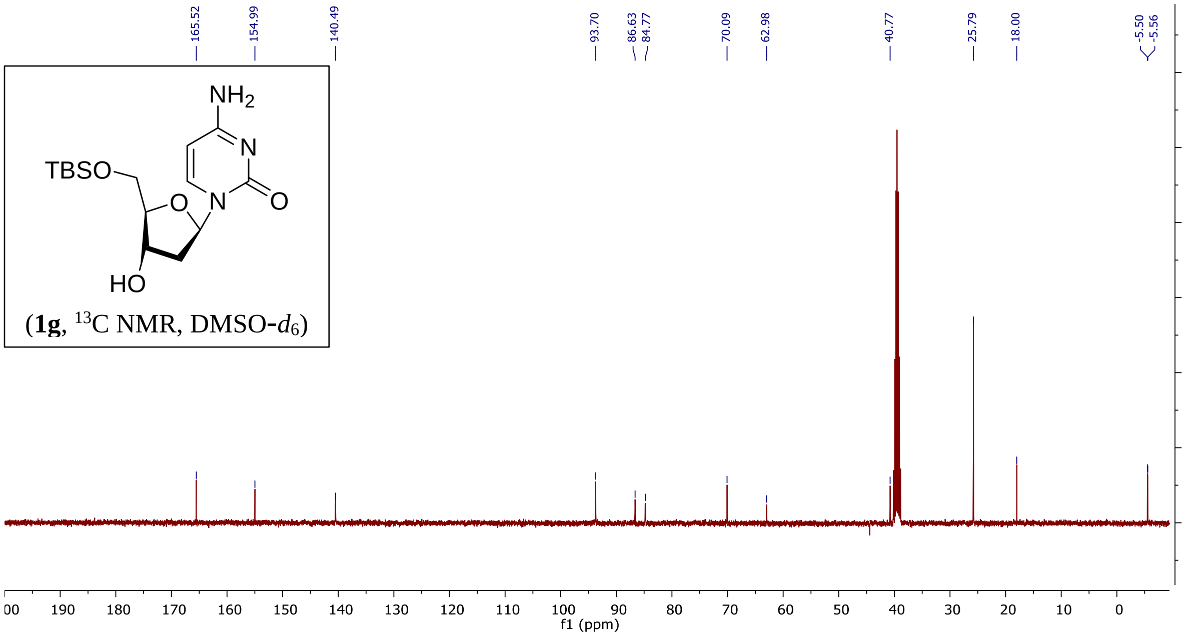

2-amino-9-((2*R*,2*R*)-5-(((*tert*-butyldimethylsilyl)oxy)methyl)-4-hydroxytetrahydrofuran-2-yl)-1,9-dihydro-6*H*-purin-6-one (1h)

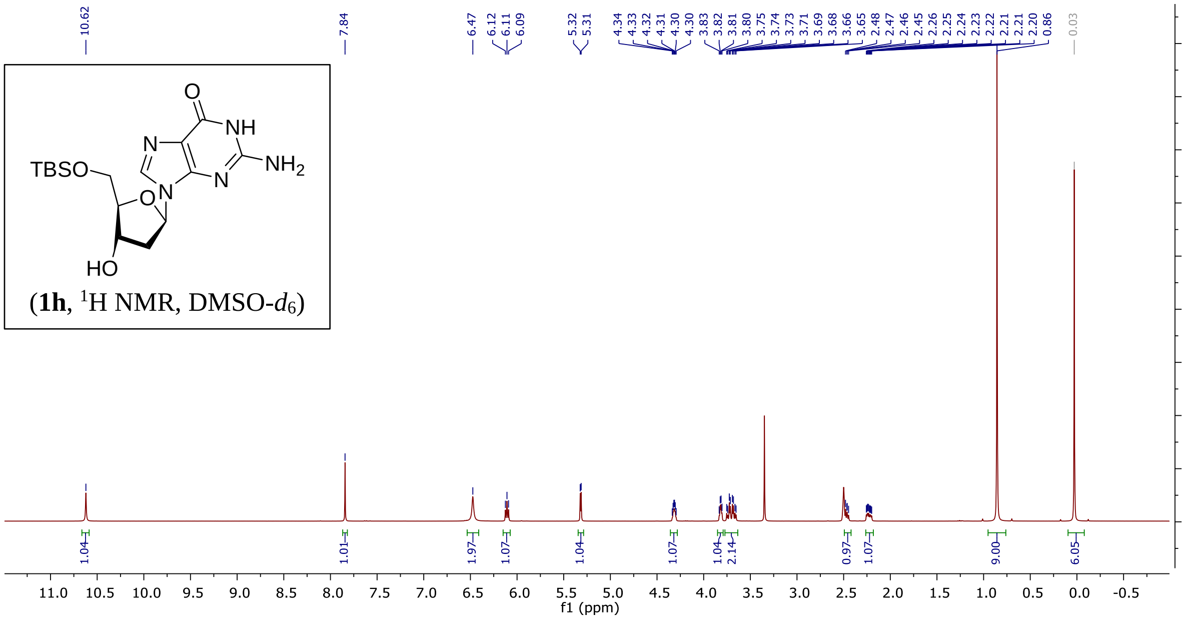

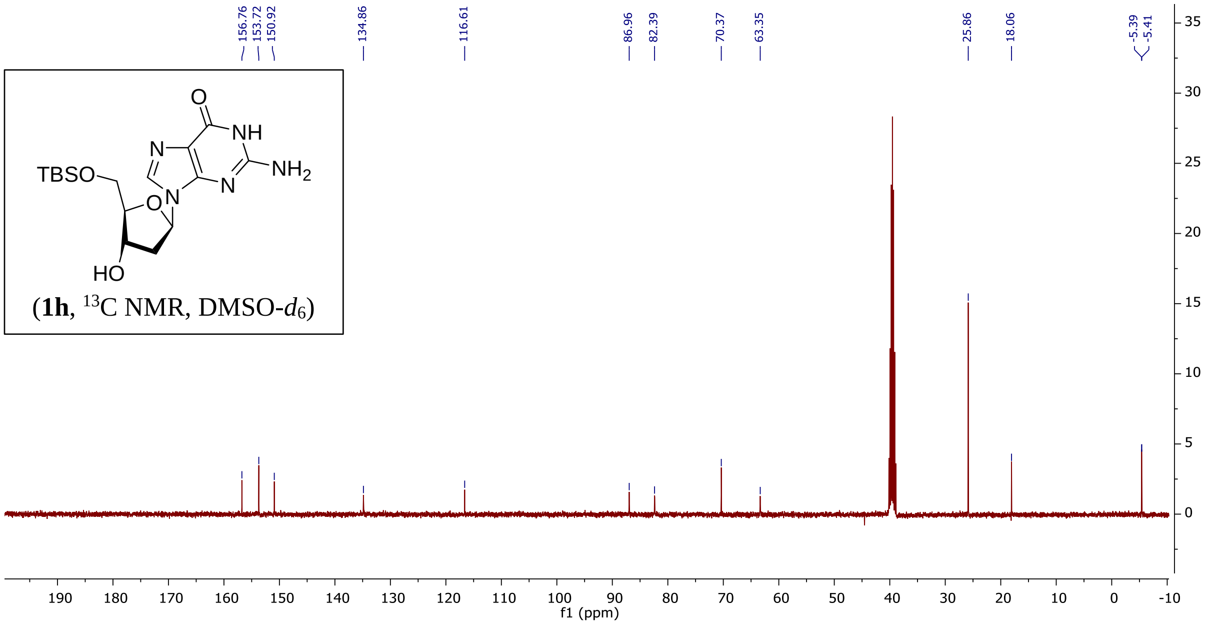

2,3,5,6-tetrafluoro-4-(trifluoromethyl)phenyl benzoate (2e)

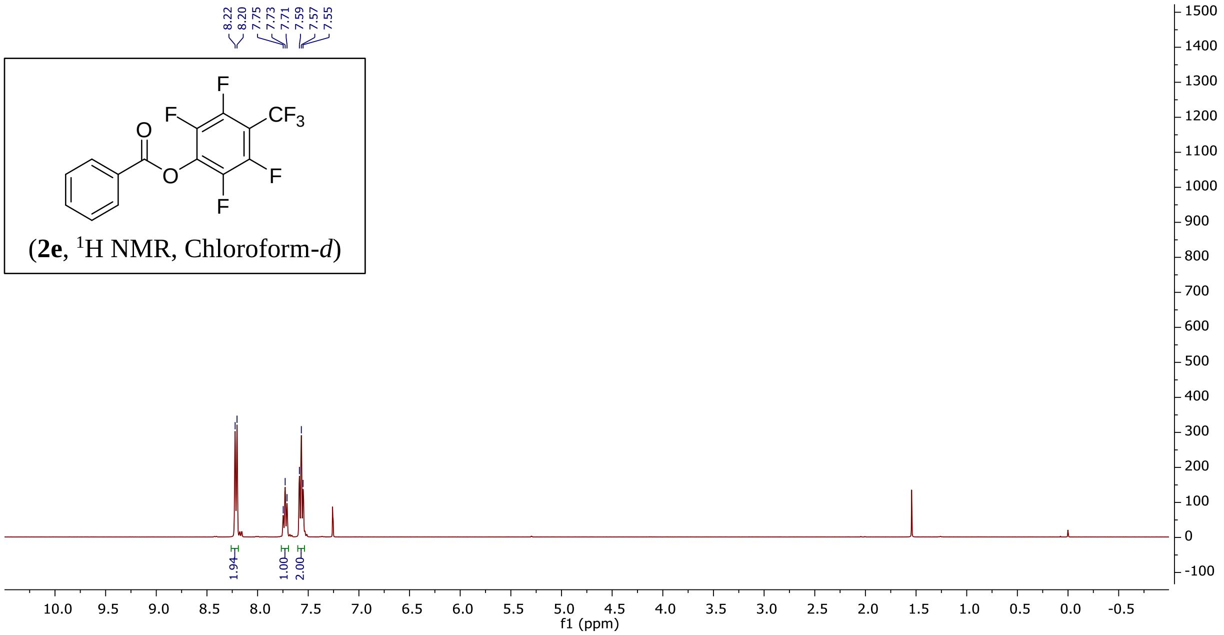

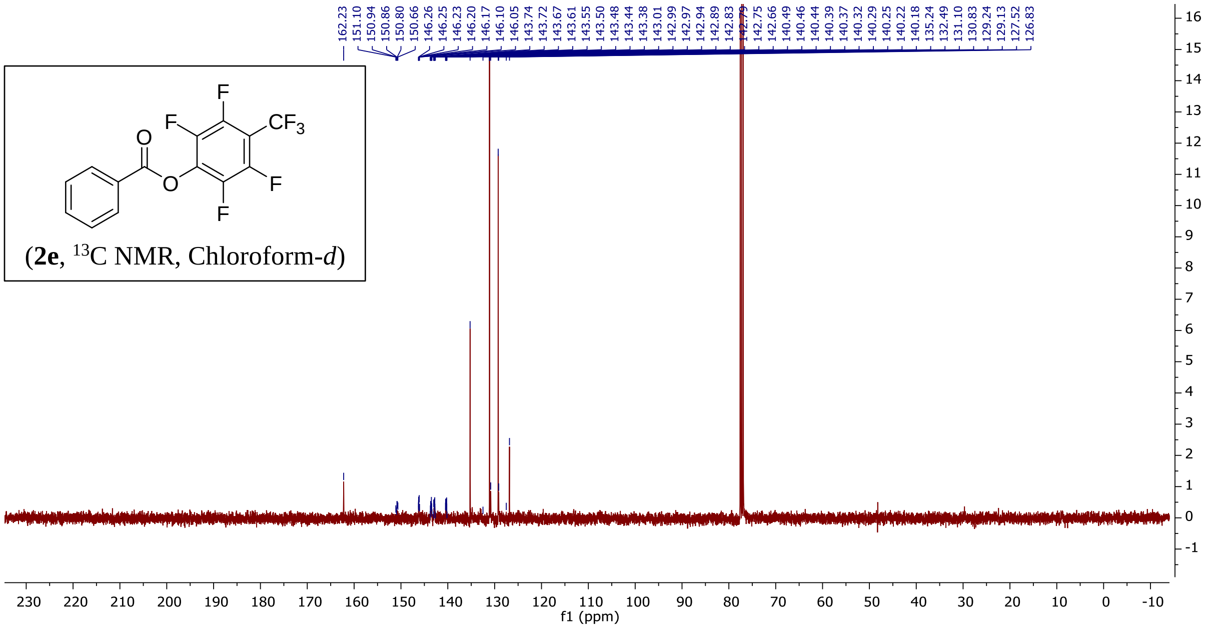

(2e, 13C NMR, Chloroform-d)
f1 (ppm)

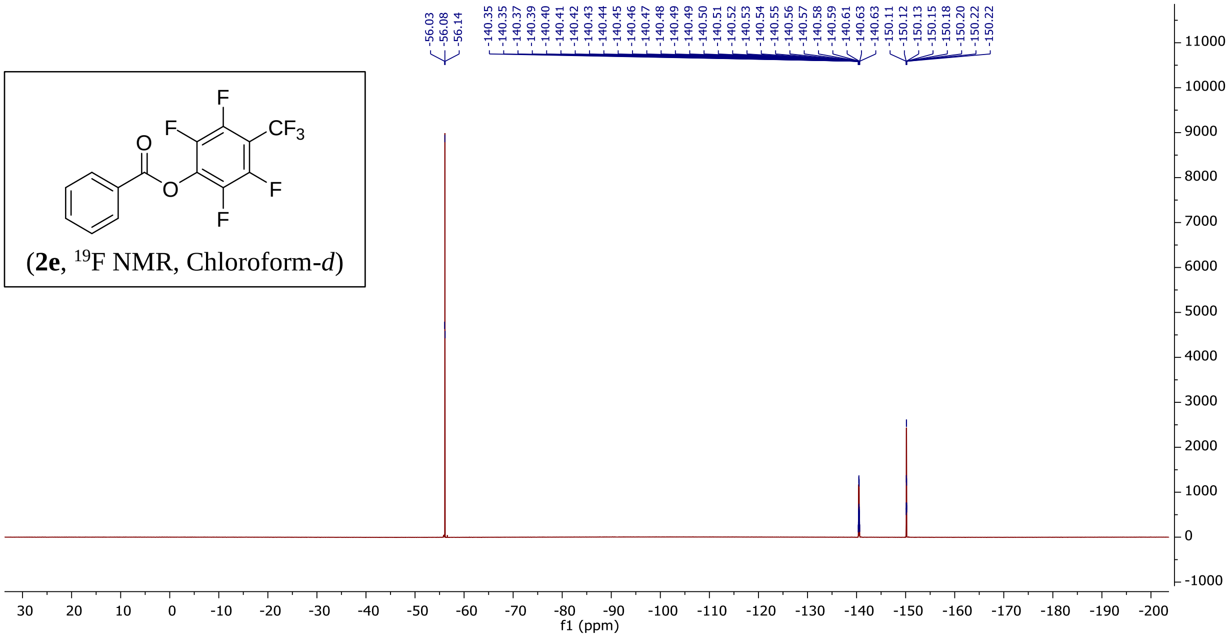

(2e, 19F NMR, Chloroform-d)
f1 (ppm)

1*H*-benzo[d][1,2,3]triazol-1-yl 1-((*tert*-butoxycarbonyl)amino)cyclopropane-1-carboxylate (2f)

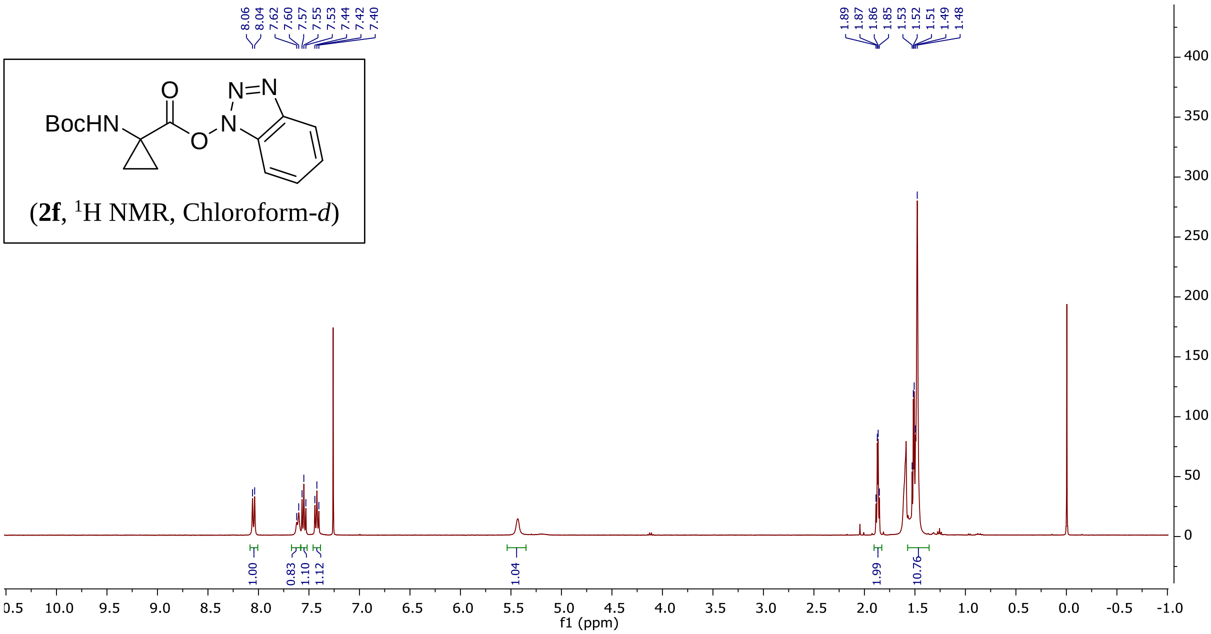

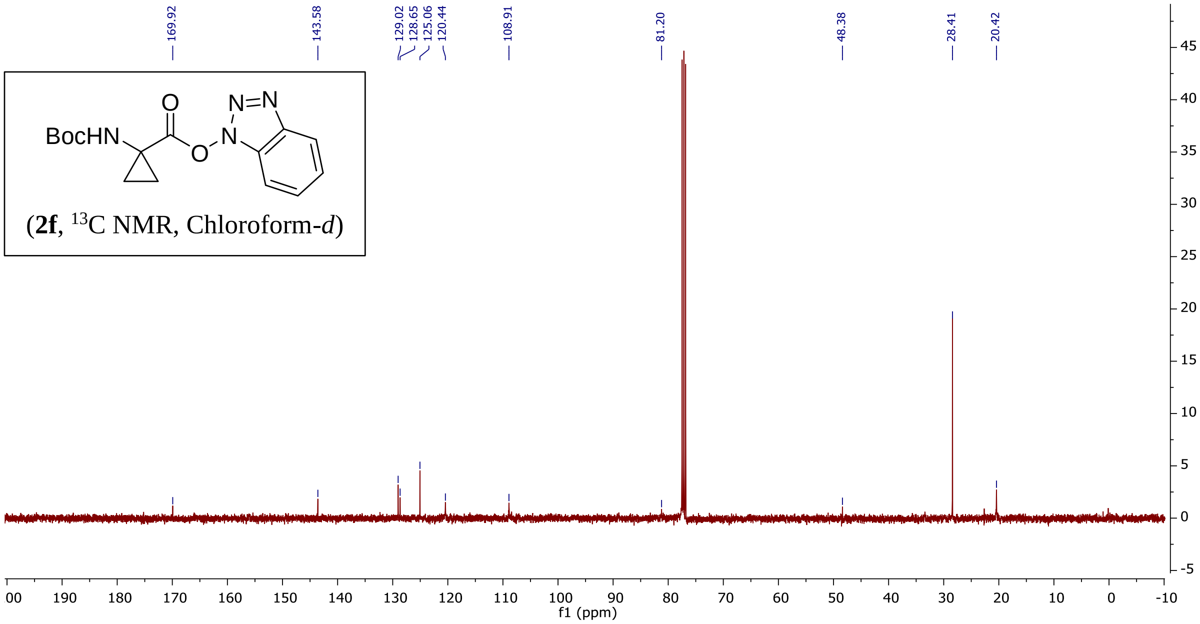

2,3,5,6-tetrafluoro-4-(trifluoromethyl)phenyl (*tert*-butoxycarbonyl)-*L*-phenylalaninate (2g)

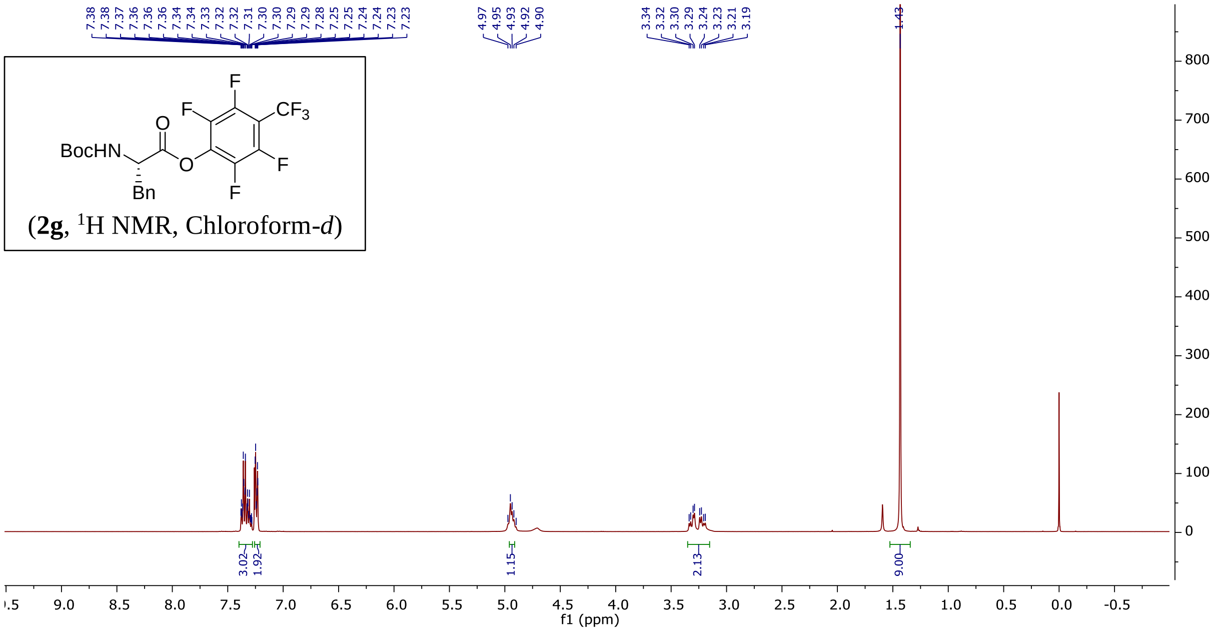

(**2g**, ^{1}H NMR, Chloroform-*d*)

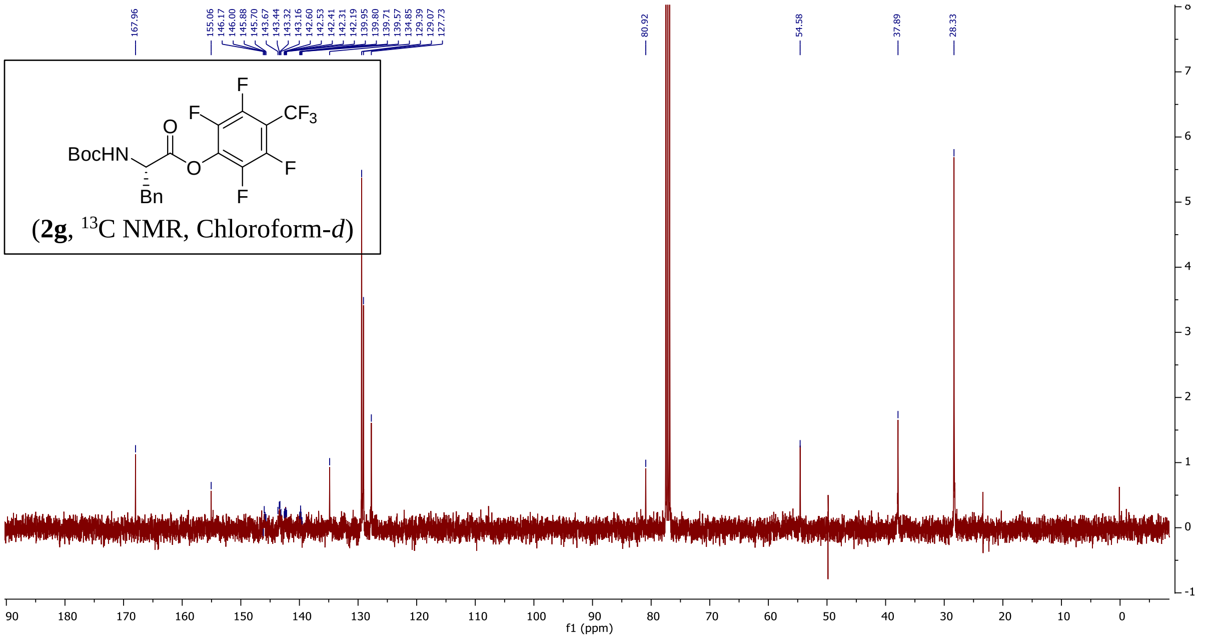

(**2g**, ^{13}C NMR, Chloroform-*d*)

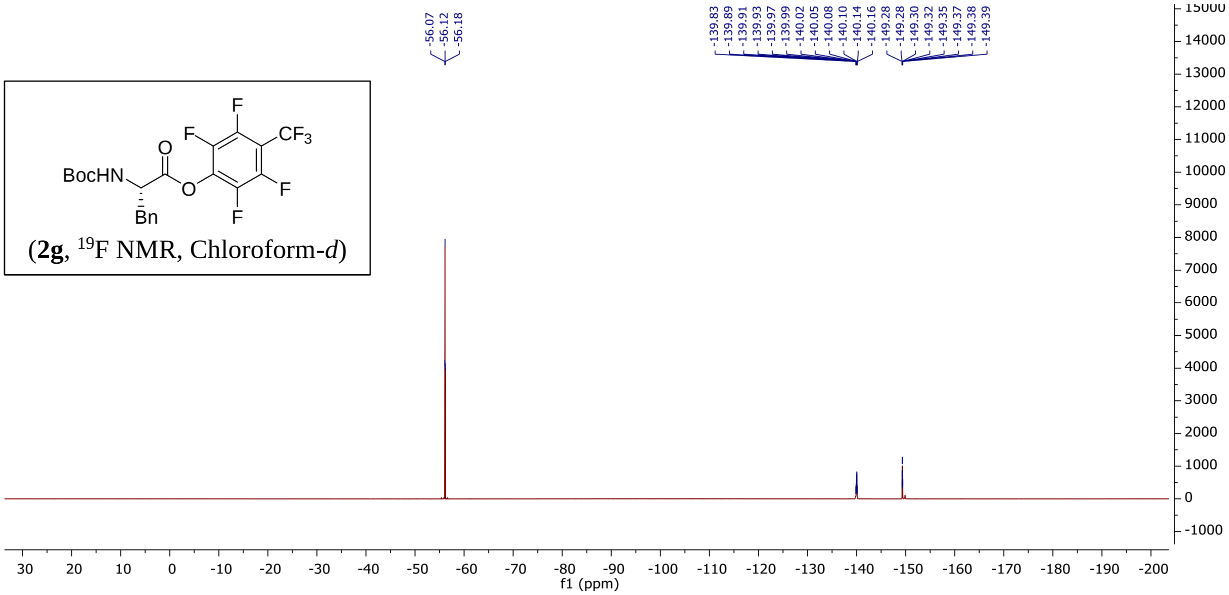

1H-benzo[d][1,2,3]triazol-1-yl 3-((9S,12S,15S)-9-((tert-butoxycarbonyl)amino)-15-isobutyl-12-isopropyl-3,10,13-trioxo-1-phenyl-2-oxa-4,11,14-triazahexadecan-16-amido)benzoate (2h)

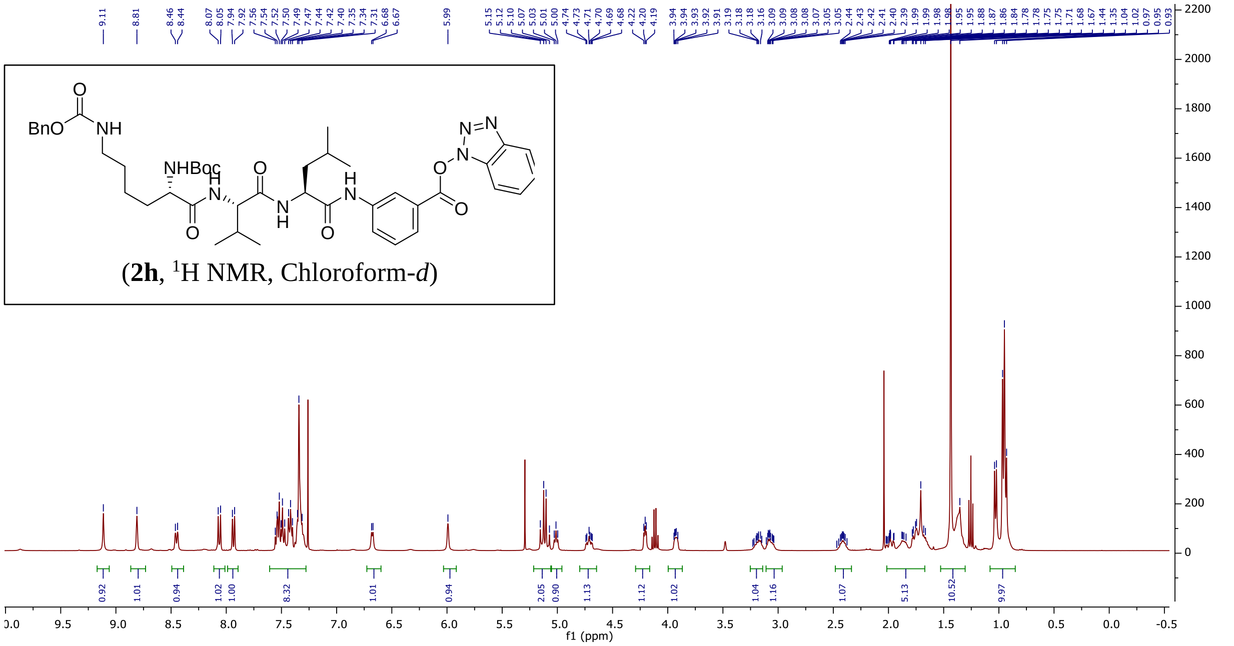

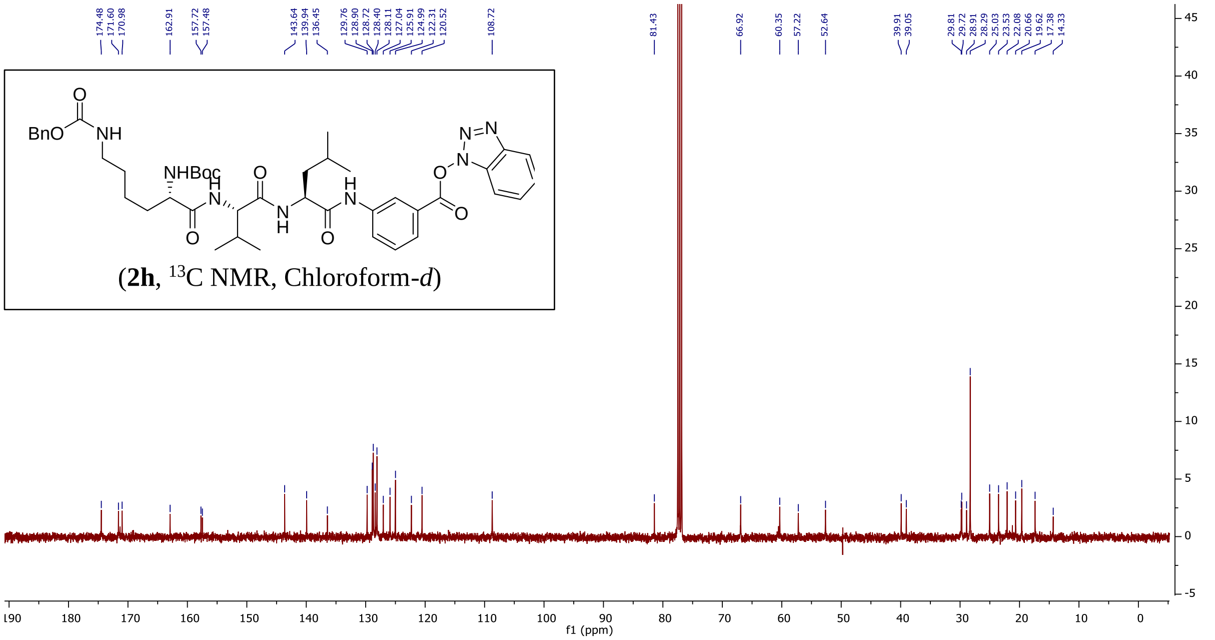

O-(((2R,2R)-5-(6-amino-9H-purin-9-yl)-3-(benzoyloxy)tetrahydrofuran-2-yl)methyl) O-benzyl (S)-phosphorothioate triethylammonium (3a)

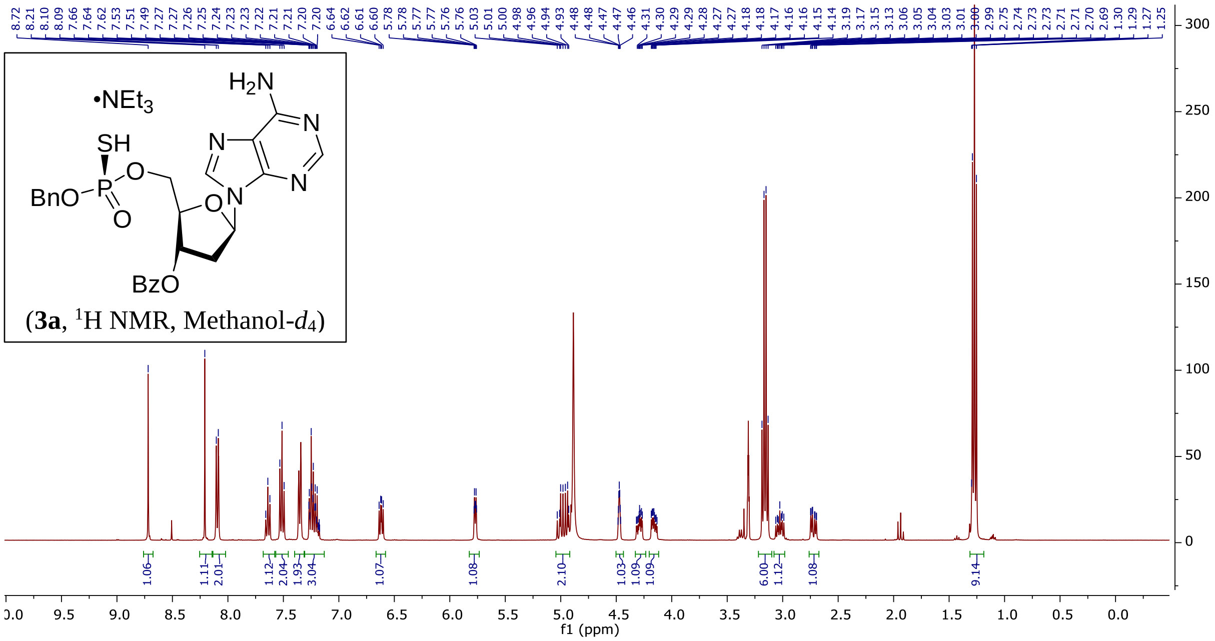

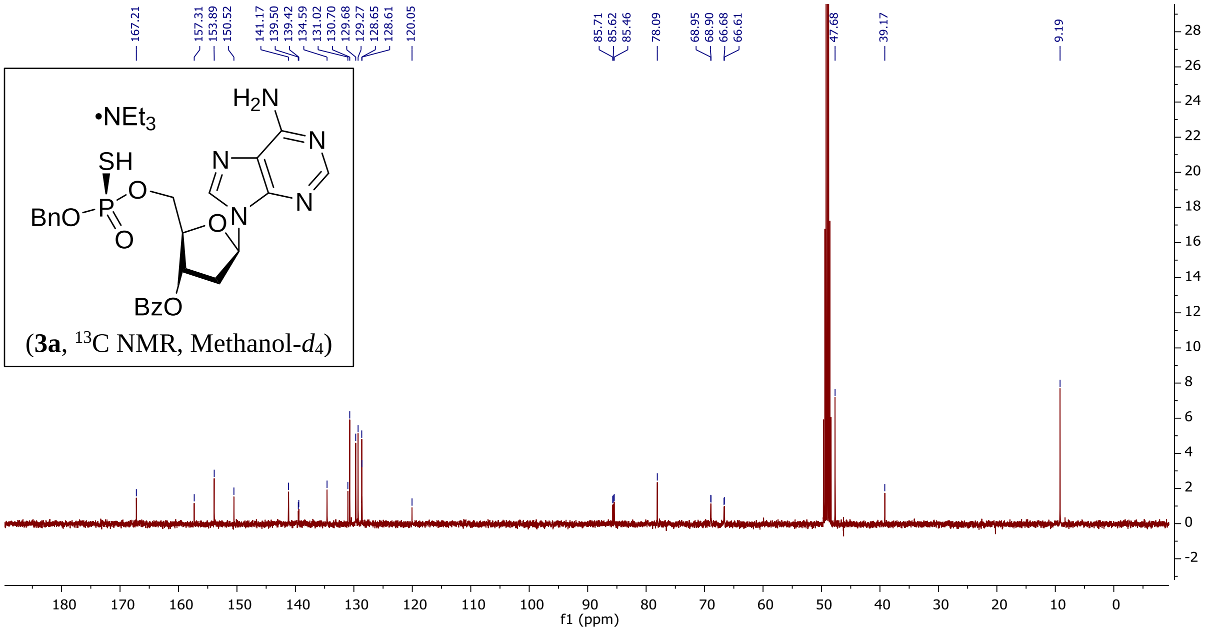

(**3a**, ^{13}C NMR, Methanol-d_4)

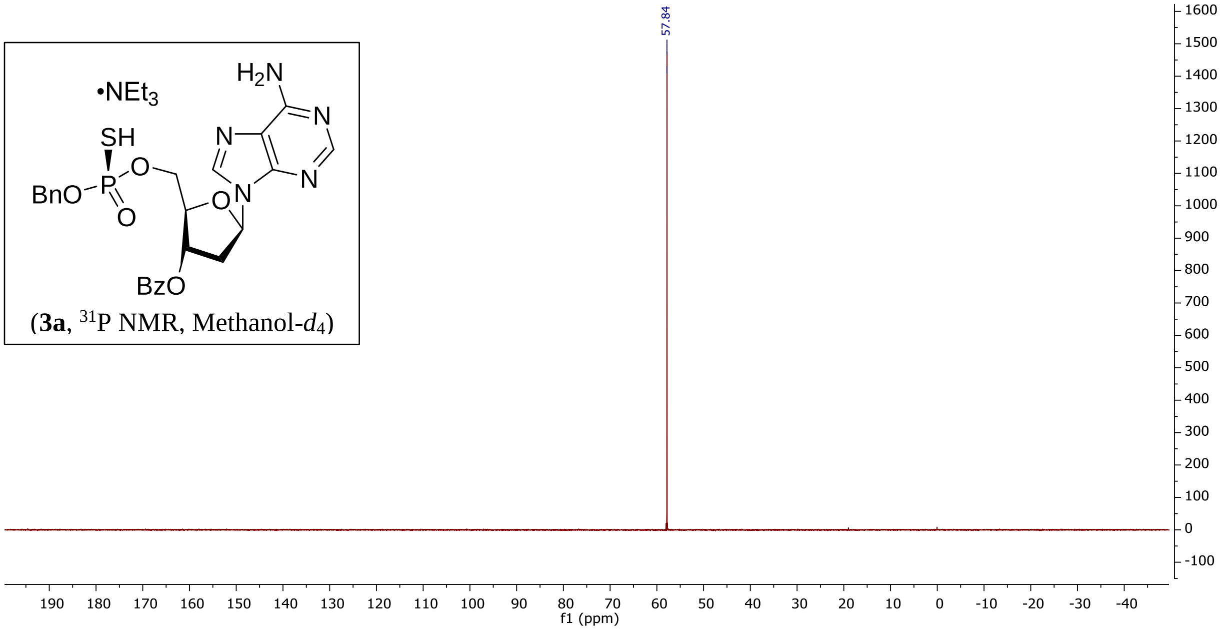

(**3a**, ^{31}P NMR, Methanol-d_4)

_O_-(((2_R_,2_R_)-5-(6-benzamido-9_H_-purin-9-yl)-3-hydroxytetrahydrofuran-2-yl)methyl) _O_-benzyl (_S_)-phosphorothioate triethylammonium (4a)

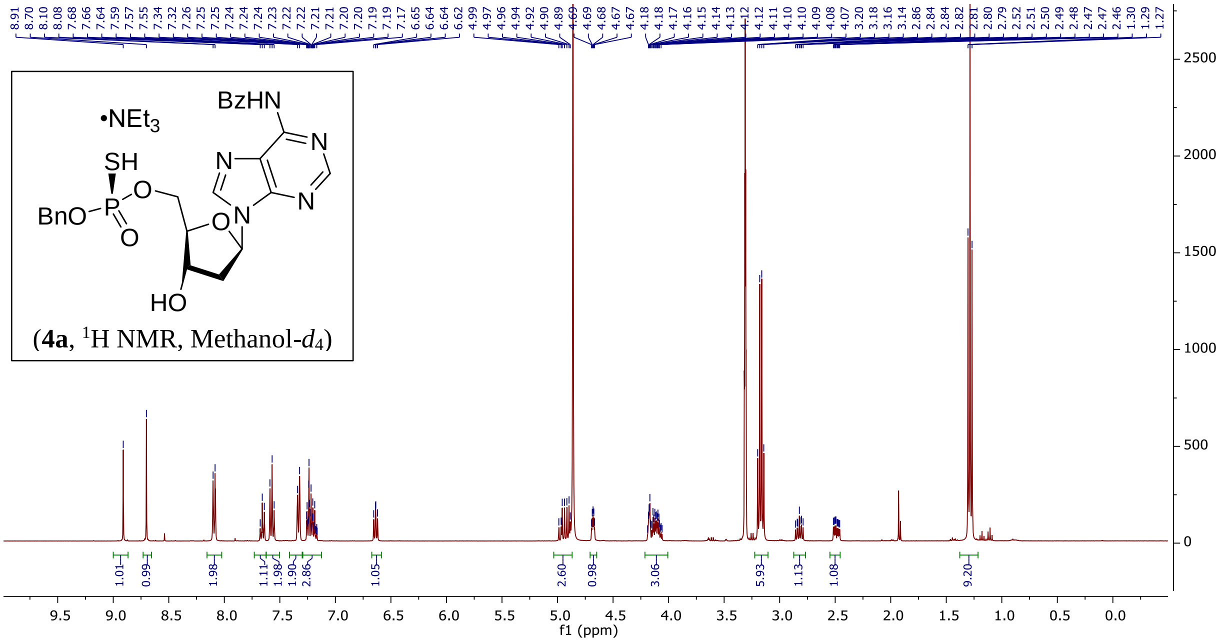

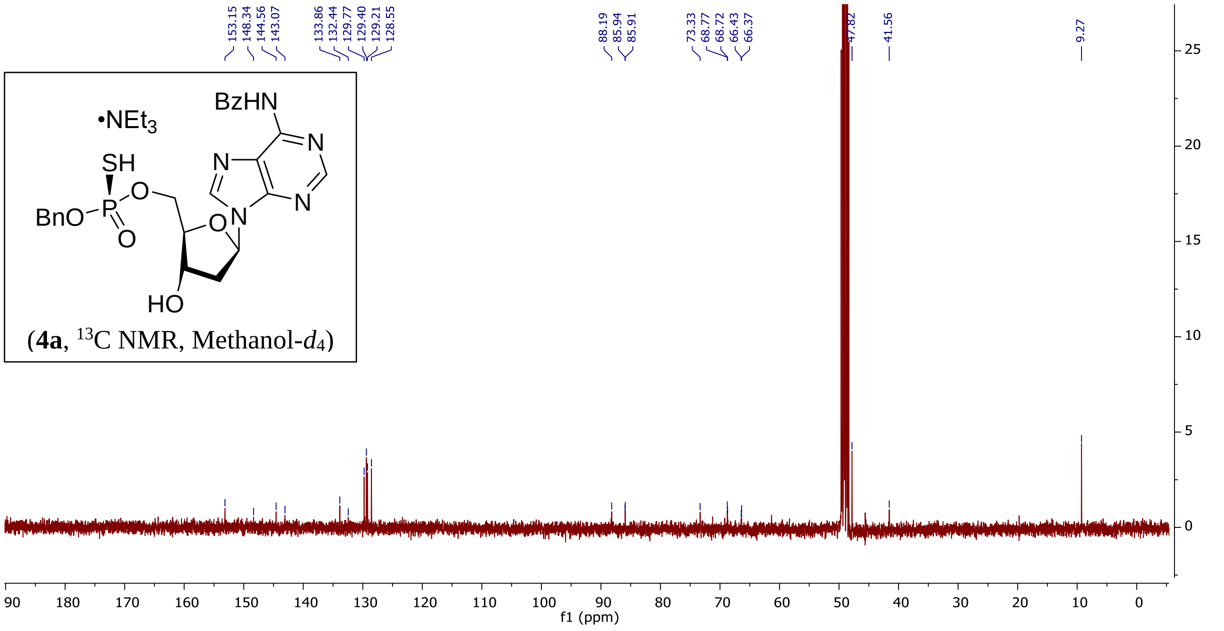

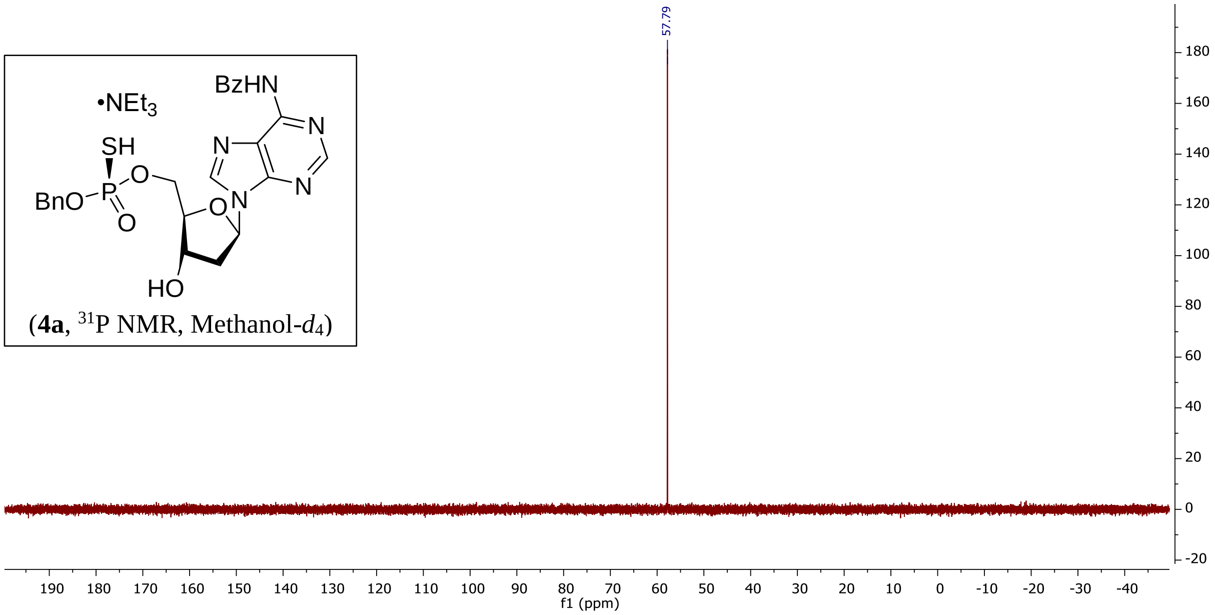

O-(((1R,4R)-4-(6-amino-9H-purin-9-yl)-2-hydroxycyclopentyl)methyl) O-benzyl (R)-phosphorothioate triethylammonium (3b)

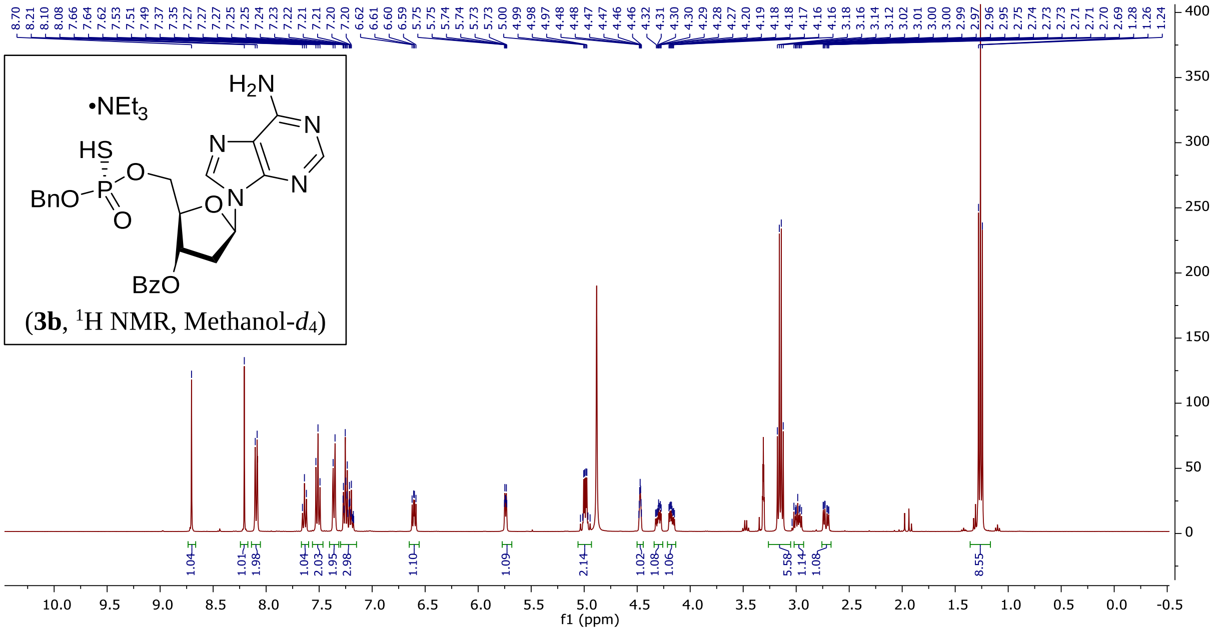

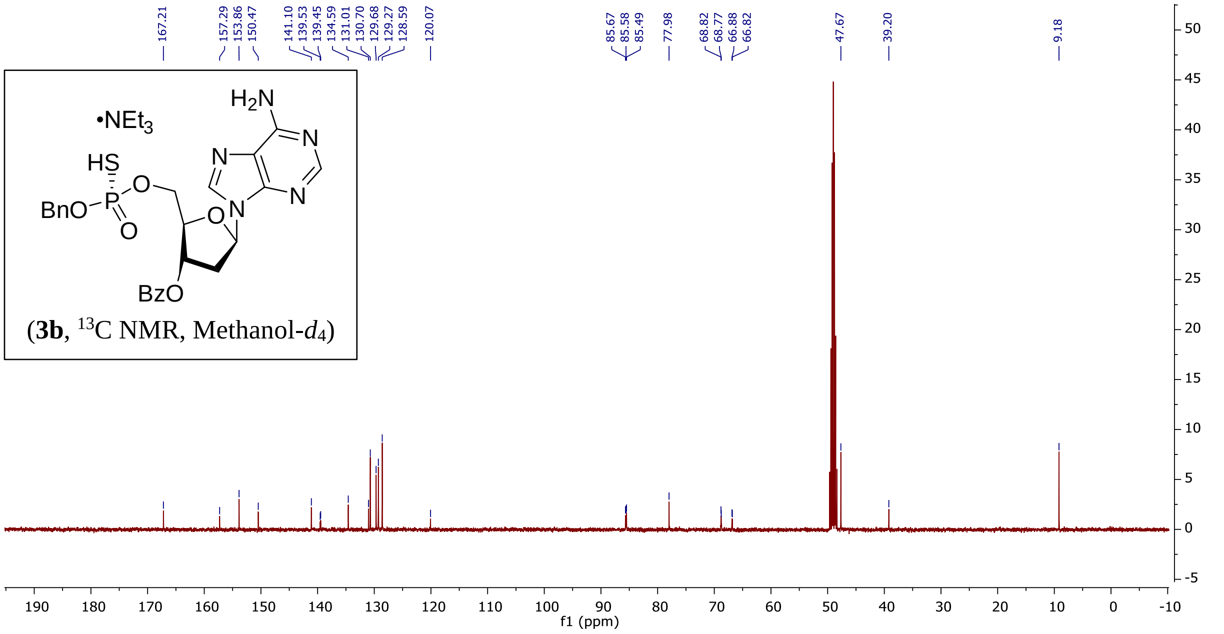

(**3b**, ^{13}C NMR, Methanol-d_4)

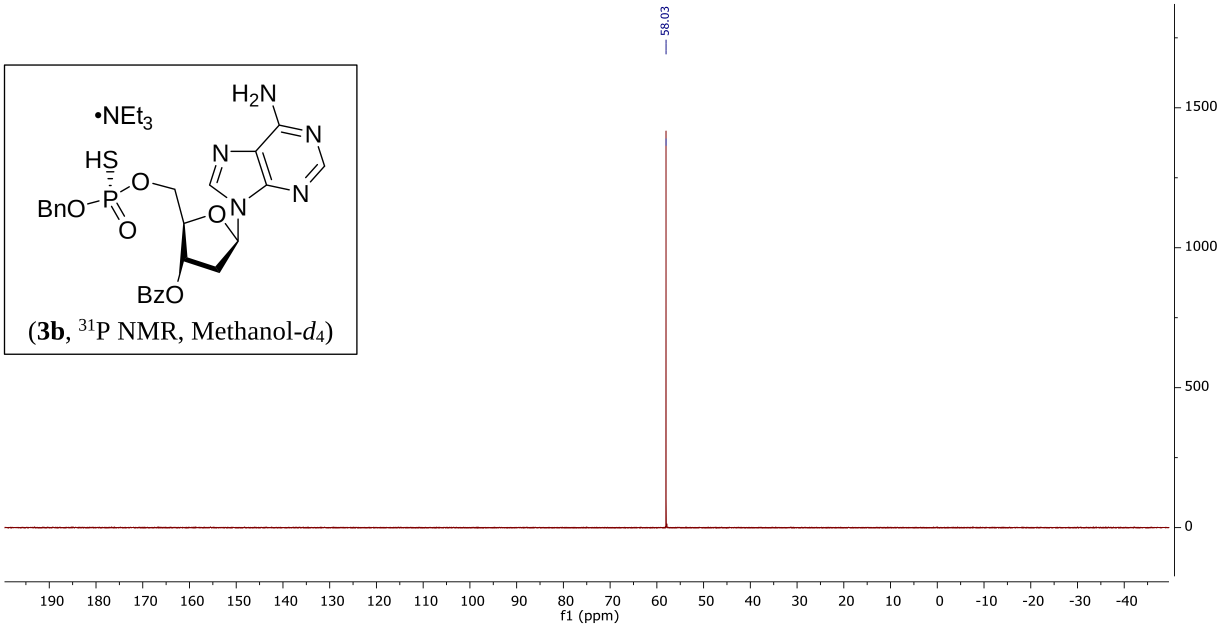

(**3b**, ^{31}P NMR, Methanol-d_4)

***O*-(((1*R*,4*R*)-4-(6-benzamido-9*H*-purin-9-yl)-2-hydroxycyclopentyl)methyl) *O*-benzyl (*R*)-phosphorothioate triethylammonium (4b)**

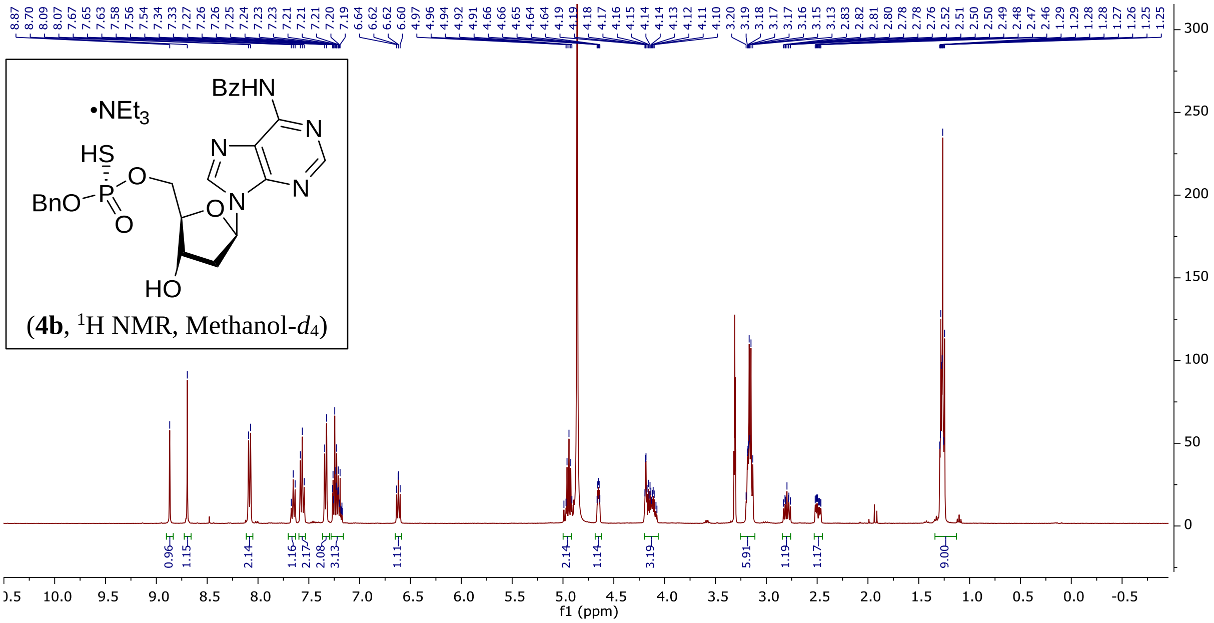

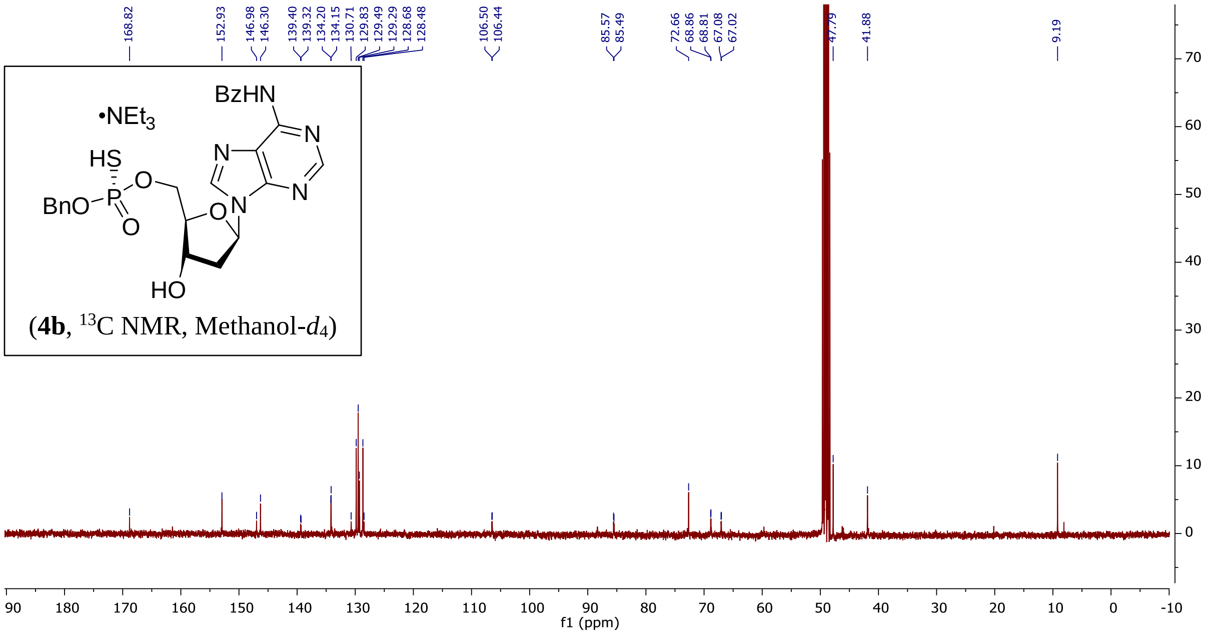

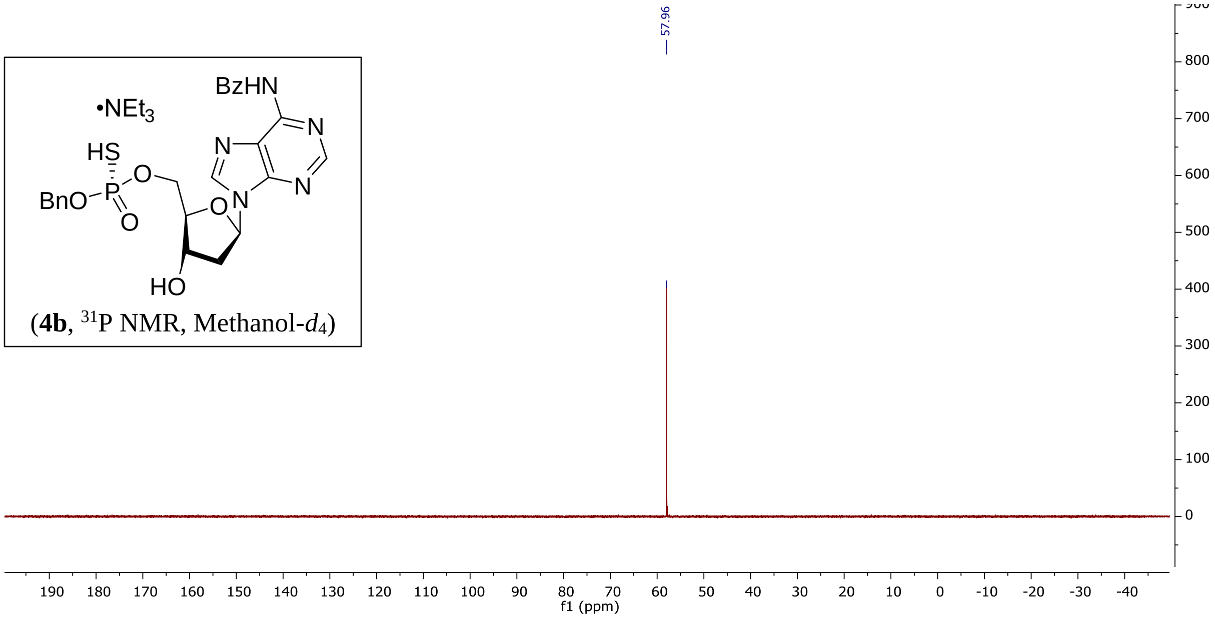

((*2R,2R*)-5-(6-amino-9*H*-purin-9-yl)-3-(benzoyloxy)tetrahydrofuran-2-yl)methyl benzyl phosphate triethylammonium (3c)

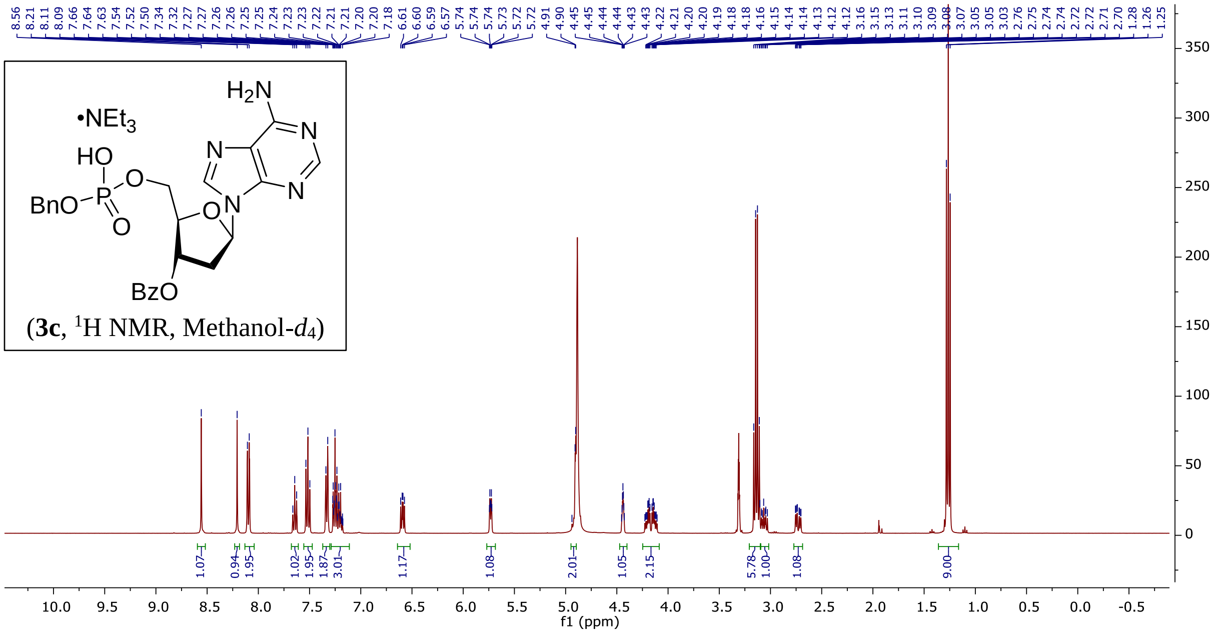

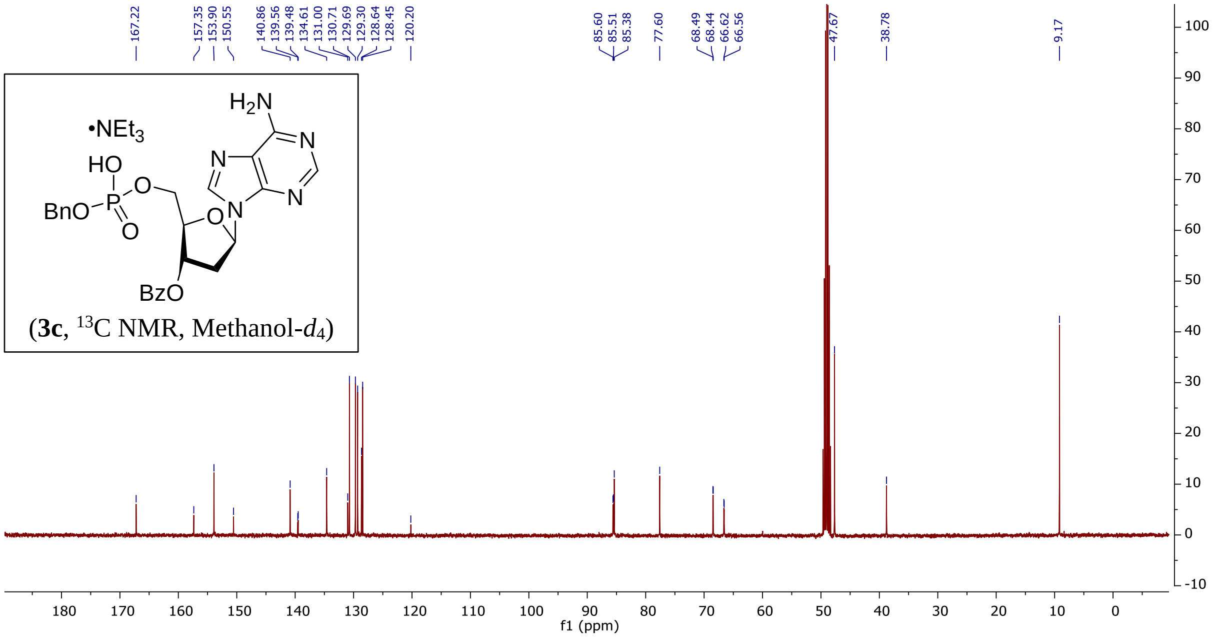

(**3c**, ^{13}C NMR, Methanol-d_4)

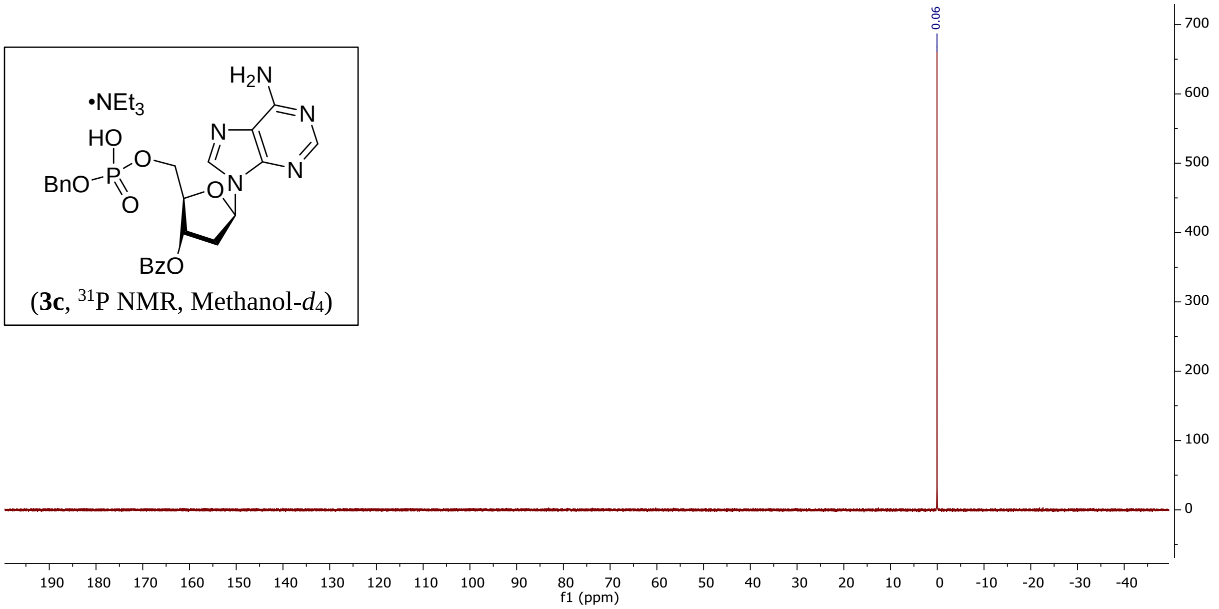

(**3c**, ^{31}P NMR, Methanol-d_4)

((*2R*,*2R*)-5-(6-benzamido-9*H*-purin-9-yl)-3-hydroxytetrahydrofuran-2-yl)methyl benzyl phosphate triethylammonium (4c)

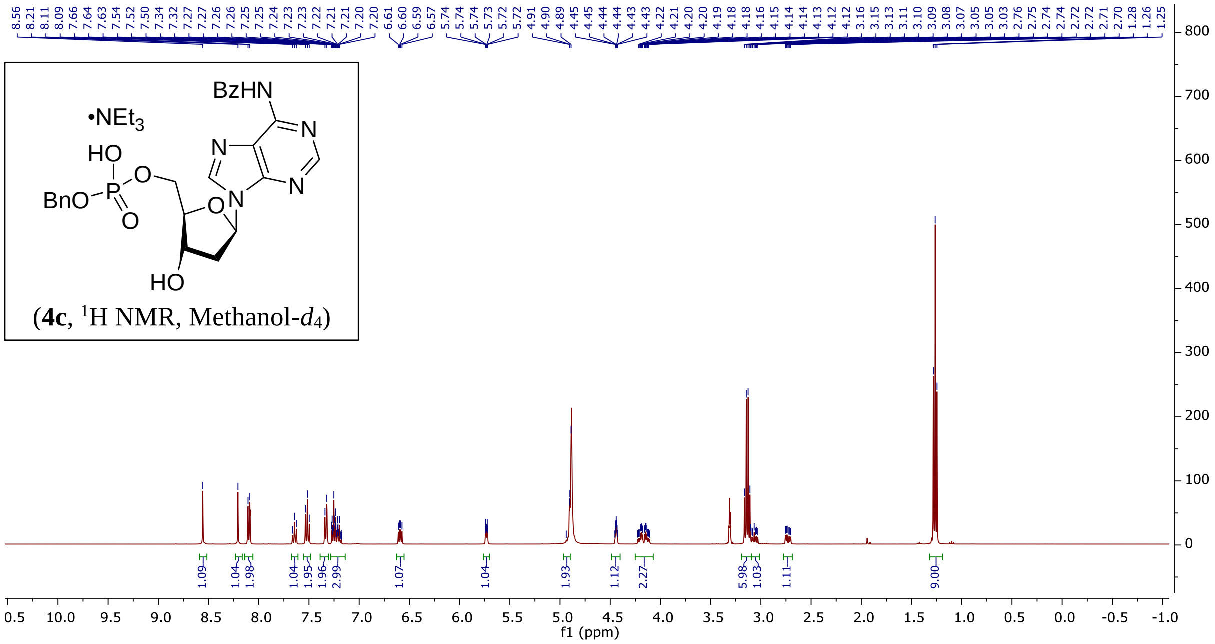

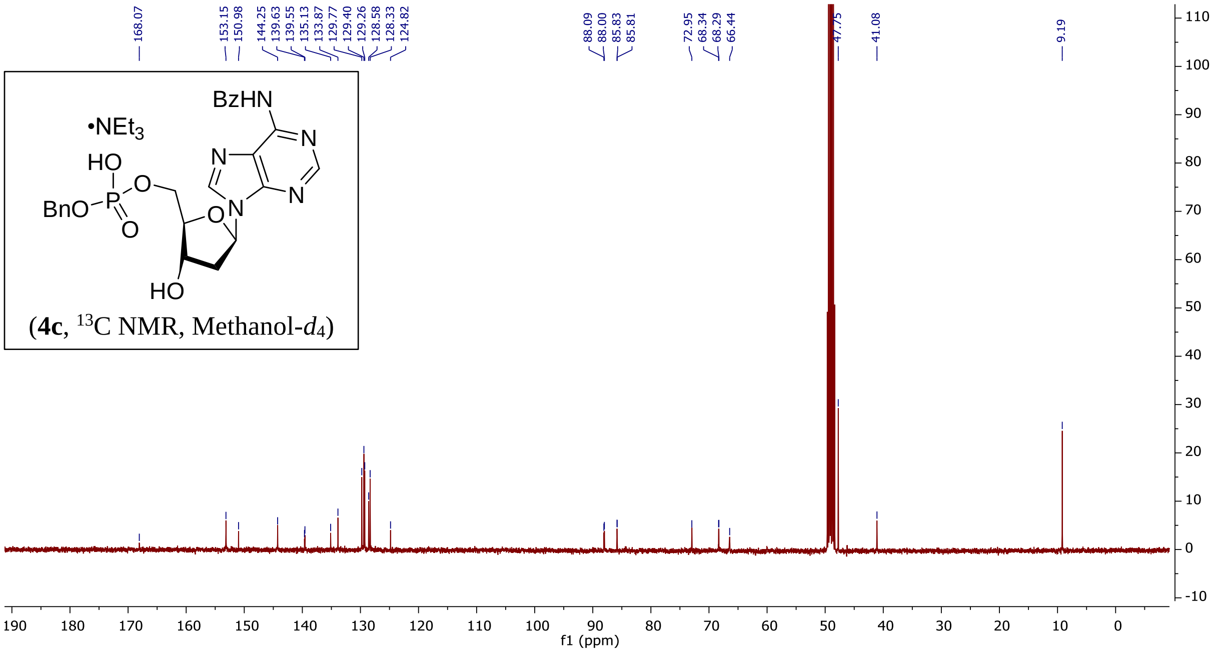

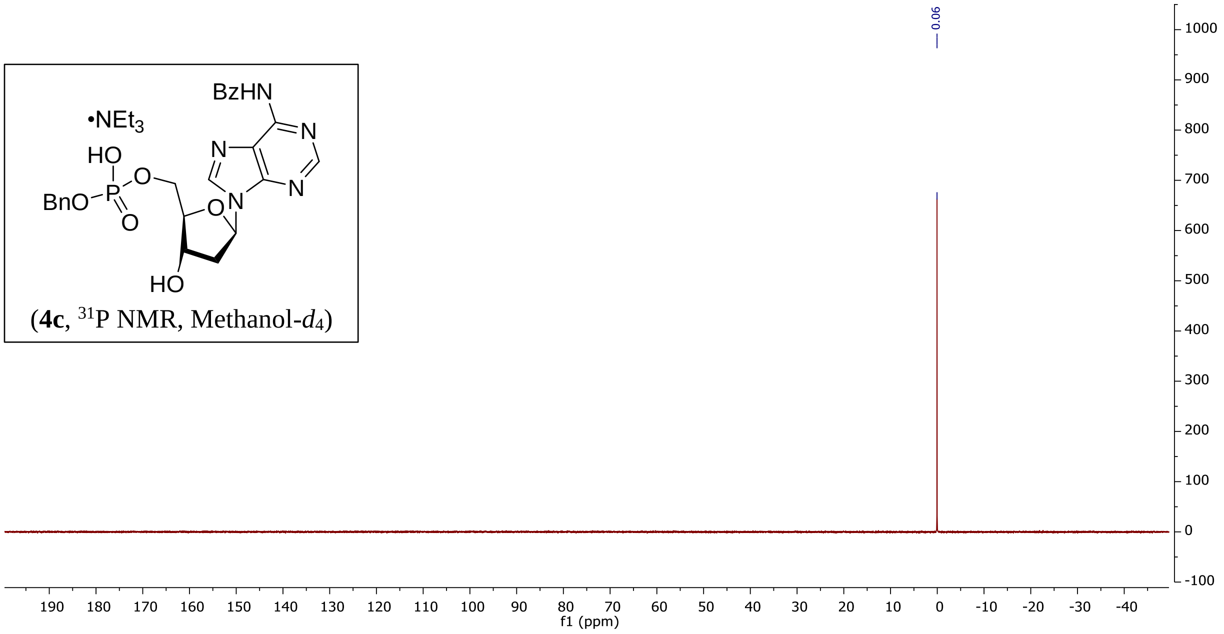

(*2R,2R*)-5-(6-amino-9*H*-purin-9-yl)-2-(((*tert*-butyldimethylsilyl)oxy)methyl)tetrahydrofuran-3-yl benzoate (3d)

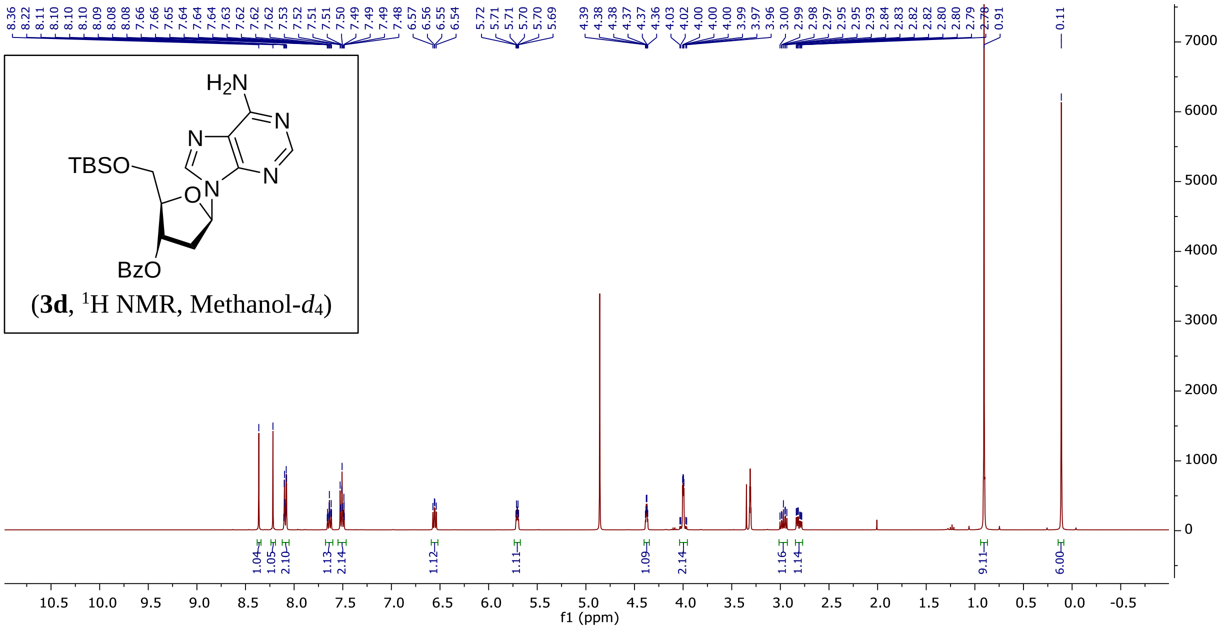

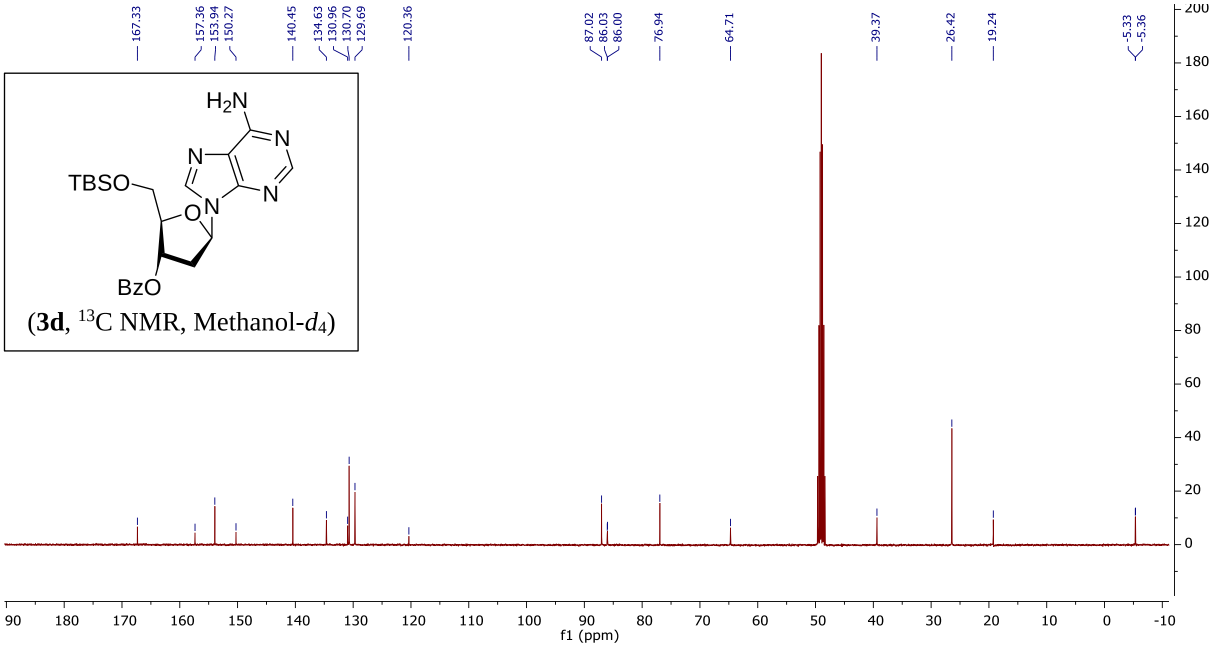

N-(9-((2R,2R)-5-(((tert-butyldimethylsilyl)oxy)methyl)-4-hydroxytetrahydrofuran-2-yl)-9H-purin-6-yl)benzamide (4d)

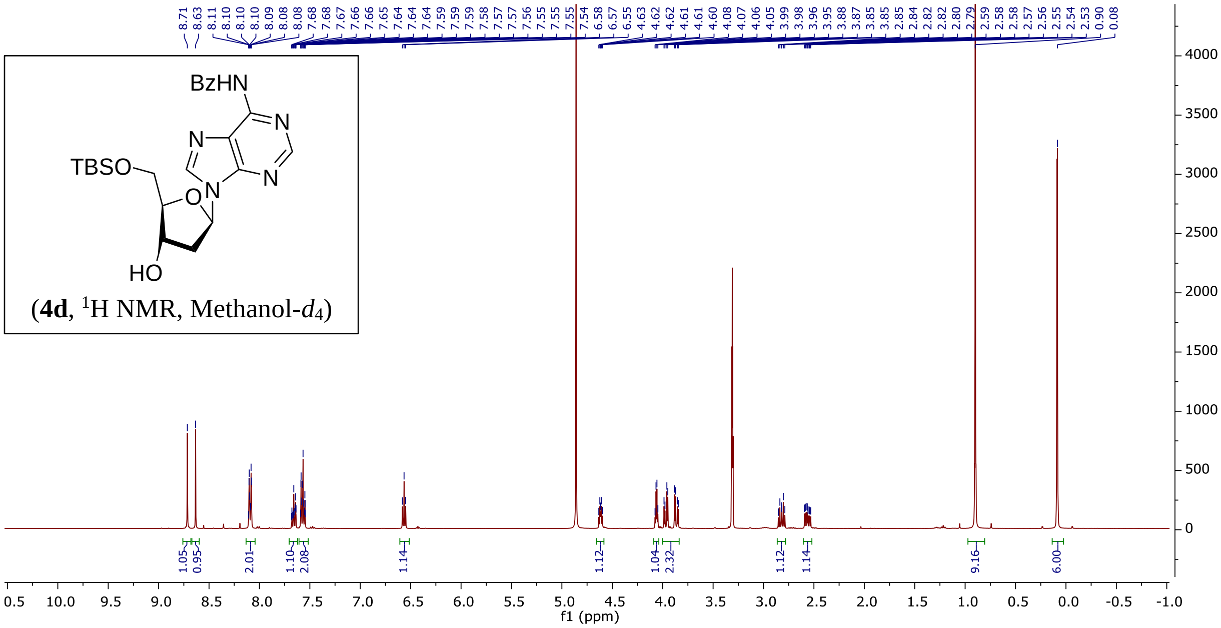

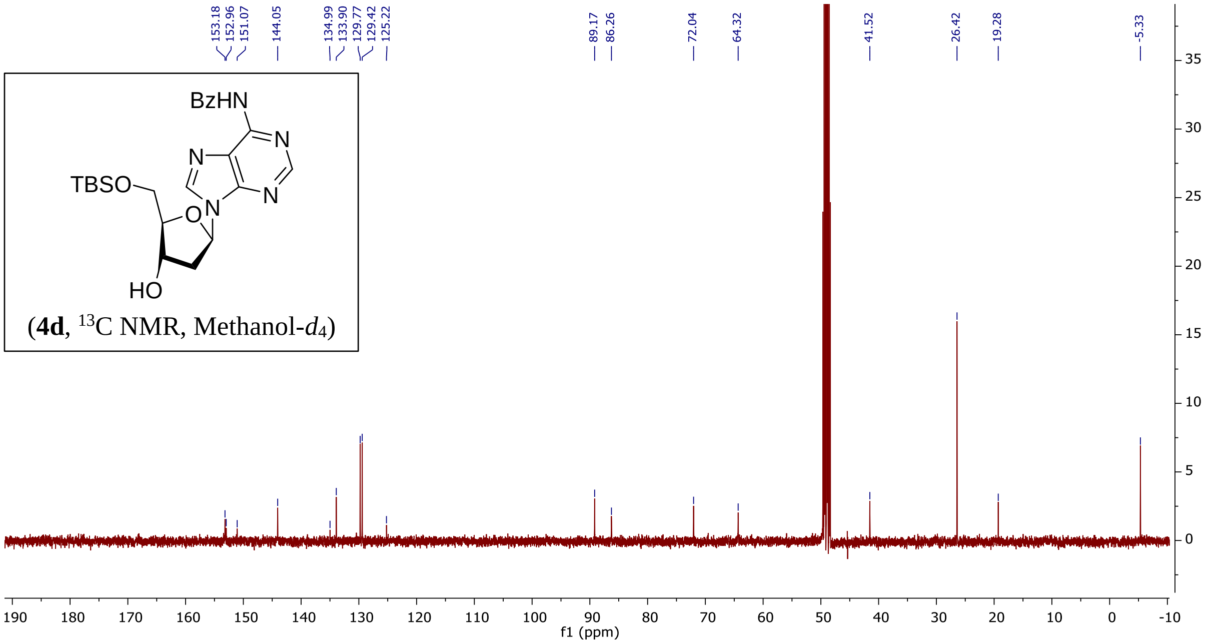

((*3aR*,*6aR*)-6-(6-amino-9*H*-purin-9-yl)-2,2-dimethyltetrahydrofuro[3,4-*d*][1,3]dioxol-4-yl)methyl benzoate (3e)

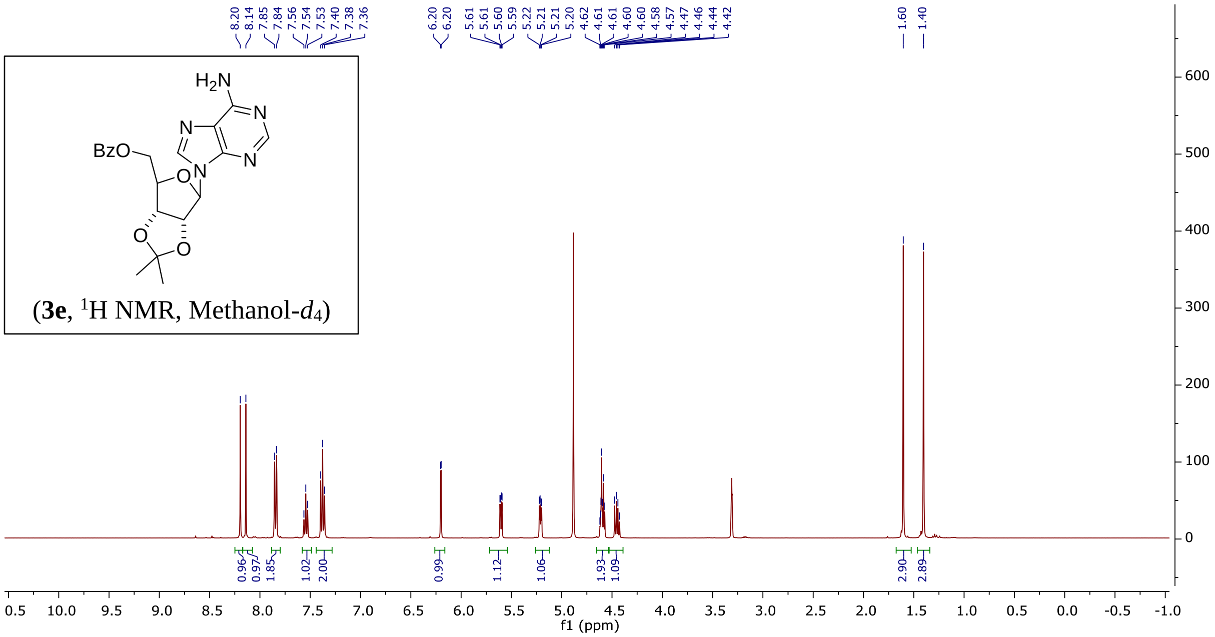

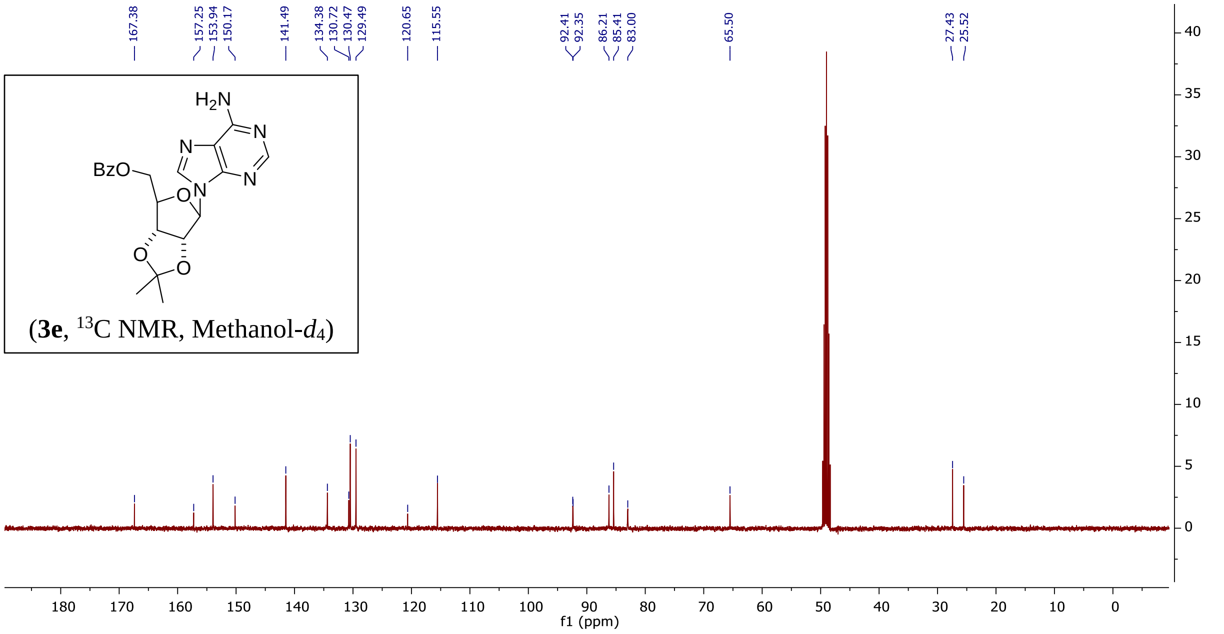

N-(9-((*3aR,6aR*)-6-(hydroxymethyl)-2,2-dimethyltetrahydrofuro[3,4-*d*][1,3]dioxol-4-yl)-9*H*-purin-6-yl)benzamide (4e)

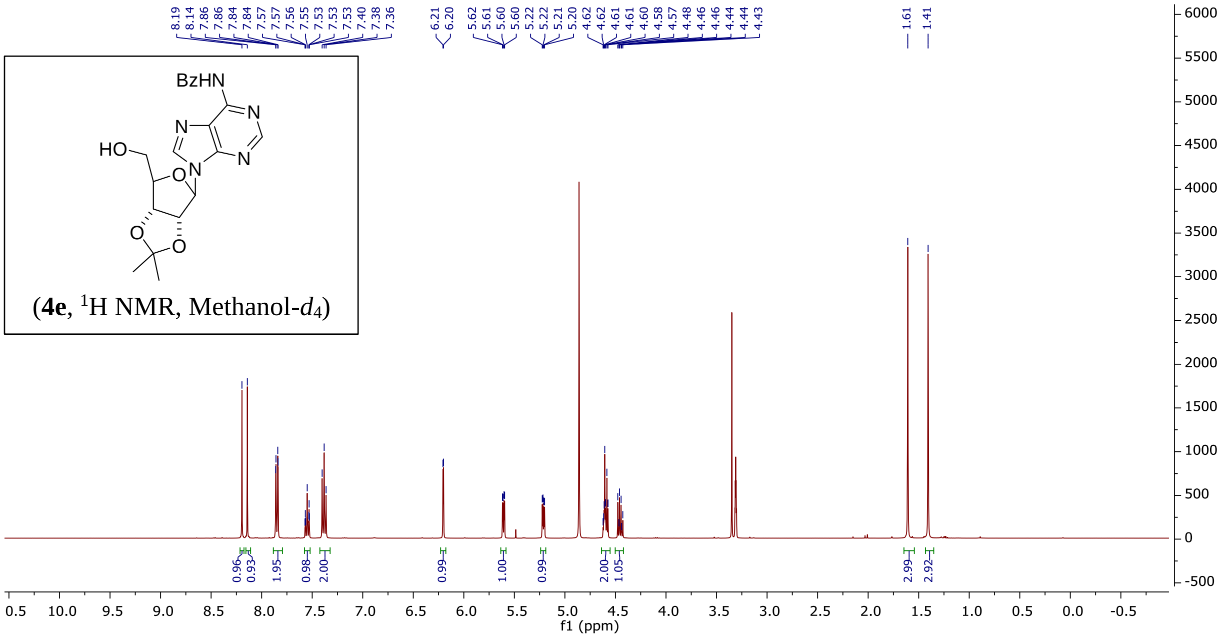

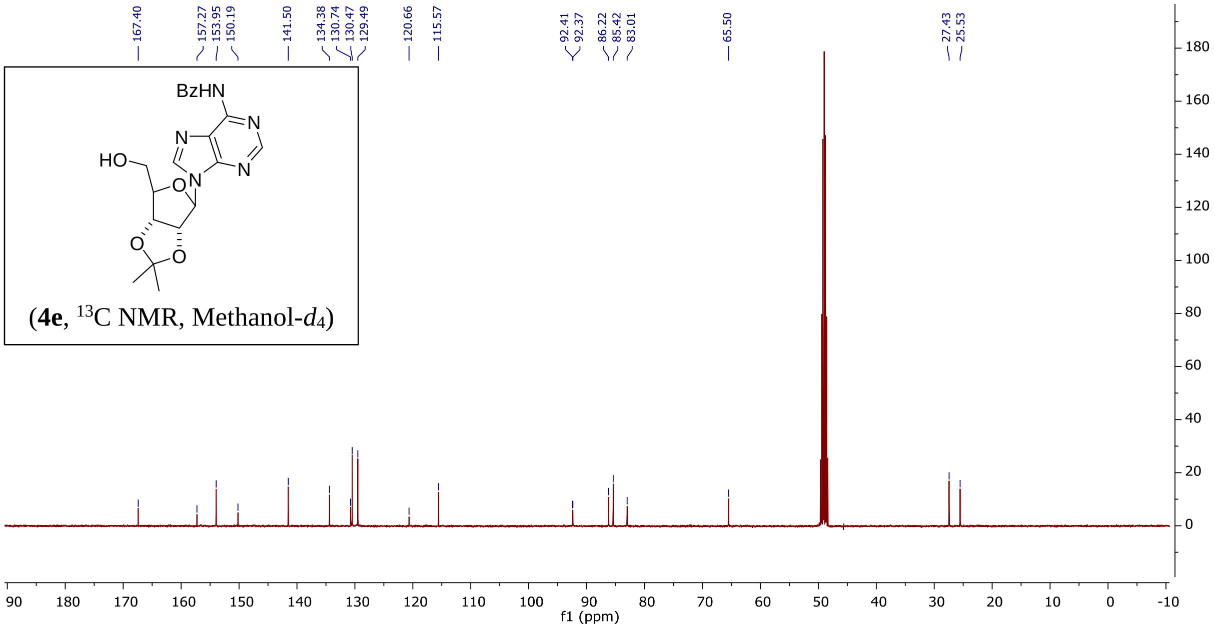

(9*R*,9*aR*)-8-(6-amino-9*H*-purin-9-yl)-2,2,4,4-tetraisopropyltetrahydro-6*H*-furo[3,2-*f*][1,3,5,2,4]trioxadisilocin-9-yl benzoate (3f)

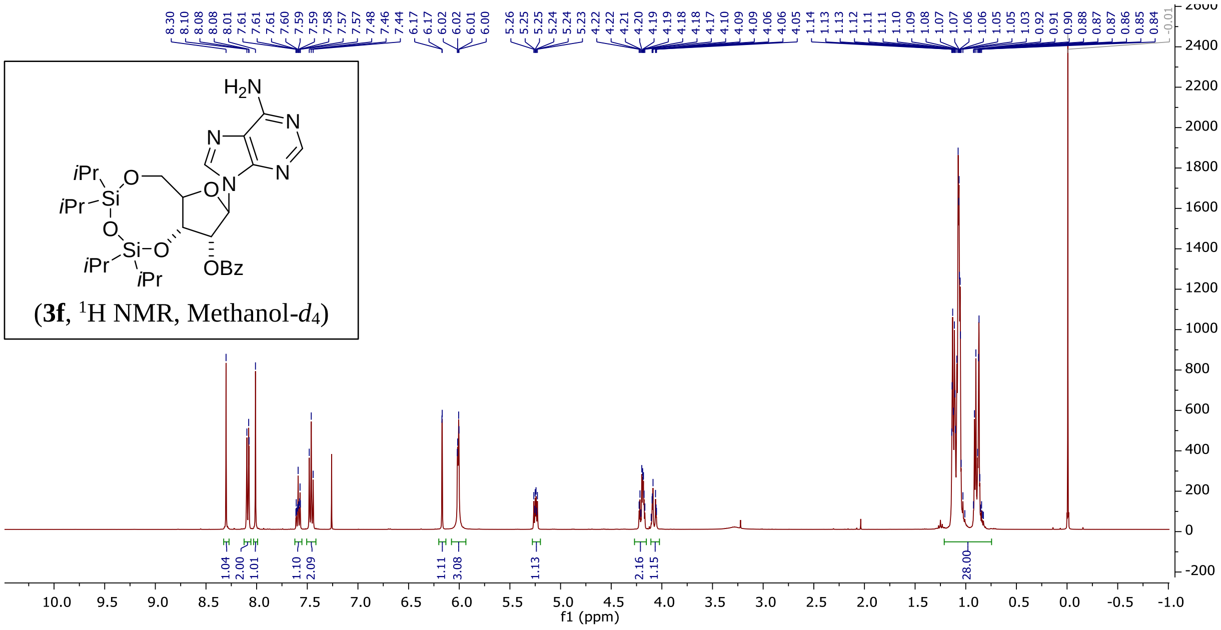

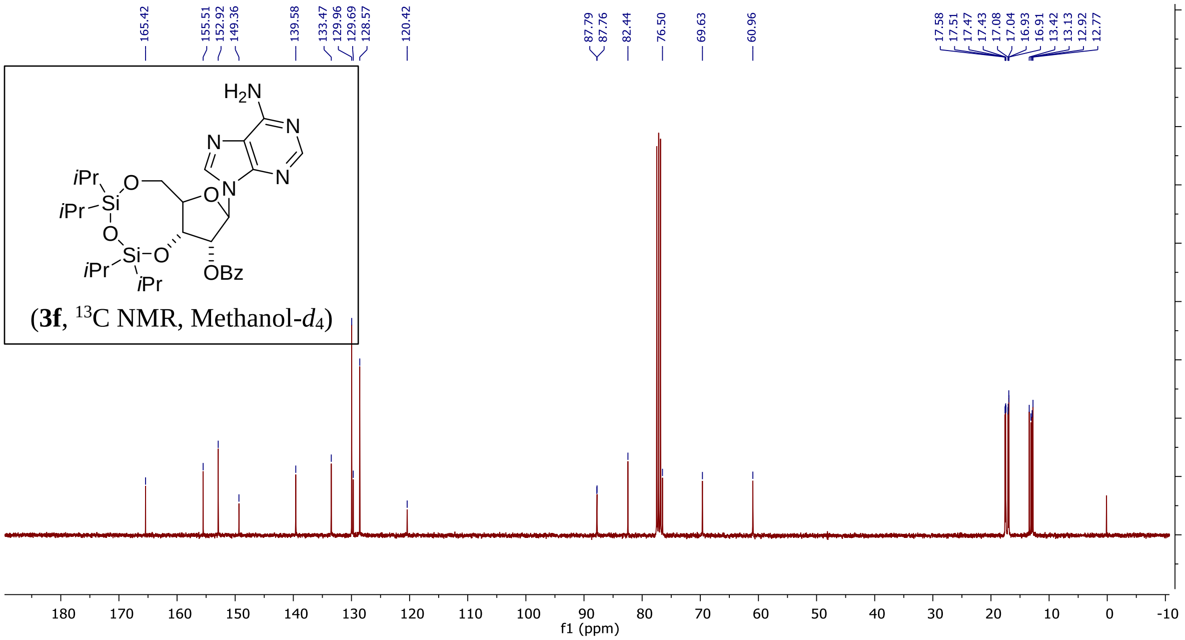

(**3f**, ^{13}C NMR, Methanol-d_4)

N-(9-((9*R*,9*aS*)-9-hydroxy-2,2,4,4-tetraisopropyltetrahydro-6*H*-furo[3,2-*f*][1,3,5,2,4]trioxadisilocin-8-yl)-9*H*-purin-6-yl)benzamide (4f)

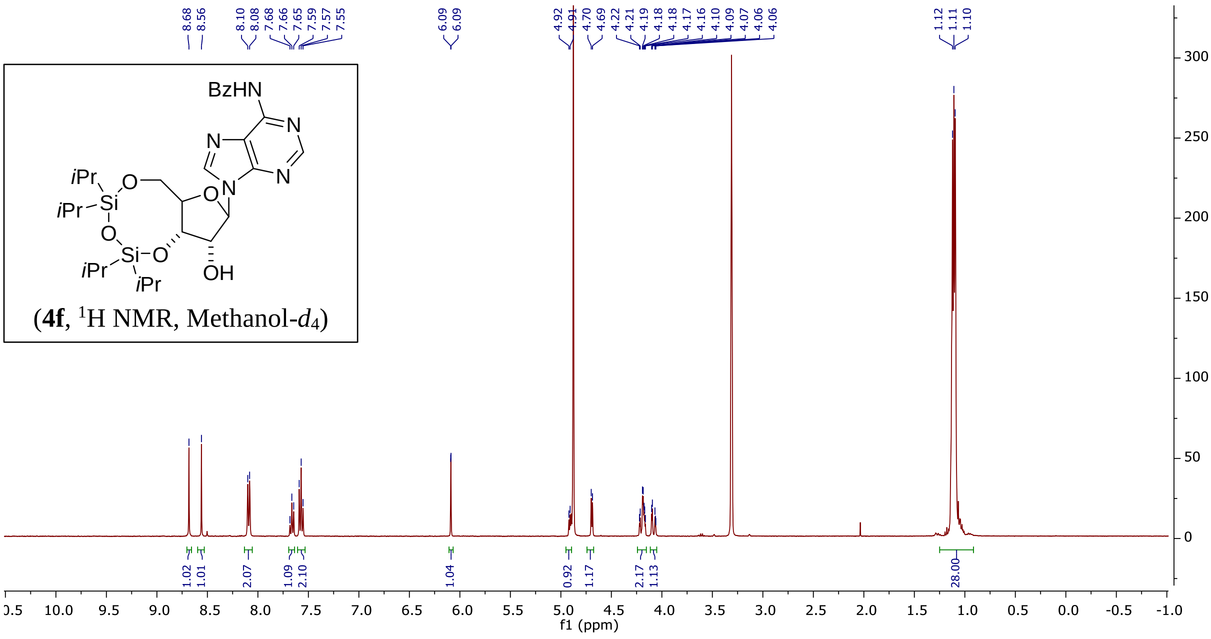

(**4f**, ^{1}H NMR, Methanol-d_4)

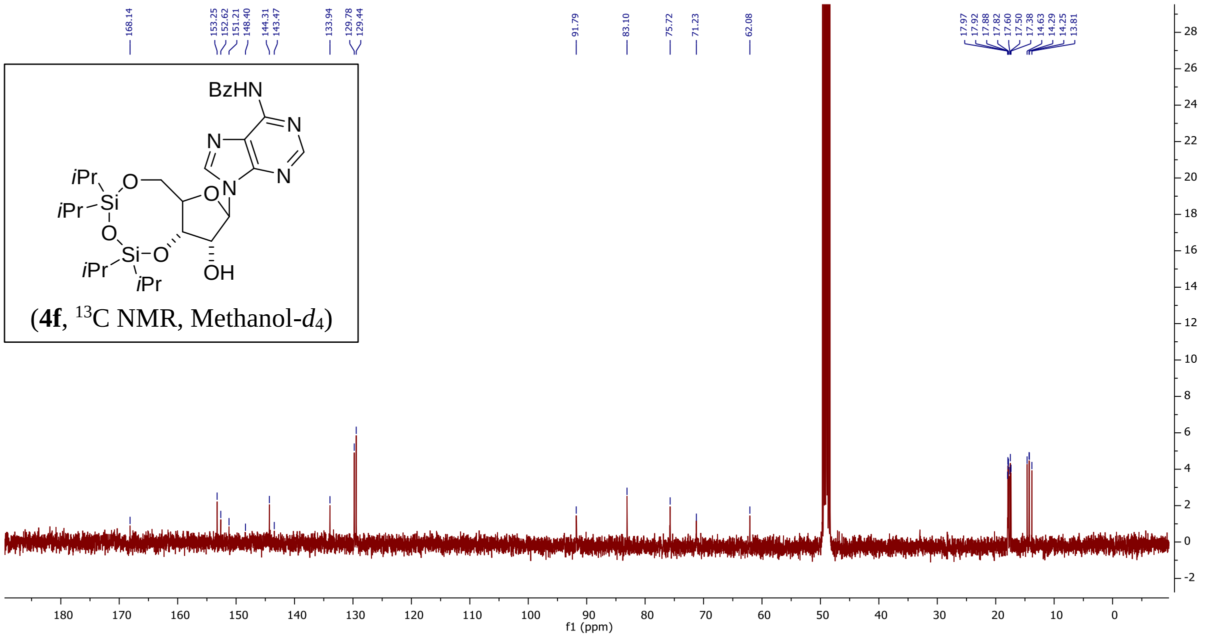

(**4f**, ^{13}C NMR, Methanol-d_4)

(*2R*,*5R*)-5-(4-amino-2-oxopyrimidin-1(2*H*)-yl)-2-(((*tert*-butyldimethylsilyl)oxy)methyl)tetrahydrofuran-3-yl benzoate (3g)

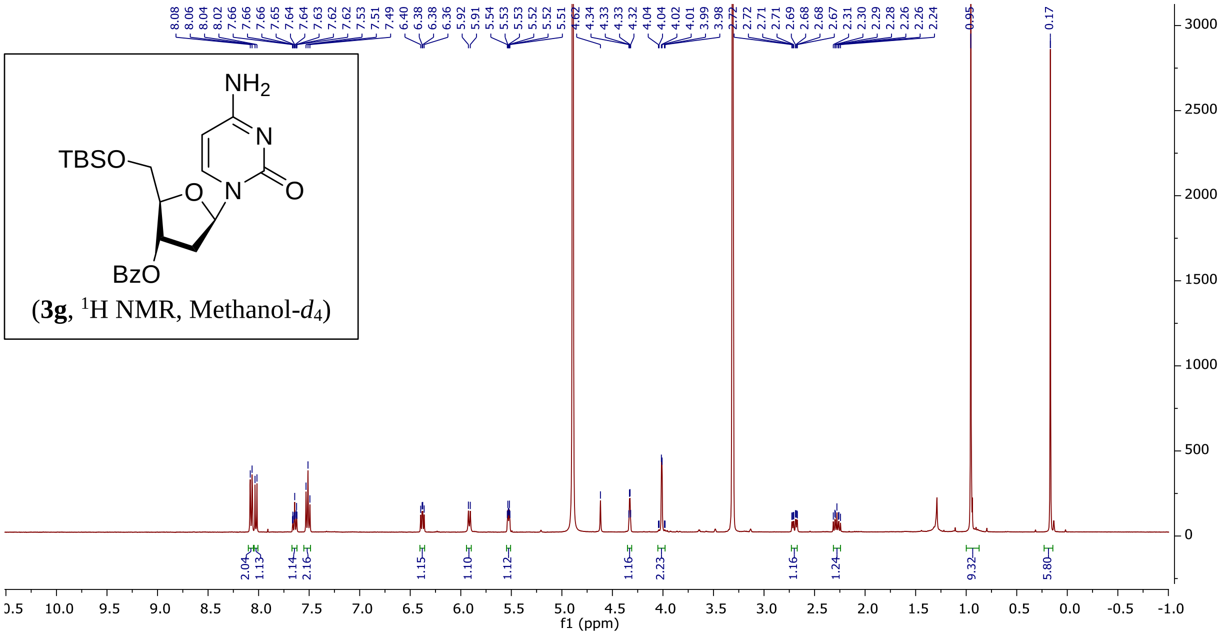

(**3g**, ^{1}H NMR, Methanol-d_4)

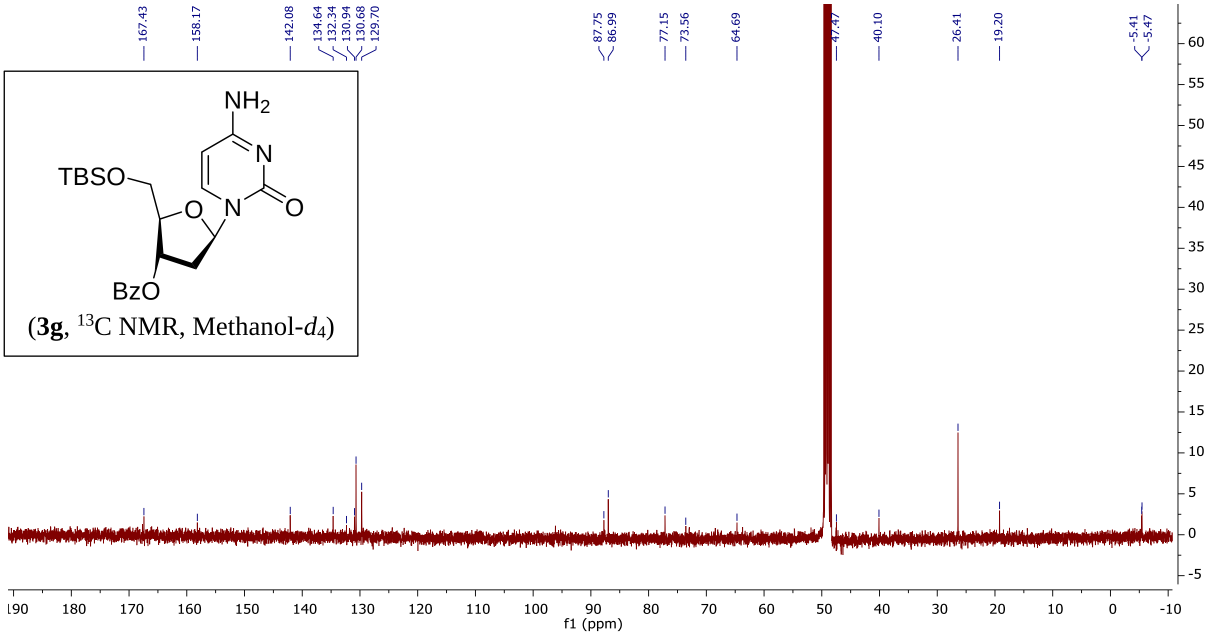

(**3g**, ^{13}C NMR, Methanol-d_4)

N-(1-((2R,5R)-5-(((*tert*-butyldimethylsilyl)oxy)methyl)-4-hydroxytetrahydrofuran-2-yl)-2-oxo-1,2-dihydropyrimidin-4-yl)benzamide (4g)

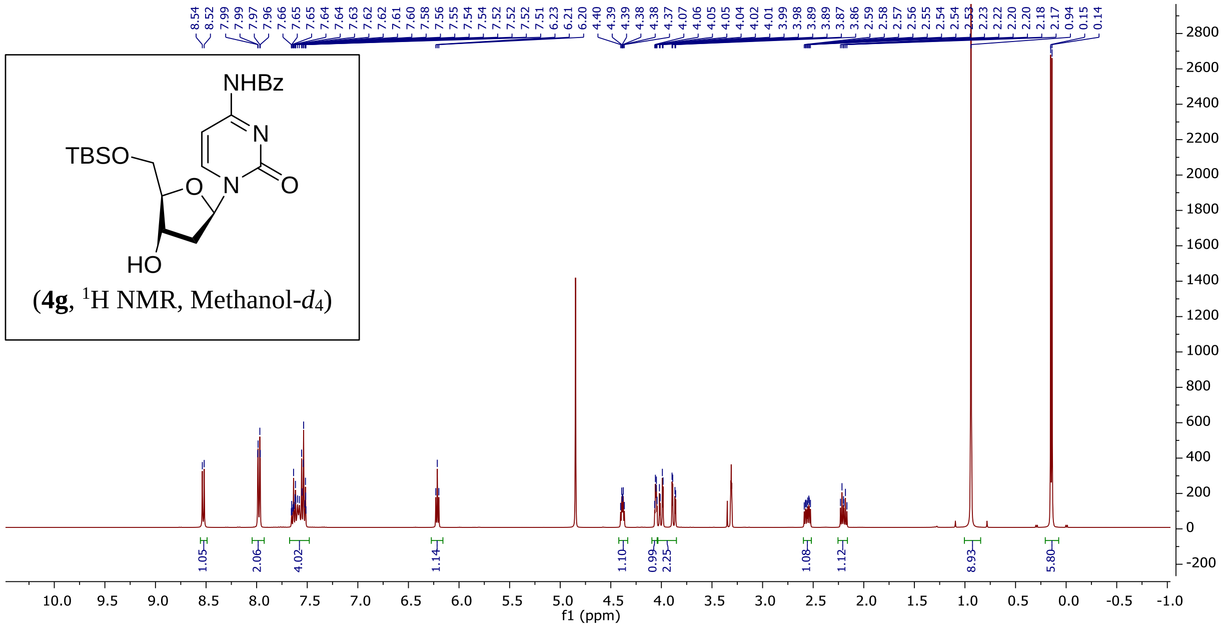

(**4g**, ^{1}H NMR, Methanol-d_4)

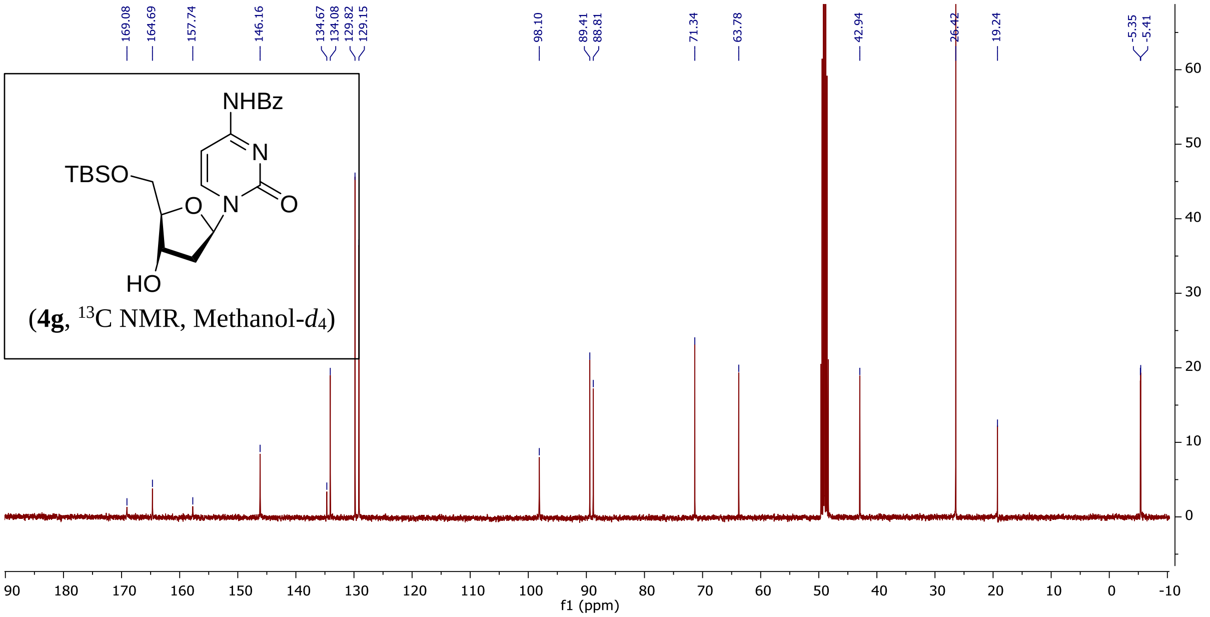

(2*R*,5*R*)-5-(4-benzamido-2-oxopyrimidin-1(2*H*)-yl)-2-(((*tert*-butyldimethylsilyl)oxy)methyl)tetrahydrofuran-3-yl benzoate (5g)

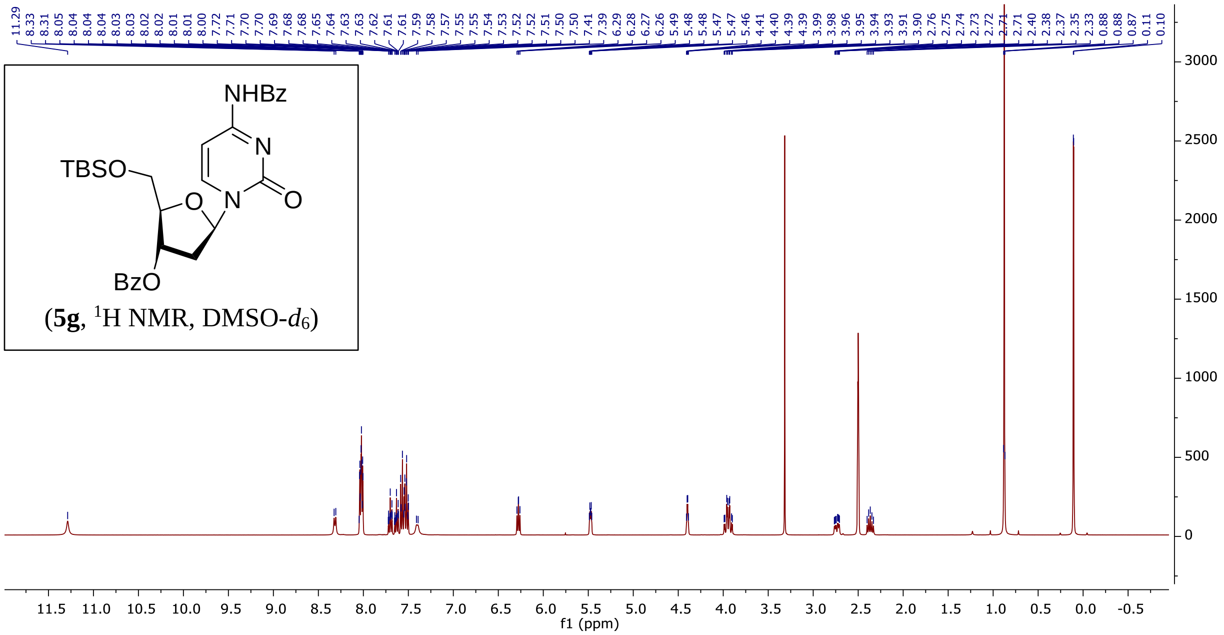

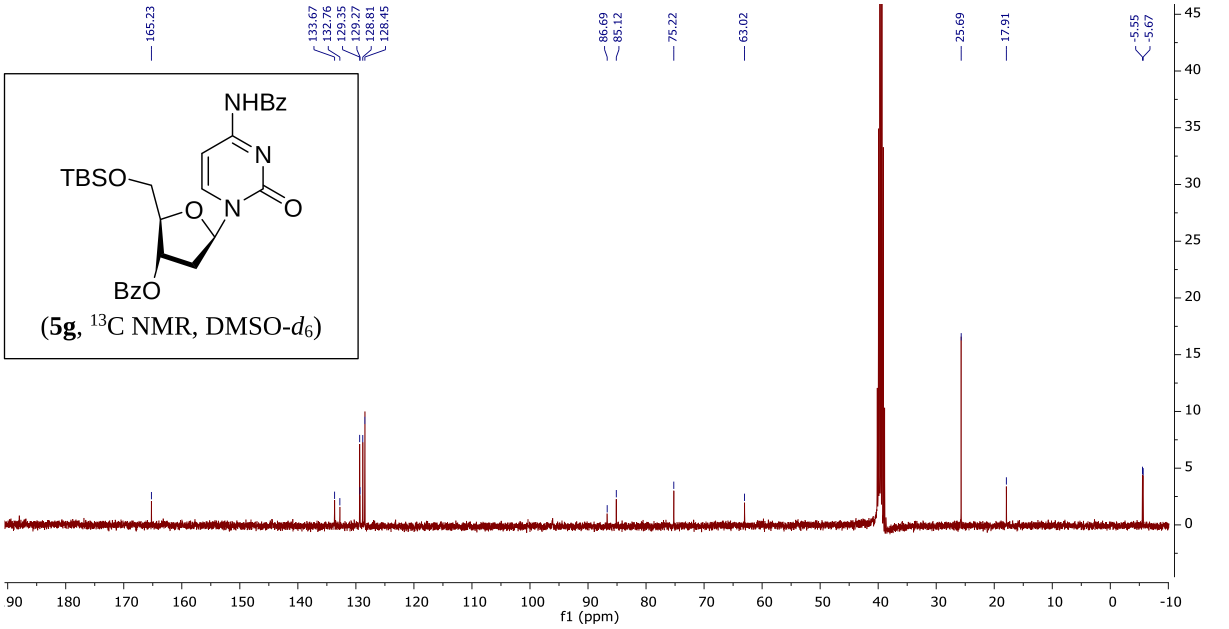

(**5g**, ^{13}C NMR, DMSO-d_6)

(*2R*,5*R*)-5-(2-amino-6-oxo-1,6-dihydro-9*H*-purin-9-yl)-2-(((*tert*-butyldimethylsilyl)oxy)methyl)tetrahydrofuran-3-yl benzoate (3h)

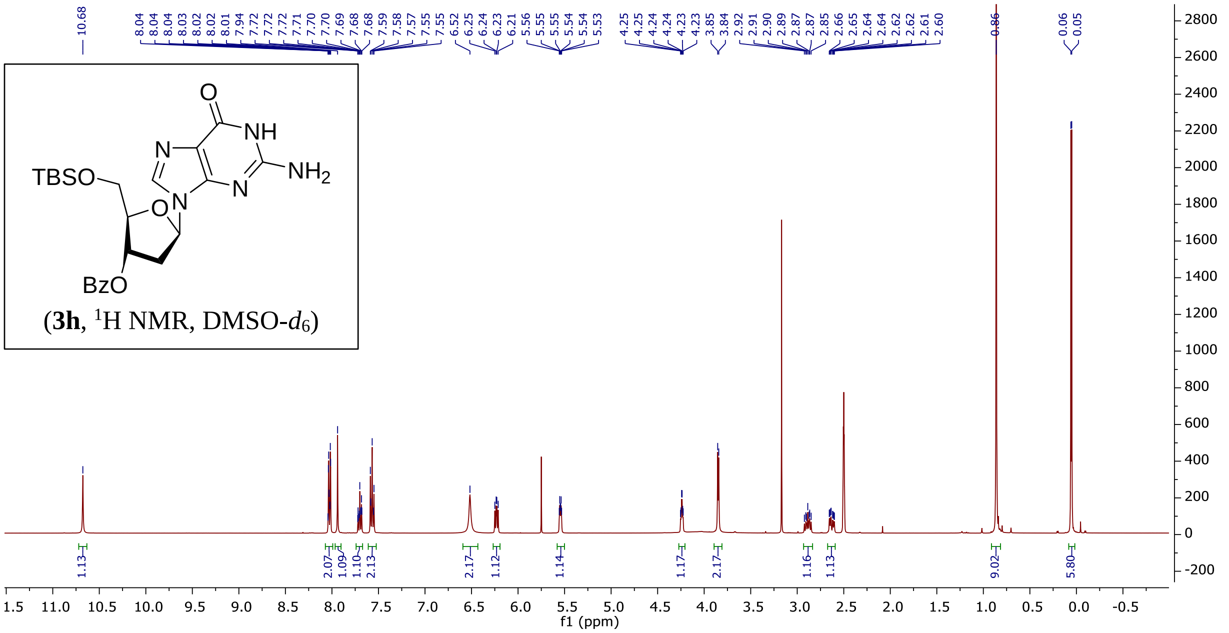

(**3h**, ^{1}H NMR, DMSO-d_6)

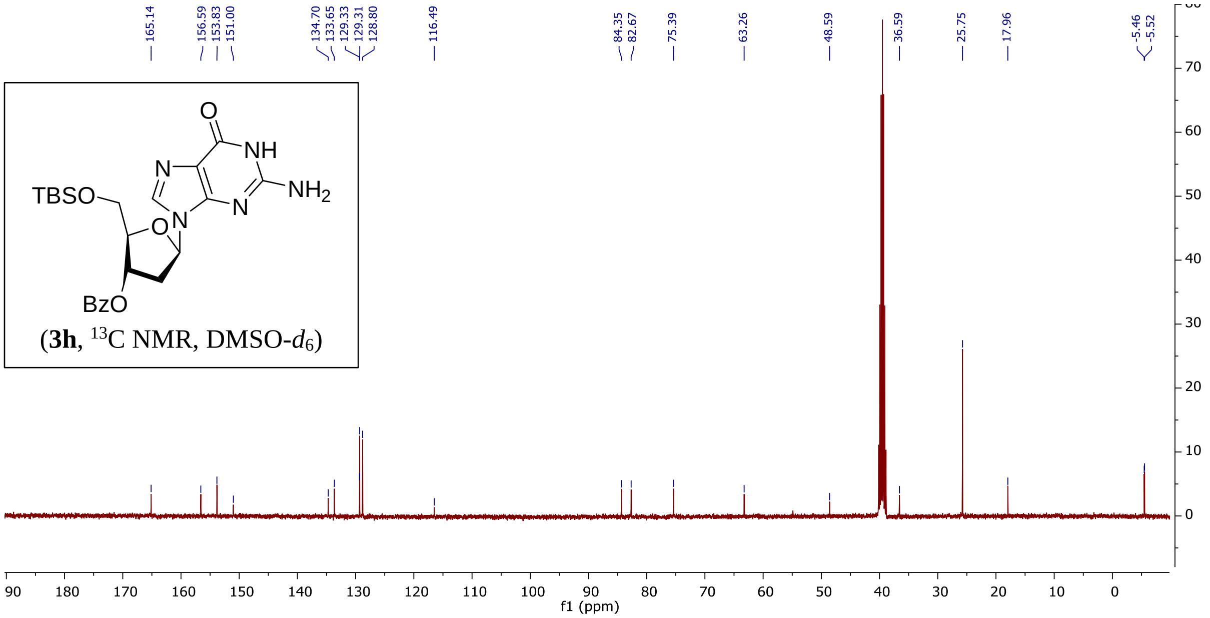

(**3h**, ^{13}C NMR, DMSO-d_6)

O-(((2R,5R)-3-acetoxy-5-(6-amino-9H-purin-9-yl)tetrahydrofuran-2-yl)methyl) O-benzyl (S)-phosphorothioate triethylammonium (3ab)

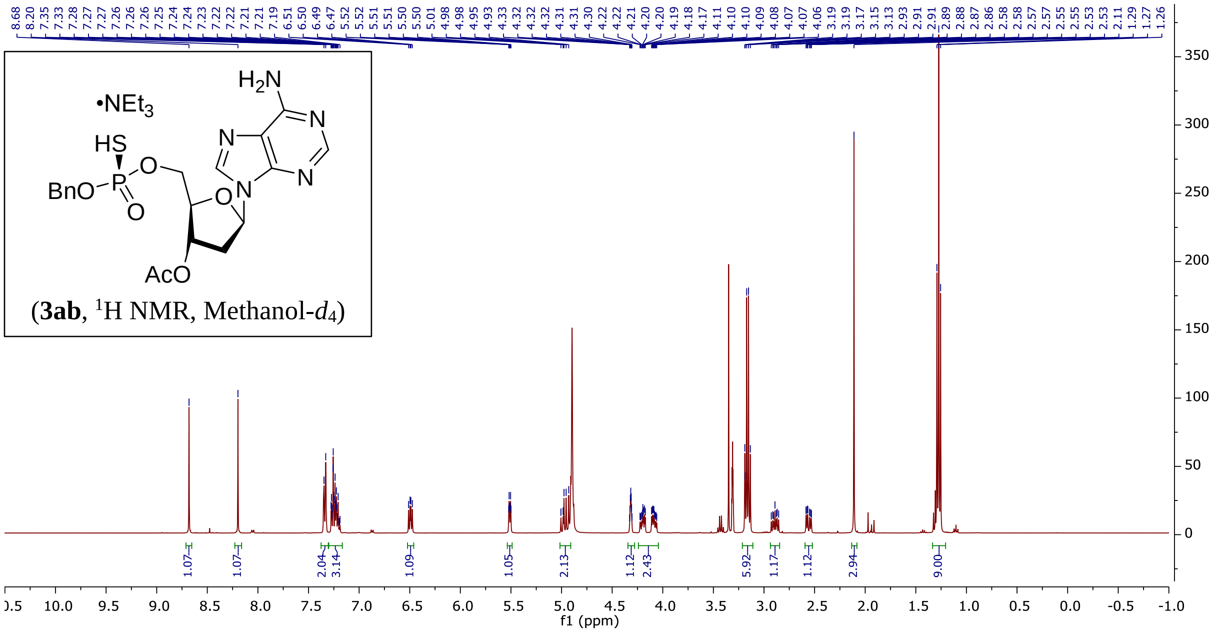

(**3ab**, ^{1}H NMR, Methanol-d_4)

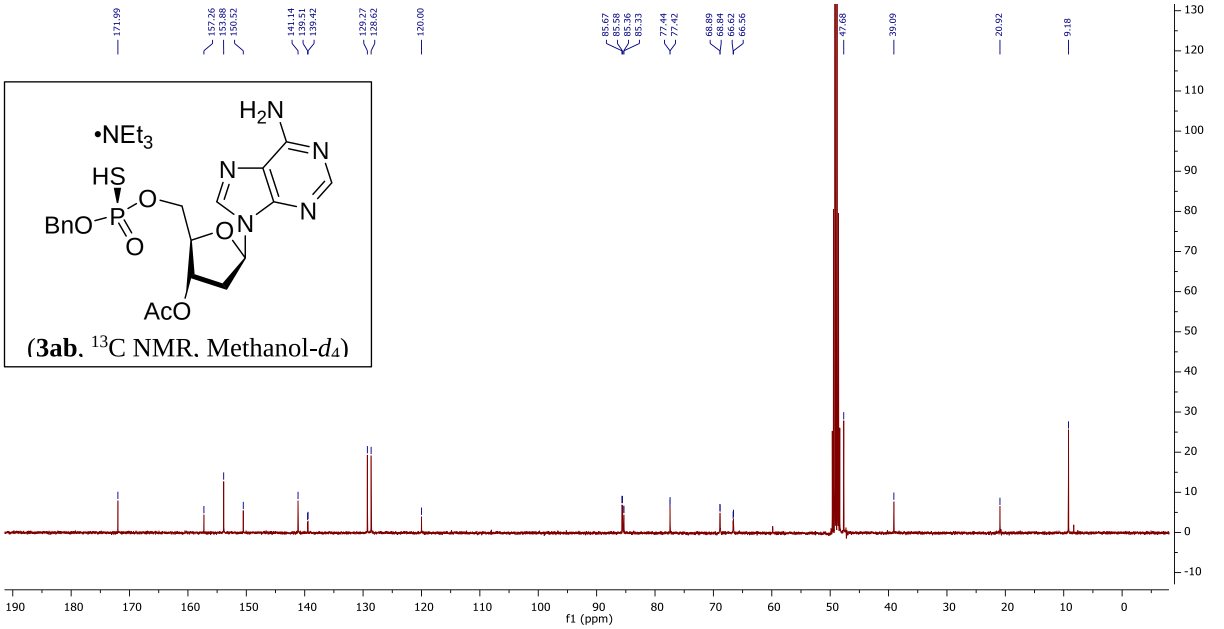

(**3ab**, ^{13}C NMR, Methanol-d_4)

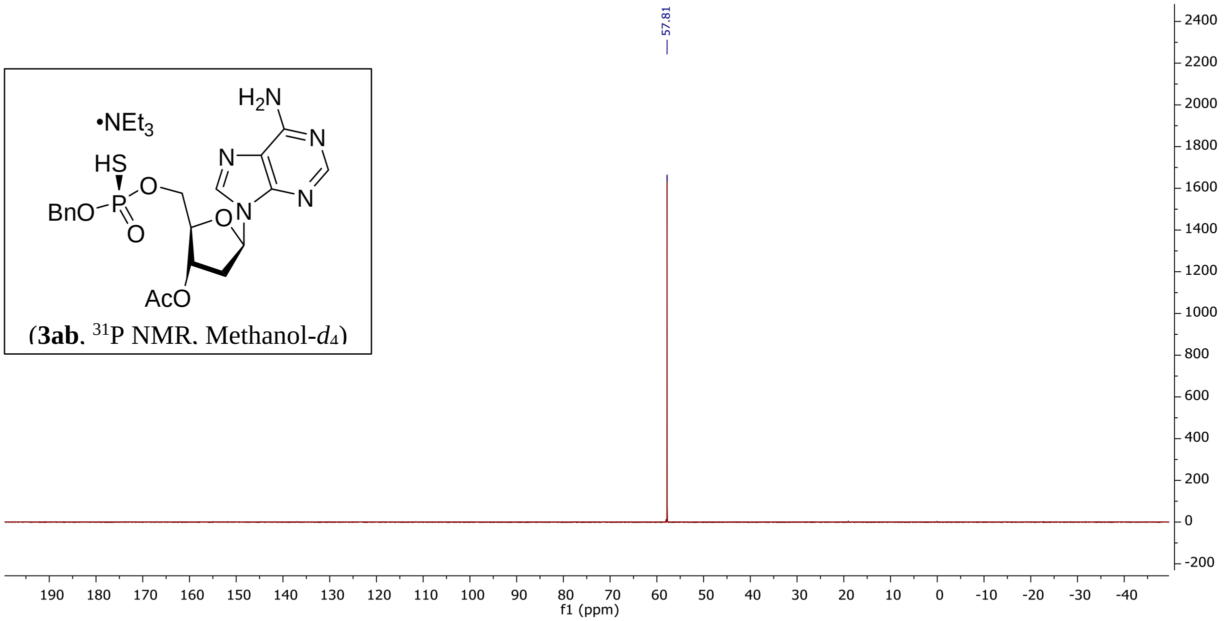

(**3ab**, ^{31}P NMR, Methanol-d_4)

_O_-(((2_R_,5_R_)-5-(6-acetamido-9_H_-purin-9-yl)-3-hydroxytetrahydrofuran-2-yl)methyl) _O_-benzyl (_S_)-phosphorothioate triethylammonium (4ab)

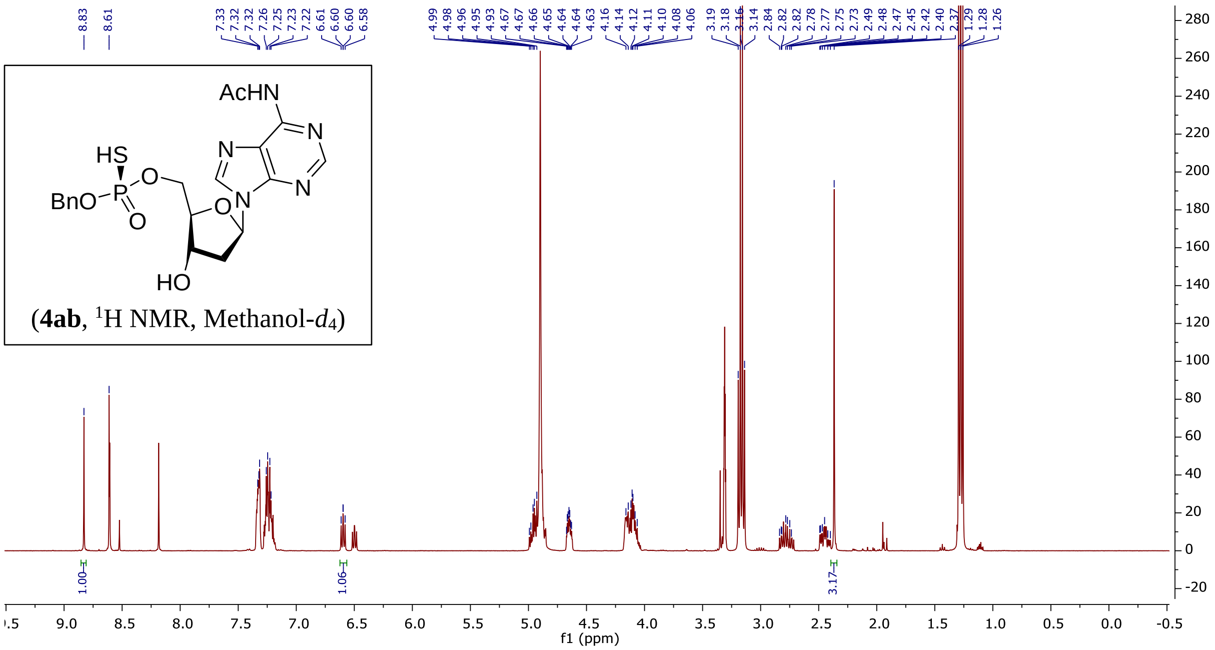

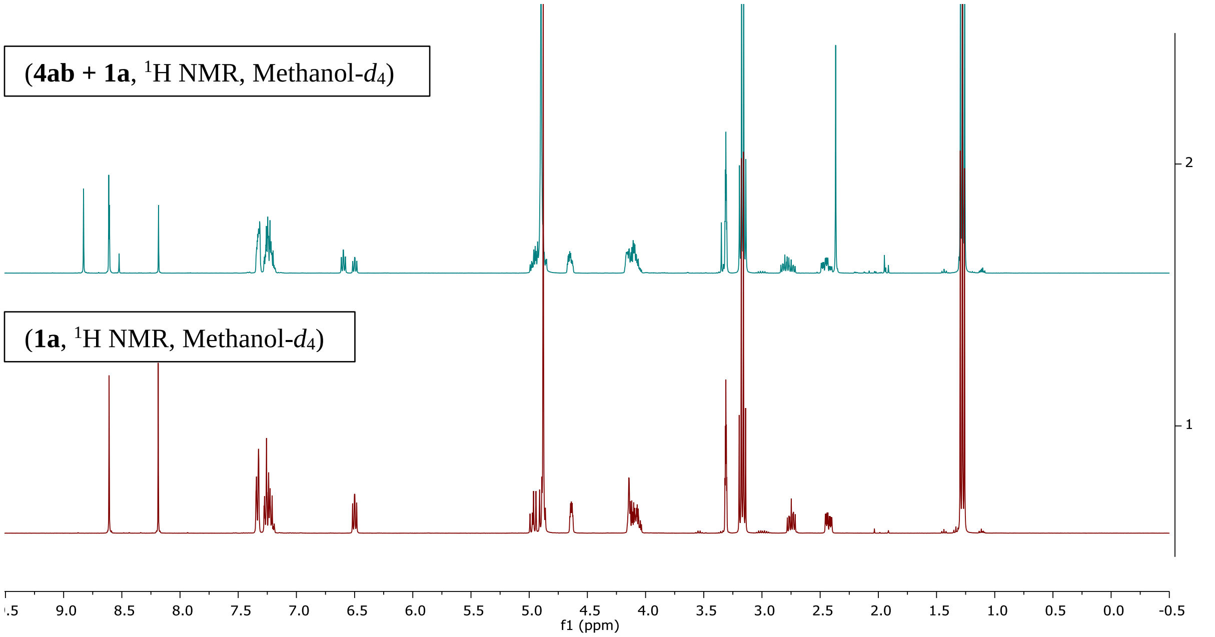

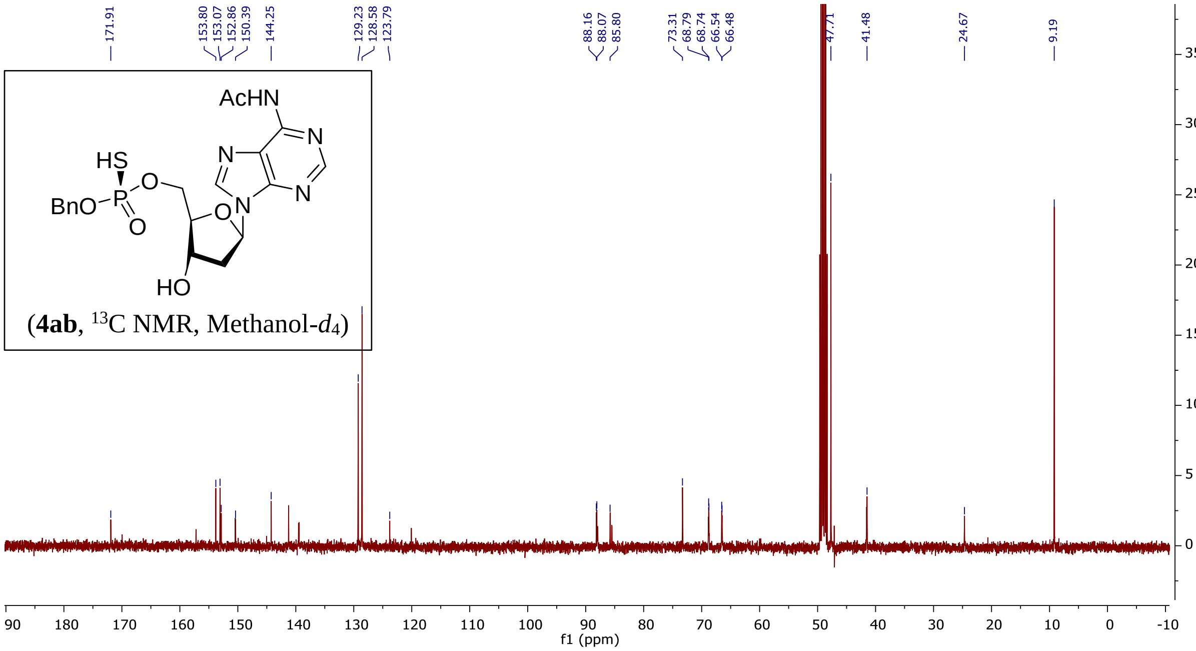

171.91
153.80
153.07
152.86
150.39
144.25
129.23
128.58
123.79
88.16
88.07
85.80
73.31
68.79
68.74
66.54
66.48
47.71
41.48
24.67
9.19
AcHN
N
N
N
N
HS
O
BnO
P
O
O
N
HO
(4ab, ^{13}C NMR, Methanol-d_4)
f1 (ppm)

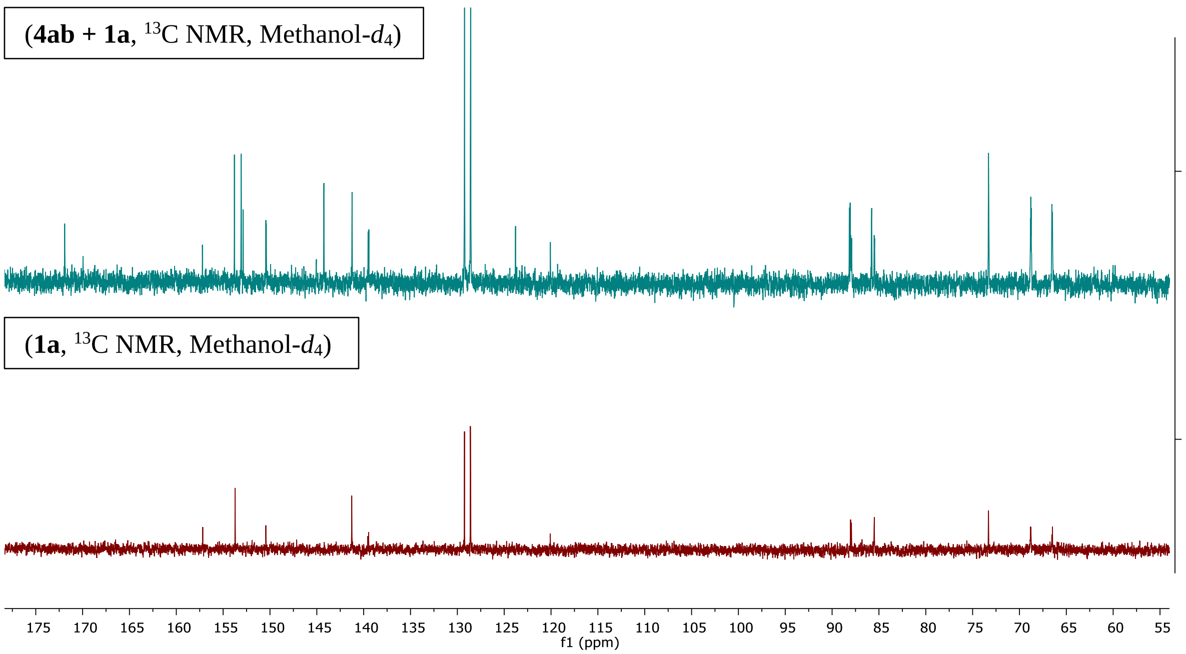

(4ab + 1a, ^{13}C NMR, Methanol-d_4)
(1a, ^{13}C NMR, Methanol-d_4)
f1 (ppm)

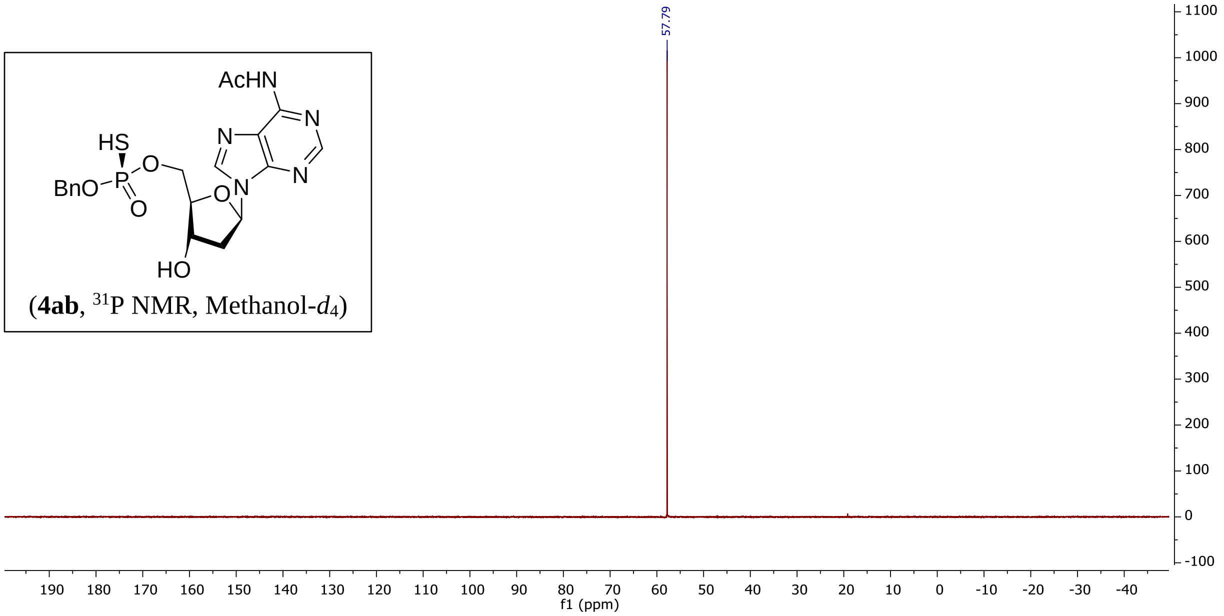

O-(((2R,5R)-5-(6-amino-9H-purin-9-yl)-3-((1-((tert-butoxycarbonyl)amino)cyclopropane-1-carbonyl)oxy)tetrahydrofuran-2-yl)methyl) O-benzyl (S)-phosphorothioate triethylammonium (3af)

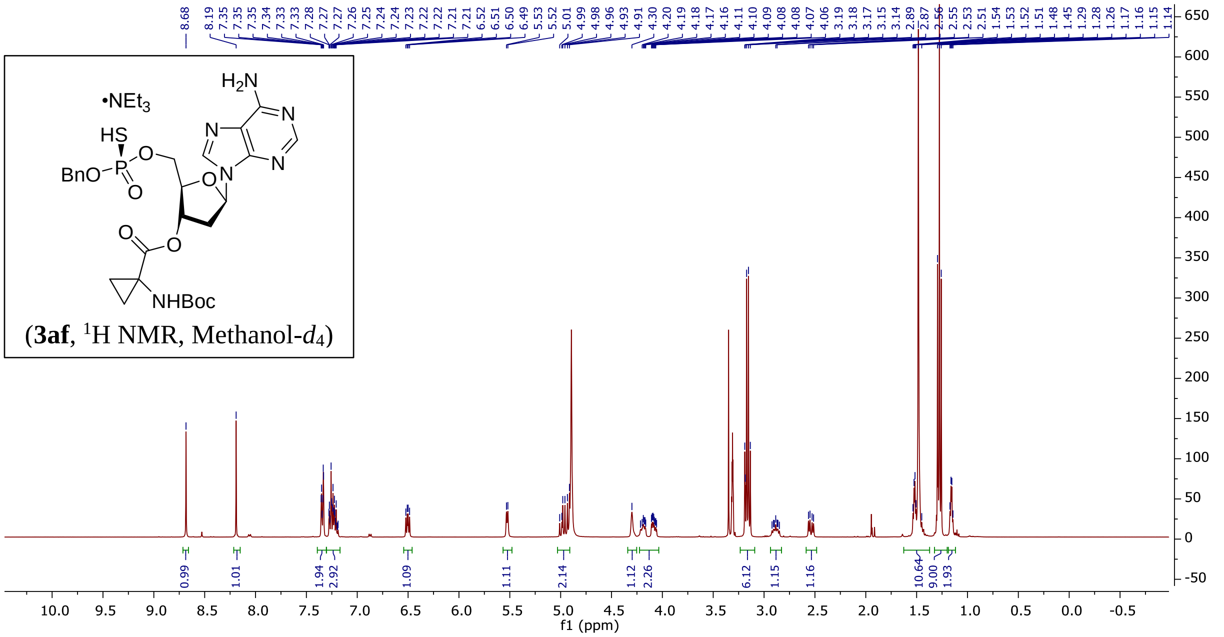

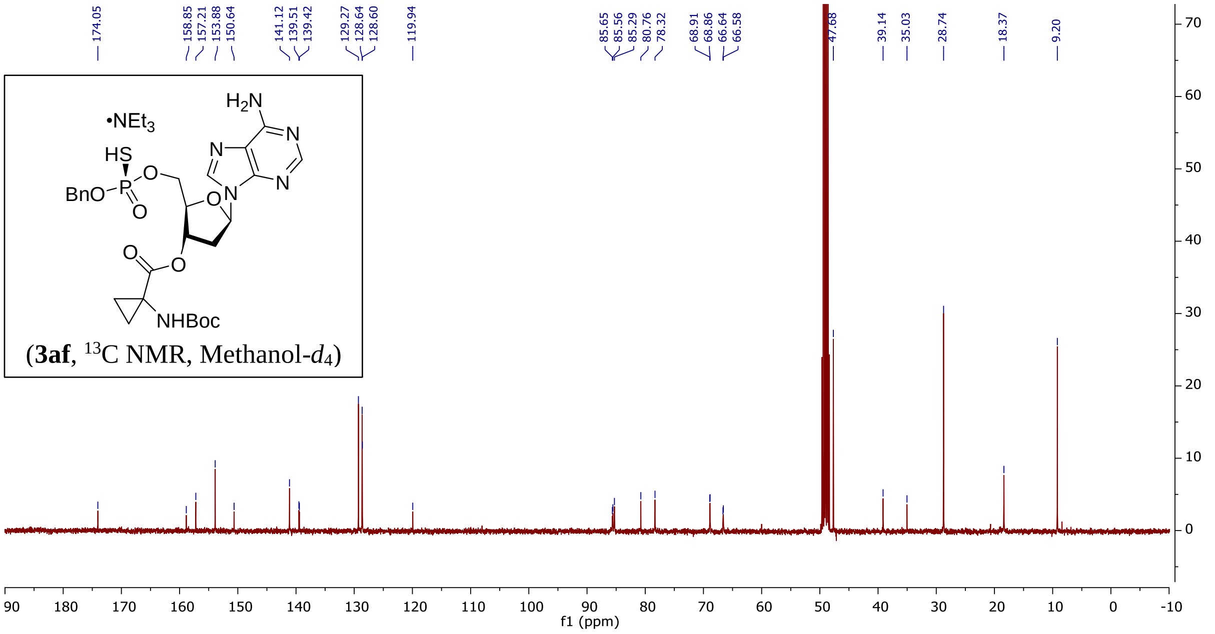

(**3af**, ^{13}C NMR, Methanol-d_4)

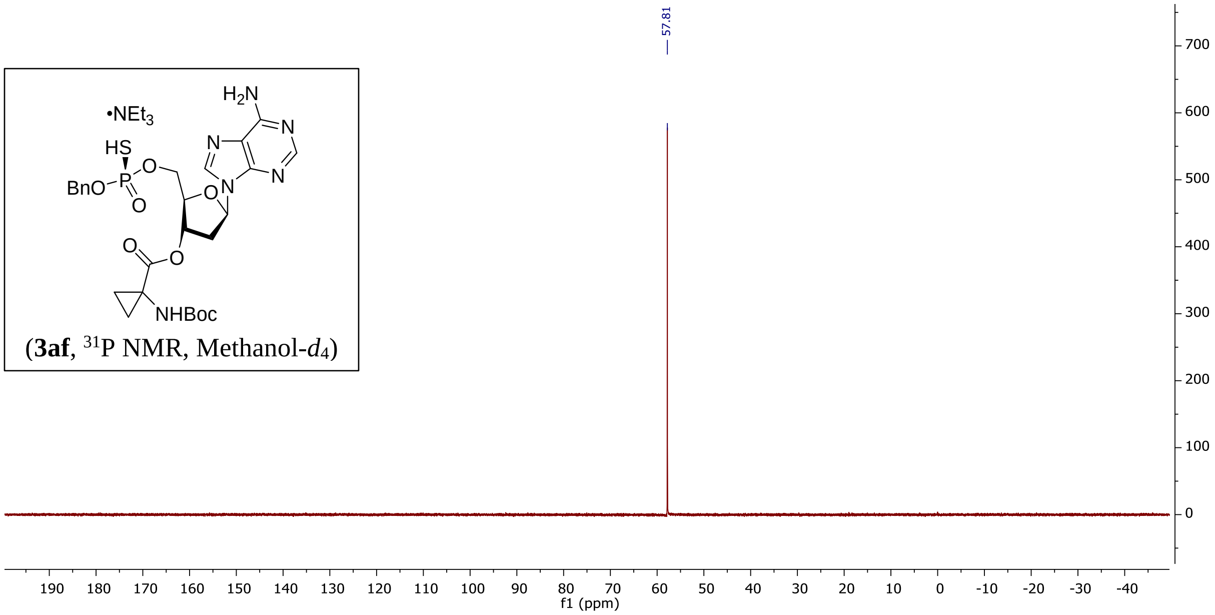

(**3af**, ^{31}P NMR, Methanol-d_4)

_O_-benzyl _O_-(((2_R_,5_R_)-5-(6-(1-((_tert_-butoxycarbonyl)amino)cyclopropane-1-carboxamido)-9_H_-purin-9-yl)-3-hydroxytetrahydrofuran-2-yl)methyl) (_S_)-phosphorothioate triethylammonium (4af)

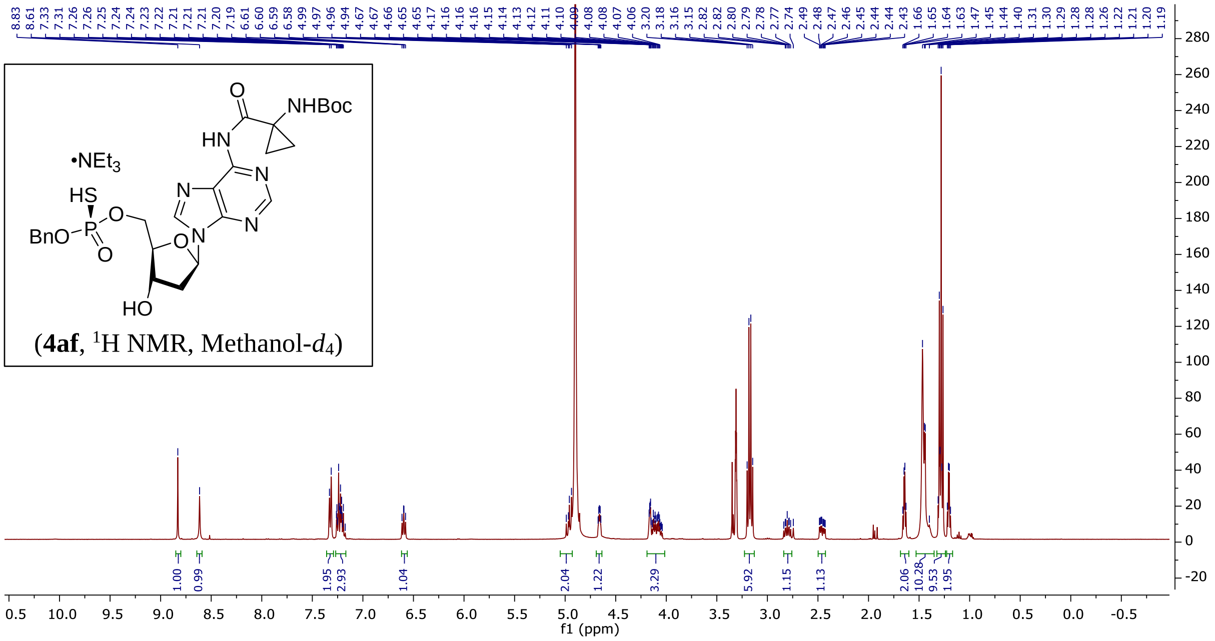

(**4af**, ^{1}H NMR, Methanol-d_4)

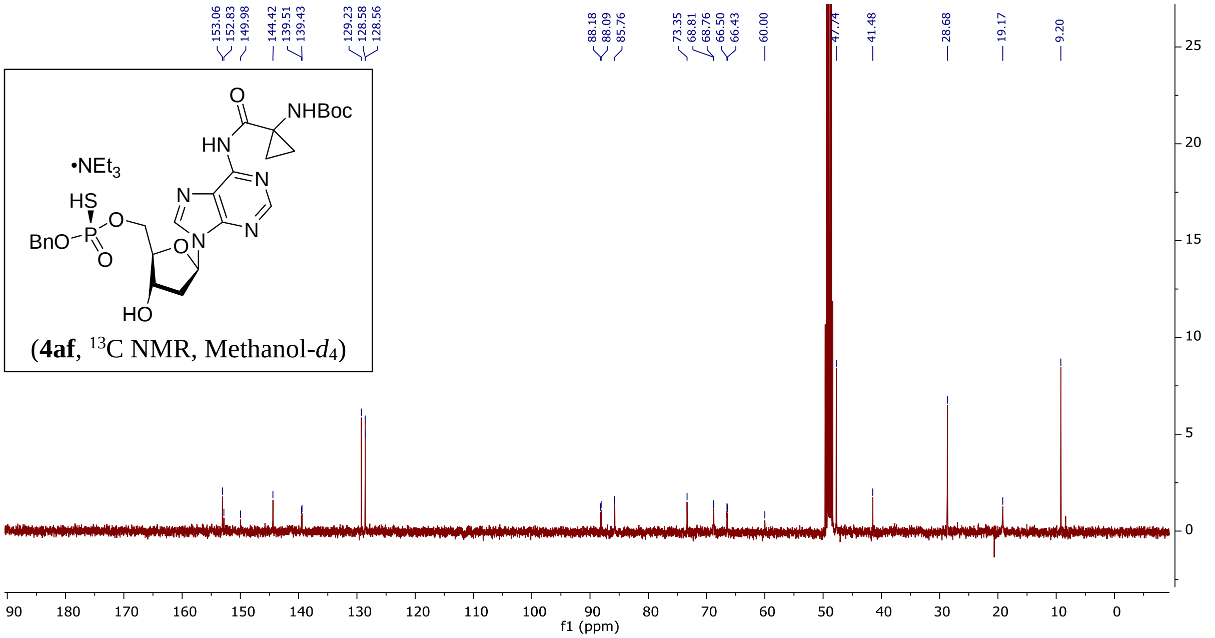

(**4af**, ^{13}C NMR, Methanol-d_4)

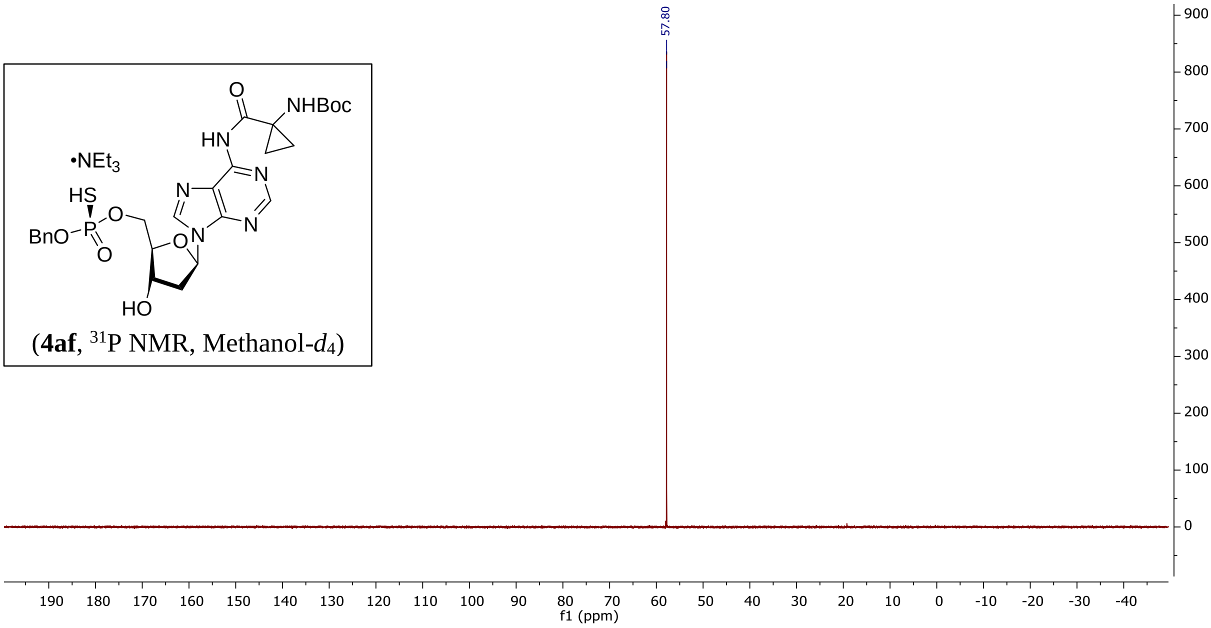

($\mathbf{4af}$, ^{31}P NMR, Methanol-d_4)

O-(((2R,5R)-5-(6-amino-9H-purin-9-yl)-3-(((tert-butoxycarbonyl)-L-phenylalanyl)oxy)tetrahydrofuran-2-yl)methyl) O-benzyl (S)-phosphorothioate triethylammonium (3ag)

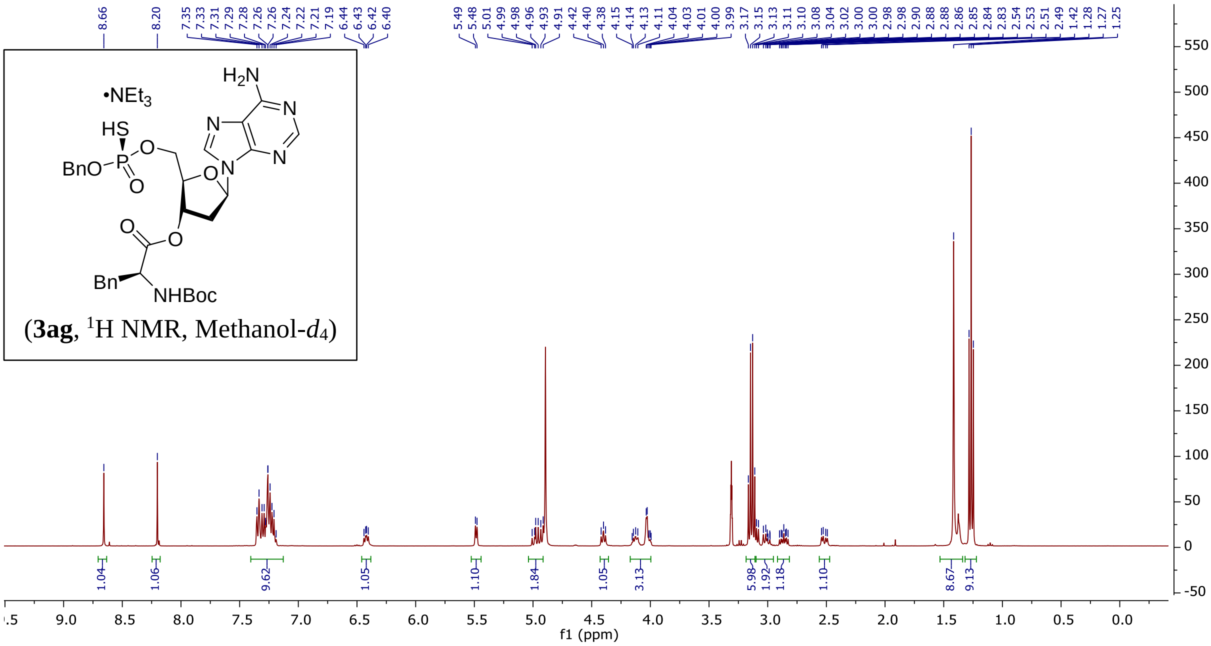

($\mathbf{3ag}$, ^{1}H NMR, Methanol-d_4)

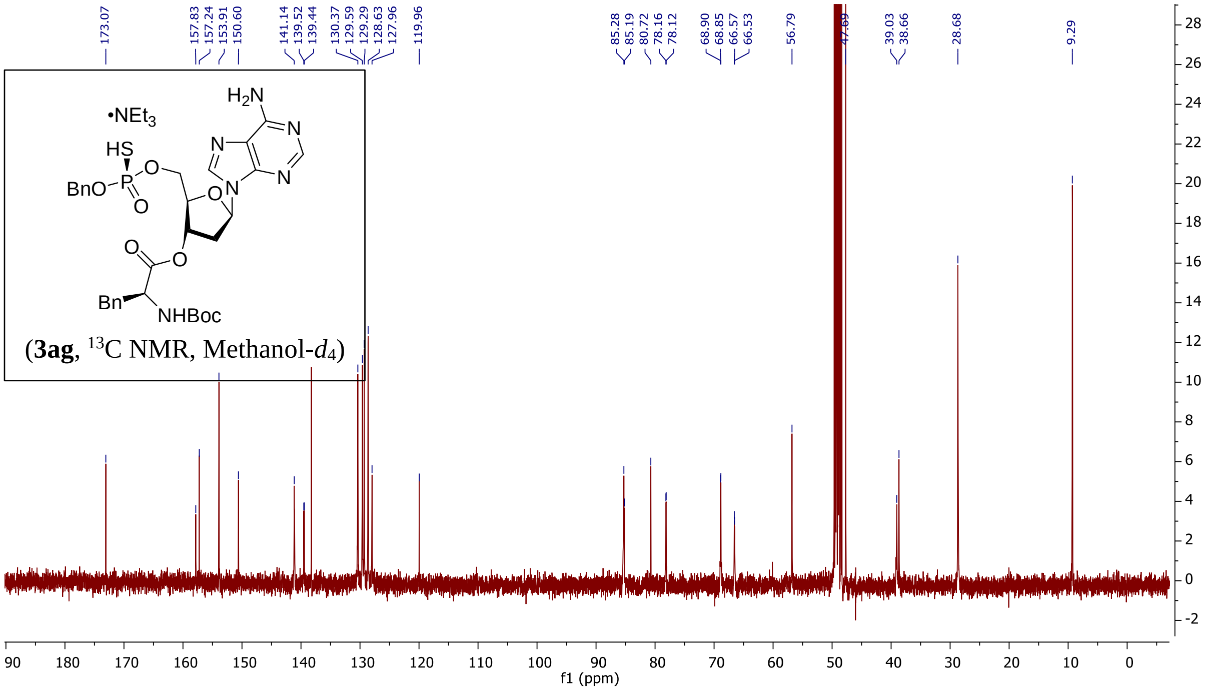

(**3ag**, ^{13}C NMR, Methanol-d_4)

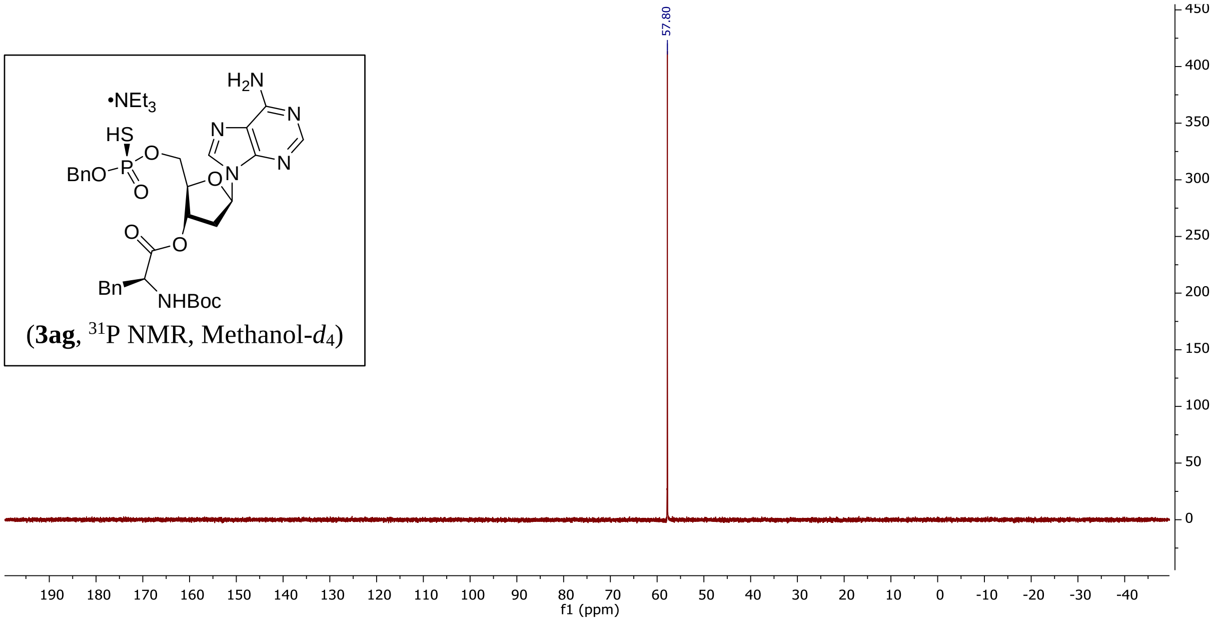

(**3ag**, ^{31}P NMR, Methanol-d_4)

O-benzyl O-(((2R,5R)-5-(6-((S)-2-((tert-butoxycarbonyl)amino)-3-phenylpropanamido)-9H-purin-9-yl)-3-hydroxytetrahydrofuran-2-yl)methyl) (S)-phosphorothioate triethylammonium (4ag)

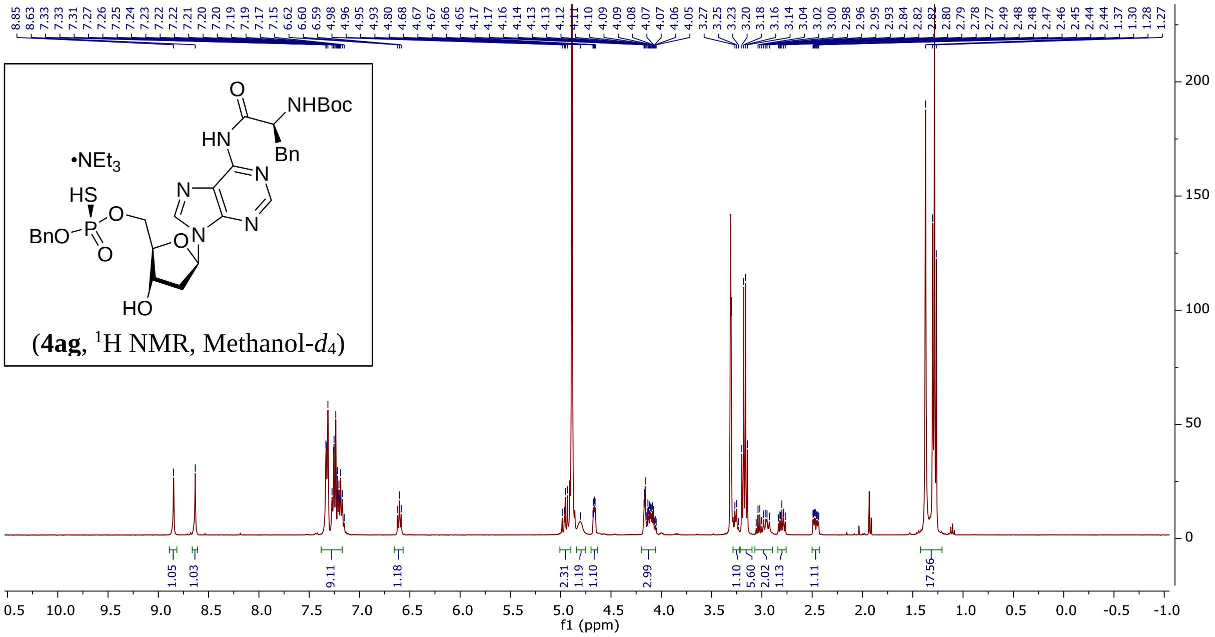

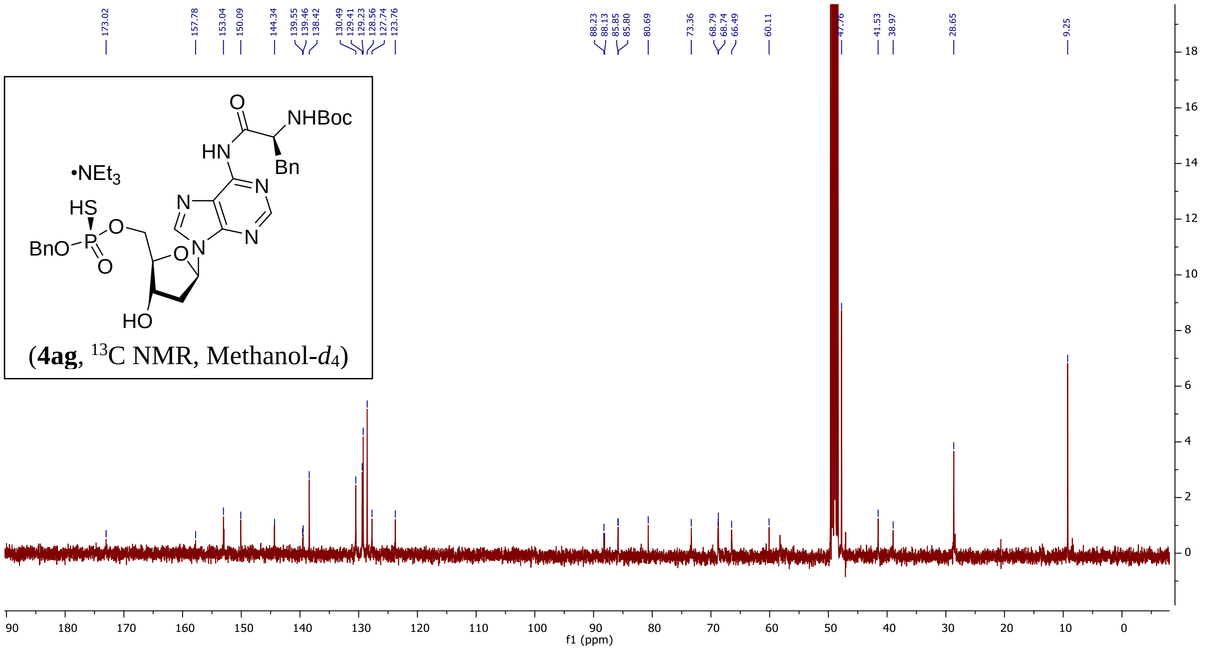

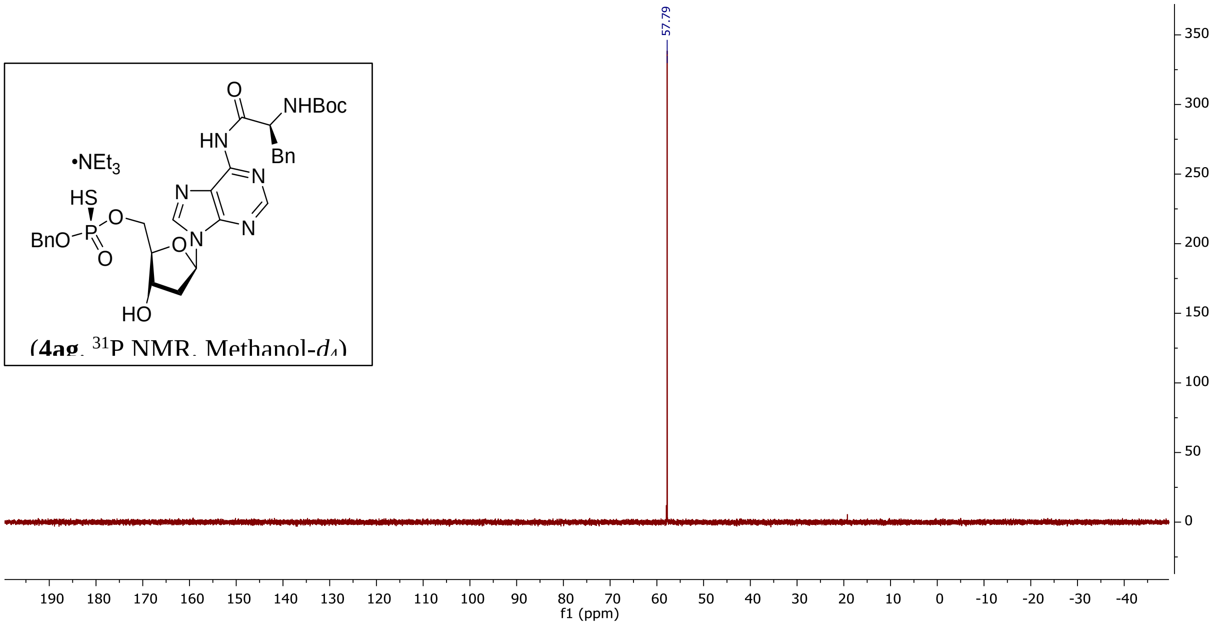

(**4ag**, ^{31}P NMR, Methanol-d_4)

(2R,5R)-5-(6-amino-9H-purin-9-yl)-2-((((R)-(benzyloxy)(mercapto)phosphoryl)oxy)methyl)tetrahydrofuran-3-yl 3-((9S,12S,15S)-9-((tert-butoxycarbonyl)amino)-15-isobutyl-12-isopropyl-3,10,13-trioxo-1-phenyl-2-oxa-4,11,14-triazahexadecan-16-amido)benzoate triethylammonium (3ah)

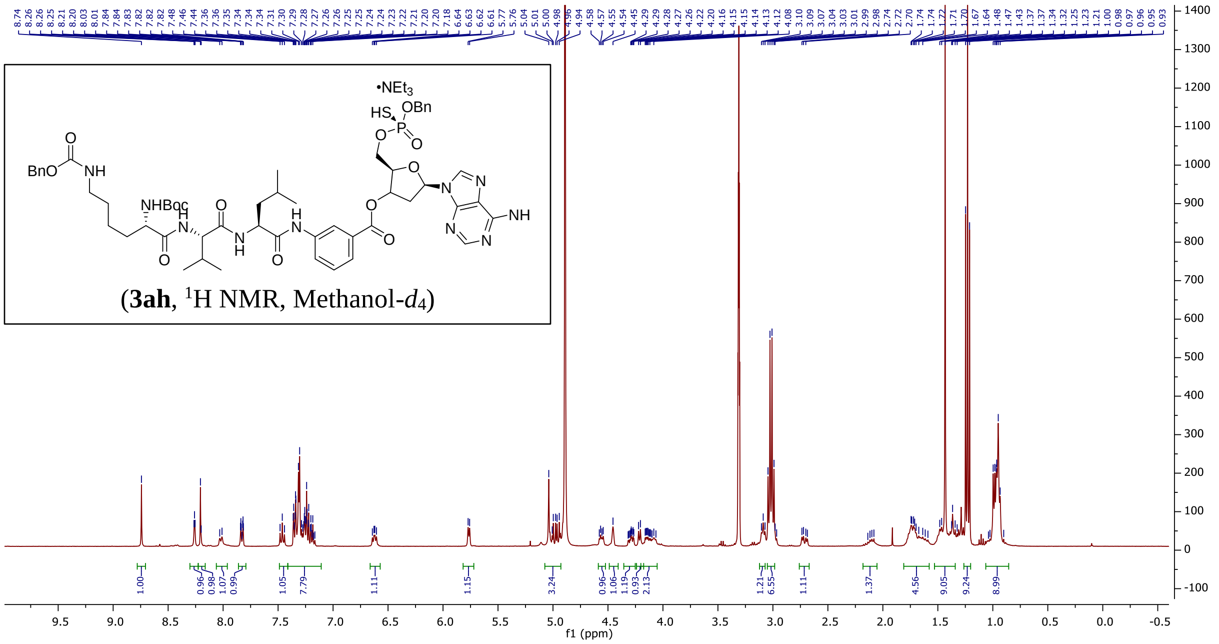

(**3ah**, ^{1}H NMR, Methanol-d_4)

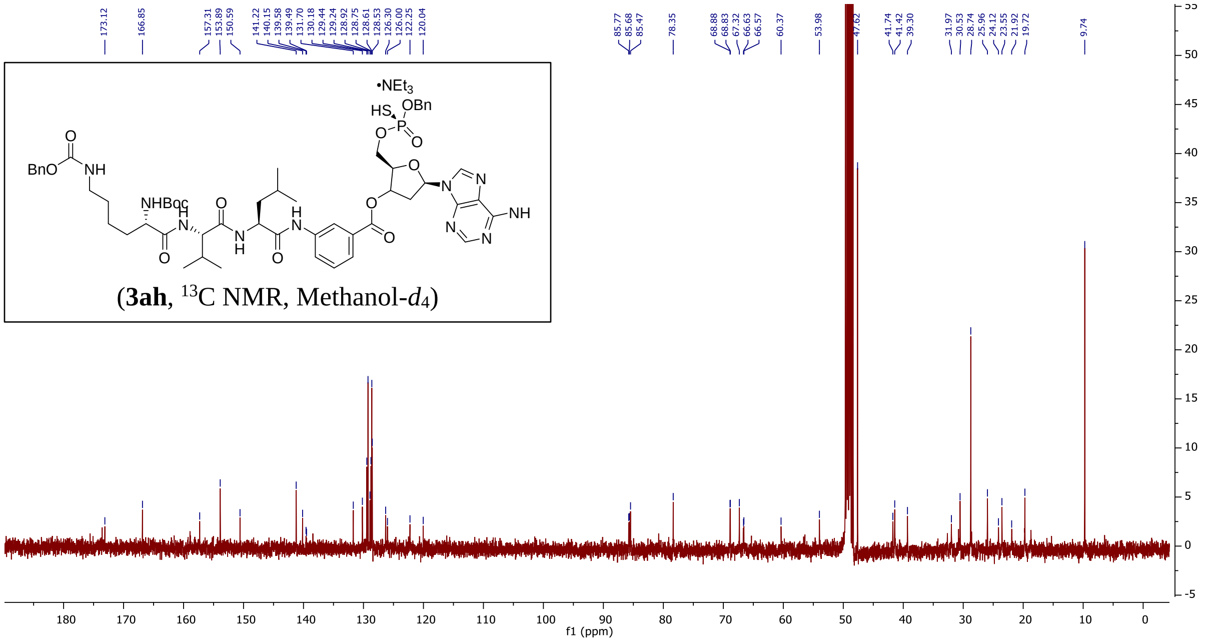
(3ah, 13C NMR, Methanol-d4)
f1 (ppm)

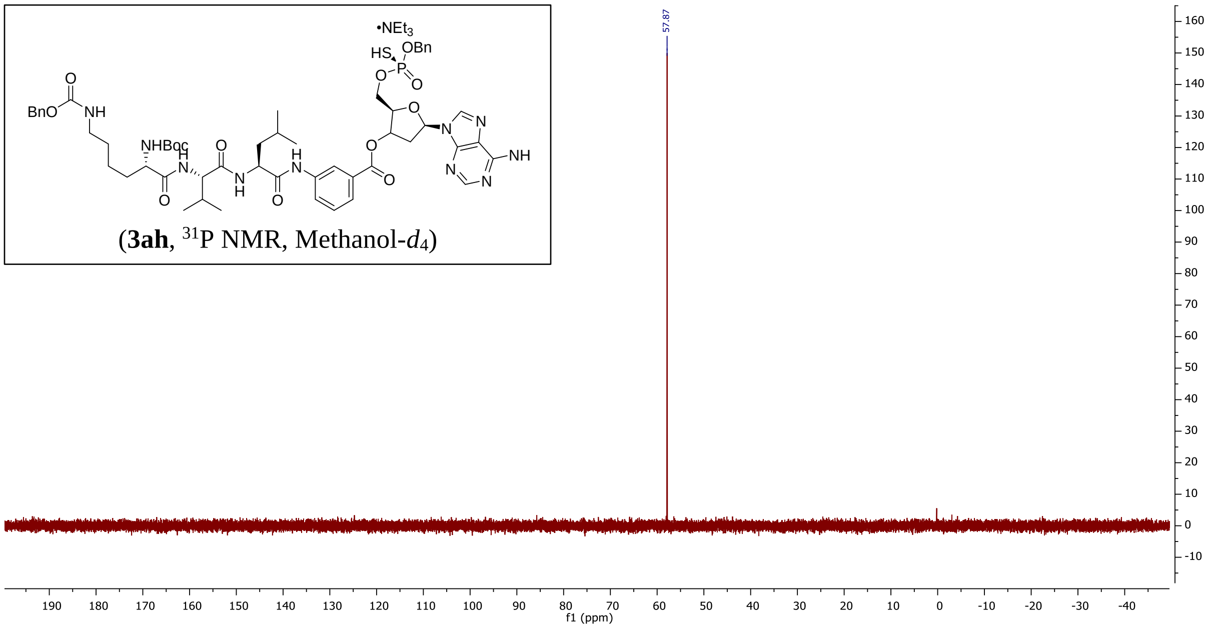
(3ah, 31P NMR, Methanol-d4)
57.87
f1 (ppm)

benzyl tert-butyl ((5S)-6-(((2S)-1-(((2S)-1-((3-((9-((2R,5R)-5-((((R)-(benzyloxy)(mercapto)phosphoryl)oxy)methyl)-4-hydroxytetrahydrofuran-2-yl)-9H-purin-6-yl)carbamoyl)phenyl)amino)-4-methyl-1-oxopentan-2-yl)amino)-3-methyl-1-oxobutan-2-yl)amino)-6-oxohexane-1,5-diyl)dicarbamate triethylammonium (4ah)

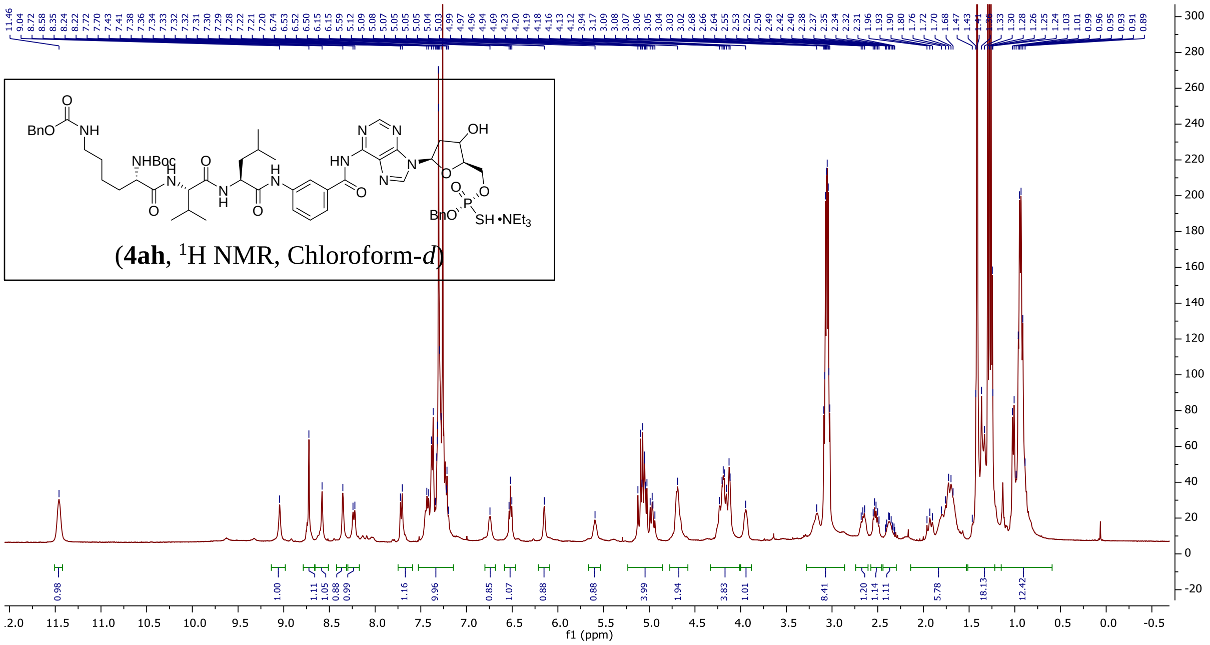

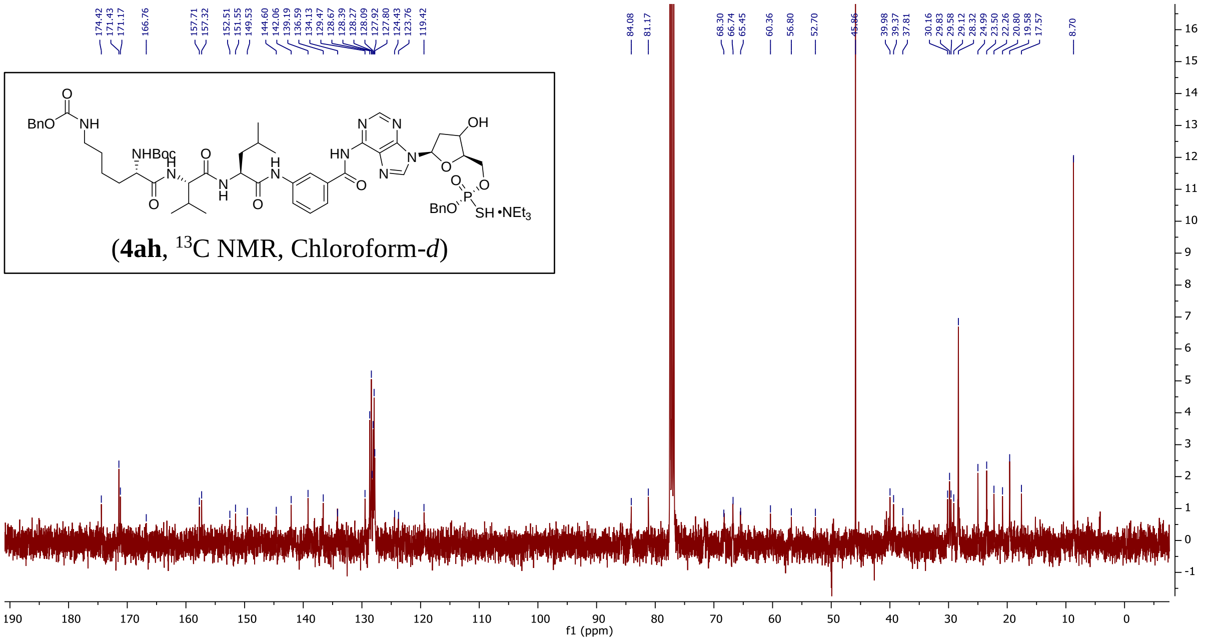

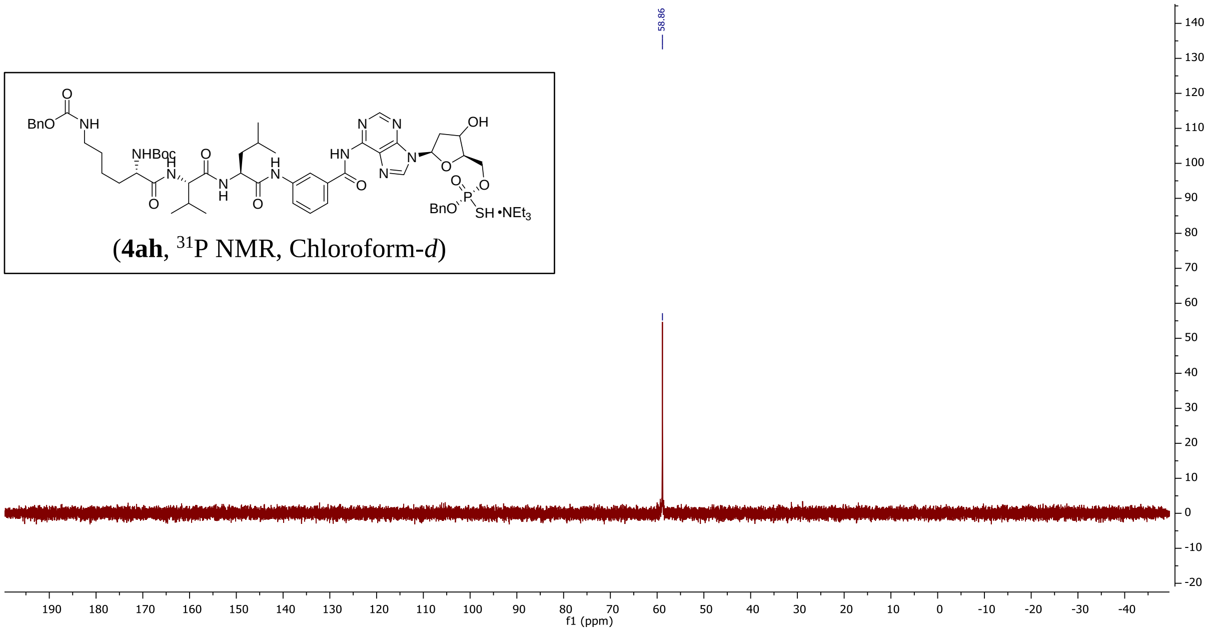

(**4ah**, ^{31}P NMR, Chloroform-*d*)

6.2 Crude ^{1}H NMR spectra

3a (condition A, 3a:4a > 99:1)

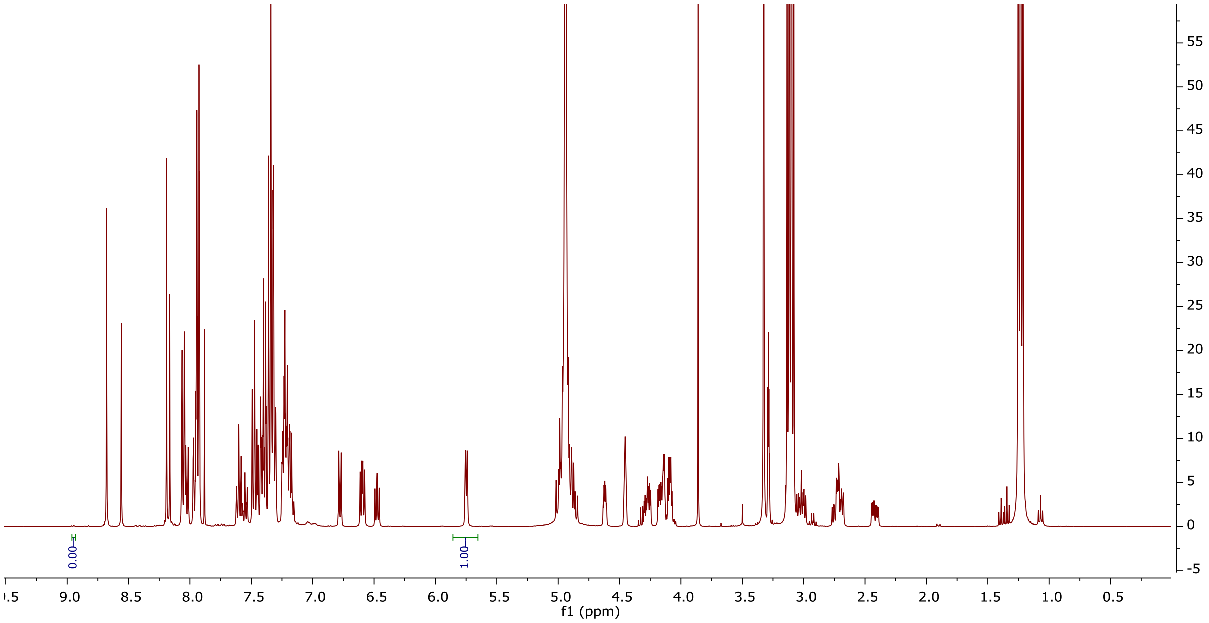

4a (condition B, 3a:4a > 2:98)

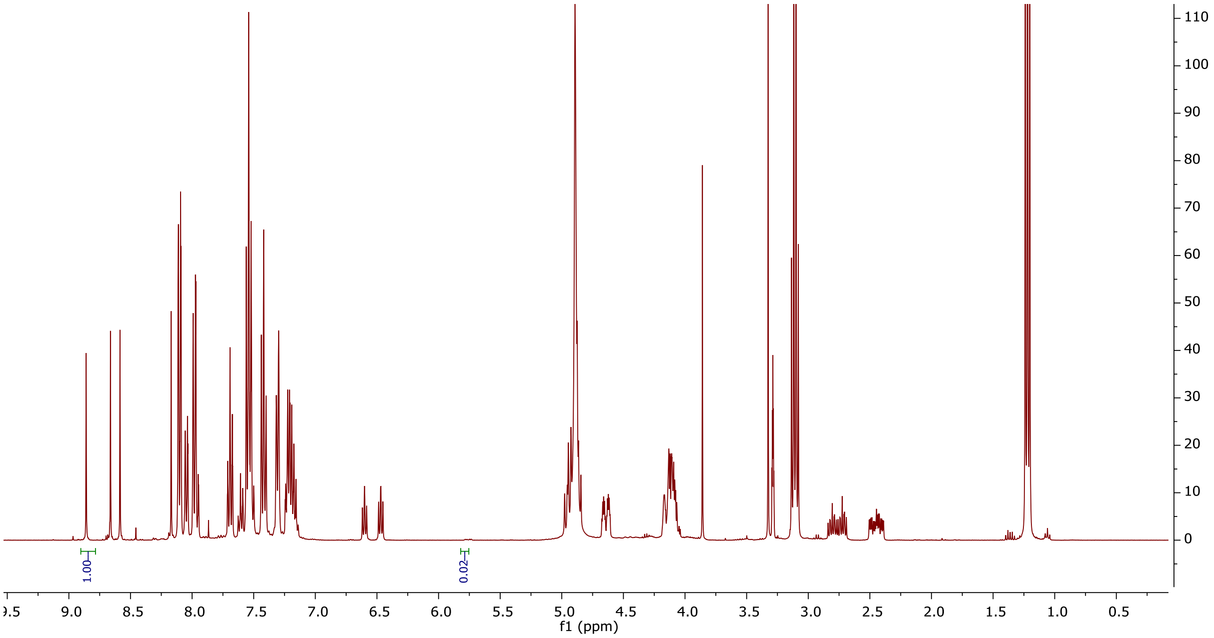

3b (condition B, 3b:4b = 99:1)

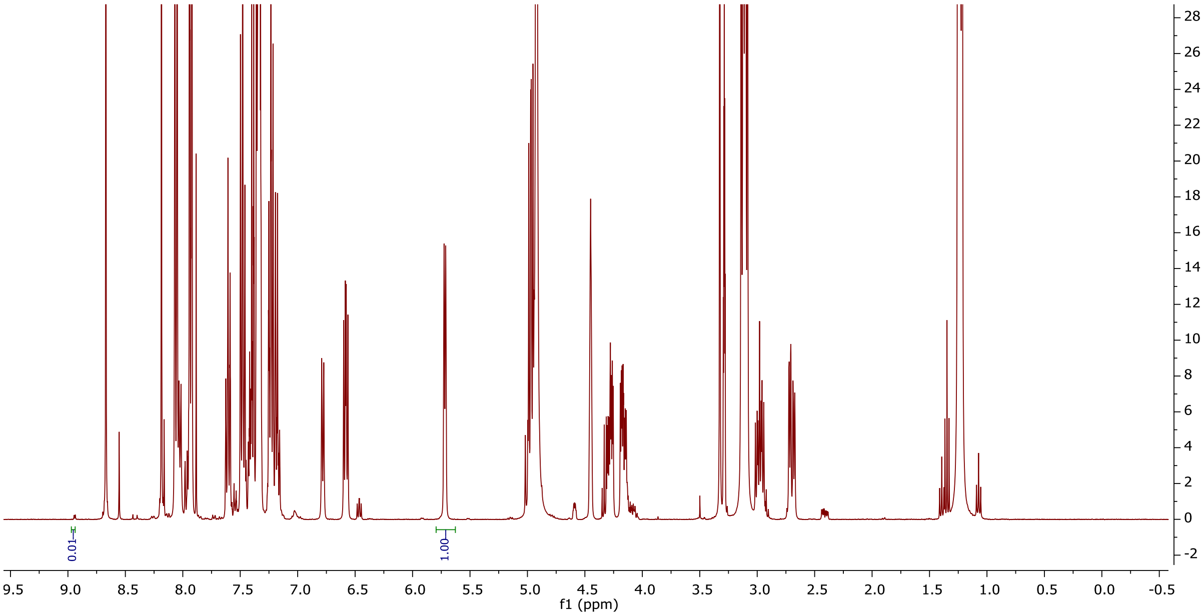

4b (condition B, 3b:4b = 3:97)

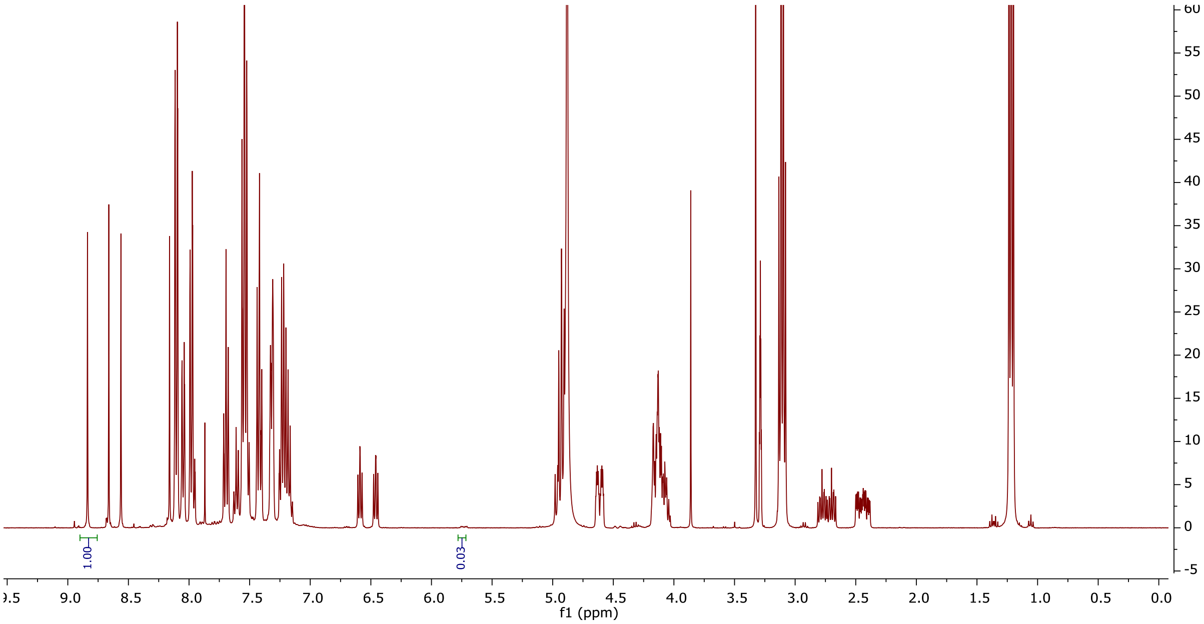

3c (condition A, 3c:4c = 99:1)

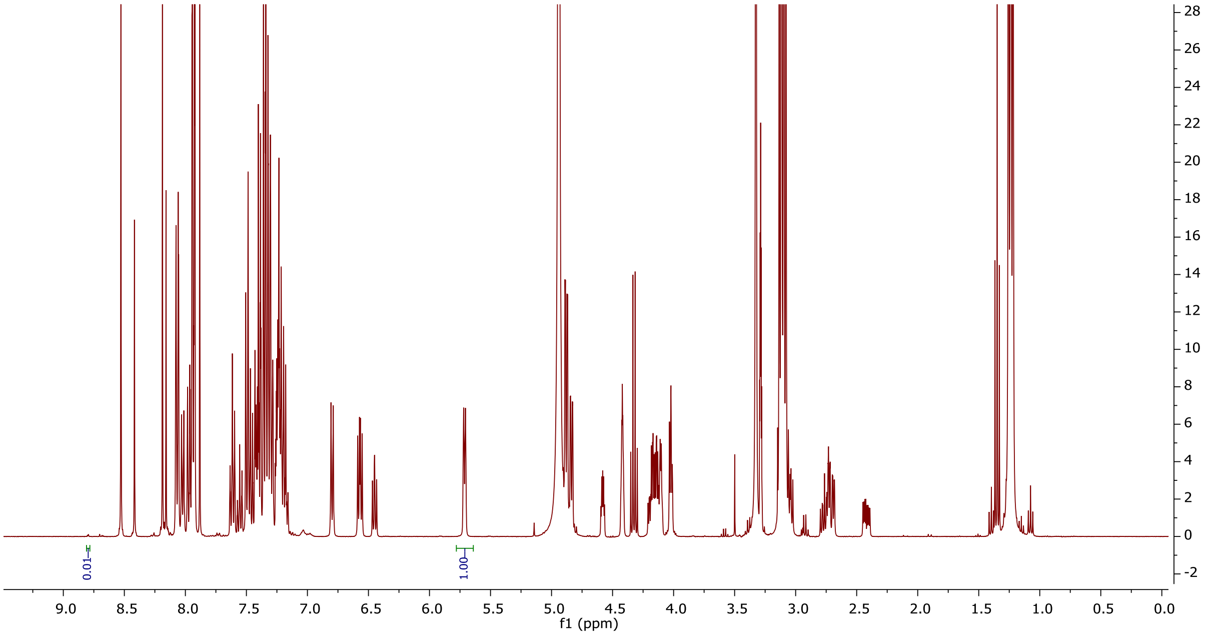

4c (condition B, 3c:4c = 4:96)

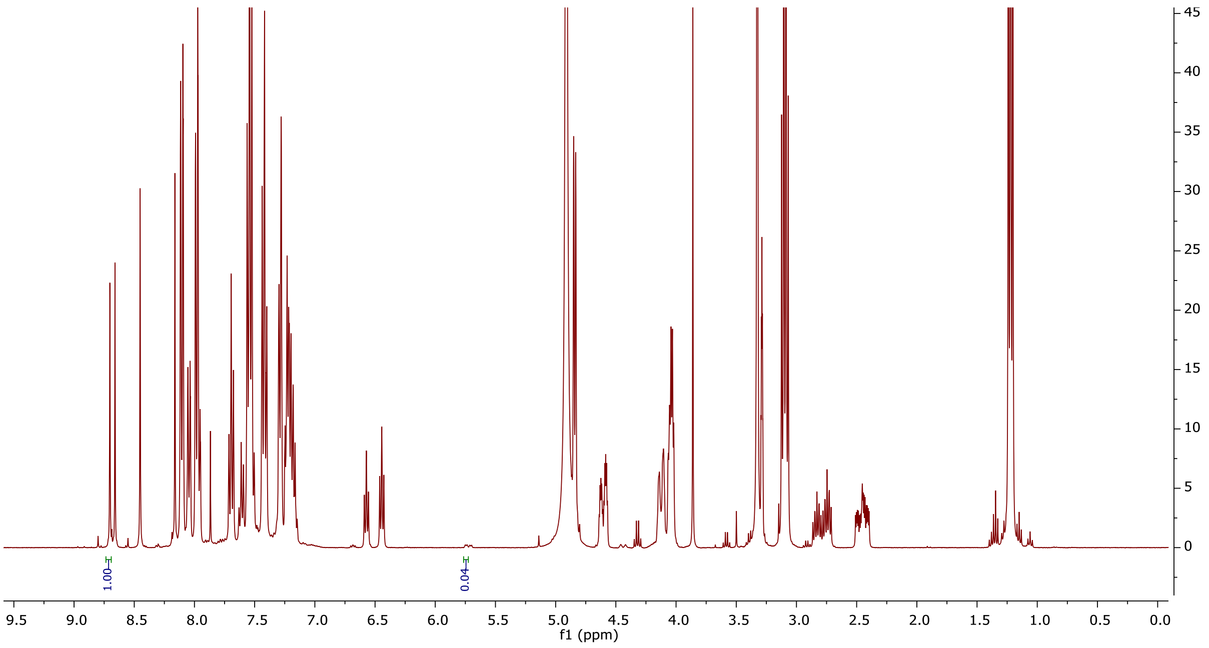

3d (condition A, 3d:4d > 99:1)

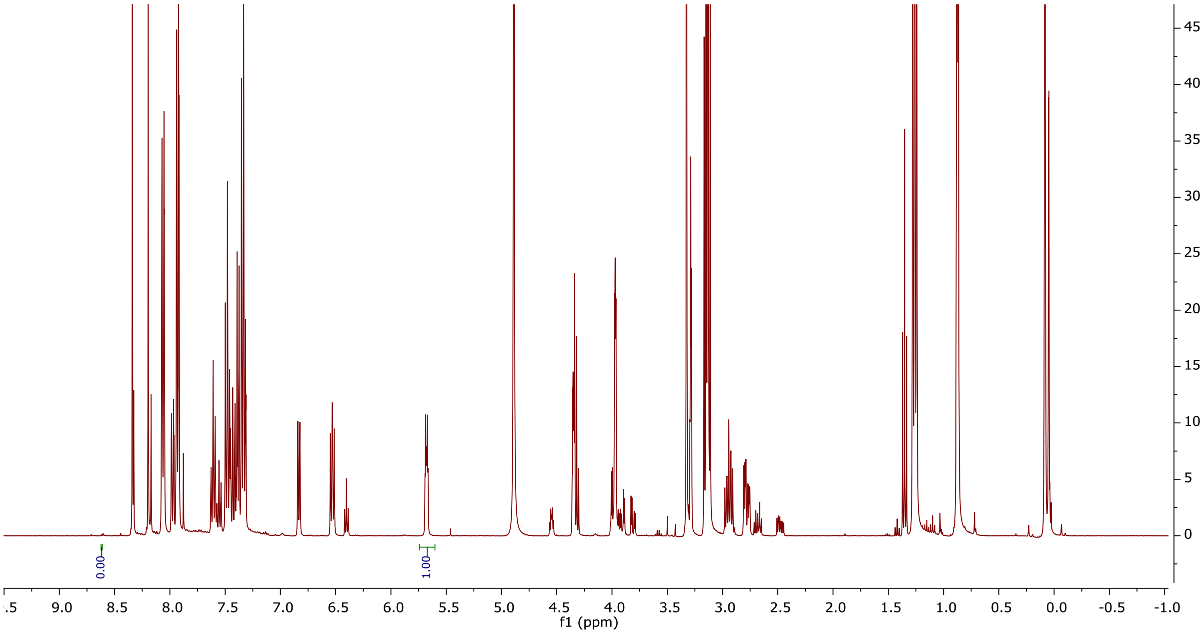

4d (condition B, 3d:4d = 4:96)

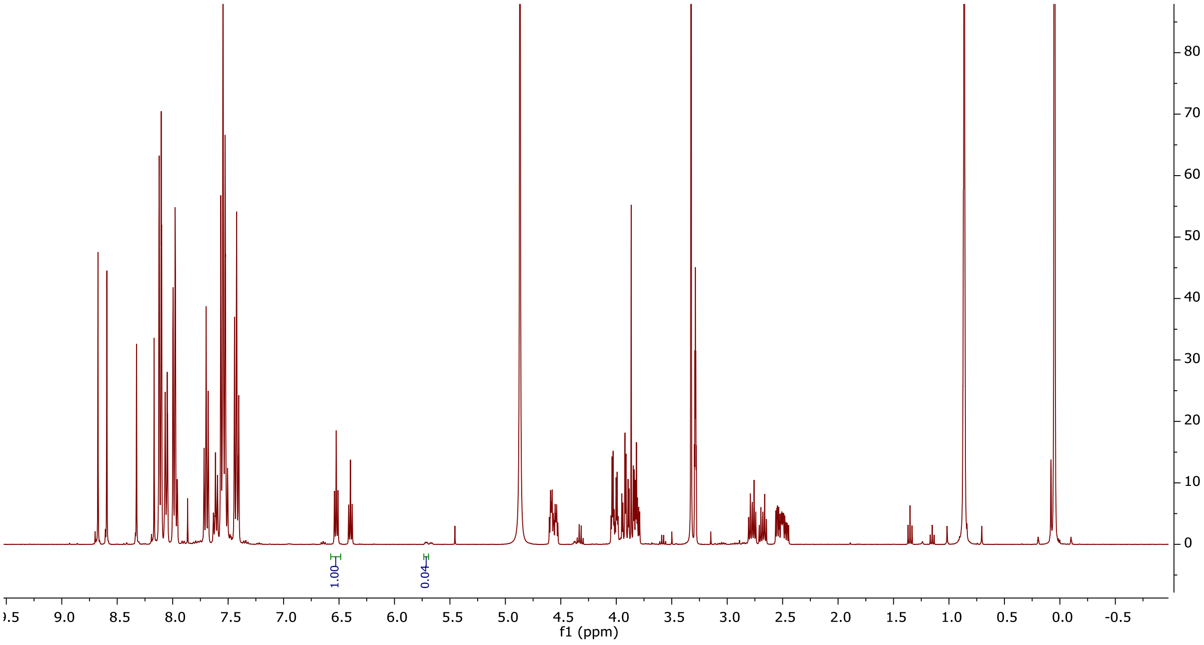

3e (condition A, 3e:4e = 99:1)

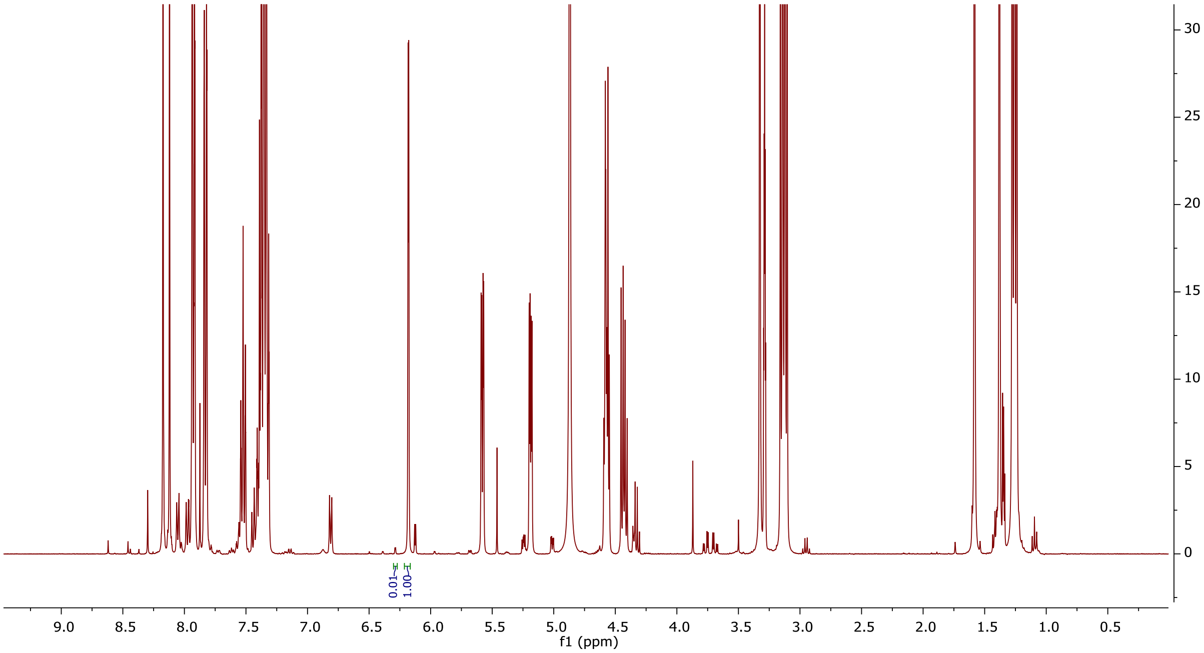

4e (condition B, 3e:4e = 8:92)

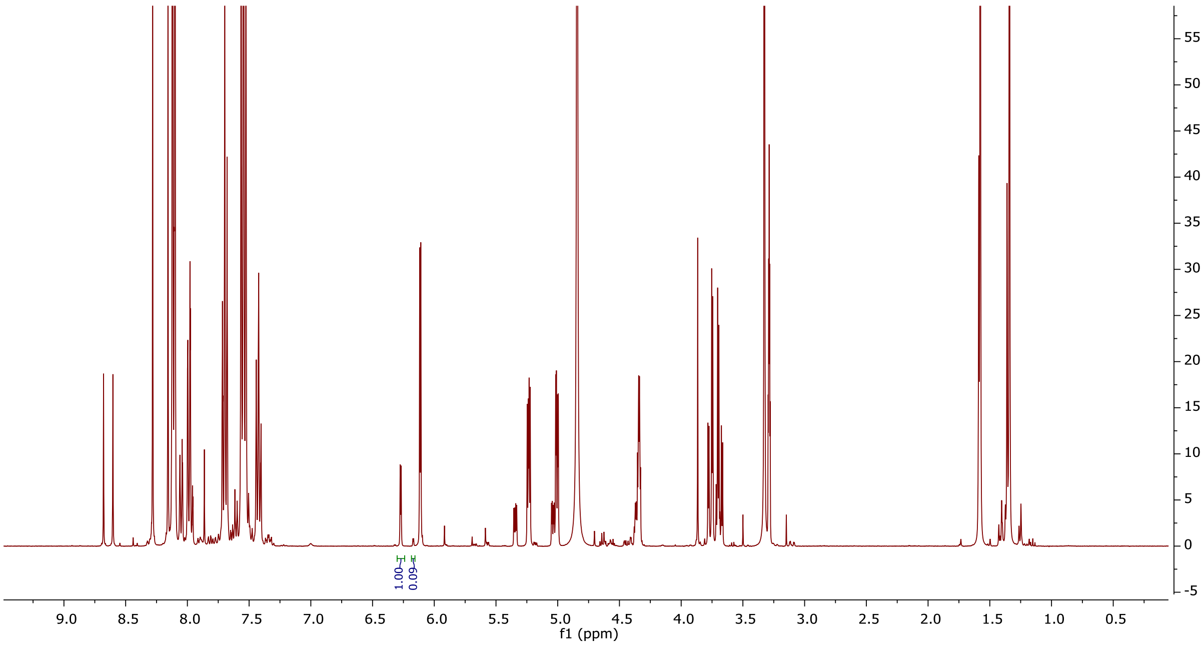

3f (condition A, 3f:4f > 99:1)

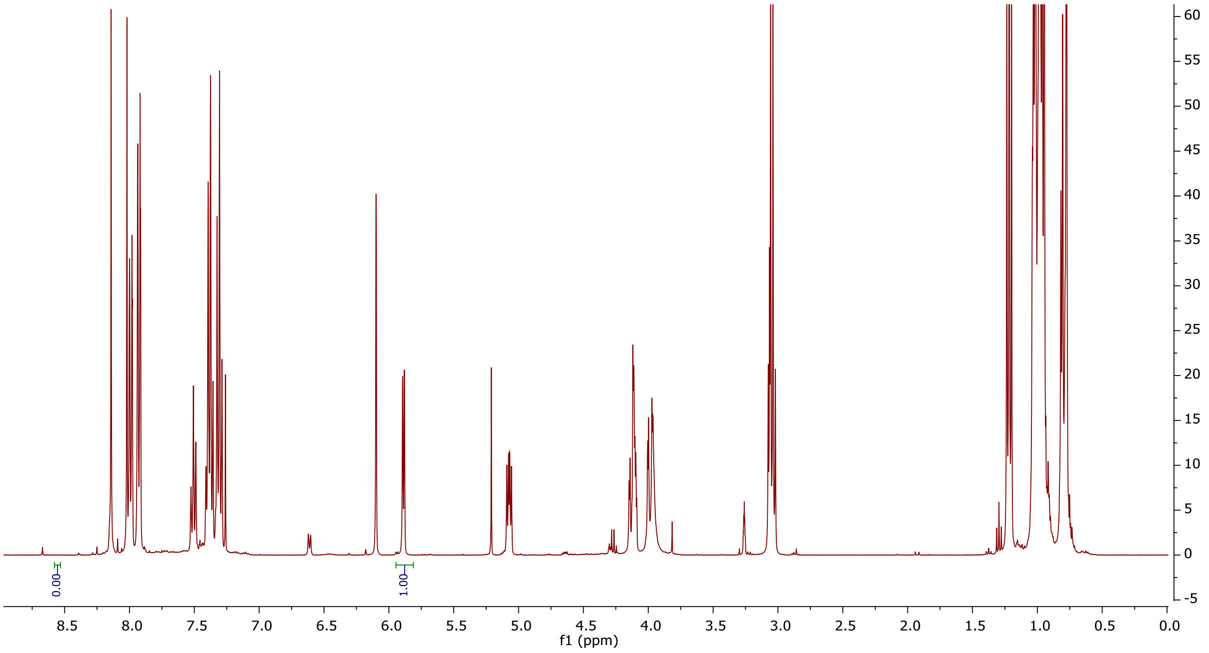

4f (condition B, 3f:4f = 2:98)

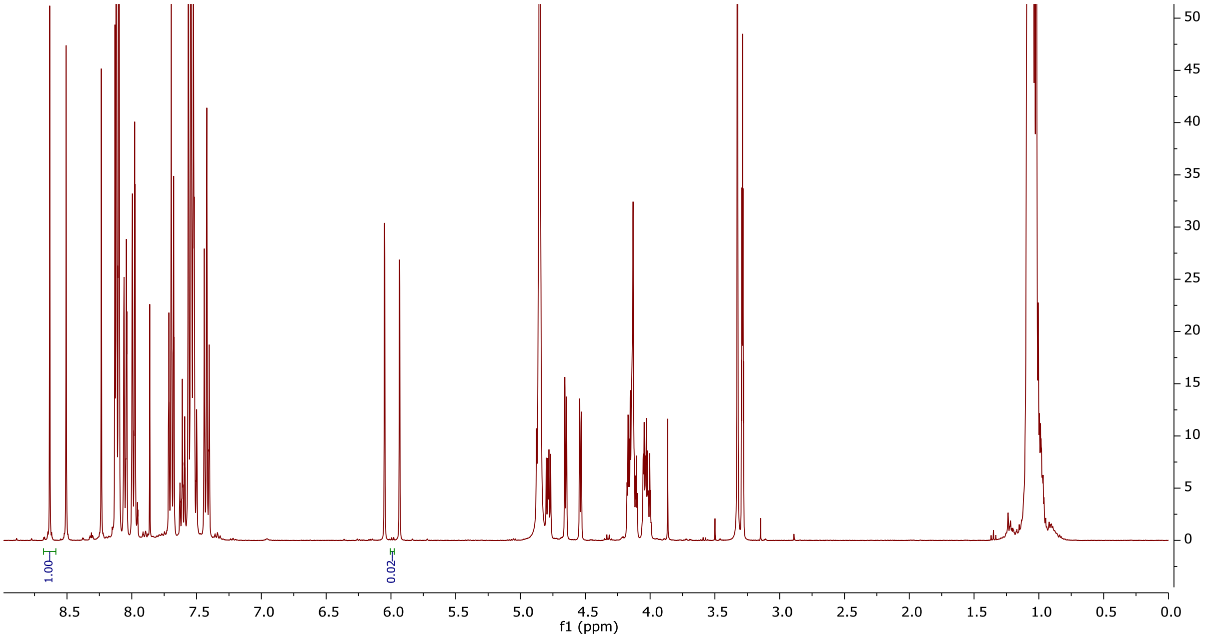

3g (condition A, spectra of the mixture of 3g and 4g after flash column, 3g:4g = 87:13)

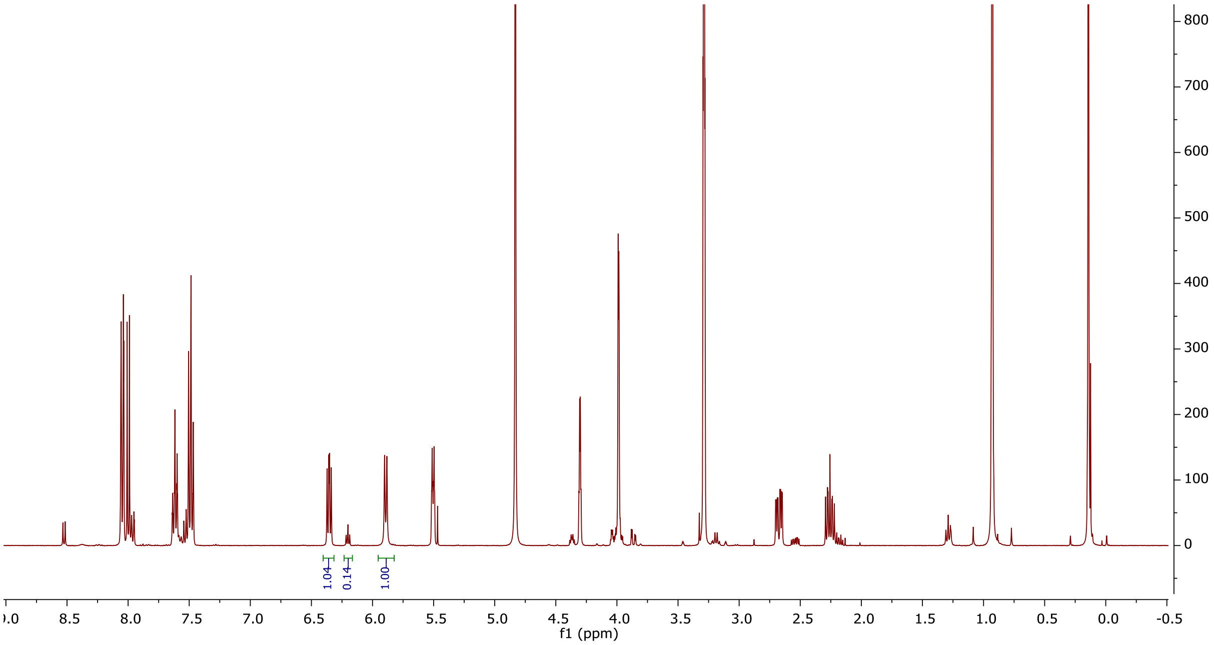

4g (condition B, 3g:4g = 1:99)

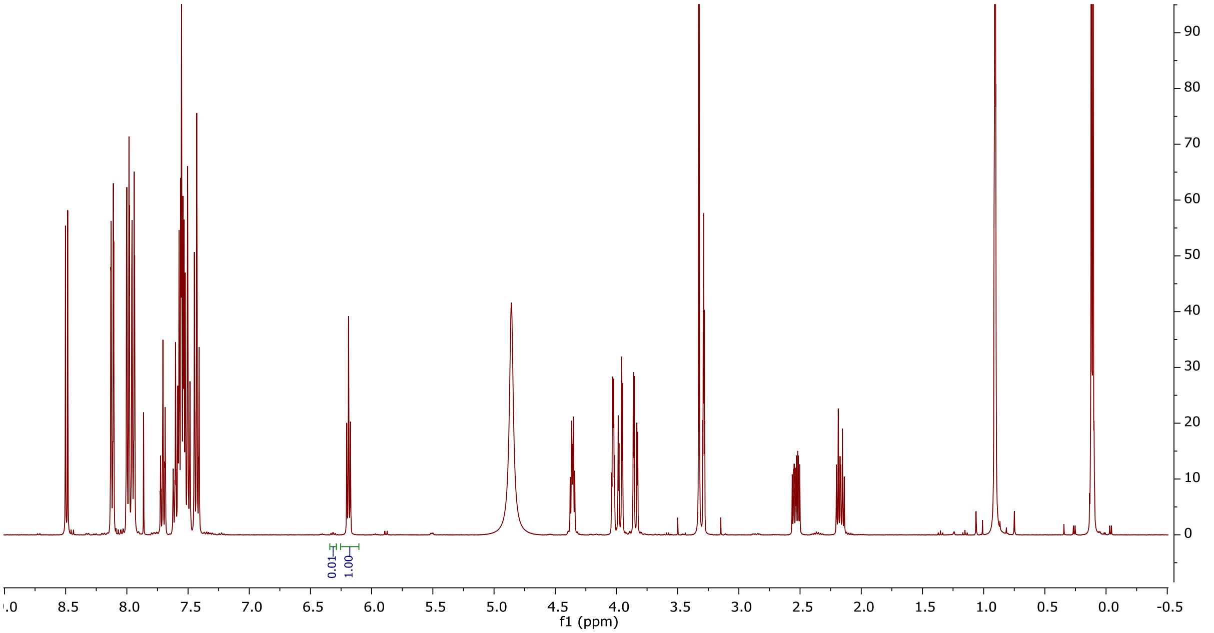

3h (condition A, 3h:4h > 99:1)

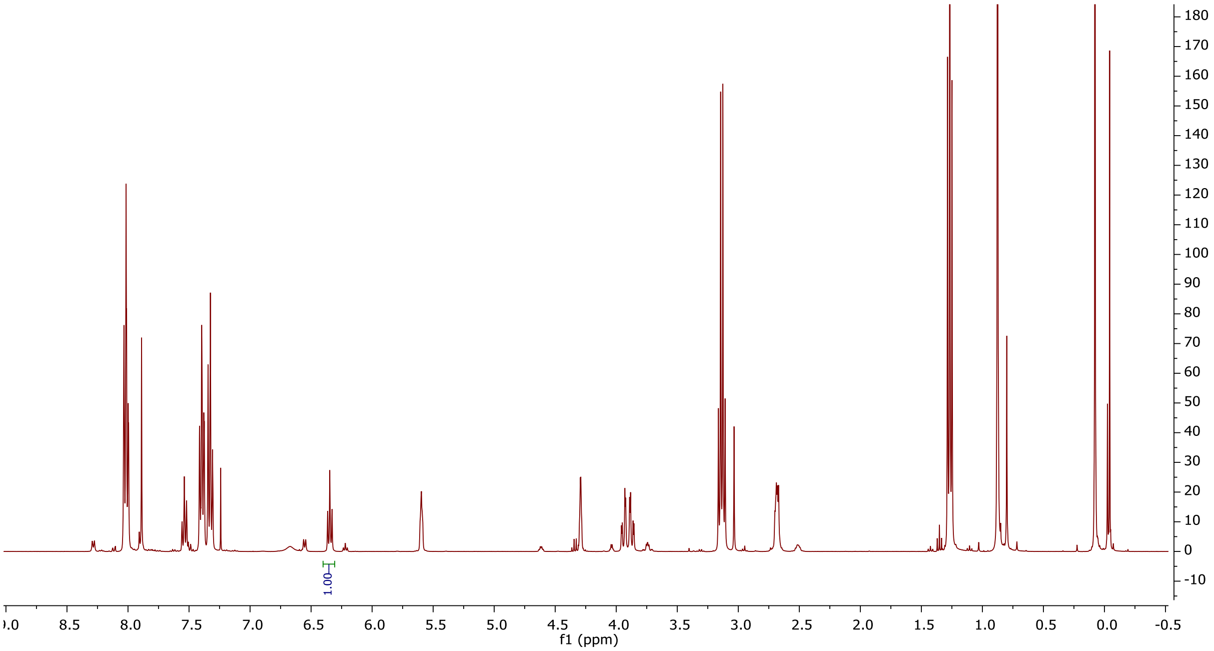

4h (condition B, *N. D.*)

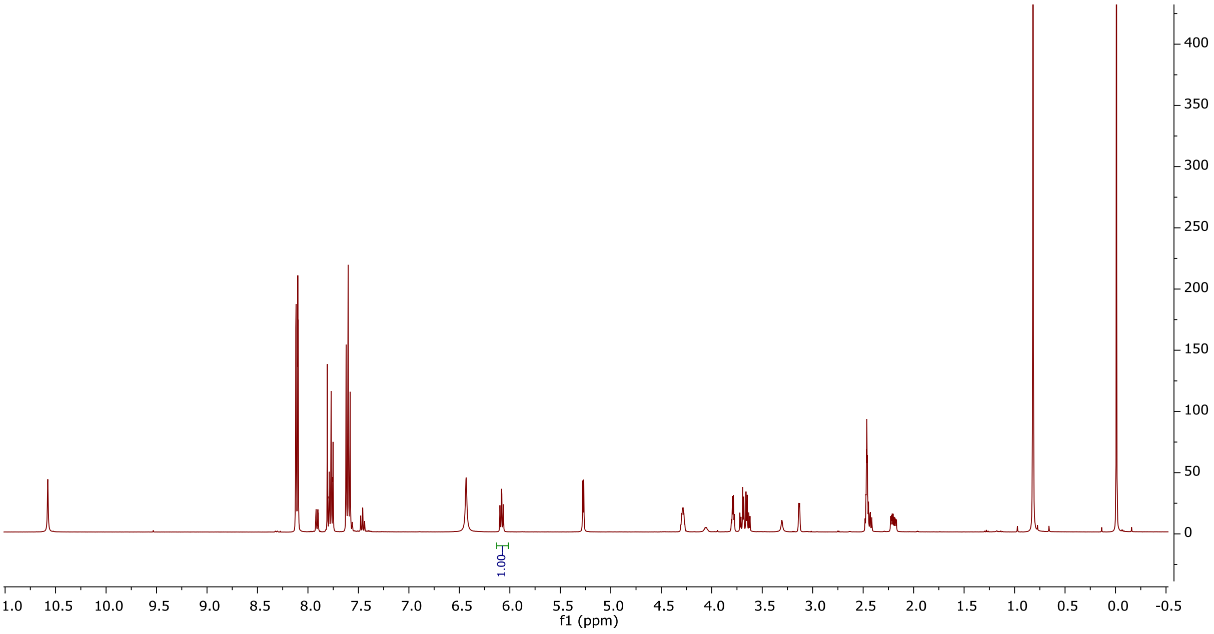

3ab (2b as the substrate, condition A, 3ab:4ab > 99:1)

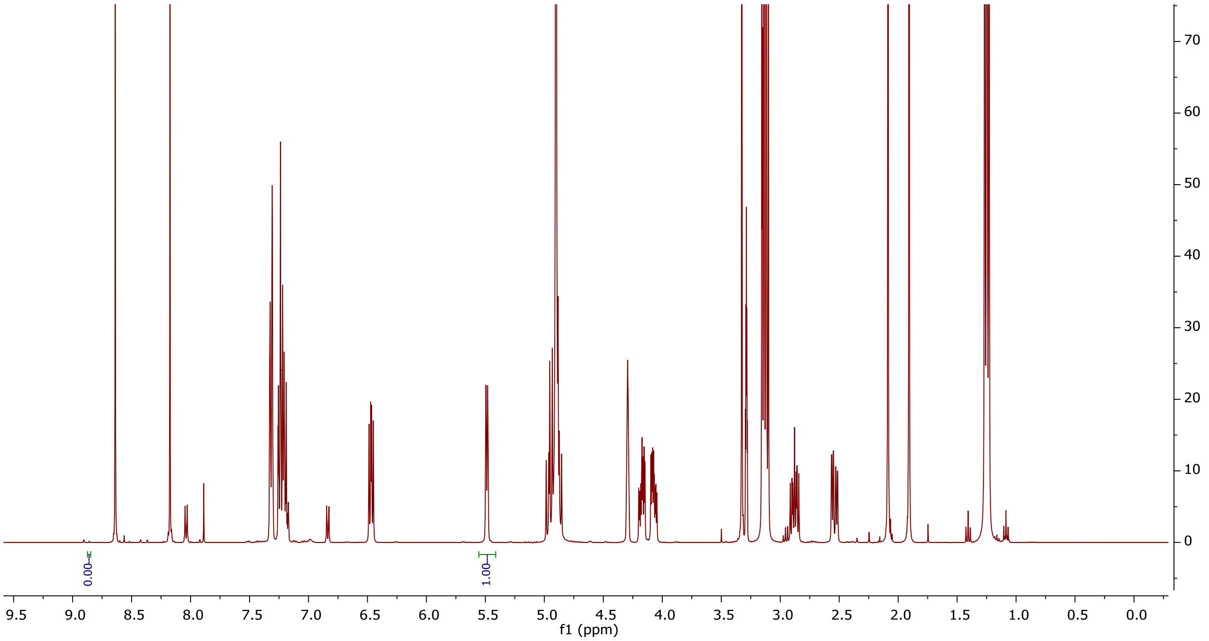

4ab (2b as the substrate, condition B, 3ab:4ab = 17:83)

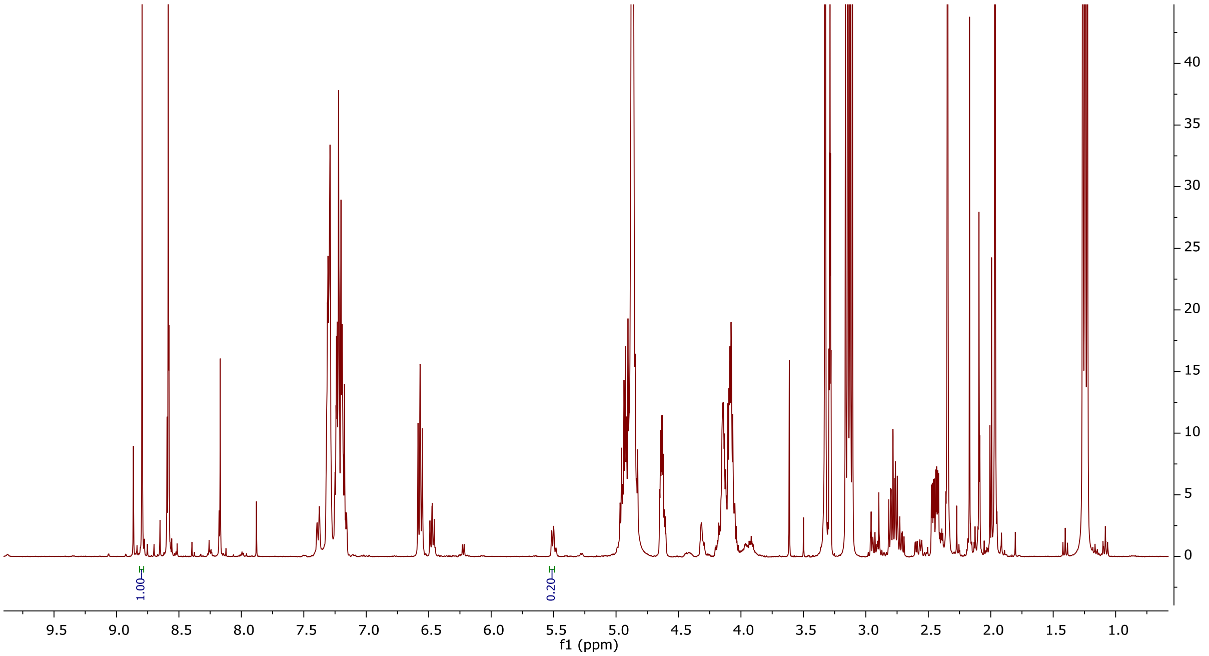

3a (2c as the substrate, condition A, 3a:4a = 91:9)

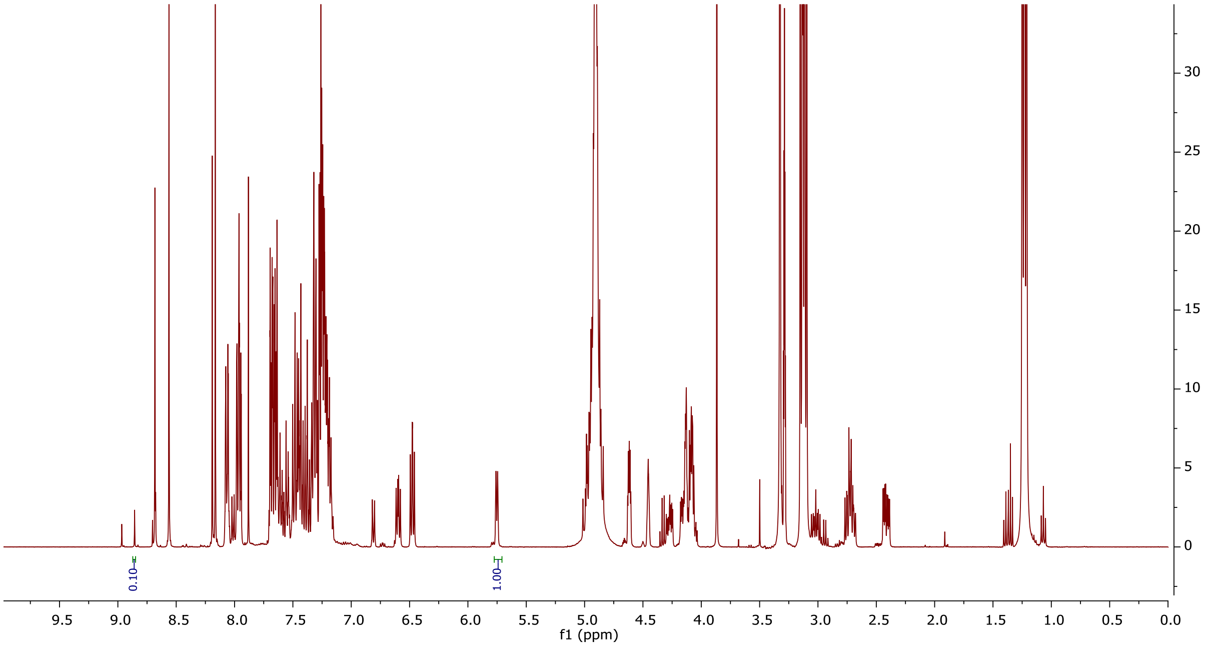

4a (2c as the substrate, condition B, 3a:4a = 8:92)

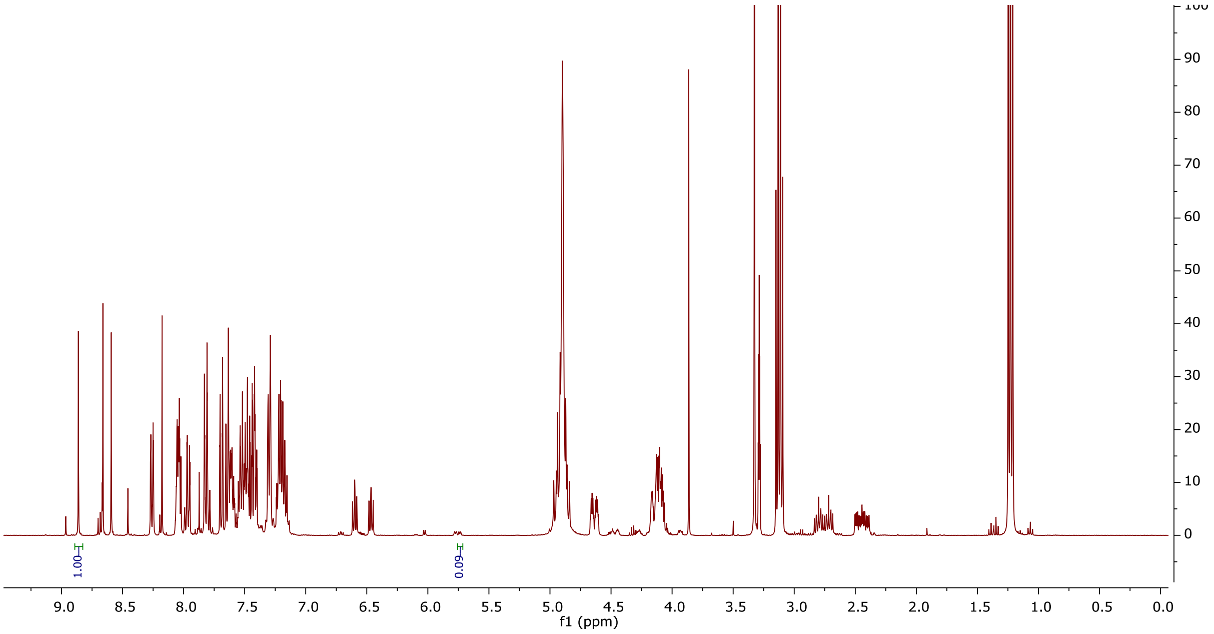

3a (2d as the substrate, condition A, < 2% conversion)

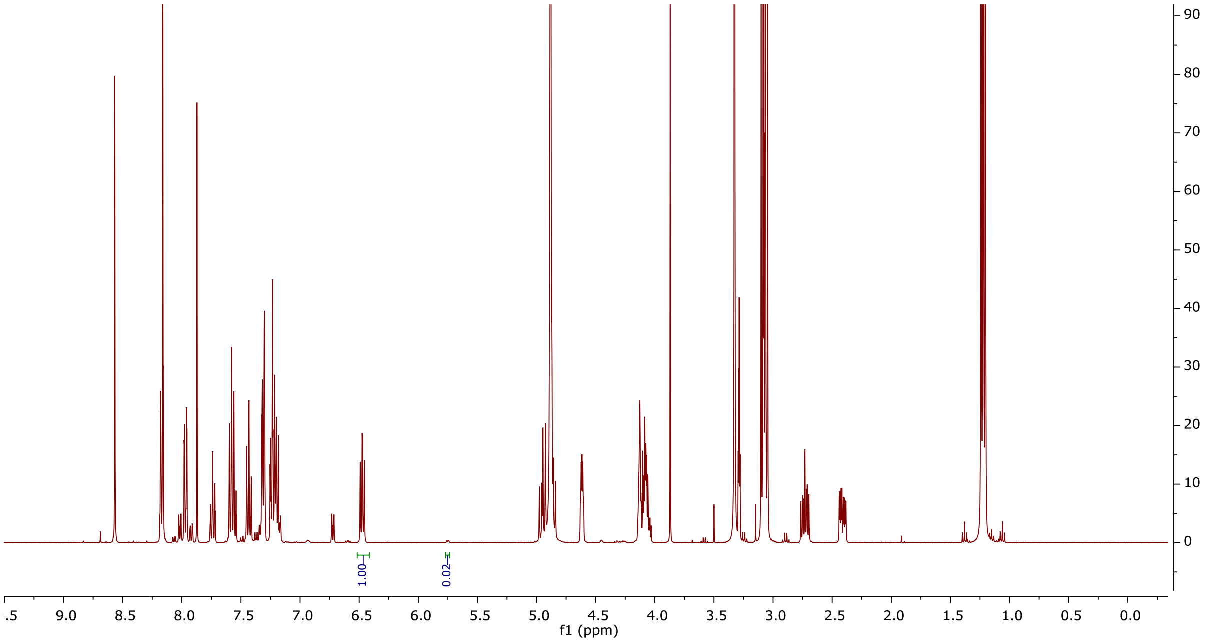

4a (2d as the substrate, condition B, < 2% conversion)

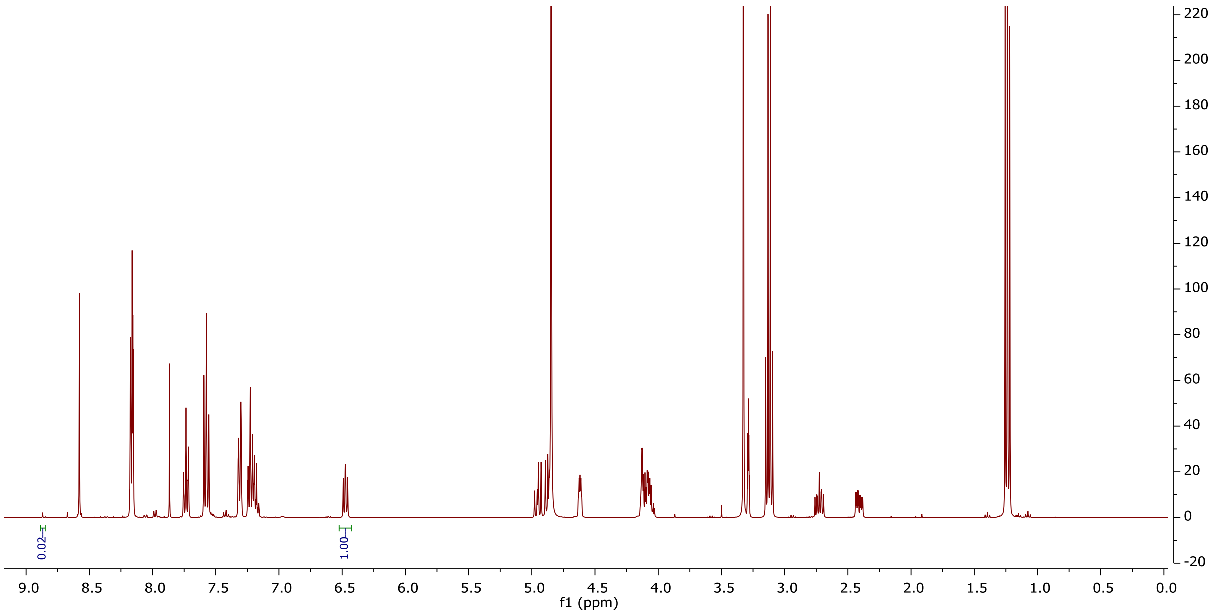

3a (2e as the substrate, condition A, 3a:4a > 99:1)

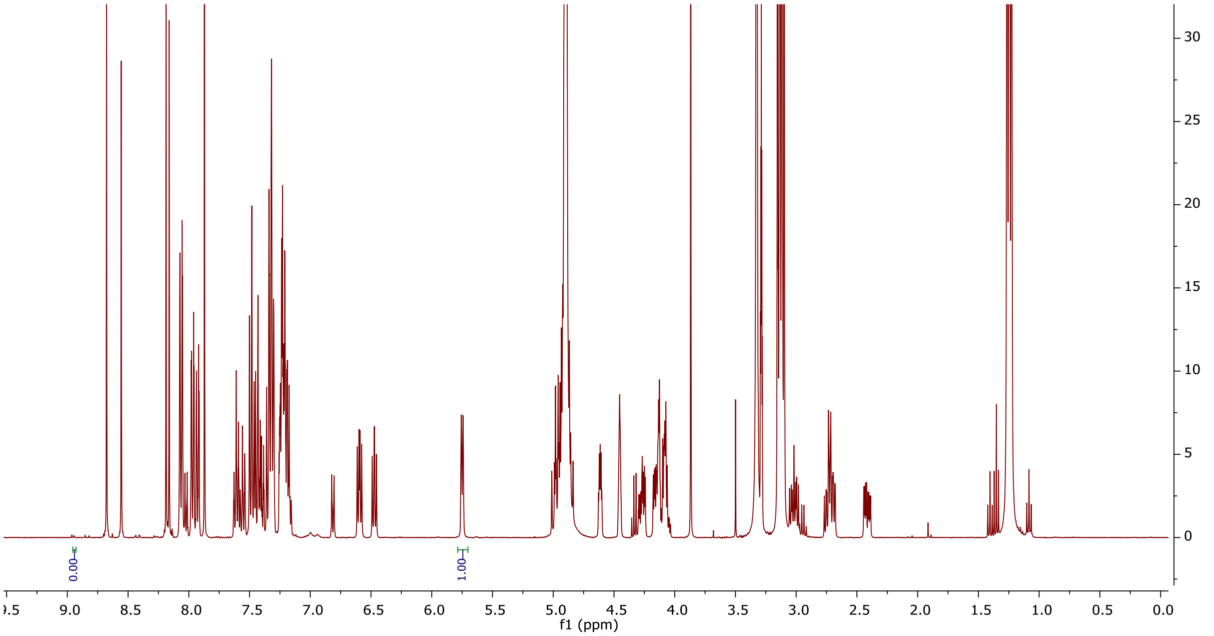

4a (2e as the substrate, condition B, 3a:4a = 1:99)

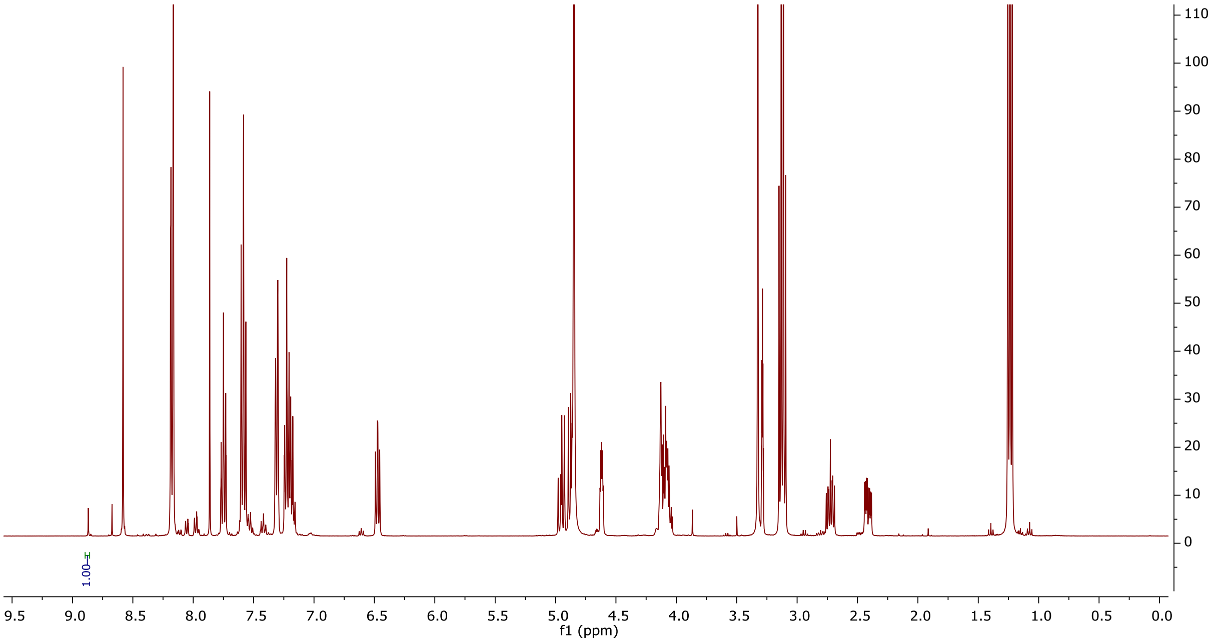

3af (2f as the substrate, condition A, 3af:4af = 97:3)

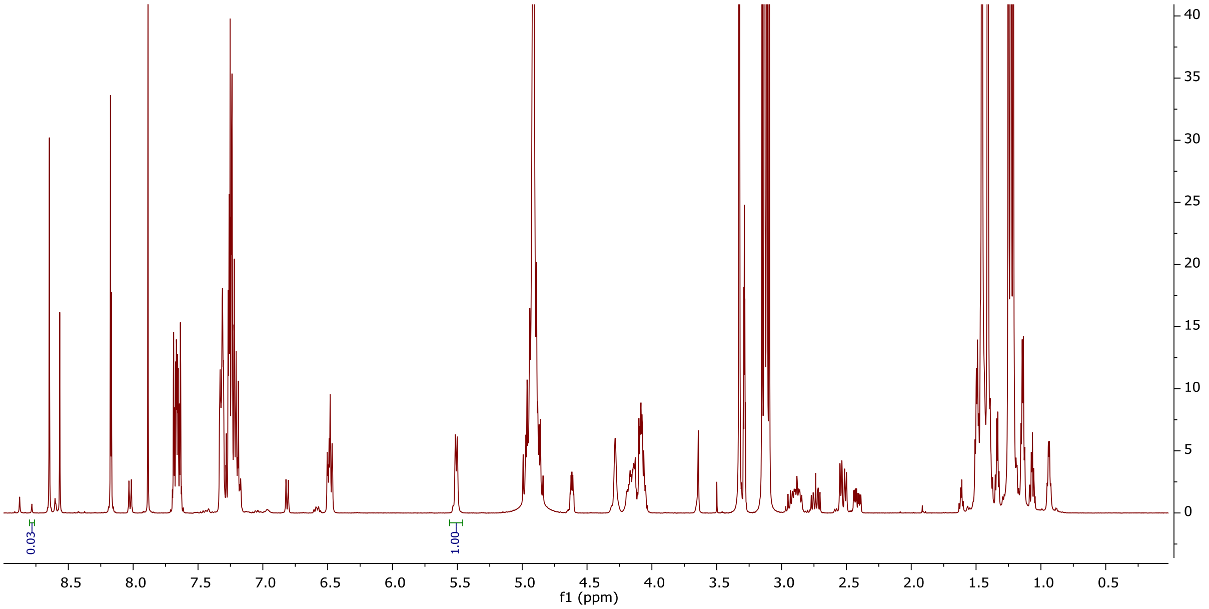

4af (2f as the substrate, condition B, 3af:4af = 12:88)

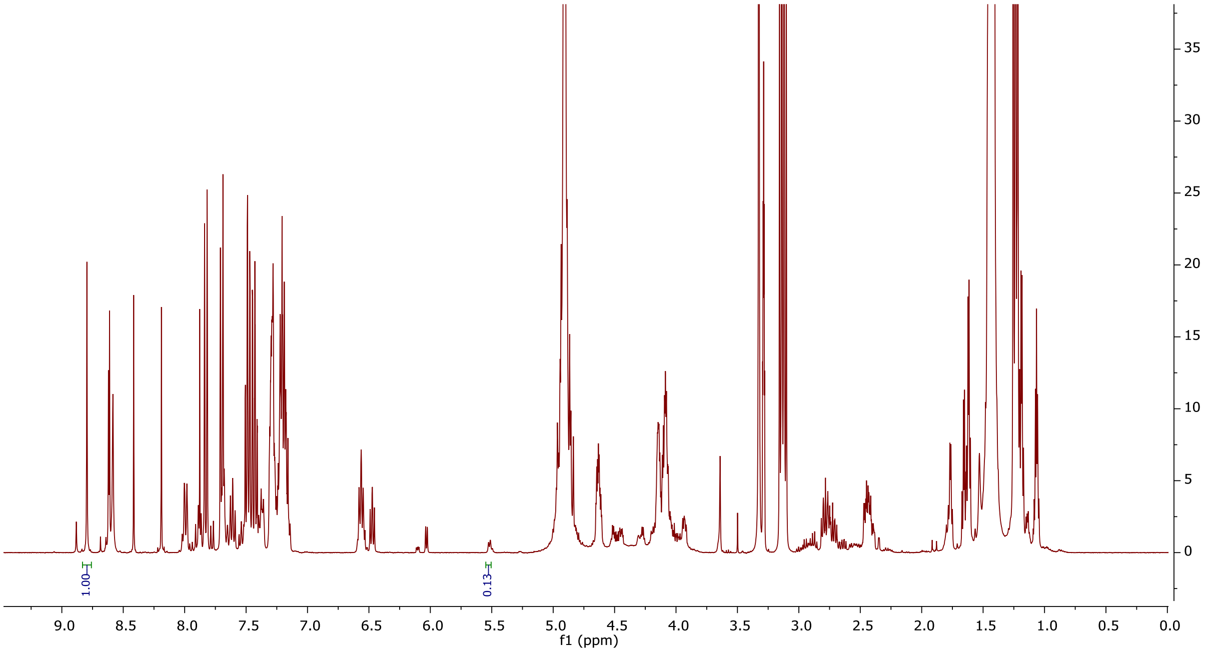

3ag (2g as the substrate, condition A, 3ag:4ag = 99:1)

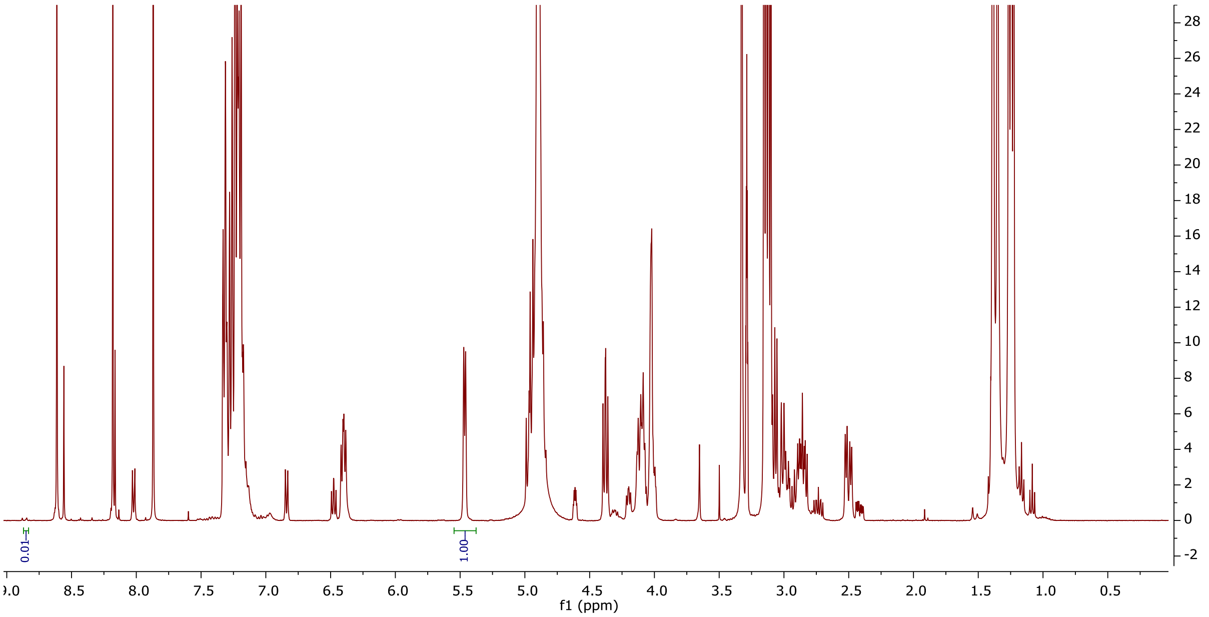

4ag (2g as the substrate, condition B, 3ag:4ag = 8:92)

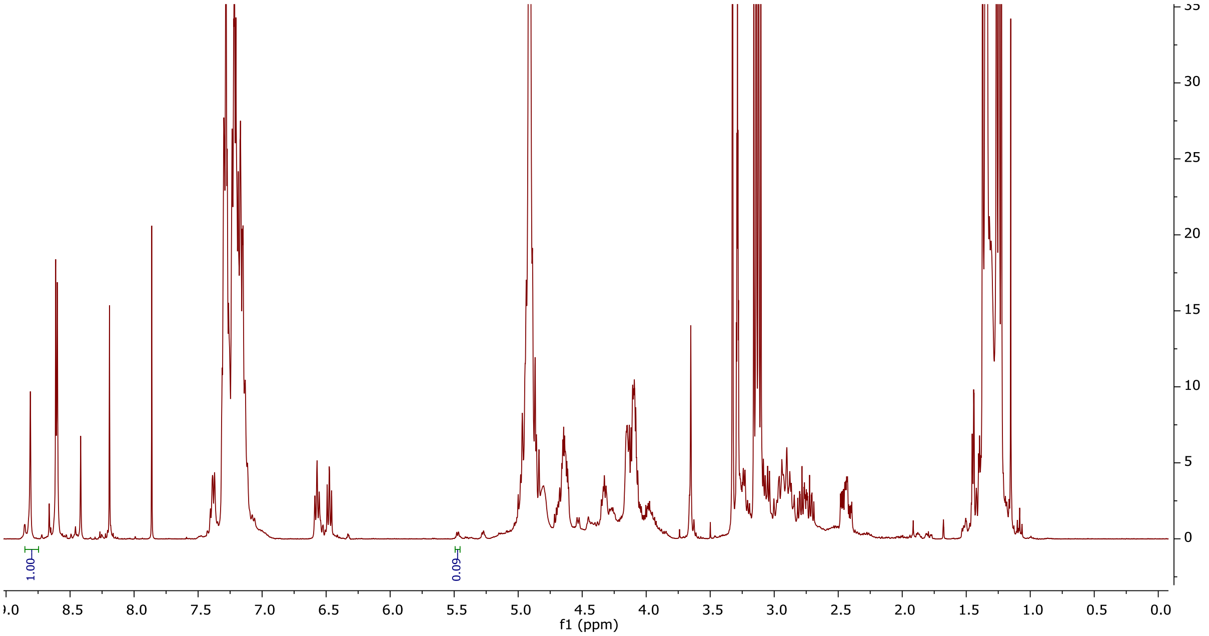

3ah (2h as the substrate, condition A, 3ah:4ah = 93:7)

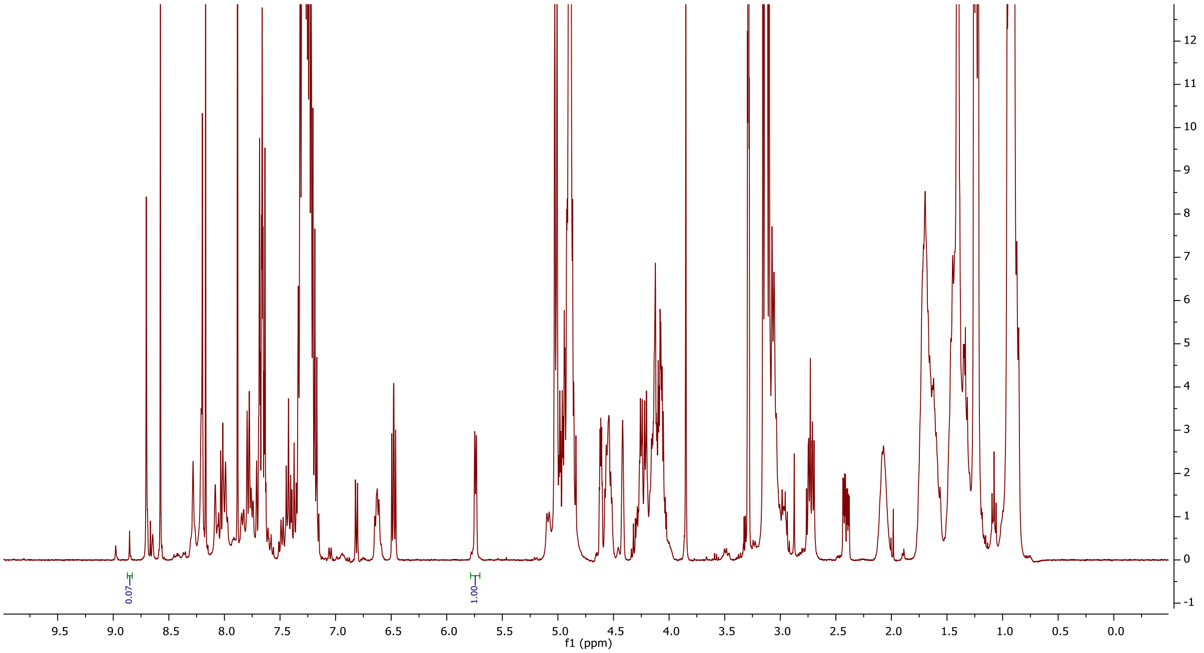

4ah (2h as the substrate, condition A, 3ah:4ah = 12:88)

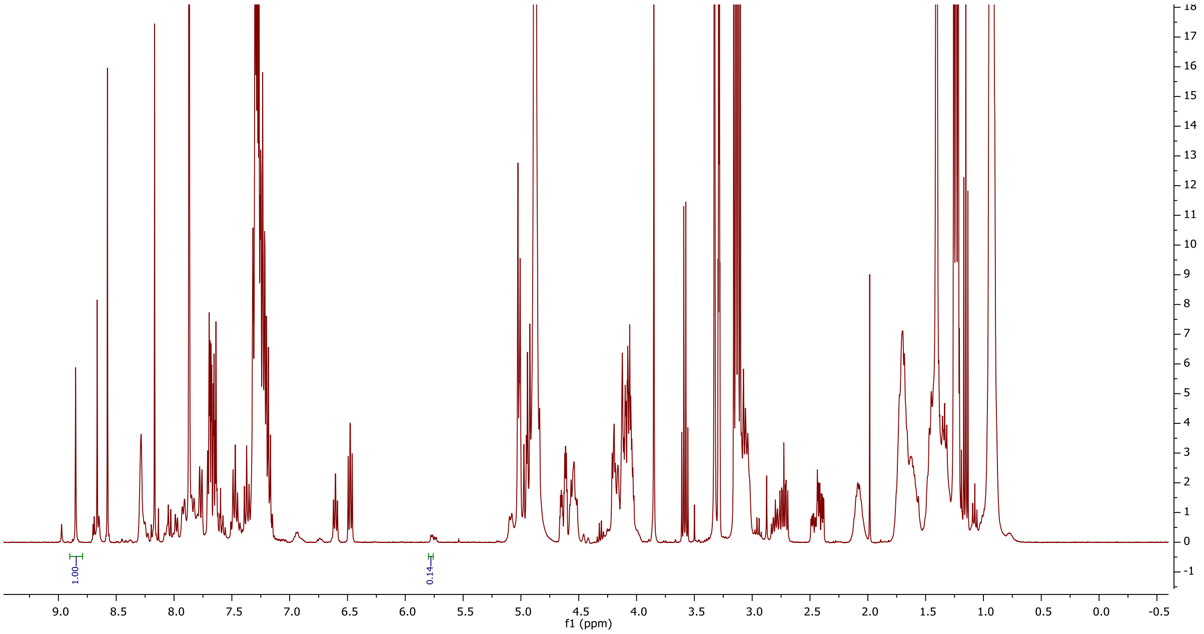

6.3 Competition experiments

Figure S3. Equation 1, condition A

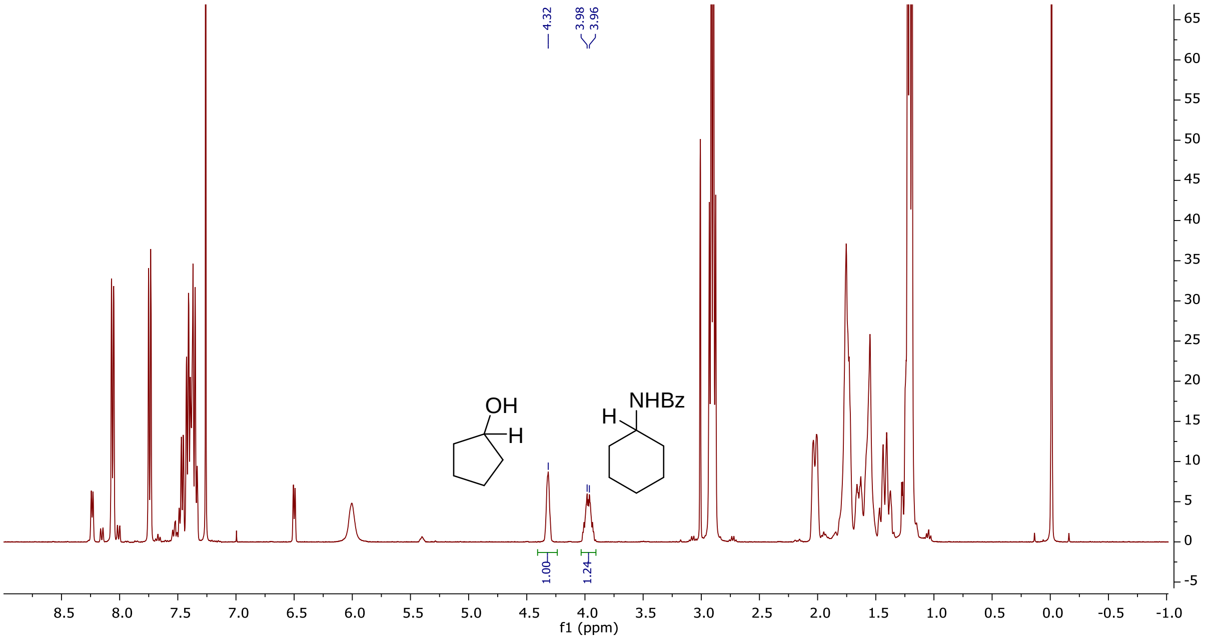

Figure S3. Equation 1, condition B

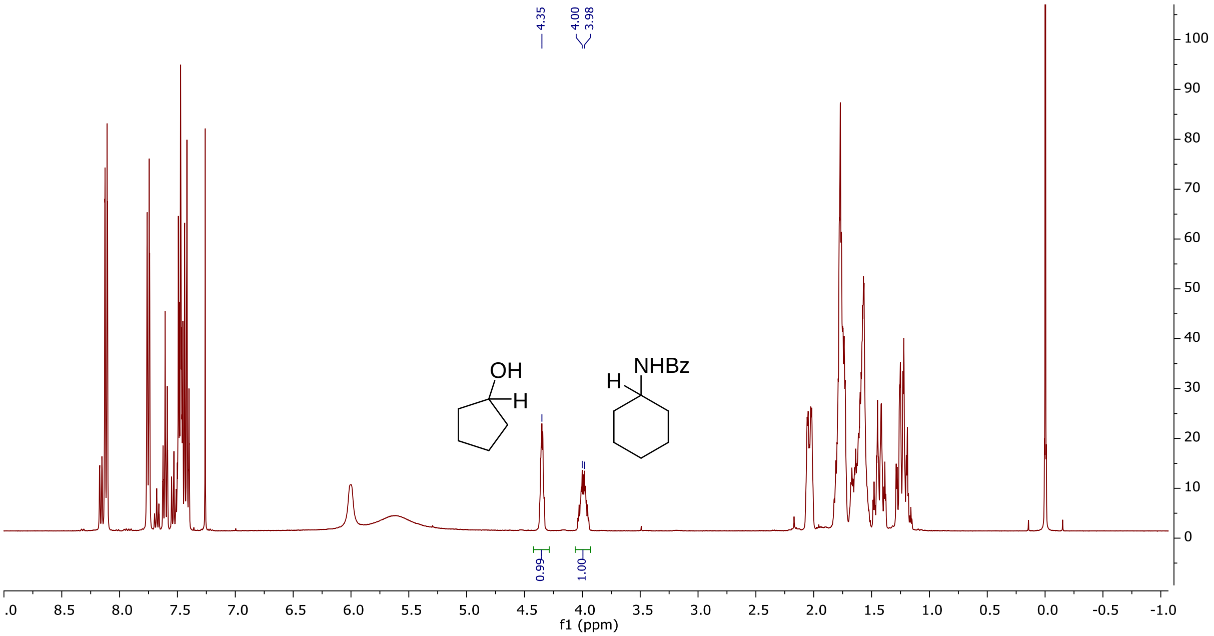

Figure S3. Equation 2, condition A

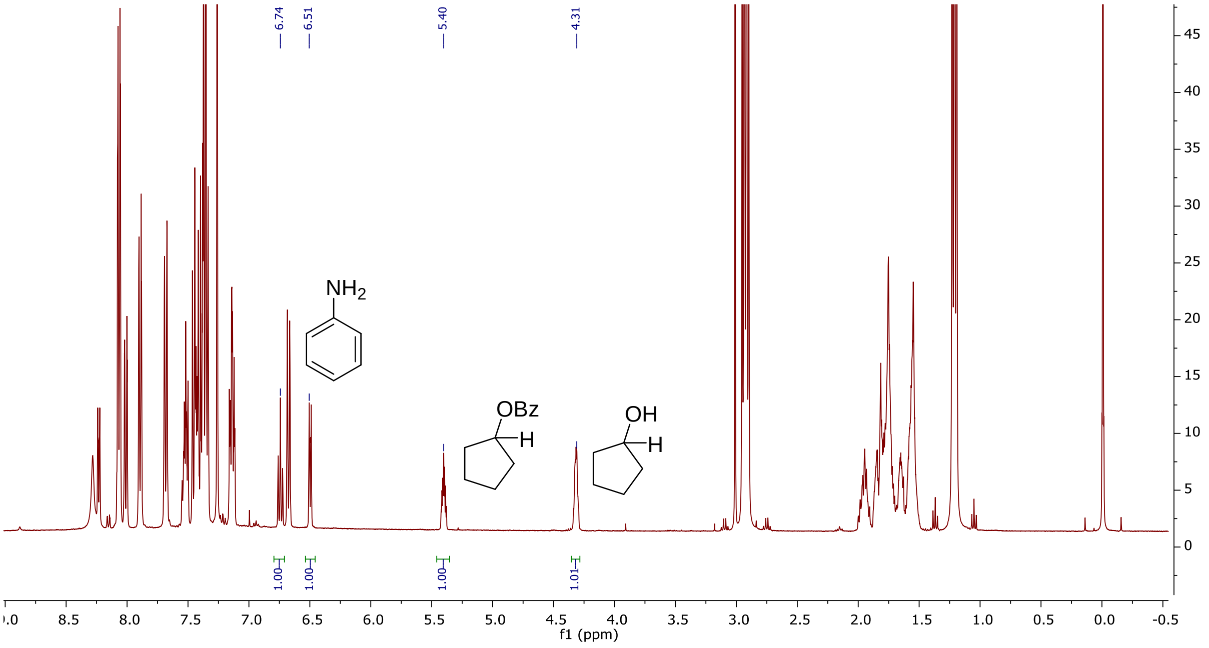

Figure S3. Equation 2, condition B

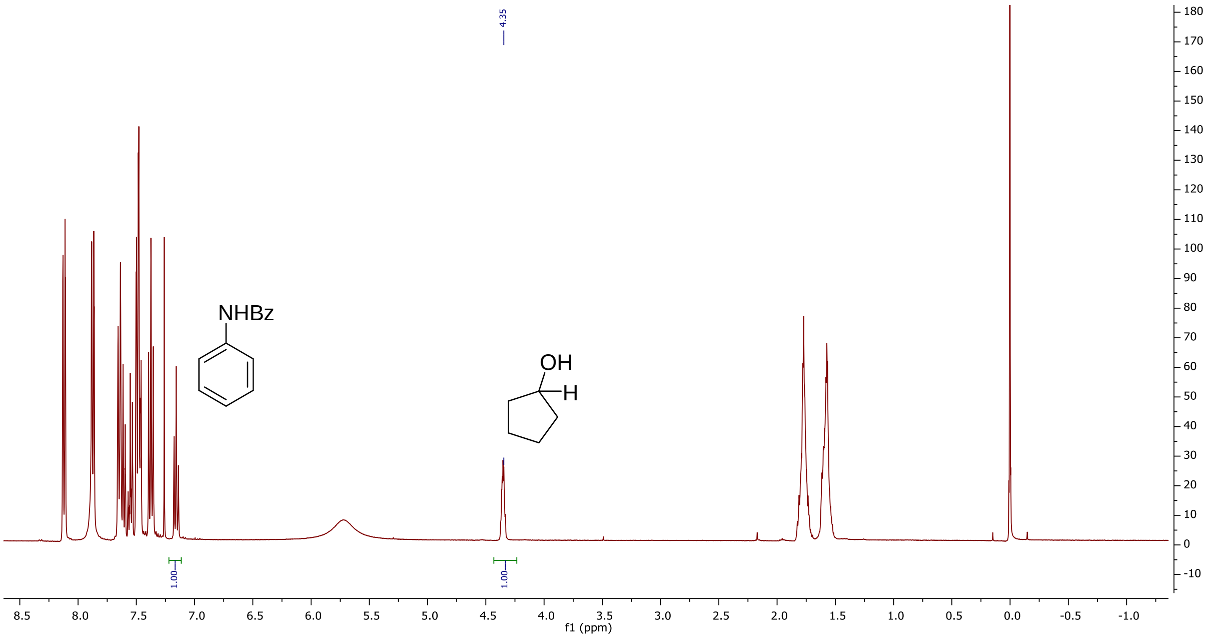

Figure S3. Equation 3, condition A

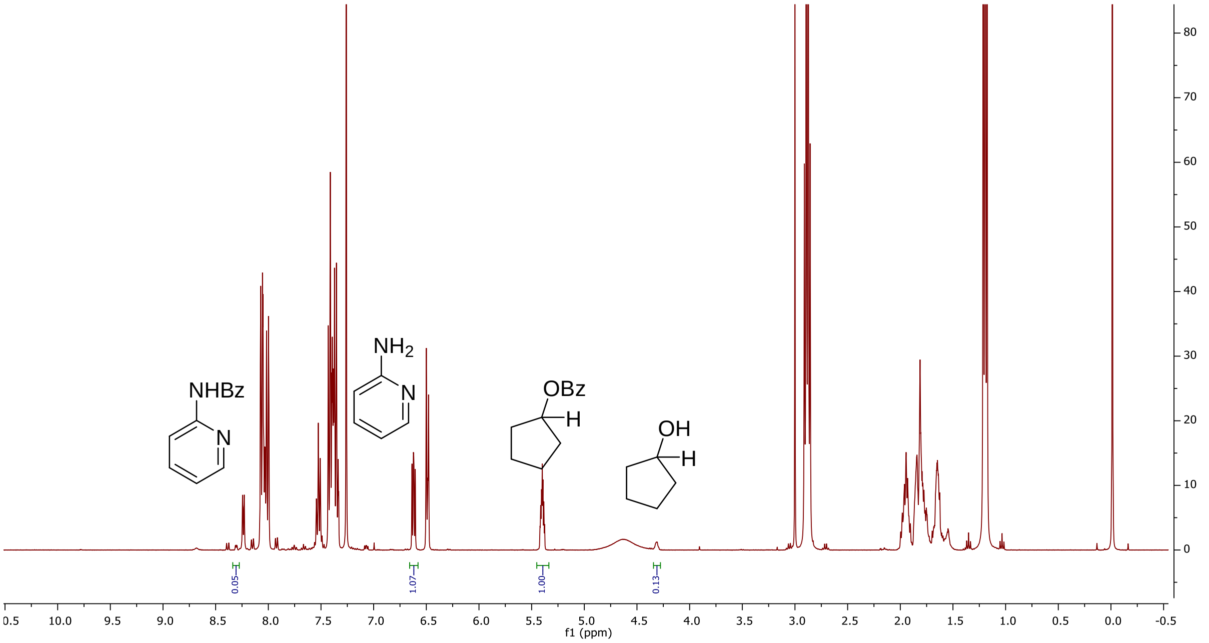

Figure S3. Equation 3, condition B

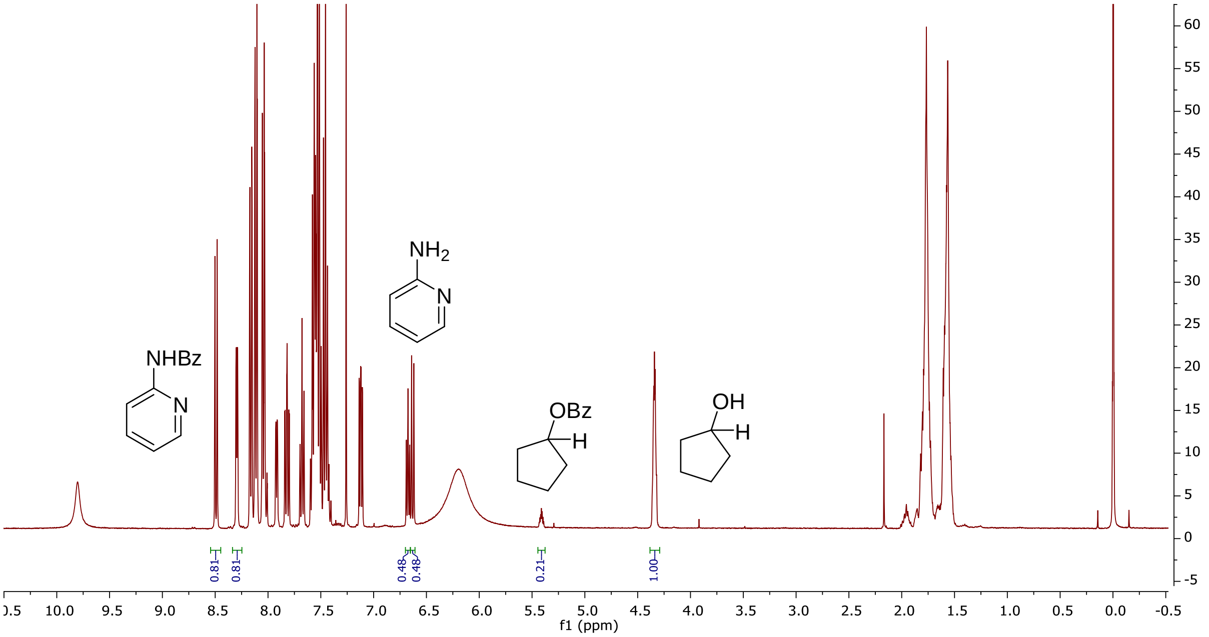

Figure S4. Equation 1, entry 1

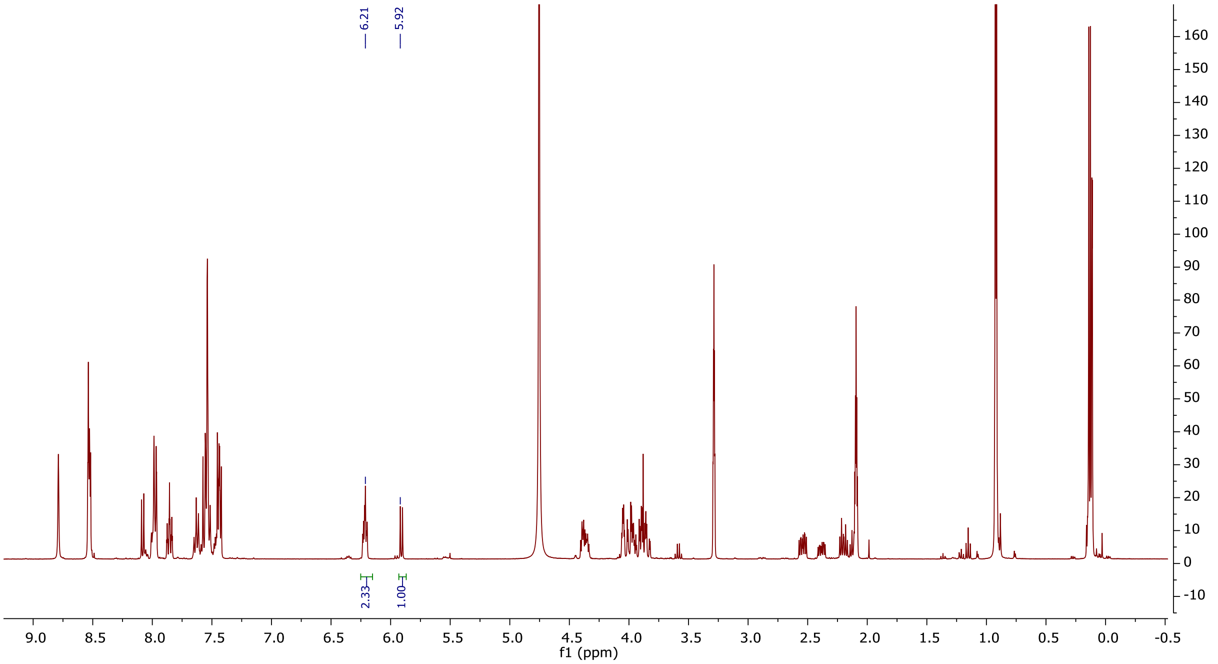

Figure S4. Equation 1, entry 2

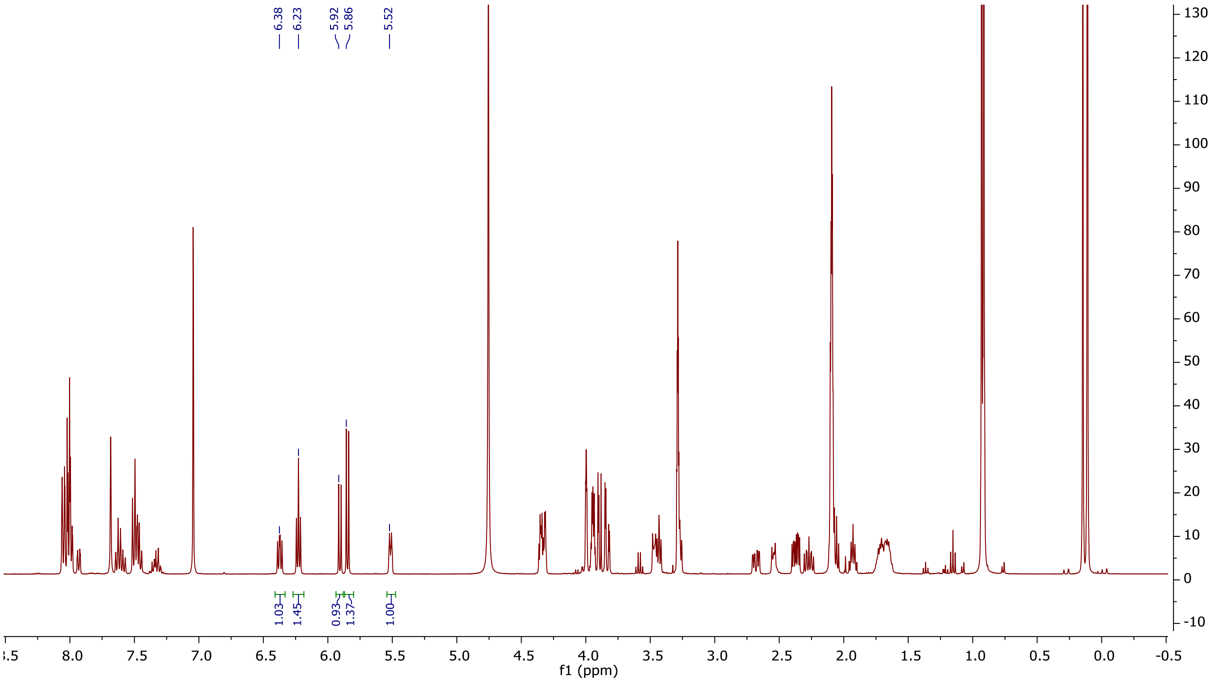

Figure S4. Equation 2, entry 1

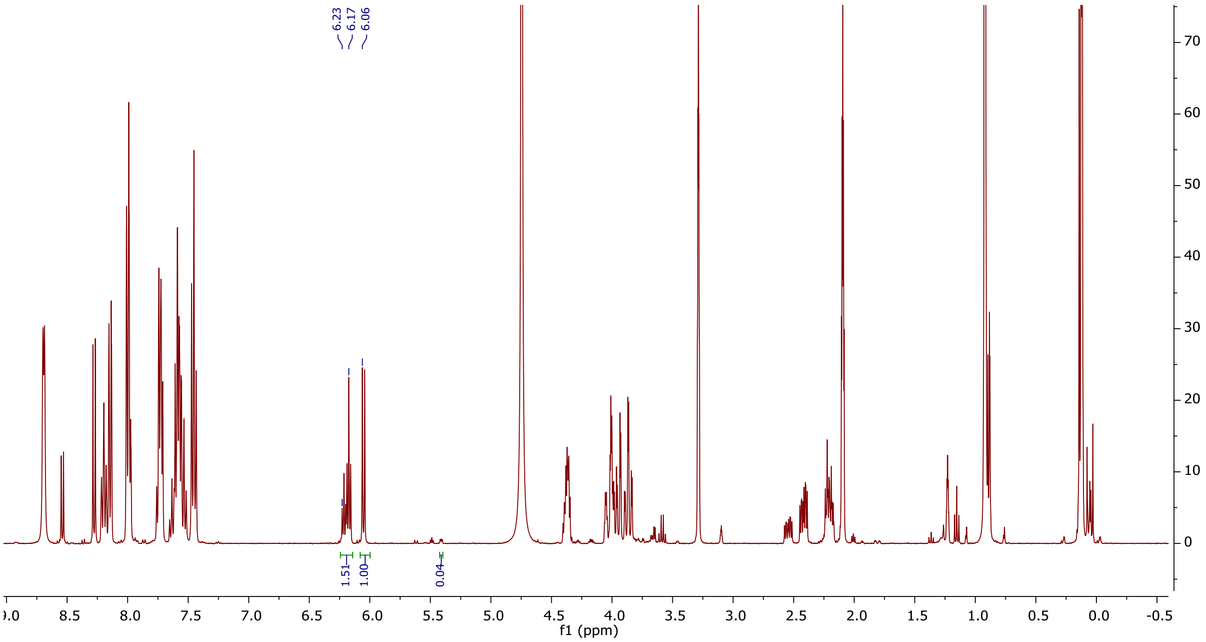

Figure S4. Equation 2, entry 2

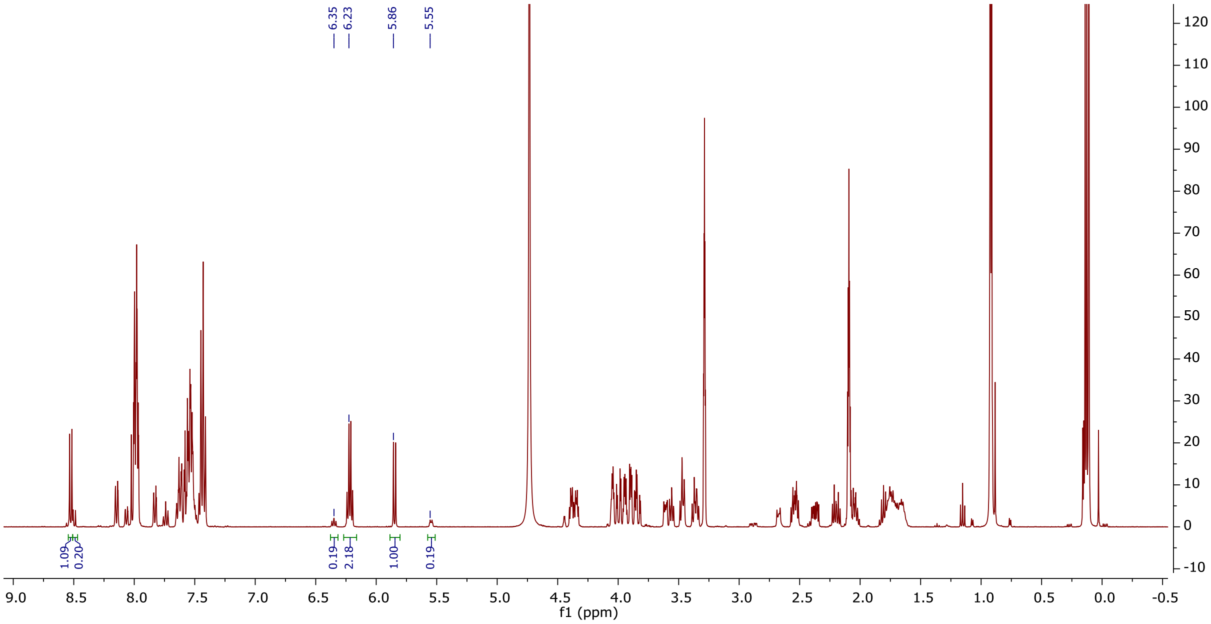

Figure S4. Equation 3, entry 1

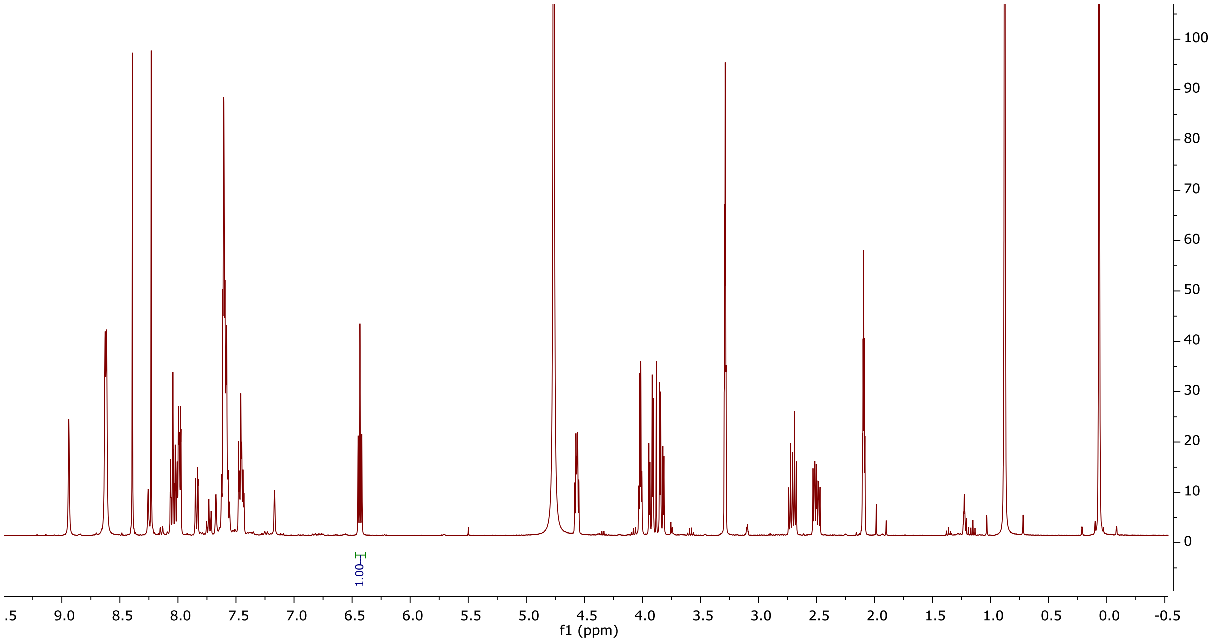

Figure S4. Equation 3, entry 2

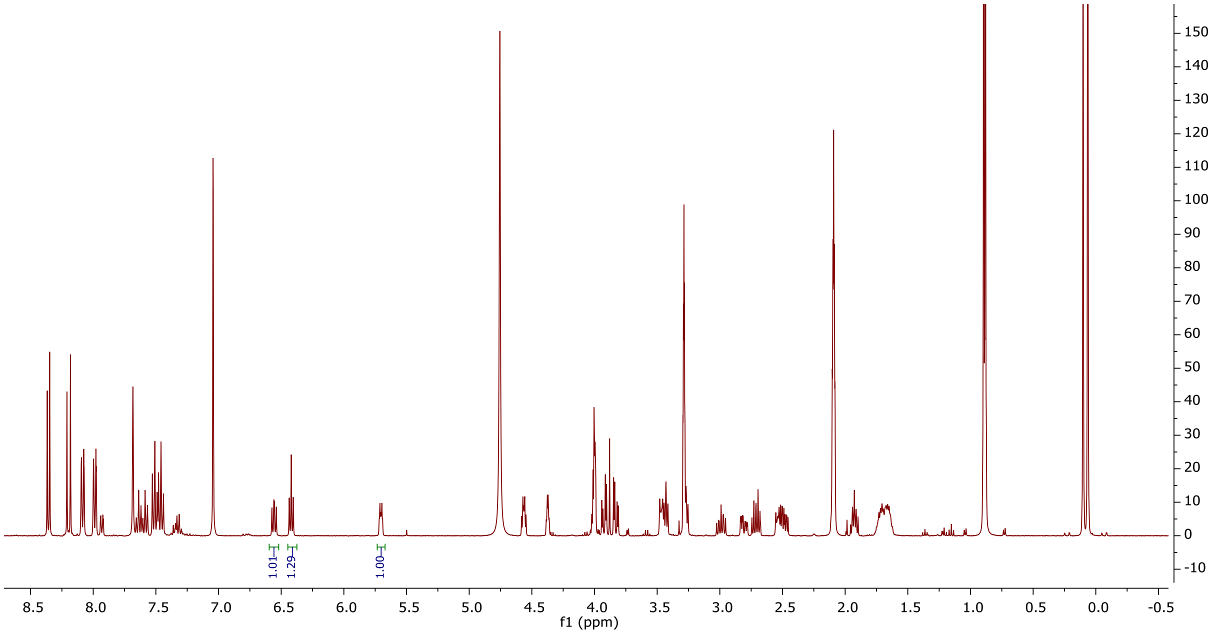

Reference

1. H. Nelson, W. Richard, H. Brown, A. Medlin, C. Light, S. T. Heller, *Angew. Chem. Int. Ed.* **2021**, *60*, 22818-22825; *Angew.Chem.* **2021**,*133*, 23000–23007.
